# Supplementary material for: Design and 22-step synthesis of highly potent D-ring modified and linker-equipped analogs of spongistatin 1
Source: Nat Commun. 2018 Nov 9;9:4710. doi: 10.1038/s41467-018-07259-x (PMC6226463; doi:10.1038/s41467-018-07259-x)
Supplement: Supplementary file 1 — Supplementary Information [file 41467_2018_7259_MOESM1_ESM.pdf]

Supplementary Information

for

Design and 22-step synthesis of highly potent D-ring modified and linker-equipped analogs of spongistatin 1

Suen *et al*.

TABLE OF CONTENTS

Supplementary Figures
- Supplementary Figure 1: Synthesis of aldehyde **14** 2
- Supplementary Figure 2: Synthesis of silyl enol ether **15** 2
- Supplementary Figure 3: Synthesis of aldehyde **9a** 3
- Supplementary Figure 4: Synthesis of diaminophenol **24** 3
- Supplementary Figure 5: Synthesis of **6a** from **21** 3
- Supplementary Figure 6: Synthesis of azido acids **S28** and **S33** 4
- Supplementary Figure 7: Synthesis of **6b** from **25** 4
- Supplementary Figure 8: Synthesis of **6c** from **25** 4
- Supplementary Figure 9: Synthesis of aldehyde **34** 5
- Supplementary Figure 10: Synthesis of **13** from **37** 5

Supplementary Methods – Experimental Procedures and Characterization Data
- General Information 6
- Synthesis of aldehyde **14** 7
- Synthesis of silyl enol ether **15** 19
- Synthesis of aldehyde **9b** from **14** and **15** 31
- Synthesis of aldehyde **9a** 39
- Synthesis of diaminophenol **24** 60
- Synthesis of **21** from **12** and **9a** 64
- Synthesis of **25** from **12** and **9b** 67
- Synthesis of **6a** from **21** 69
- Synthesis of azido acids **S28** and **S33** 77
- Synthesis of **6b** from **25** 81
- Synthesis of **6c** from **25** 87
- Synthesis of **33** 93
- Synthesis of aldehyde **34** 101
- Synthesis of **13** from **33** and **34** 107
- Synthesis of **39** from **13** and **11** 119
- Synthesis of EF fragment **7** from **39** 121
- Synthesis of **5a** from **7** and **6a** 125
- Synthesis of **5b** from **7** and **6b** 133
- Synthesis of **5c** from **7** and **6c** 142
- *In Vitro* Cell Growth Inhibition Assays
  - Assay protocols 151
  - Supplementary Table 1: Full details for assay results reported in Fig. 5b 151

Supplementary References 152

Supplementary Figures

The synthesis of aldehyde **14** is described in Supplementary Figure 1. The one pot asymmetric hydroformylation-allylation reaction to produce **S1** is modeled after a similar reaction we employed in our synthesis of zincophorin methyl ester.[1]

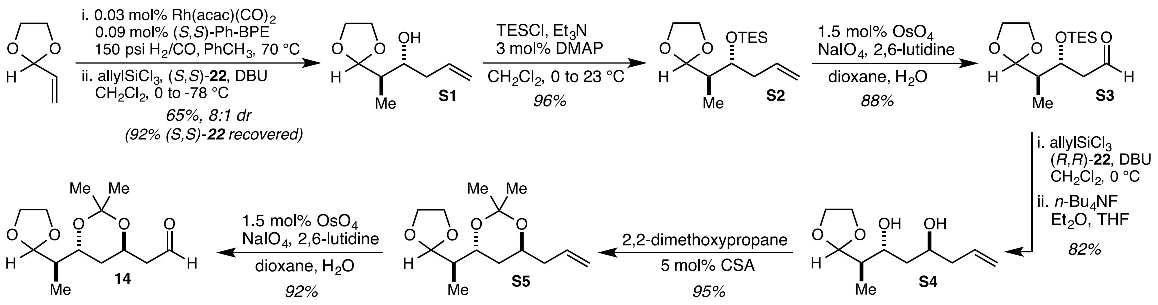

Supplementary Figure 1. Synthesis of aldehyde **14**.

The synthesis of silyl enol ether **15** is described in Supplementary Figure 2. The asymmetric alkylation to produce **S8** and the reductive auxiliary removal to produce **S9** follow the Myers protocol.[2] The Wacker oxidation of **S10** to **S11** was carried out using Sigman's method.[3]

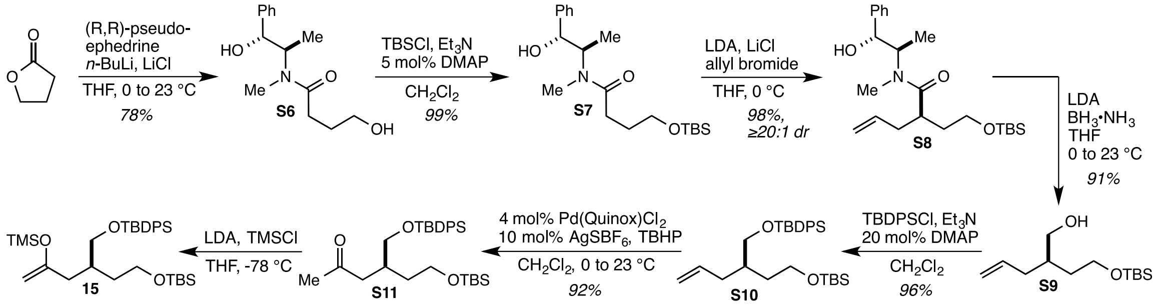

Supplementary Figure 2. Synthesis of silyl enol ether **15**.

The synthesis of aldehyde **9a** is described in Supplementary Figure 3.

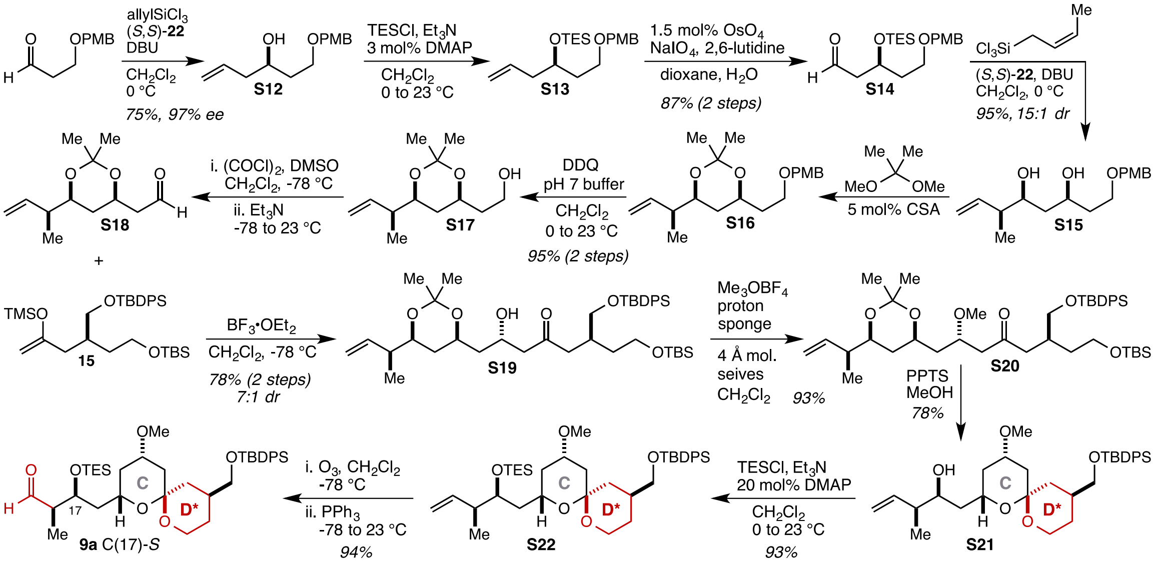

Supplementary Figure 3. Synthesis of aldehyde **9a**.

The synthesis of diaminophenol ligand **24** is described in Supplementary Figure 4.

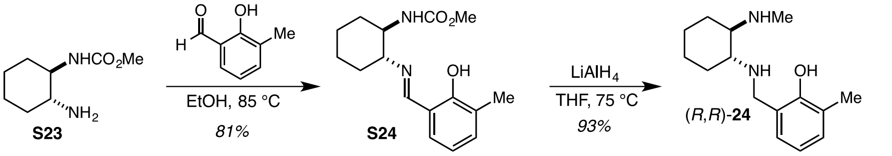

Supplementary Figure 4. Synthesis of diaminophenol **24**.

The synthesis of **6a** from **21** is described in Supplementary Figure 5.

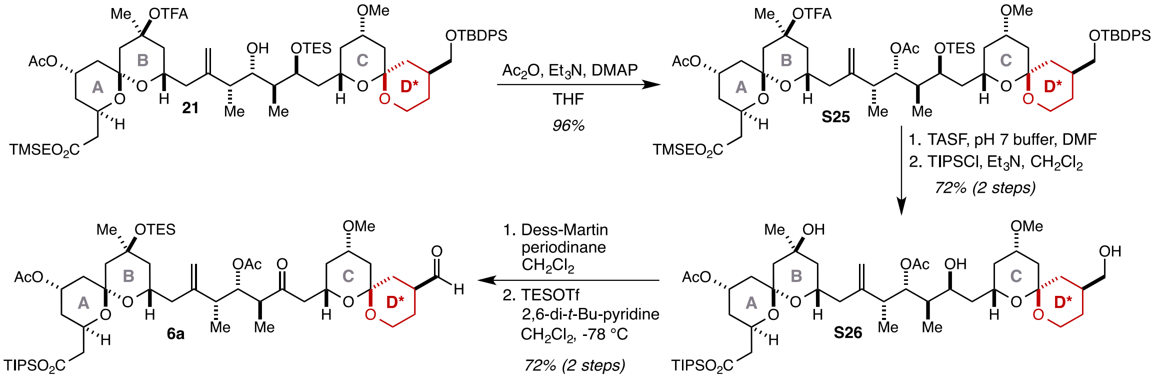

Supplementary Figure 5. Synthesis of **6a** from **21**

The syntheses of azido acids **S28** and **S33** are described in Supplementary Figure 6.

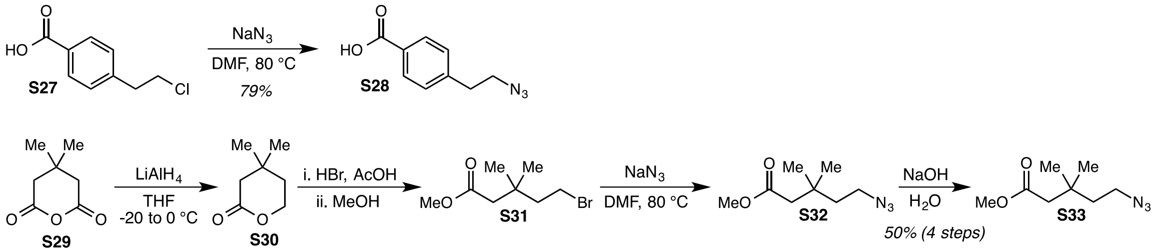

Supplementary Figure 6. Synthesis of azido acids **S28** and **S33**.

The synthesis of **6b** from **25** and **S28** is described in Supplementary Figure 7.

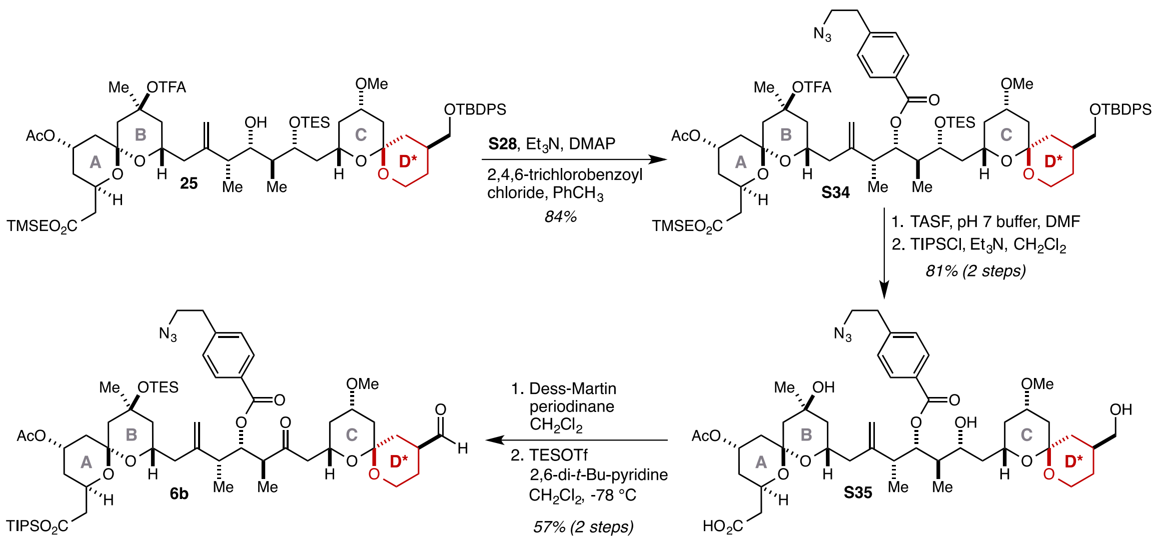

Supplementary Figure 7. Synthesis of **6b** from **25**.

The synthesis of **6c** from **25** and **S33** is described in Supplementary Figure 8.

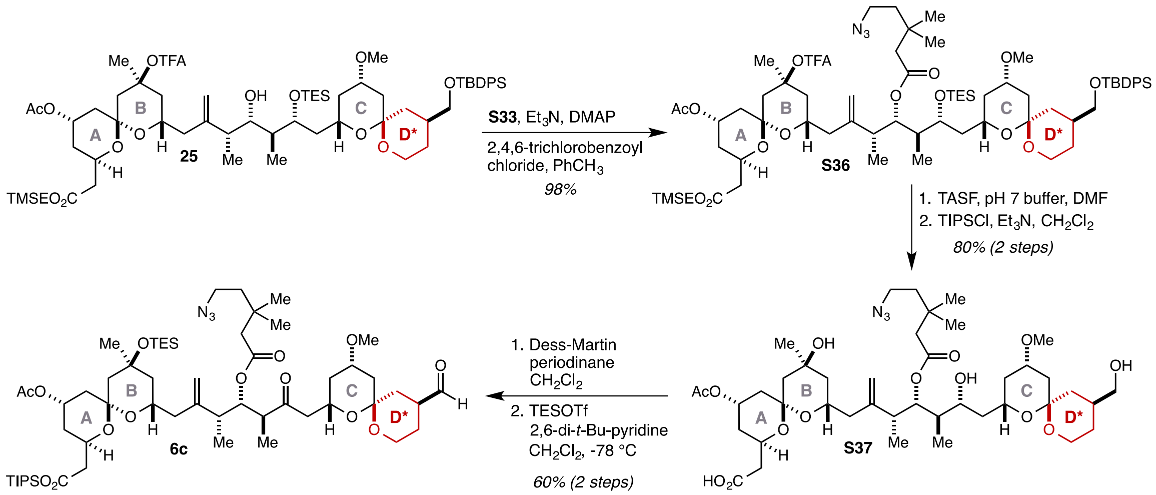

Supplementary Figure 8. Synthesis of **6c** from **25**.

The synthesis of aldehyde **34** is described in Supplementary Figure 9.

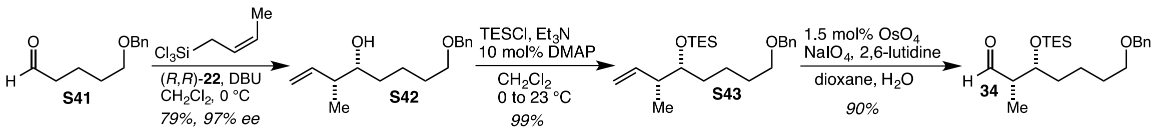

Supplementary Figure 9. Synthesis of aldehyde **34**.

The synthesis of dichloride **13** from **37** is described in Supplementary Figure 10.

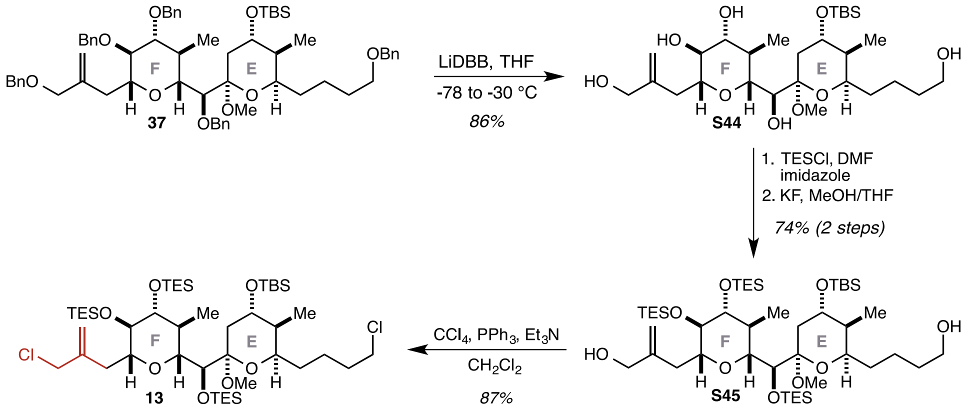

Supplementary Figure 10. Synthesis of **13** from **37**.

Supplementary Methods – Experimental Procedures and Characterization Data

General Information

All reactions were carried out under an atmosphere of nitrogen in flame-dried glassware with magnetic stirring unless otherwise indicated. Degassed solvents were purified by passage through an activated alumina column. Thin-layer chromatography (TLC) was carried out on glass backed silica gel TLC plates (250 μm) from Silicycle; visualization by UV light, cerium ammonium molybdate (CAM), phosphomolybdic acid (PMA), p-anisaldehyde stain, or potassium permanganate (KMnO4) stain. HPLC analysis was carried out on an Agilent 1200 Series using a Chiralpak OD-H (250 × 4.5 mm ID) column. Diastereomeric ratios for all compounds were determined by ^{1}H NMR analysis of the unpurified reaction mixtures. ^{1}H NMR spectra were recorded on a Bruker AVIII 400 (400 MHz) and AVIII 500 (500 MHz) spectrometer and are reported in ppm, relative to residual protonated solvent peak ($CDCl_3$, 7.26 ppm) unless otherwise indicated. Data are reported as follows: (bs= broad singlet, s = singlet, d = doublet, t = triplet, q = quartet, m = multiplet, dd = doublet of doublets, ddd = doublet of doublet of doublets, ddt = doublet of doublet of triplets, td = triplet of doublets; coupling constant(s) in Hz; integration). Proton decoupled ^{13}C NMR spectra were recorded on a Bruker AVIII 400 (100 MHz) and AVIII 500 (125 MHz) spectrometer and are reported in ppm from $CDCl_3$ internal standard (77.00 ppm) unless otherwise indicated. High-resolution mass spectra were obtained from the Columbia University Mass Spectrometry Facility on a Waters XEVO G2XS QToF mass spectrometer equipped with a UPC2 SFC inlet, electrospray ionization (ESI) probe, atmospheric pressure chemical ionization (APCI) probe. Infrared spectra were recorded on a Perkin Elmer Paragon 1000 FT-IR spectrometer. Optical rotations were recorded on a Jasco DIP-1000 digital polarimeter. pH 7.00 buffered silica gel was prepared as follows: A 1000 mL round bottom flask charged with silica gel (250 g) and 25.0 mL of pH 7.00 buffer solution (potassium dihydrogen phosphate/sodium hydroxide, Fluka Analytical) was stirred on rotavapor under atmospheric pressure overnight.

SAFETY STATEMENT: No unexpected or unusually high safety hazards were encountered.

Synthesis of aldehyde **14**

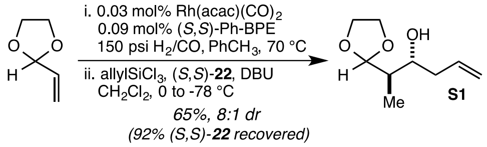

Supplementary Figure 11. Synthesis of **S1**.

To a metal cylinder of a Parr bomb (without a glass liner) was added 2-vinyl-1,3-dioxolane (1.50 mL, 15.0 mmol) in toluene (3.50 mL), Rh(acac)$(CO)_2$ (1.16 mg, 0.450 mmol), and (S,S)-Ph-BPE (6.83 mg, 1.35 mmol).[4] The Parr bomb was charged to 500 psi with H_2/CO (1:1) and vented (repeated 5 times). The bomb was then pressurized to 150 psi and heated to 70 °C (oil bath, external temperature. After 12 h, the bomb was allowed to cool to room temperature and de-pressurized. Analysis of an aliquot by ^{1}H NMR spectroscopy indicated full conversion of the dioxolane and a 9:1 branched to linear aldehyde ratio. This solution was used directly in the allylation step without purification.

To a cooled (0 °C) solution of (*S,S*)-**22**[5,6] (4.79 g, 16.5 mmol) in CH_2Cl_2 (50.0 mL) was added DBU (7.40 mL, 49.5 mmol) and allyltrichlorosilane (2.61 mL, 18.0 mmol). The reaction mixture was allowed to warm to room temperature for 1 h. The reaction mixture was cooled to -78 °C, and the solution of the aldehyde from above was added, rinsing with CH_2Cl_2 (5.0 mL). After 3h, the reaction mixture was quenched by the addition of *n*-Bu_4NF (4.73 g, 15.0 mmol). After 1h, the reaction mixture was concentrated to dryness and re-suspended in Et_2O (60.0 mL) in order to fully precipitate the DBU•HCl salts. The mixture was filtered, concentrated, and purified by silica gel flash column chromatography 0:100 → 20:80 EtOAc:Hexanes to afford alcohol **S1** (1.70 g, 65% yield, 8:1 dr) as a colorless oil. The moderate diastereoselectivity reflects the only moderately enantioselective hydroformylation reaction. The mixture was carried forward and the minor diastereomer could be separated at the stage of intermediate **S5**.

Recovery of (*S,S*)-**22**: After elution of **S1**, the chromatography column was flushed with CH_2Cl_2/MeOH/Et_3N (100:5:1) and the filtrate was concentrated to give a beige solid. The residue was dissolved in minimal boiling hexanes (about 70 °C), and the resulting solution was allowed to cool to room temperature slowly. The resulting white solid was collected by filtration through a frit, with cold hexane rinses, and then dried *in vacuo* (with gentle warming with an oil bath set to 40 °C) to give recovered diaminophenol (*S,S*)-**22** (4.42 g, 92% yield) as white crystals.

OR $[\alpha]_D^{23}$ -1.8° (*c* 1.0, $CHCl_3$)

IR (ATR) 3502, 3013, 2889, 1640, 1462, 1403, 1215, 1103, 1043, 993, 920 cm^{-1}

^{1}H NMR (500 MHz, CDCl3) δ 5.92 (dddd, *J* = 16.8, 10.3, 7.8, 6.2 Hz, 1H), 5.23 – 5.02 (m, 2H), 4.91 (d, *J* = 4.5 Hz, 1H), 4.05 – 3.95 (m, 2H), 3.94 – 3.83 (m, 2H), 3.73 (td, *J* = 7.8, 3.6 Hz, 1H), 3.28 – 3.01 (m, 1H), 2.41 (dddt, *J* = 14.1, 6.5, 3.4, 1.5 Hz, 1H), 2.25 – 2.14 (m, 1H), 1.89 (pd, *J* = 7.1, 4.5 Hz, 1H), 0.96 (d, *J* = 7.0 Hz, 3H)

^{13}C NMR (125 MHz, CDCl3) δ 135.0, 117.6, 106.7, 71.9, 65.1, 64.9, 41.4, 39.0, 11.3

HRMS (ASAP+) calculated for $C_9H_{16}O_3$ $[M+H]^+$: 172.1099; found 172.1095

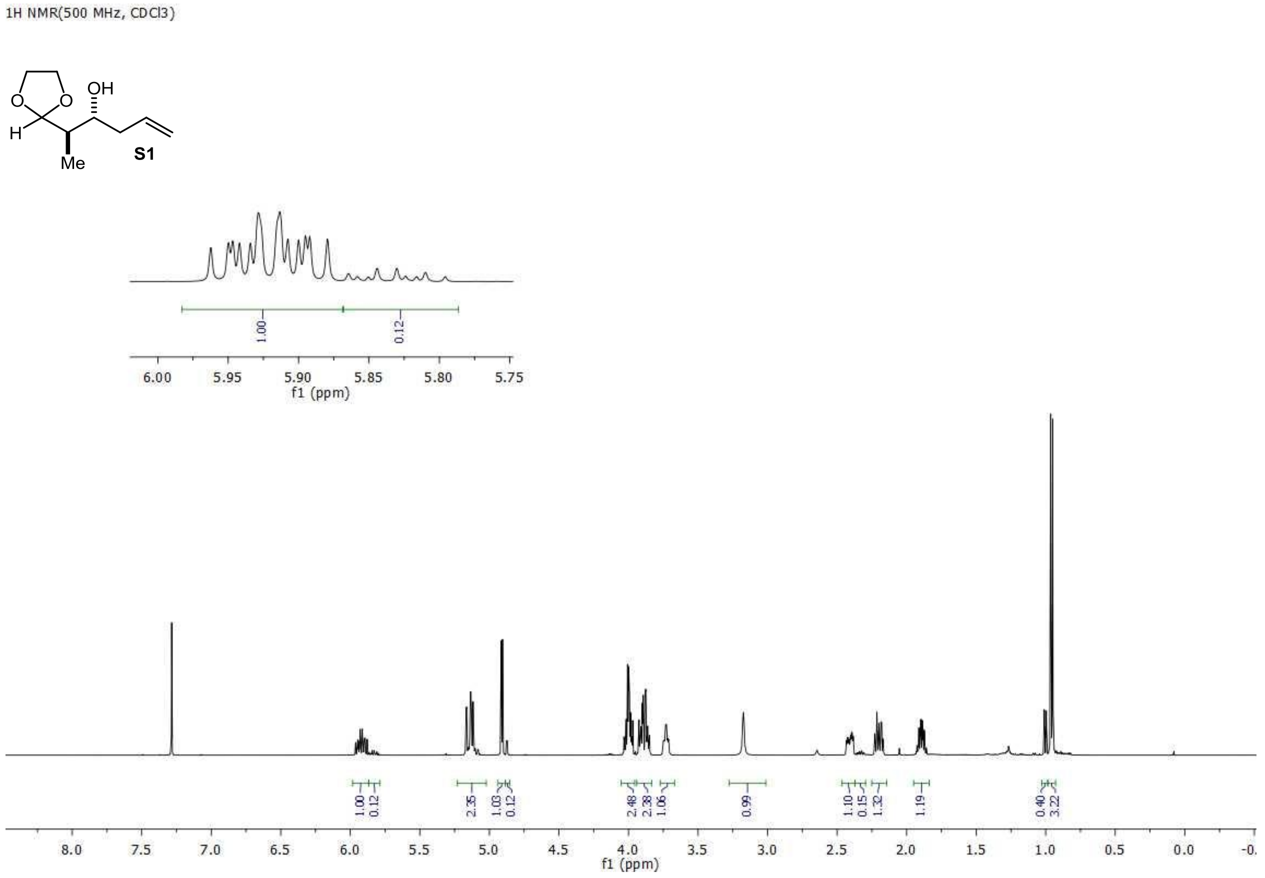

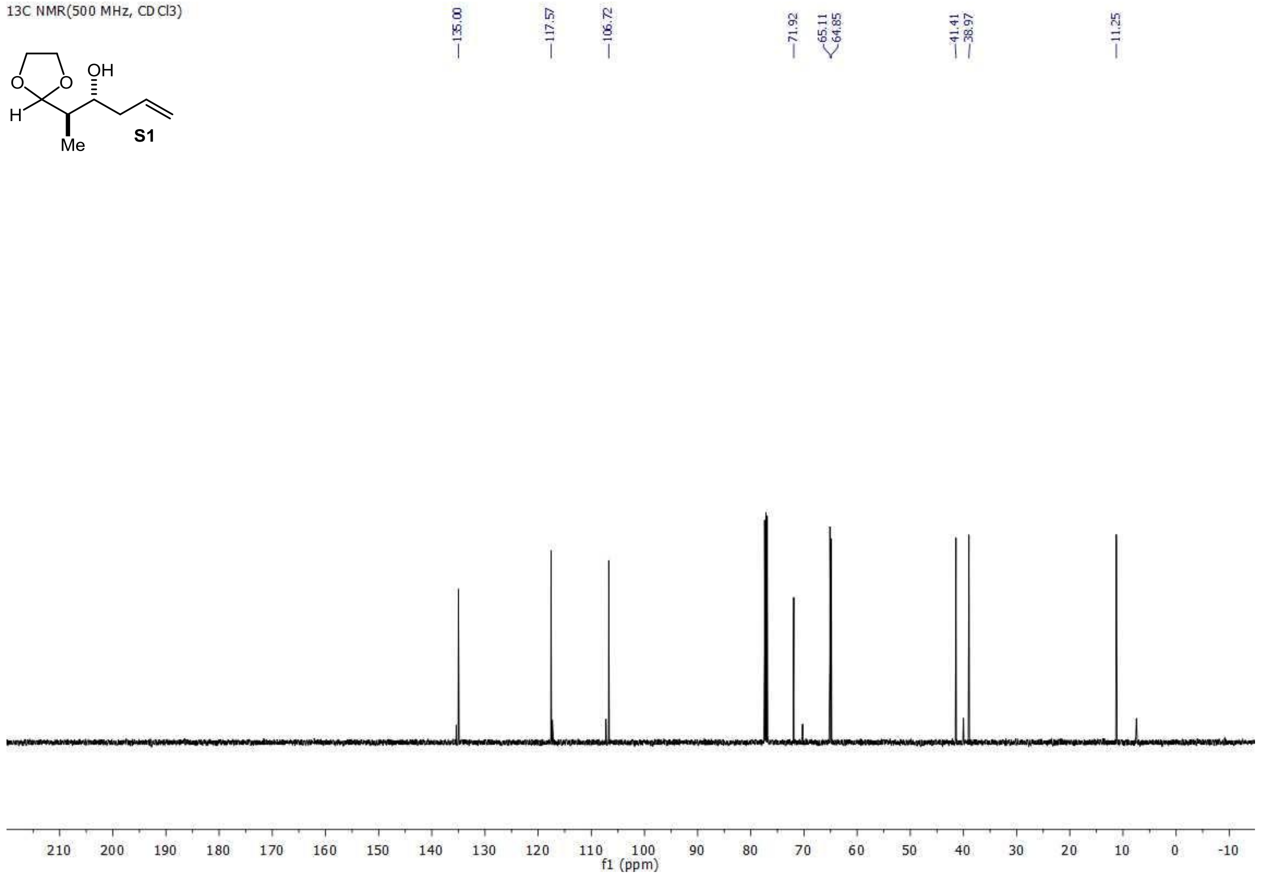

Supplementary Figure 12. ^{1}H NMR and ^{13}C NMR of **S1**.

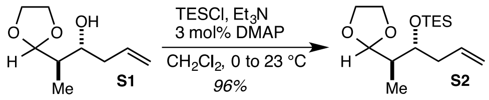

Supplementary Figure 13. Synthesis of **S2**.

To a cooled (0 °C) solution of **S1** (1.50 g, 7.69 mmol) in CH_2Cl_2 (28 mL) was added Et_3N (1.39 mL, 9.99 mmol) followed by the slow addition of TESCl (1.29 mL, 7.69 mmol) and DMAP (28.2 mg, 0.231 mmol). The resulting mixture was stirred at room temperature for 2 h. MeOH (0.3 mL) was added to quench unreacted TESCl and the resulting solution was concentrated. The residue was resuspended in hexanes and the Et_3N•HCl salts were removed by filtration. The filtrate was concentrated and purified by silica gel column chromatography eluting with 0:100 → 10:90 EtOAc:Hexanes to give product **S2** as a pale yellow oil (2.10 g, 7.38 mmol, 96% yield). This material was carried forward as an 8:1 mixture of diastereomers.

OR $[\alpha]_D^{23}$ -22.8° (*c* 1.0, $CHCl_3$)

IR (ATR) 3075, 2952, 2911, 2877, 1641, 1460, 1413, 1377, 1237, 1166, 1067 cm^{-1}

^{1}H NMR (500 MHz, $CDCl_3$) δ 5.90 (dddd, *J* = 16.9, 10.2, 7.6, 6.4 Hz, 1H), 5.13 – 5.01 (m, 2H), 4.89 (d, *J* = 4.4 Hz, 1H), 4.05 – 3.93 (m, 2H), 3.93 – 3.79 (m, 3H), 2.37 – 2.17 (m, 2H), 1.99 – 1.89 (m, 1H), 0.98 (t, *J* = 7.9 Hz, 9H), 0.91 (d, *J* = 7.0 Hz, 3H), 0.68 – 0.57 (m, 6H)

^{13}C NMR (125 MHz, CDCl3) δ 135.5, 116.9, 105.1, 72.9, 65.0, 65.0, 42.4, 38.5, 8.8, 7.1, 5.2

HRMS (ASAP+) calculated for $C_{15}H_{31}O_3Si$ $[M+H]^+$: 287.2042; found 287.2037

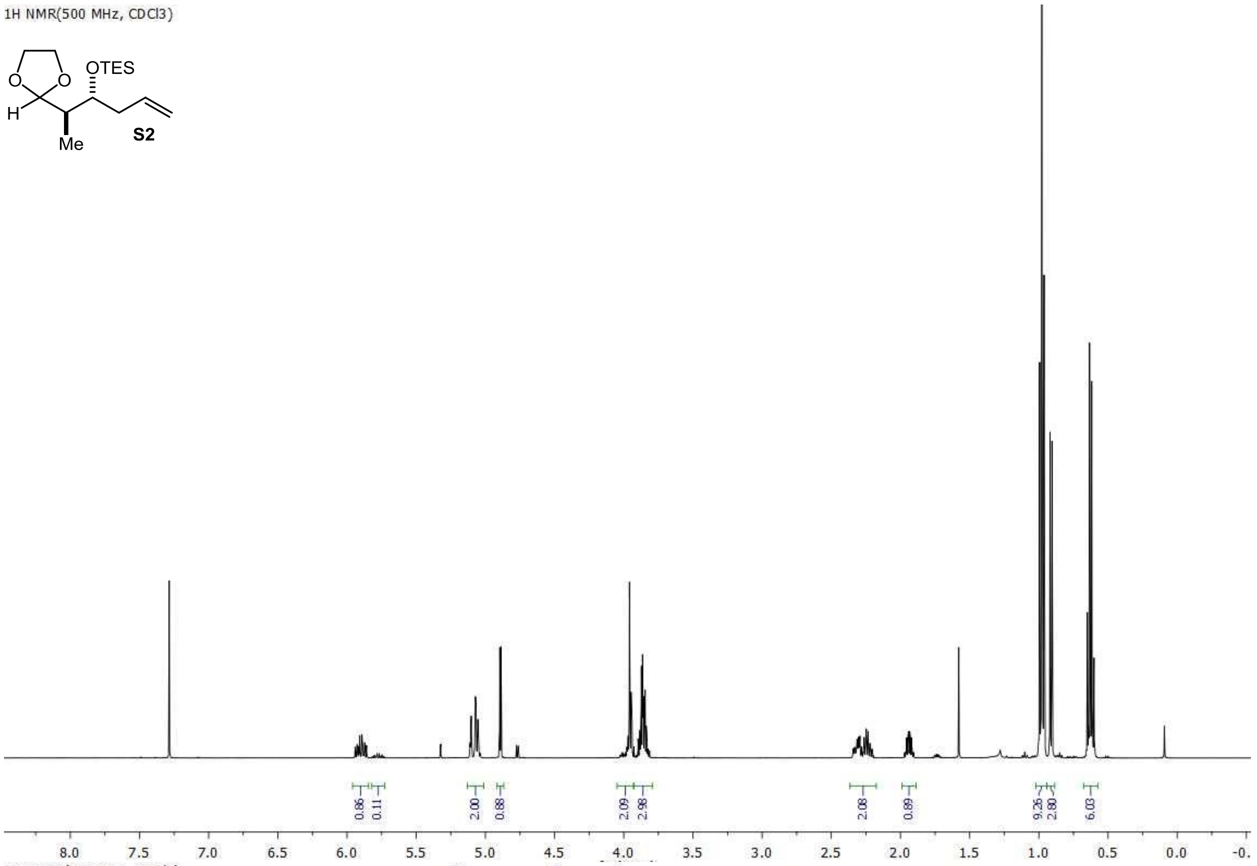

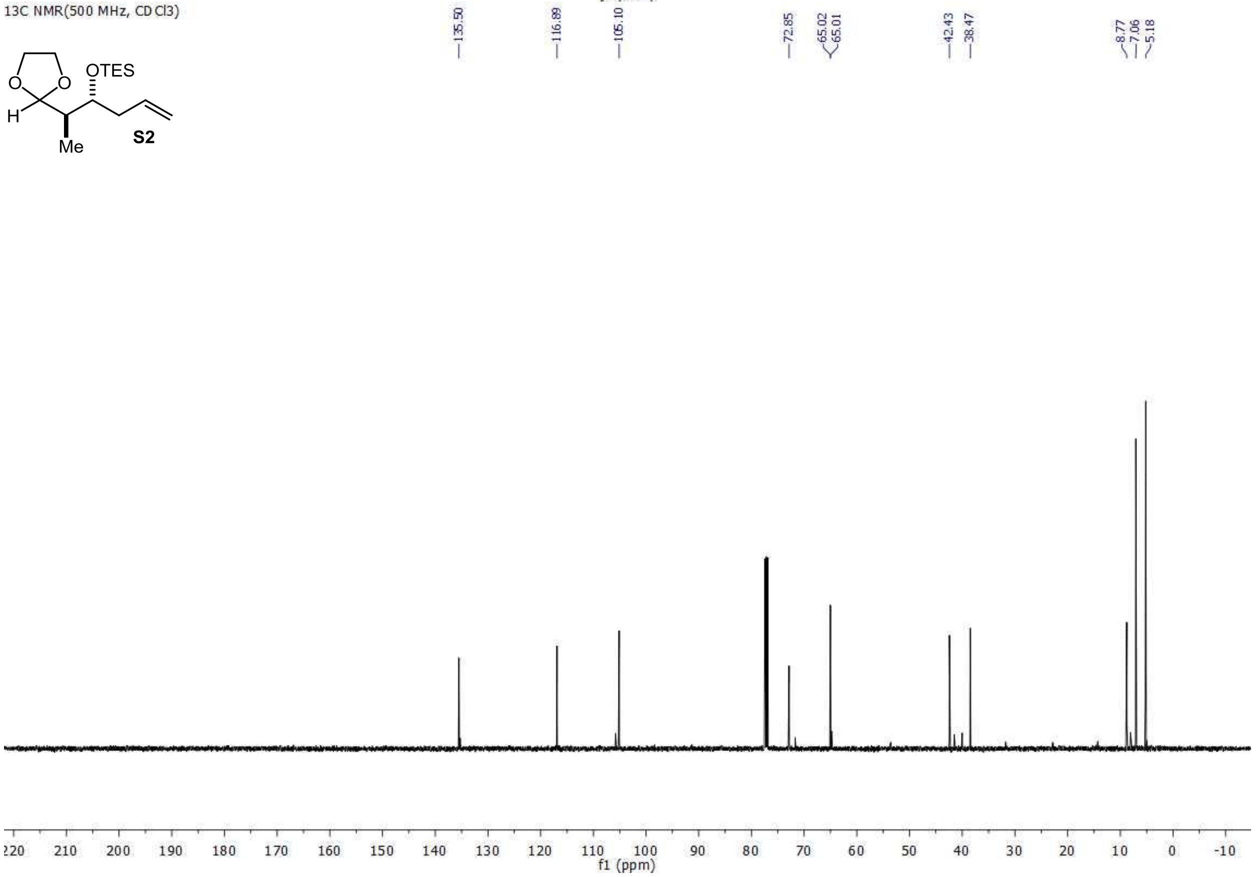

Supplementary Figure 14. ^{1}H NMR and ^{13}C NMR of **S2**.

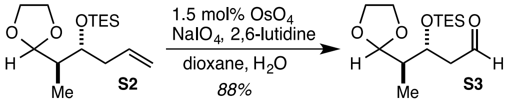

Supplementary Figure 15. Synthesis of **S3**.

To a solution of **S2** (2.0 g, 6.48 mmol) in dioxane:H_2O (60 mL:20 mL) were sequentially added 2,6-lutidine (1.05 mL, 9.08 mmol), OsO_4 (26 mg, 0.10 mmol), and $NaIO_4$ (3.43 g, 16.1 mmol). The reaction mixture was stirred at room temperature for 4 h. The reaction mixture was cooled to 0 °C and carefully quenched by the slow addition of saturated aqueous $Na_2S_2O_3$ (70 mL). The layers were separated and the aqueous layer was extracted with Et_2O (3 x 200 mL). The combined organic layers were dried over $MgSO_4$, filtered and concentrated. The residue was purified by silica gel column chromatography eluting with 0:100 → 15:85 EtOAc:Hexanes to give pure **S3** as a colorless oil (1.74 g, 6.14 mmol, 88% yield). This material was carried forward as an 8:1 mixture of diastereomers.

OR $[\alpha]_D^{22}$ +6.2º (*c* 1.0, $CHCl_3$)

IR (thin film, cm^{-1}) 2954, 2877, 1726, 1460, 1410, 1239, 1080, 1006, 816, 733

^{1}H NMR (500 MHz, $CDCl_3$ δ 9.82 (d, *J* = 2.6 Hz, 1H), 4.75 (d, *J* = 4.7 Hz, 1H), 4.46 (dt, *J* = 6.9, 4.7 Hz, 1H), 4.00 – 3.78 (m, 4H), 2.68 – 2.51 (m, 2H), 2.02 (dtd, *J* = 11.7, 7.0, 3.4 Hz, 1H), 1.02 – 0.87 (m, 13H), 0.62 (q, *J* = 7.9 Hz, 7H)

^{13}C NMR (126 MHz, $CDCl_3$) δ 202.5, 105.0, 68.3, 65.0, 65.0, 47.7, 43.3, 8.7, 6.9, 5.1

HRMS (ASAP+): calculated for $C_{14}H_{29}O_4Si$ $[M+H]^+$: 289.1835; found 289.1838

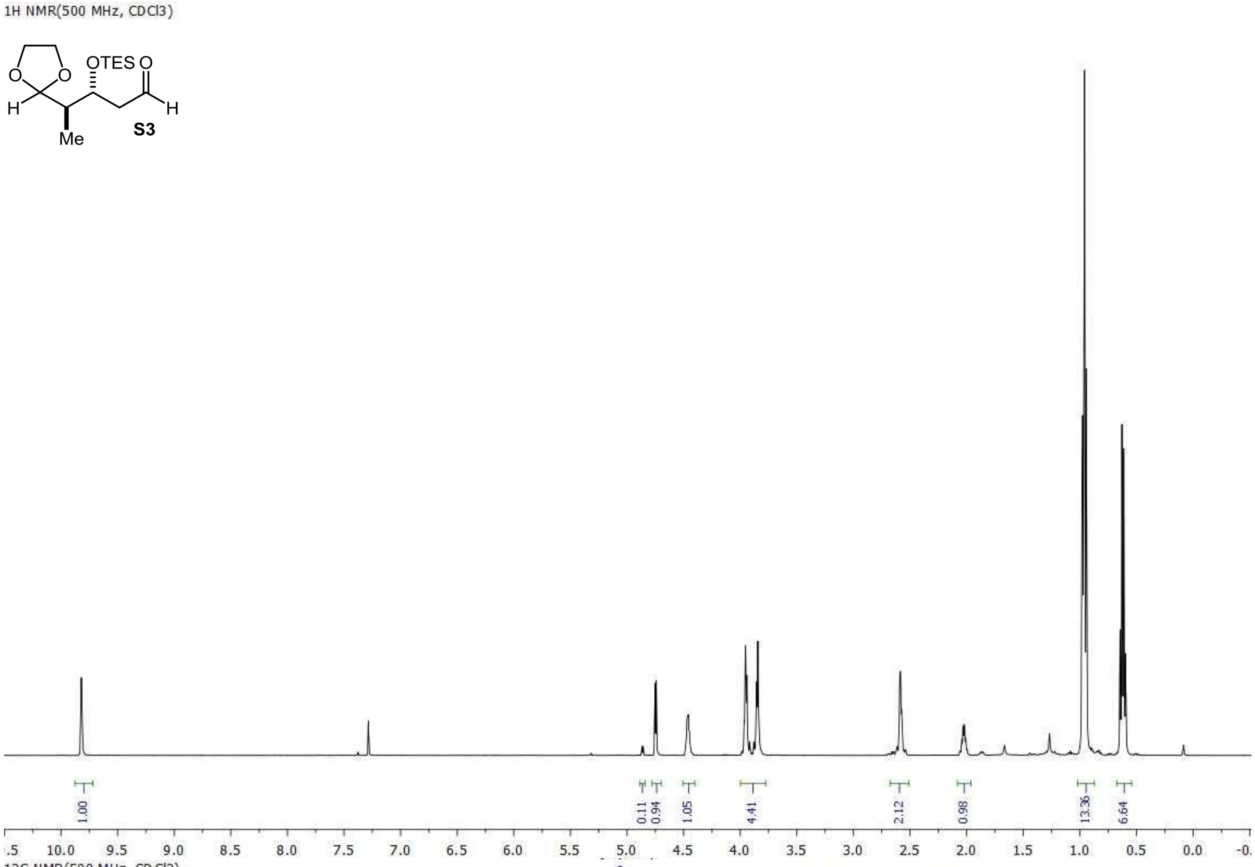

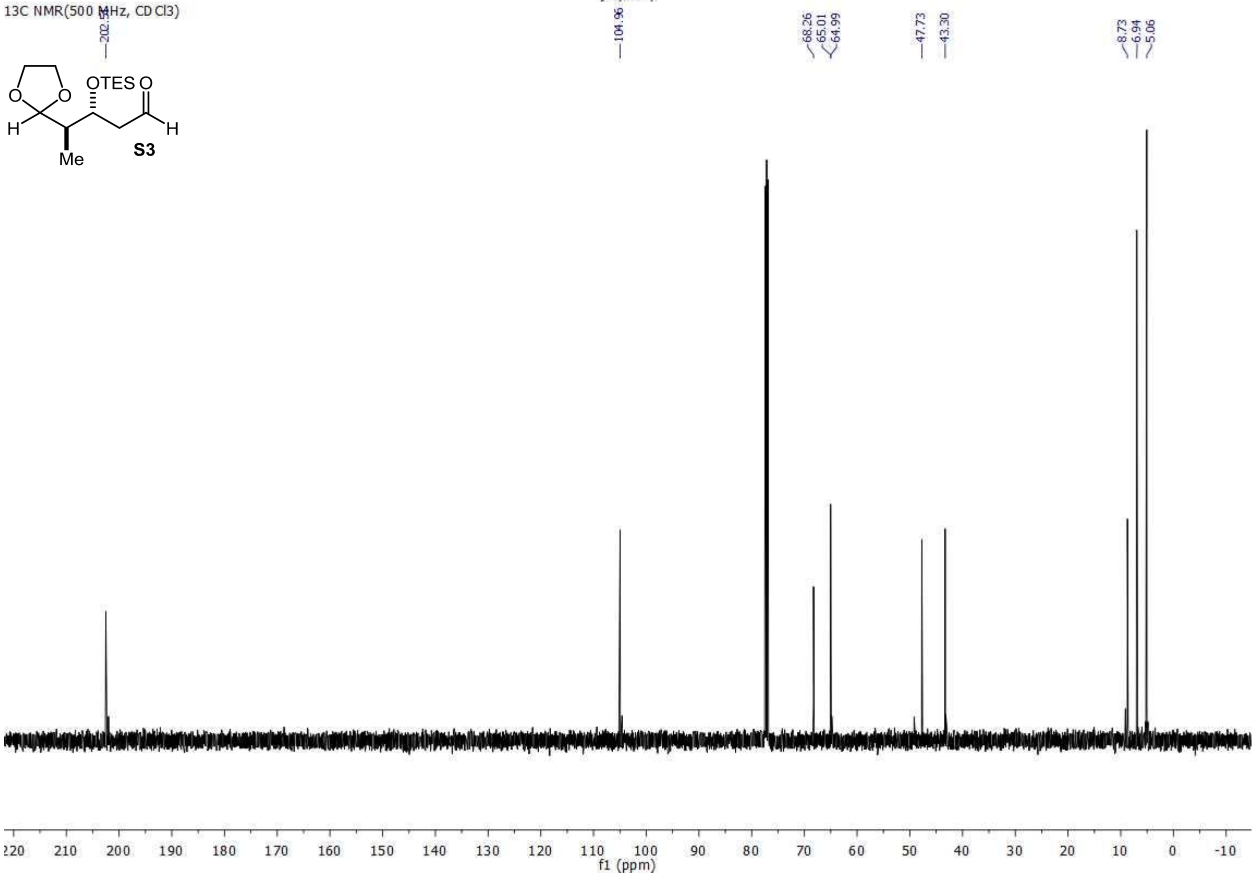

Supplementary Figure 16. ^{1}H NMR and ^{13}C NMR of **S3**.

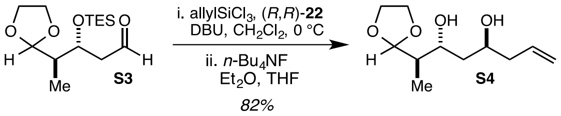

Supplementary Figure 17. Synthesis of **S4**.

Diol **S4** was prepared using a modified literature procdure.[5] To a cooled (0 °C) solution of (*R,R*)-**22** (1.80 g, 6.20 mmol) in CH_2Cl_2 (25 mL) was added DBU (2.47 mL, 18.6 mmol). The allyltrichlorosilane (1.08 mL, 7.44 mmol) was then added slowly. The ice/water bath was removed and after 1 h the mixture was recooled to 0 °C. Aldehyde **S3** (1.70 g, 5.89 mmol) was added and the resulting solution is stirred at 0 °C for 3 h. The mixture was concentrated and the residue was resuspended in Et_2O (100 mL). The mixture was stirred vigorously for 20 min to ensure complete precipitation of the DBU•HCl salts. The mixture was then filtered through a frit, and the filtrate was treated with TBAF (24.8 mL, 24.8 mmol, 1 M in THF). The mixture was filtered through a plug of silica gel, rinsing with EtOAc. The filtrate was concentrated and the residue was purified by chromatography on silica gel eluting with 0:100 → 20:80 EtOAc:Hexanes to provide pure **S4** as a pale yellow oil (1.05 g, 4.83 mmol, 82% yield). This material was carried forward as an 8:1 mixture of diastereomers.

OR $[\alpha]_D^{22}$ +9.6° (*c* 1.0, $CHCl_3$)

IR (thin film, cm^{-1}) 3389, 2896, 1641, 1405, 1160, 1104, 1049, 996, 923, 601

^{1}H NMR (500 MHz, $CDCl_3$) δ 5.85 (ddt, *J* = 17.2, 10.1, 7.2 Hz, 1H), 5.21 – 5.05 (m, 2H), 4.87 (d, *J* = 4.8 Hz, 1H), 4.01 (dq, *J* = 8.3, 4.8, 3.9 Hz, 4H), 3.95 – 3.83 (m, 2H), 2.29 (qt, *J* = 13.7, 6.9 Hz, 2H), 1.99 – 1.83 (m, 1H), 1.75 – 1.61 (m, 2H), 0.93 (d, *J* = 7.0 Hz, 3H)

^{13}C NMR (126 MHz, $CDCl_3$) δ 135.1, 117.7, 107.4, 70.7, 68.1, 65.2, 64.8, 42.2, 41.6, 38.9, 11.8

HRMS repeated attempts to observe the molecular ion using ESI, APCI, and ASAP all failed.

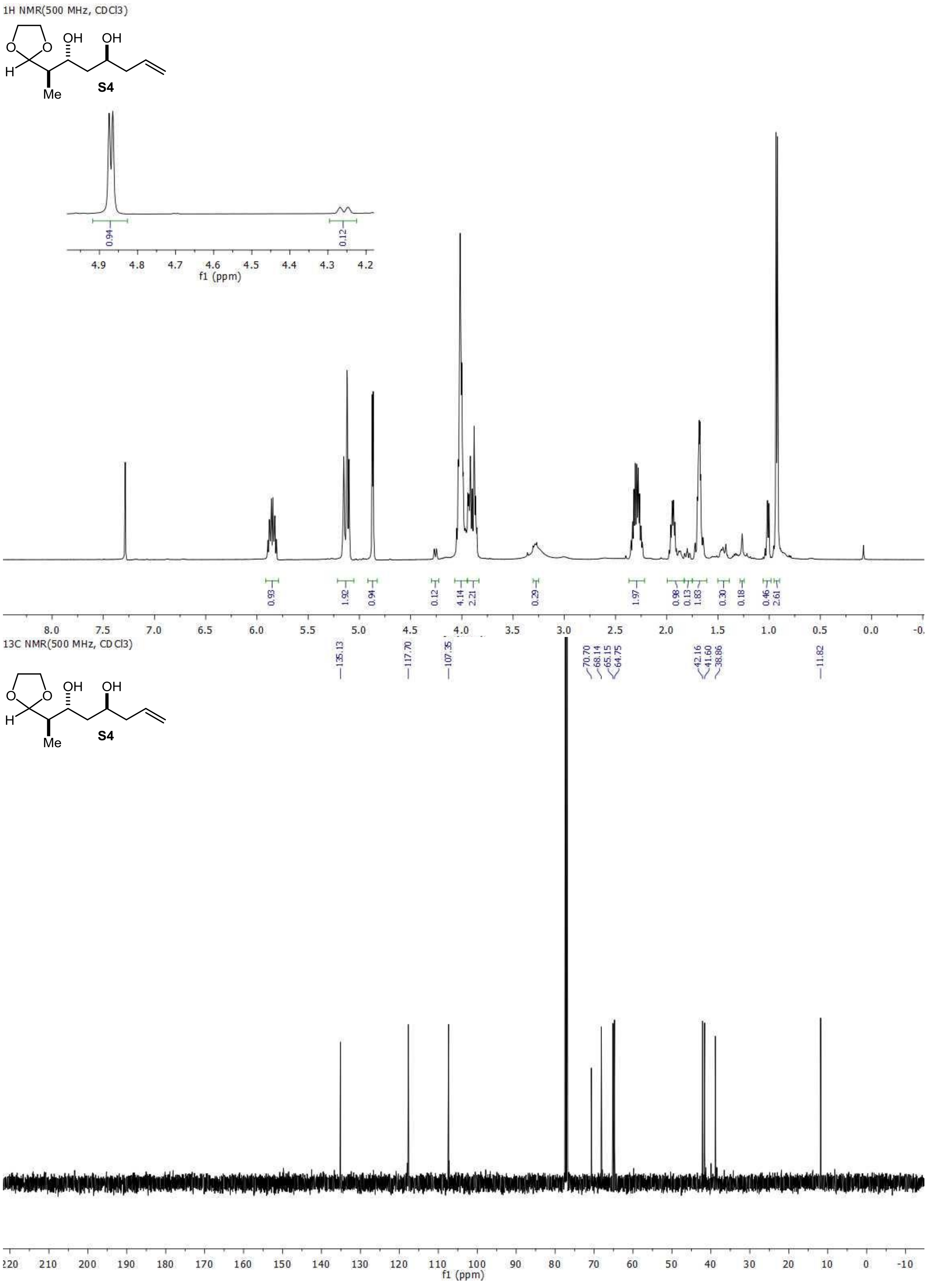

Supplementary Figure 18. ^{1}H NMR and ^{13}C NMR of **S4**.

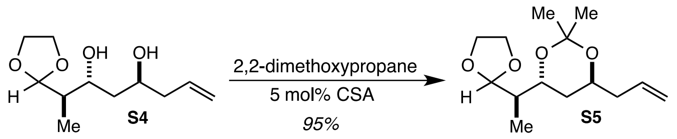

Supplementary Figure 19. Synthesis of **S5**.

To a solution of **S4** (1.00 g, 4.34 mmol) in dimethoxypropane (30 mL) was added camphor sulfonic acid (CSA) (50 mg, 0.21 mmol). The mixture was stirred at room temperature for 1 h. Saturated aqueous $NaHCO_3$ (10 mL) was added. The layers were separated and the aqueous layer was extracted with EtOAc (3 x 10 mL). The combined organic layers were washed with H_2O (1 x 15 mL), dried over Na_2SO_4, filtered, and concentrated. Purification of the residue by silica gel column chromatography eluting with 0:100 → 15:85 EtOAc:Hexanes gave pure **S5** as a clear oil (1.05 g, 4.12 mmol, 95% yield). Separation of the minor diastereomer from the first step was possible at this stage, and **S5** was isolated and taken forward as a single diastereomer (≥20:1 dr).

OR $[\alpha]_D^{22}$ +9.56º (*c* 1.0, $CHCl_3$)

IR (thin film, cm^{-1}) 2859.39, 1612.08, 1512.49, 1460.63, 1378.20, 1300.69, 1247.24, 1199.51, 1174.41, 1096.76, 1035.17, 915.21, 872.23, 817.36, 522.38

^{1}H NMR (500 MHz, $CDCl_3$) δ 5.80 (ddt, *J* = 17.1, 10.3, 6.8 Hz, 1H), 5.06 (dd, *J* = 23.2, 13.6 Hz, 2H), 4.99 (d, *J* = 2.9 Hz, 1H), 3.99 – 3.79 (m, 6H), 2.30 (dt, *J* = 13.9, 6.8 Hz, 1H), 2.17 (ddt, *J* = 20.4, 14.1, 6.8 Hz, 1H), 1.91 (dpd, *J* = 14.0, 7.0, 3.0 Hz, 1H), 1.69 (ddd, *J* = 12.7, 9.8, 6.1 Hz, 1H), 1.62 – 1.46 (m, 1H), 1.44 – 1.12 (m, 8H), 0.85 (t, *J* = 6.9 Hz, 3H)

^{13}C NMR (126 MHz, $CDCl_3$) δ 134.6, 116.8, 104.0, 100.4, 67.4, 66.3, 65.1, 65.1, 41.5, 40.2, 35.9, 24.7, 24.5, 7.7

HRMS (ASAP+) calculated for $C_{14}H_{25}O_4$ $[M+H]^+$: 257.1753; found 257.1756

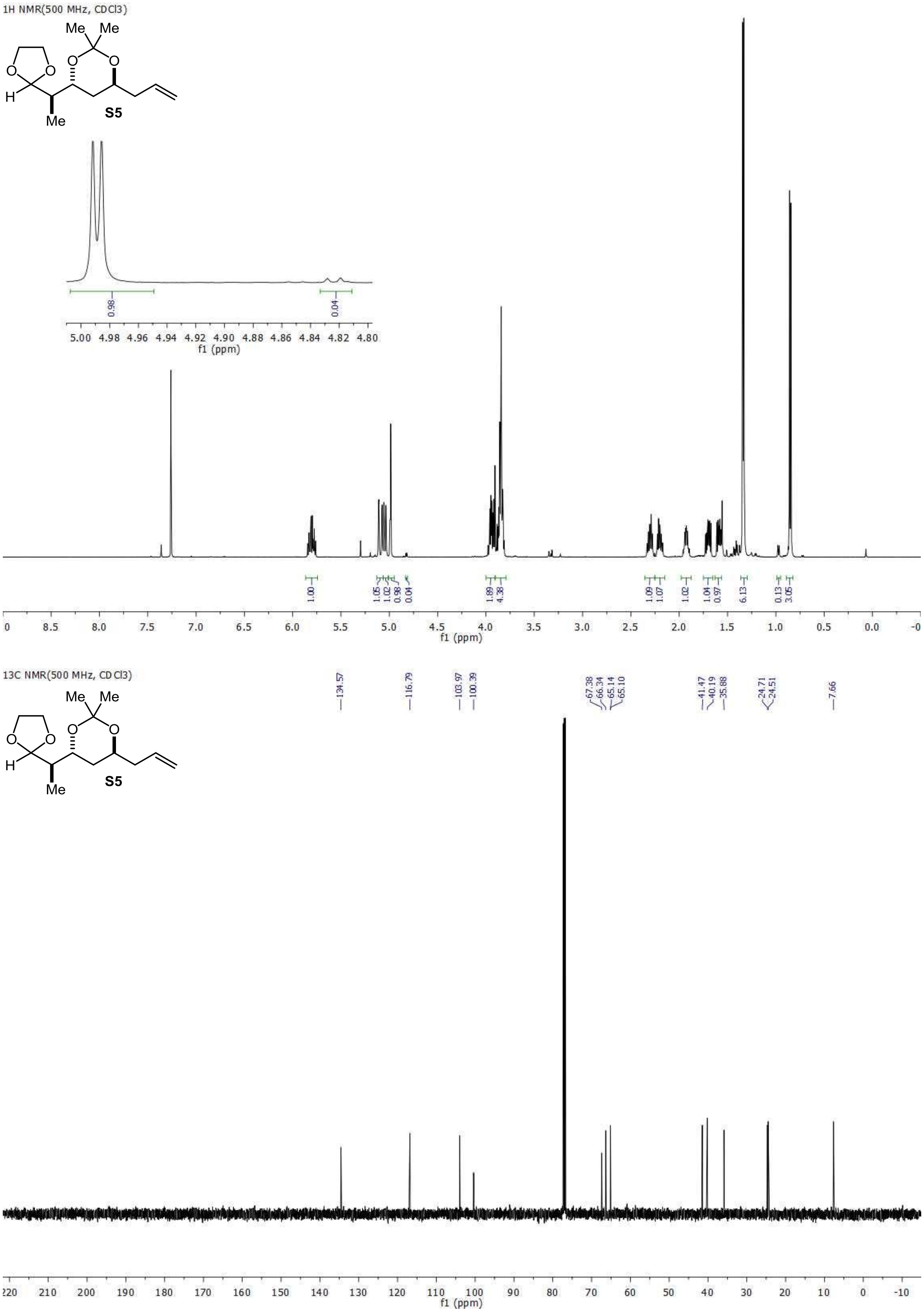

Supplementary Figure 20. ^{1}H NMR and ^{13}C NMR of **S5**.

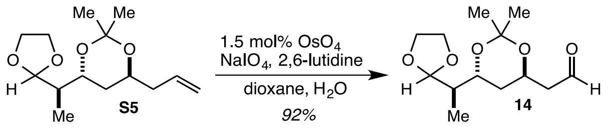

Supplementary Figure 21. Synthesis of **14**.

To a solution of **S5** (190 mg, 0.367 mmol) in dioxane:H_2O (6.0 mL:2.0 mL) were sequentially added 2,6-lutidine (0.20 mL, 0.734 mmol), OsO_4 (2.8 mg, 0.006 mmol), and $NaIO_4$ (361 mg, 0.844 mmol). The mixture was stirred at room temperature for 4 h. The reaction mixture was cooled to 0 °C and carefully quenched by the slow addition of saturated aqueous $Na_2S_2O_3$ (3.5 mL). The layers were separated and the aqueous layer was extracted with Et_2O (3 x 10 mL). The combined organic layers were dried over $MgSO_4$, filtered and concentrated. The residue was purified by silica gel column chromatography eluting with 0:100 → 30:70 EtOAc:Hexanes to give pure **14** as a colorless oil (180 mg, 0.337 mmol, 92% yield).

OR $[\alpha]_D^{22}$ +13.2º (*c* 1.0, $CHCl_3$)

IR (thin film, cm^{-1}) 2984, 2886, 1725, 1460, 1381, 1223, 1172, 1053

^{1}H NMR (500 MHz, $CDCl_3$) δ 9.74 (d, *J* = 2.3 Hz, 1H), 4.97 (d, *J* = 3.1 Hz, 1H), 4.33 (tt, *J* = 9.7, 5.4 Hz, 1H), 3.99 – 3.79 (m, 6H), 2.61 (ddd, *J* = 16.7, 8.7, 2.6 Hz, 1H), 2.49 (dd, *J* = 16.6, 4.3 Hz, 1H), 1.94 (hd, *J* = 9.6, 8.4, 4.3 Hz, 1H), 1.88 – 1.73 (m, 1H), 1.60 (ddd, *J* = 12.7, 9.5, 6.1 Hz, 1H), 1.33 (d, *J* = 10.2 Hz, 7H), 0.85 (d, *J* = 6.9 Hz, 3H)

^{13}C NMR (126 MHz, $CDCl_3$) δ 200.9, 103.9, 100.7, 67.2, 65.1, 65.1, 62.3, 49.1, 41.3, 35.8, 24.6, 24.2, 7.7

HRMS (ASAP+) calculated for $C_{13}H_{23}O_5$ $[M+H]^+$: 259.1545; found 259.1545

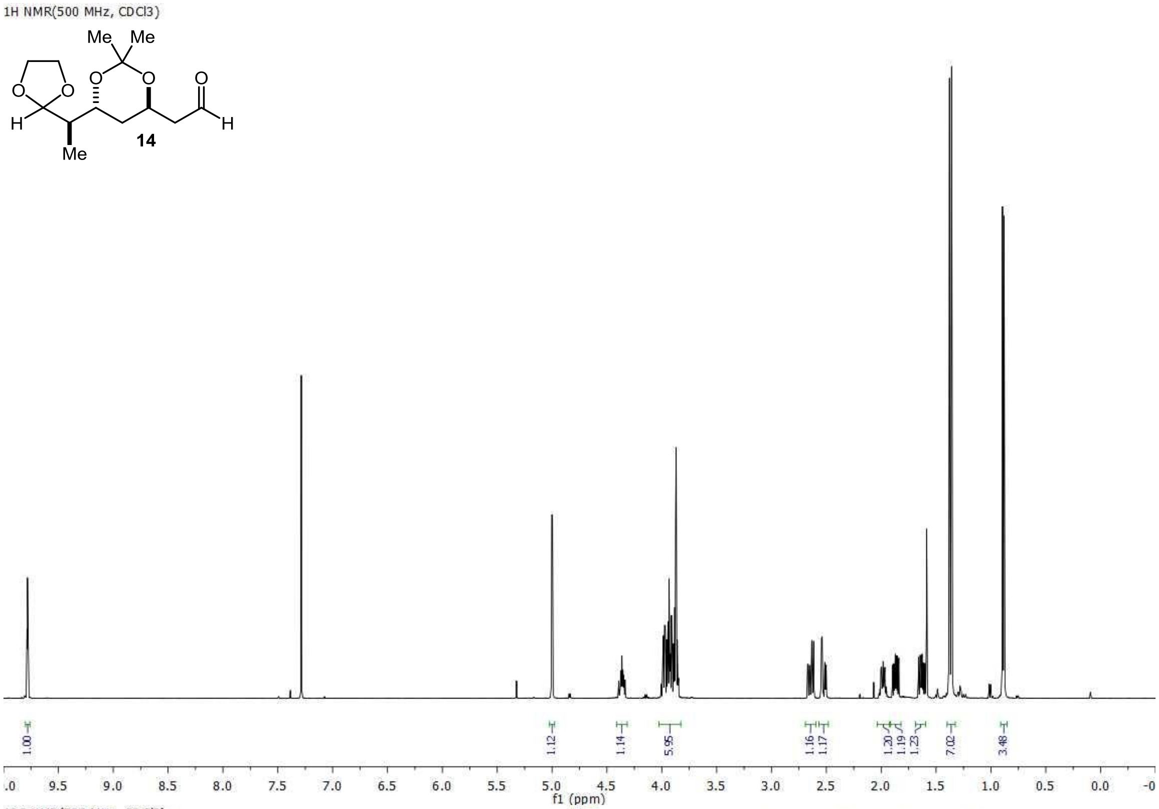

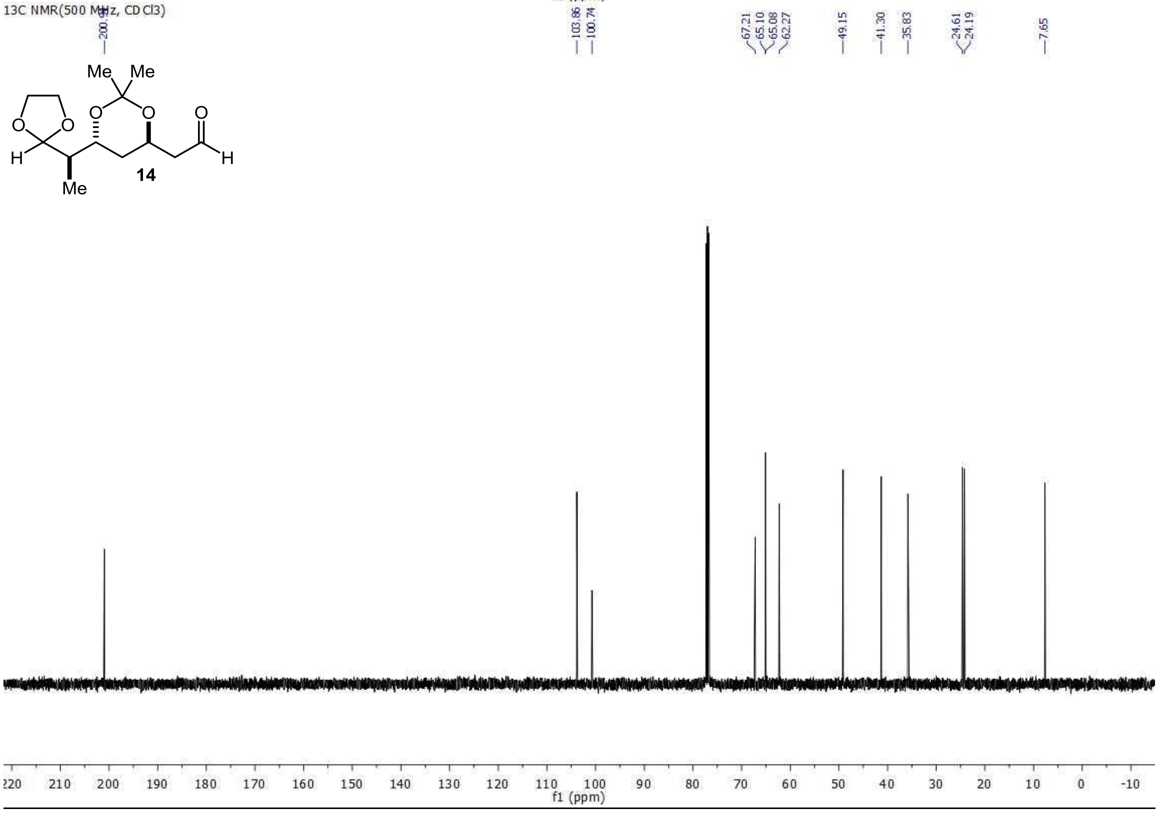

Supplementary Figure 22. ^{1}H NMR and ^{13}C NMR of **14**.

Synthesis of silyl enol ether **15**

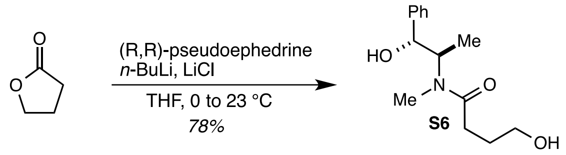

Supplementary Figure 23. Synthesis of **S6**.

Amide **S6** was prepared using a modified literature procedure.[2] To a cooled (0 °C) mixture of (*R*,*R*)-pseudoephedrine (33.0 g, 200 mmol) and LiCl (25.4 g, 600 mmol) in THF (800 mL) was added *n*-BuLi (40.0 mL, 100 mmol, 2.5 M in hexanes). The resulting mixture was stirred at 0 ºC for 30 min. γ-Butyrolactone (30.5 mL, 400 mmol) was added slowly and the solution was then warmed to room temperature over 4 h. Aqueous NaOH (1 M, 300 mL) was added and the resulting mixture was stirred for 1 h. The bulk of the THF was removed *in vacuo* and the resulting mixture was extracted with 1:9 MeOH:CH_2Cl_2 (7 x 100 mL). The combined organic layers were washed with water (1 x 200 mL), dried over Na_2SO_4, filtered, and concentrated. The residue was purified by silica gel column chromatography eluting with 6:94 MeOH:CH_2Cl_2 to give pure **S6** as a pale yellow oil (39.2 g, 156 mmol, 78% yield). The product is a 3:1 mixture of rotamers about the amide bond, (*) is used to indicate the minor rotamer peaks.

OR $[\alpha]_D^{19}$ -93.7º (*c* 1.0, $CHCl_3$)

IR (thin film, cm^{-1}) 3350, 2968, 2924, 2869, 1612, 1453, 1121, 1053, 1033, 762, 703

^{1}H NMR (500 MHz, $CDCl_3$) δ 7.40 – 7.19 (m, 5H), 4.55 (m, 1H), 4.13* (s, 1H), 4.05* (m, 1H), 3.77 – 3.59 (m, 2H), 3.56 (s, 1H)*, 3.13 (s, 1H), 2.91* (s, 3H), 2.85 (s, 3H), 2.73* (ddd, *J* = 15.9, 8.2, 5.7 Hz, 1H), 2.56 – 2.32 (m, 2H), 1.99 – 1.78* (m, 2H), 1.90 – 1.81* (m, 2H), 1.02 (d, *J* = 6.3 Hz, 3H), 0.95* (d, *J* = 6.8 Hz, 3H)

^{13}C NMR (101 MHz, $CDCl_3$) δ 175.6, 174.8*, 142.3, 141.5*, 128.8*, 128.5, 128.4*, 127.9, 127.1*, 126.7, 76.4, 75.5*, 62.4, 62.4, 58.7, 57.7*, 32.2*, 31.6, 30.7*, 27.9*, 27.6, 27.1, 15.6*, 14.5

HRMS (FAB+): calculated for $C_{14}H_{22}NO_3$ $[M+H]^+$ 252.1600; found 252.1595

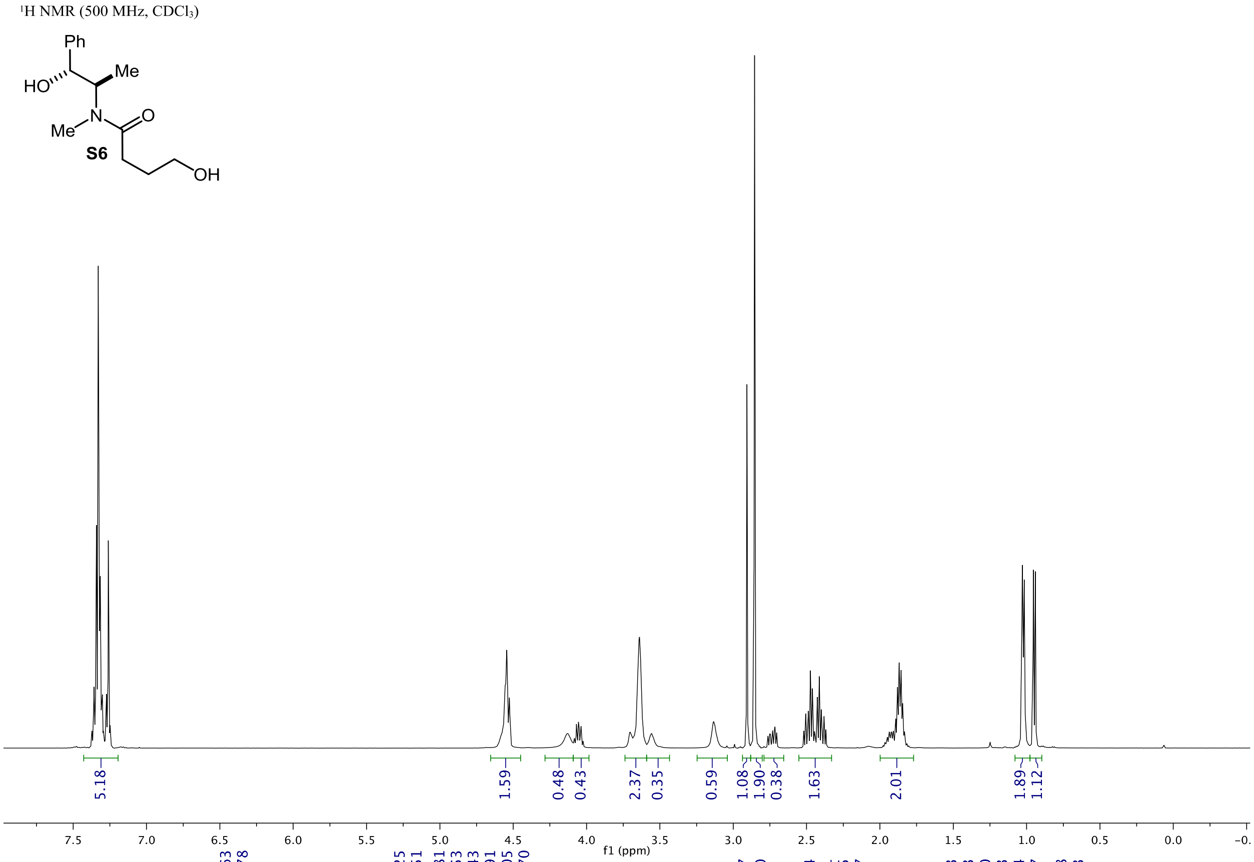

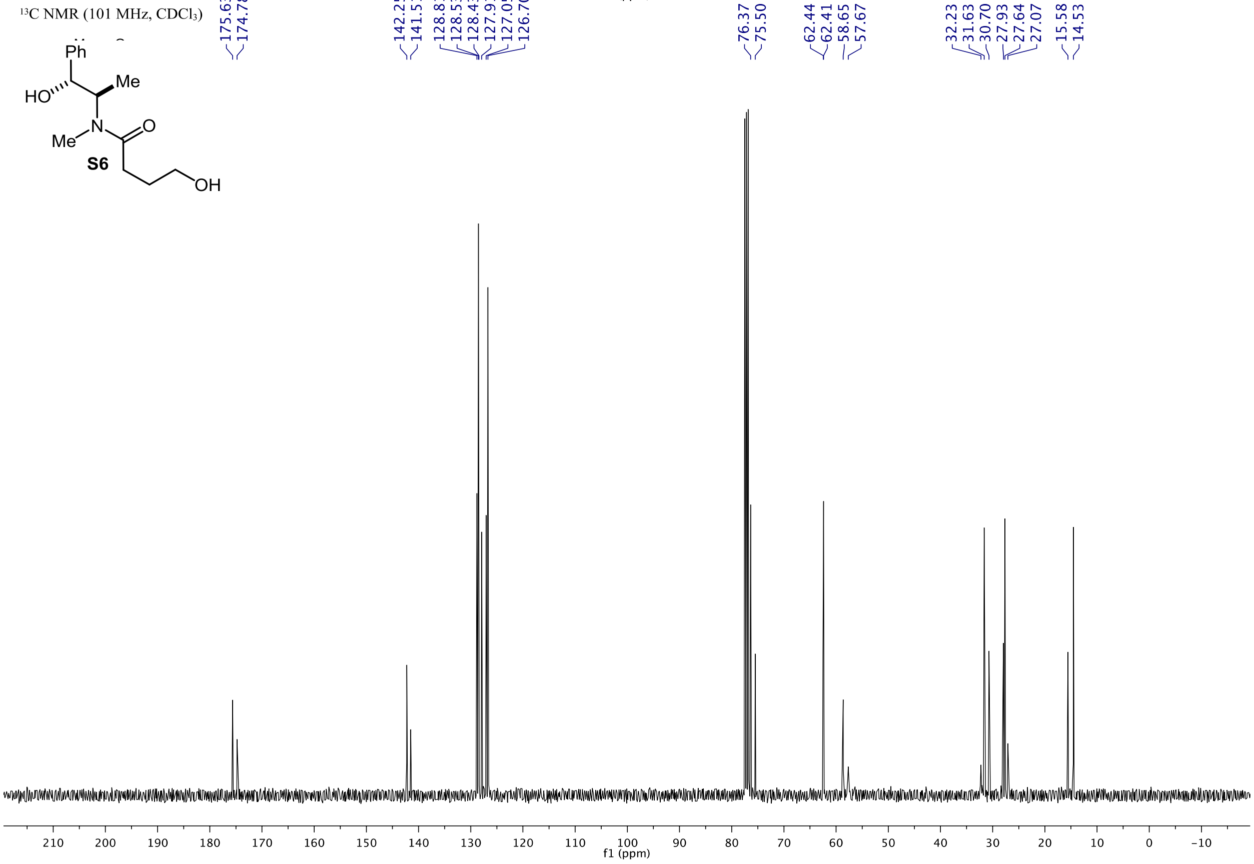

Supplementary Figure 24. ^{1}H NMR and ^{13}C NMR of **S6**.

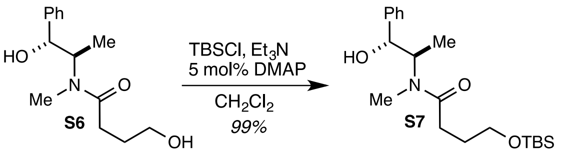

Supplementary Figure 25. Synthesis of **S7**.

To a solution of **S6** (39.2 g, 156 mmol) in CH_2Cl_2 (1.5 L) was added Et_3N (26.1 mL, 187 mmol). A solution of TBSCl (24.7 g, 164 mmol) in CH_2Cl_2 (100 mL) was added via addition funnel. DMAP (0.95 g, 7.8 mmol) was added and the resulting solution was stirred for 4 h. The reaction mixture was quenched with water (200 mL) and extracted with EtOAc (3 x 200 mL). The combined organic layers were dried over Na_2SO_4, filtered, and concentrated to give **S7** as a pale yellow oil (56.6 g, 155 mmol, 99% yield) that was used without further purification. Purification for a characterization sample was achieved by silica gel column chromatography eluting with 5:95 MeOH:CH_2Cl_2 to give pure **S7** as a pale yellow oil. The product is a 3:1 mixture of rotamers about the amide bond, (*) is used to indicate the minor rotamer peaks.

OR $[\alpha]_D^{21}$ -63.1° (*c* 1.0, $CHCl_3$)

IR (thin film, cm^{-1}) 3411, 2962, 1636, 1465, 1254, 1092

^{1}H NMR (500 MHz, $CDCl_3$) δ 7.39 – 7.27 (m, 5H), 4.63 – 4.51 (m, 1H), 4.51 – 4.38 (m, 1H), 4.29* (s, 1H), 4.13 – 3.97* (m, 1H), 3.70 – 3.56 (m, 2H), 2.91* (s, 3H), 2.82 (s, 3H), 2.60 – 2.42* (m, 1H), 2.42 – 2.30 (m, 1H), 1.93 – 1.72 (m, 2H), 1.10 (d, *J* = 6.9 Hz, 3H), 0.97* (d, *J* = 6.7 Hz, 3H), 0.89 (s, 9H), 0.88 (s, 9H), 0.07 – 0.01 (m, 6H)

^{13}C NMR (101 MHz, $CDCl_3$) δ 175.5, 174.2*, 142.6, 141.4*, 128.8*, 128.5, 127.8, 127.1*, 126.5, 77.4*, 76.7, 75.7*, 62.7*, 62.3, 58.7, 58.4*, 33.0*, 30.7, 30.2*, 28.7*, 28.3, 26.8*, 26.1*, 26.0, 18.4*, 15.4*, 14.6, -5.1*, -5.2

HRMS (FAB+) calculated for $C_{20}H_{36}NO_3Si$ $[M+H]^+$ 366.2464; found 366.2464

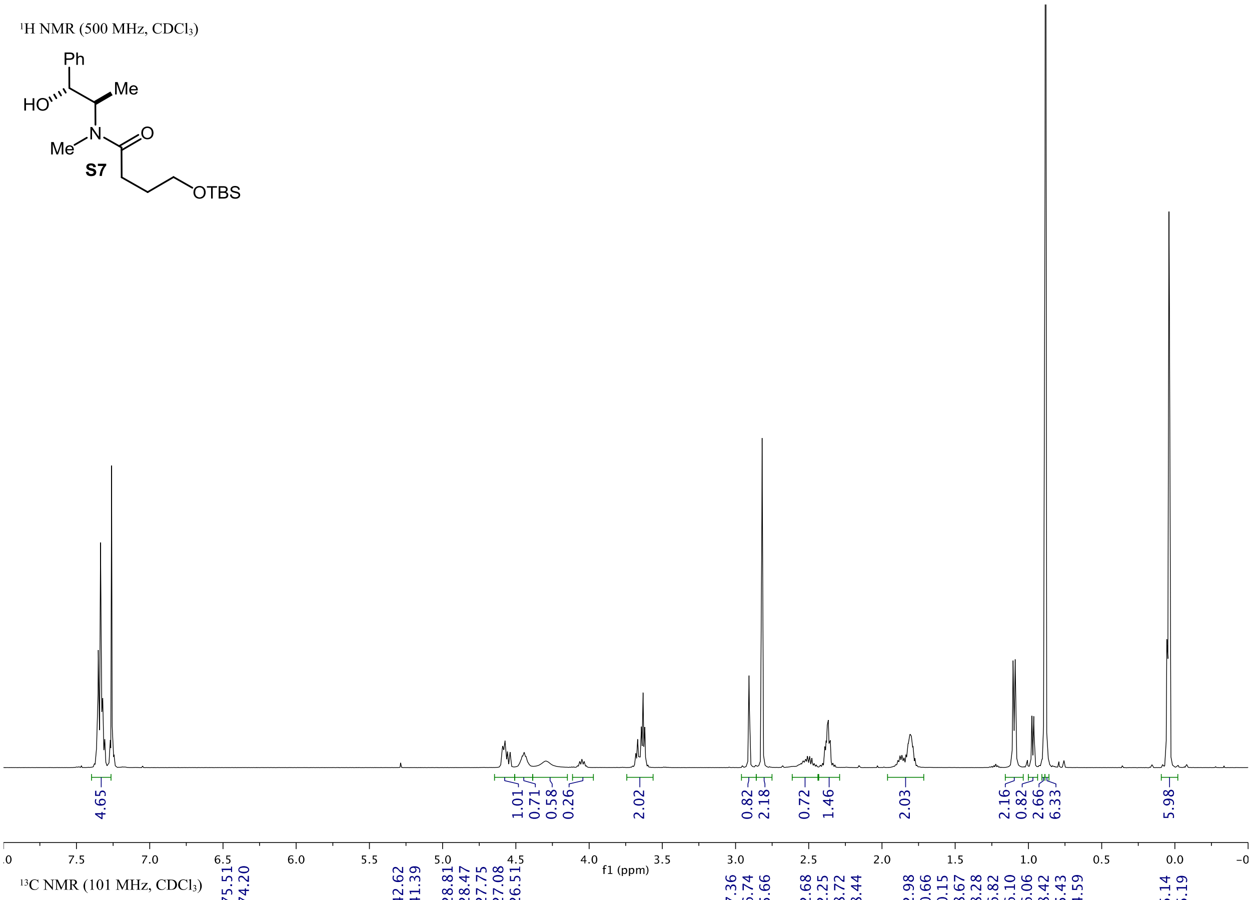

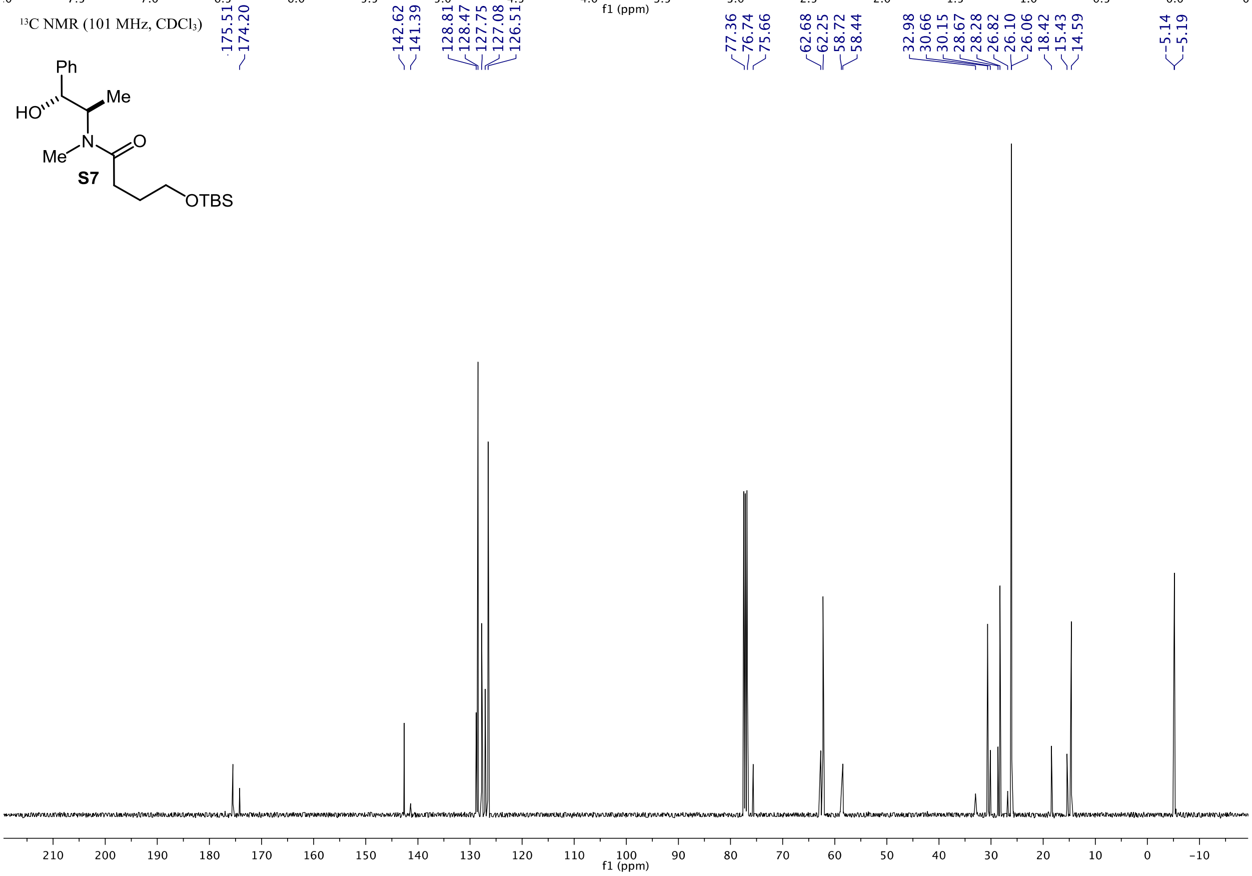

Supplementary Figure 26. ^{1}H NMR and ^{13}C NMR of **S7**.

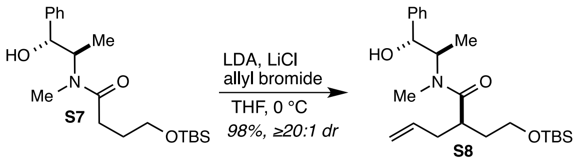

Supplementary Figure 27. Synthesis of **S8**.

Alkylation of **S7** was achieved using a modified literature procedure.[2] To a cooled (-78 °C) mixture of iPr_2NH (27.5 mL, 195 mmol) and LiCl (22.0 g, 519 mmol) in THF (100 mL) was added *n*-BuLi (73.0 mL, 182 mmol, 2.5 M in hexanes). The resulting mixture was stirred at -78 °C for 15 min and then at room temperature for 5 min, and then it was re-cooled to -78 °C. Amide **S-7** (31.6 g, 86 mmol) was added as a cooled (0 ºC) solution in THF (100 mL), rinsing with THF (20 mL). The resulting mixture was stirred for 1 h at -78 °C, then 15 min at 0 °C, and then 5 min at room temperature. The reaction mixture was re-cooled to 0 °C and allylbromide (11.2 mL, 130 mmol) was added slowly. After 1 h, the reaction was quenched by the addition of saturated aqueous NH_4Cl (50 mL) and the mixture was extracted with EtOAc (3 x 150 mL). The combined organic layers were dried over Na_2SO_4, filtered, and concentrated. The residue was purified by silica gel column chromatography eluting with 3:97 → 5:95 MeOH:CH_2Cl_2 to give pure **S-8** as a pale yellow oil (34.3 g, 85 mmol, 98% yield). The product is a 4:1 mixture of rotamers about the amide bond. (*) is used to indicate the minor rotamer peaks.

OR $[\alpha]_D^{22}$ -69.4º (*c* 1.0, $CHCl_3$)

IR (thin film, cm^{-1}) 3389, 2953, 2854, 1618, 1447, 1245, 1097, 834, 701

^{1}H NMR (400 MHz, $CDCl_3$) δ 7.41 – 7.29 (m, 4H), 7.29 – 7.21 (m, 1H), 5.91 – 5.73* (m, 1H), 5.73 – 5.55 (m, 1H), 5.18 – 4.90 (m, 2H), 4.65 – 4.51 (m, 1H), 4.43 (s, 1H), 4.33 – 4.24* (m, 1H), 3.70 – 3.58 (m, 1H), 3.57 – 3.49 (m, 1H), 3.49 – 3.42* (m, 1H), 3.18* (p, *J* = 7.0 Hz, 1H), 3.03 – 2.93 (m, 1H), 2.92* (s, 3H), 2.90* (s, 3H), 2.53 – 2.39* (m, 1H), 2.39 – 2.19 (m, 1H), 2.19 – 2.08* (m, 1H), 1.90 – 1.71 (m, 1H), 1.71 – 1.56 (m, 1H), 1.10 (d, *J* = 7.0 Hz, 3H), 1.00* (d, *J* = 6.7 Hz, 3H), 0.88 (s, 9H), 0.83* (s, 9H), 0.04 (d, *J* = 1.7 Hz, 6H), -0.01* (d, *J* = 19.9 Hz, 6H)

^{13}C NMR (101 MHz, $CDCl_3$) δ 178.0, 176.8*, 142.6, 141.2*, 136.9*, 136.0, 128.8*, 128.5, 127.7, 127.1*, 126.6, 116.8*, 116.6, 76.5, 75.7*, 60.7*, 60.5, 59.5, 58.2*, 38.3, 37.5*, 37.0*, 36.9, 36.1*, 35.7, 33.3, 27.3*, 26.1, 18.4, 15.8*, 14.7, -5.2

HRMS (FAB+) calculated for $C_{23}H_{40}NO_3Si$ $[M+H]^+$: 406.2777; found 406.2776

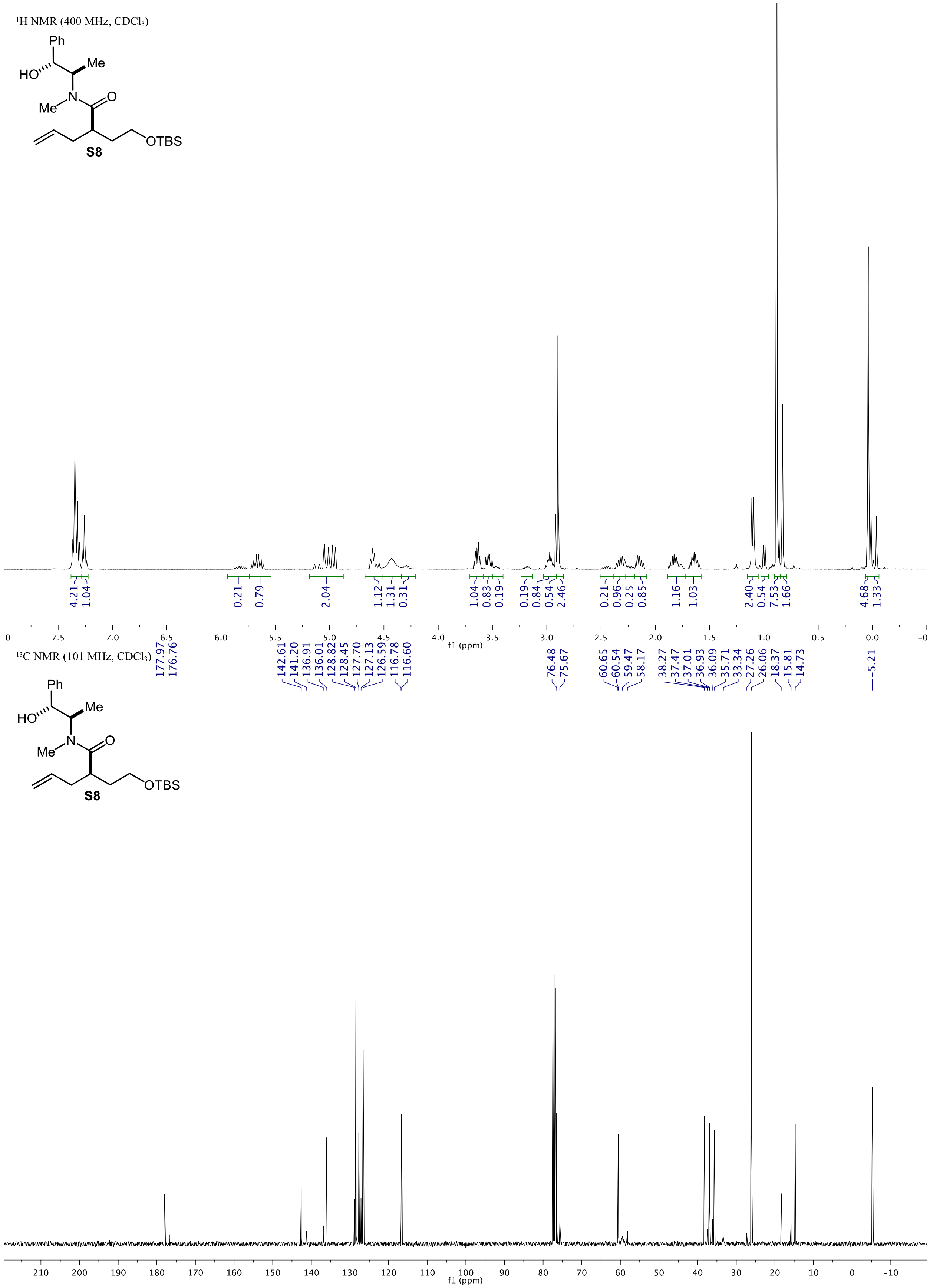

Supplementary Figure 28. ^{1}H NMR and ^{13}C NMR of **S8**.

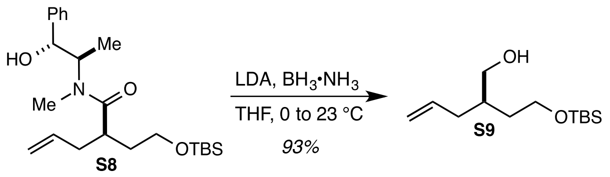

Supplementary Figure 29. Synthesis of **S9**.

To a cooled (-78 °C) solution of iPr_2NH (1.51 mL, 10.7 mmol) in THF (10 mL) was added *n*-BuLi (4.0 mL, 9.9 mmol, 2.5 M in hexanes). The resulting solution was stirred at -78 ºC for 10 min and at 0 ºC for 10 min. BH_3•NH_3 (0.31 g, 10.2 mmol) was added in 1 portion [***CAUTION!!***: gas evolution]. The resulting mixture was stirred at 0 ºC for 15 min and at room temperature for 15 min before being re-cooled to 0 °C. Amide **S8** (1.03 g, 2.5 mmol) was added as a solution in THF (6.0 mL) via addition funnel. The resulting mixture was warmed to room temperature and stirred for 5 h. The reaction mixture was re-cooled to 0 ºC, and then carefully quenched with saturated aqueous NH_4Cl (10 mL). The mixture was extracted with Et_2O (3 x 5.0 mL), and the combined organic layers were dried over $MgSO_4$, filtered, and concentrated. The residue was purified by silica gel column chromatography eluting with 8:92 EtOAc:Hexanes to give pure **S9** as a colorless oil (0.58g, 2.4 mmol, 93% yield).

OR $[\alpha]_D^{22}$ +7.1º (*c* 1.0, $CHCl_3$); Reisman has reported a synthesis of the enantiomer of **S9**[7] and reported its optical rotation as: $[\alpha]_D^{25}$ -8.4° (*c* 0.93, $CHCl_3$)

IR (thin film, cm^{-1}) 3416, 2926, 1640, 1470, 1249, 1088

^{1}H NMR (400 MHz, $CDCl_3$) δ 5.91 – 5.64 (m, 1H), 5.12 – 4.93 (m, 2H), 3.77 (ddd, *J* = 10.4, 6.2, 4.2 Hz, 1H), 3.71 – 3.54 (m, 2H), 3.47 (dd, *J* = 11.1, 6.5 Hz, 1H), 3.12 (s, 1H), 2.24 – 1.92 (m, 2H), 1.83 – 1.61 (m, 2H), 1.61 – 1.40 (m, 1H), 0.90 (s, 9H), 0.08 (s, 6H)

^{13}C NMR (101 MHz, $CDCl_3$) δ 137.1, 116.4, 66.1, 62.0, 39.6, 36.6, 35, 26, 26, 18, -5.3, -5.3

HRMS (FAB+) calculated for $C_{13}H_{29}O_2Si$ $[M+H]^+$: 245.1930; found 245.1937

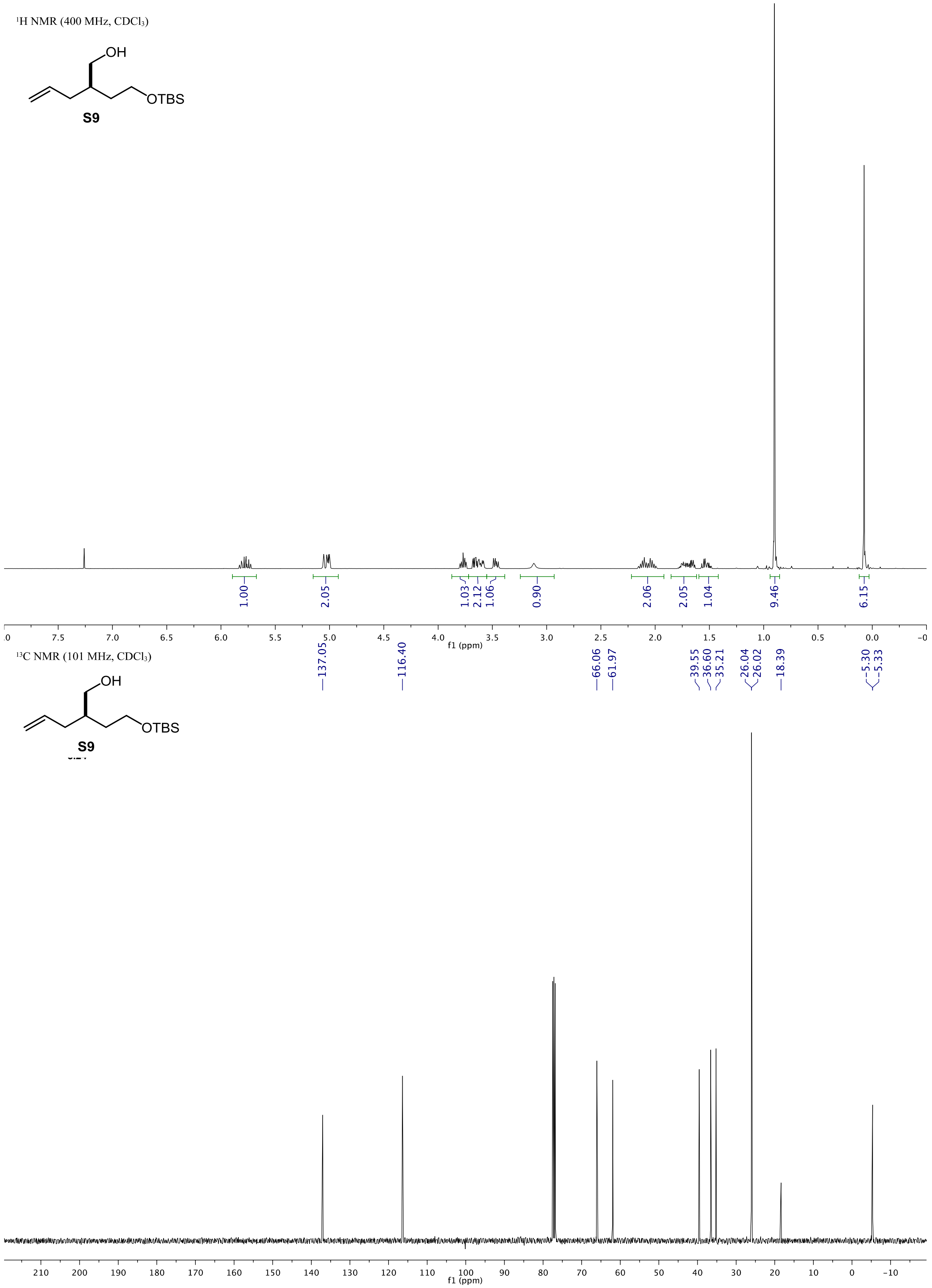

Supplementary Figure 30. ^{1}H NMR and ^{13}C NMR of **S9**.

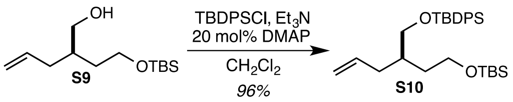

Supplementary Figure 31. Synthesis of **S10**.

To a solution of alcohol **S9** (37.2 g, 152 mmol) in CH_2Cl_2 (1.2 L) were sequentially added Et_3N (32.0 mL, 228 mmol), TBDPSCl (50.4 mL, 193 mmol), and DMAP (3.71 g, 30.4 mmol). The resulting mixture was stirred for 12 h. MeOH (8.0 mL) was added to quench unreacted TBDPSCl and the resulting mixture was concentrated. The residue was resuspended in hexanes and the resulting precipitated Et_3N•HCl salts were removed by filtration. The filtrate was washed with water (1 x 200 mL) and saturated NH_4Cl (2 x 150 mL). The aqueous layers were extracted with EtOAc (3 x 100 mL). The combined organic layers were dried over Na_2SO_4, filtered, and concentrated to give **S10** as a pale yellow oil (70.6 g, 146 mmol, 96% yield) which was used without further purification. Purification for a characterization sample was achieved by silica gel column chromatography eluting with 2:98 EtOAc:Hexanes to give pure **S10** as a pale yellow oil.

OR $[\alpha]_D^{18}$ -5.1° (*c* 1.0, $CHCl_3$)

IR (thin film, cm^{-1}) 2928, 2858, 1471, 1428, 1253, 1090, 1056, 1033, 1007, 833, 775, 702, 505

^{1}H NMR (400 MHz, $CDCl_3$) δ 7.75 – 7.59 (m, 4H), 7.51 – 7.31 (m, 6H), 5.87 – 5.65 (m, 1H), 5.12 – 4.90 (m, 2H), 3.73 – 3.47 (m, 4H), 2.35 – 2.19 (m, 1H), 2.19 – 2.05 (m, 1H), 1.78 (hept, *J* = 6.0 Hz, 1H), 1.70 – 1.47 (m, 2H), 1.08 (d, *J* = 1.0 Hz, 9H), 0.89 (d, *J* = 0.9 Hz, 9H), 0.03 (dd, *J* = 2.0, 0.9 Hz, 6H)

^{13}C NMR (101 MHz, $CDCl_3$) δ 137.3, 135.8, 134.1, 134.1, 129.7, 127.7, 116.1, 66.0, 61.4, 37.5, 35.7, 34.1, 27.1, 26.1, 19.5, 18.5, -5.1

HRMS (FAB+) calculated for $C_{29}H_{47}O_2Si_2$ $[M+H]^+$: 483.3115; found 483.3103

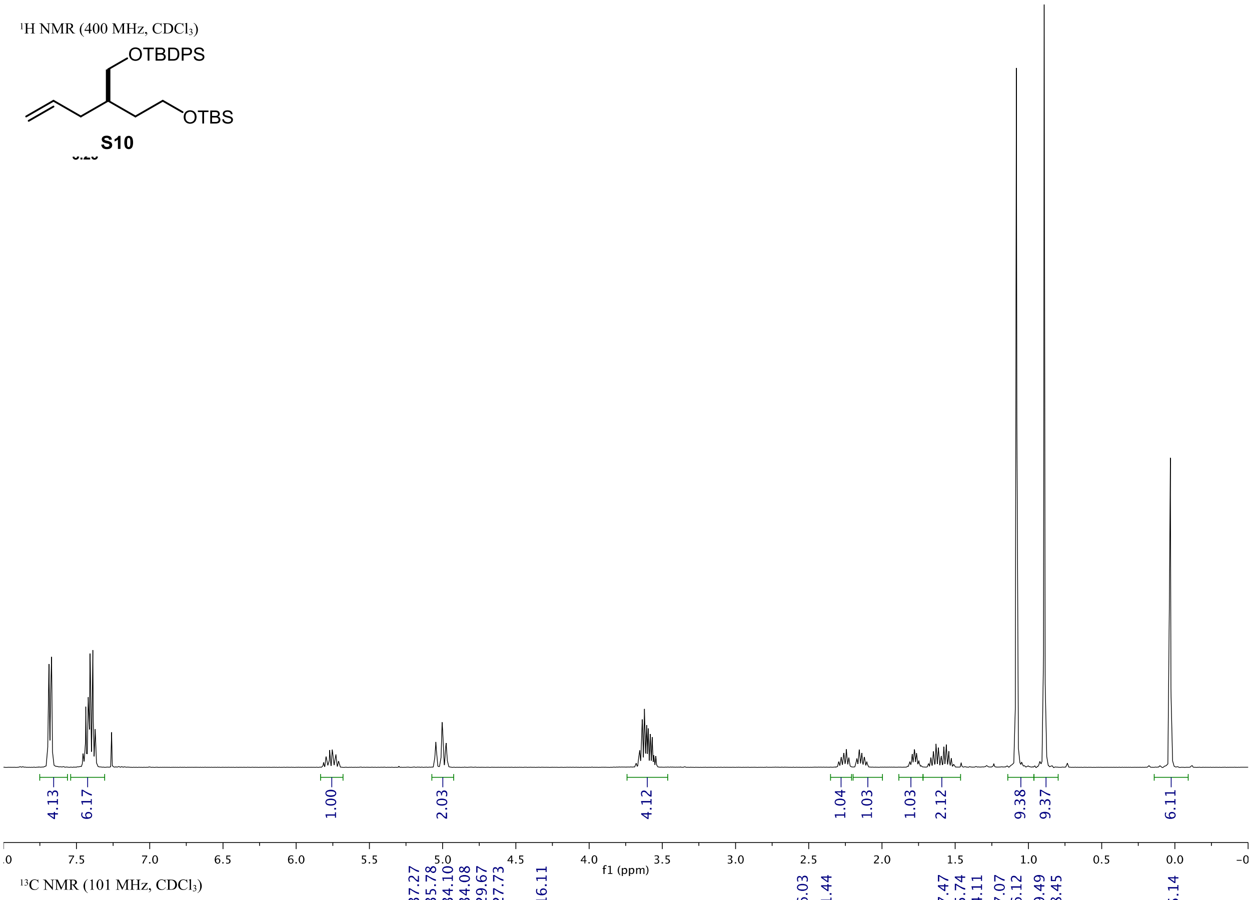

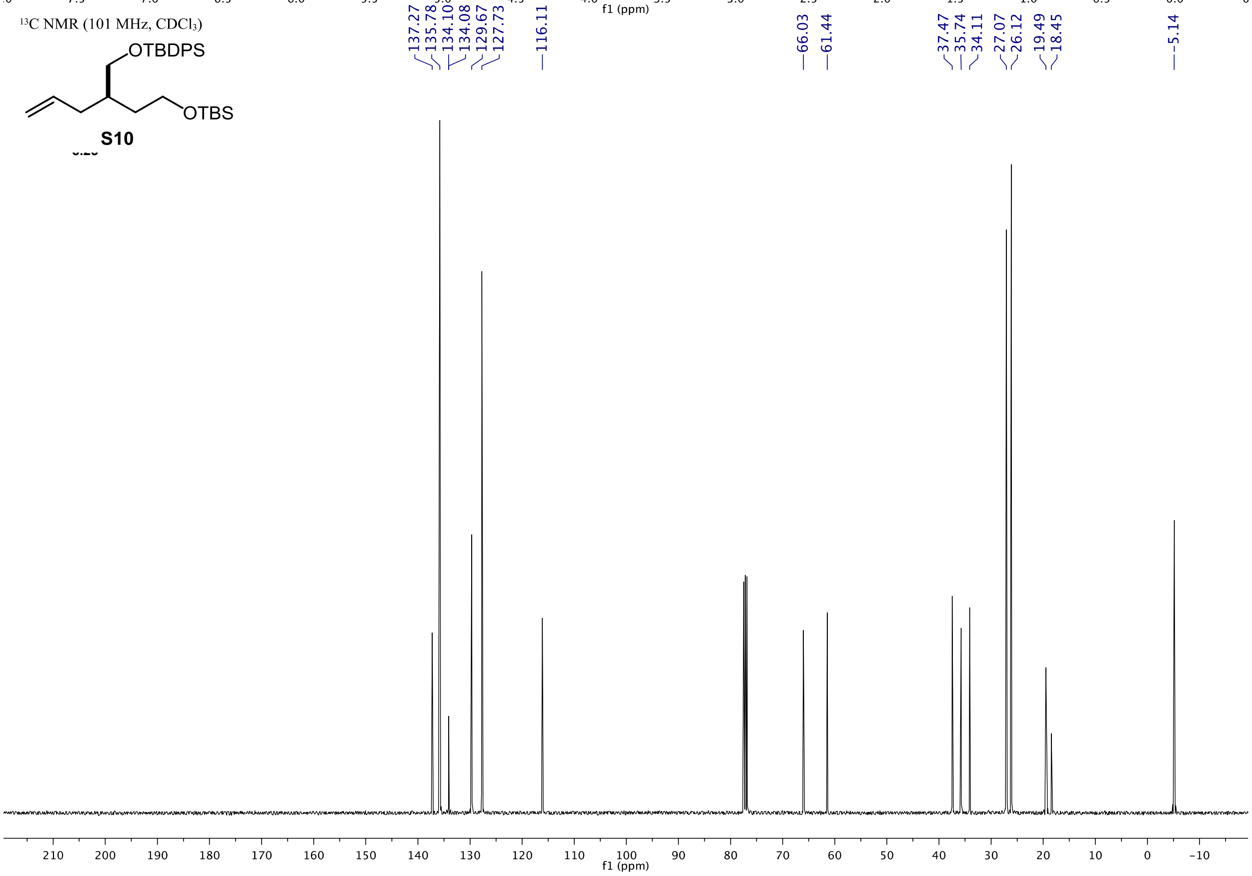

Supplementary Figure 32. ^{1}H NMR and ^{13}C NMR of **S10**.

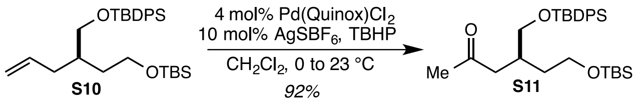

Supplementary Figure 33. Synthesis of **S11**.

Wacker oxidation of alkene **S10** was performed using Sigman's protocol.[3] To a suspension of $AgSbF_6$ (3.35 g, 9.8 mmol) in CH_2Cl_2 (200 mL) was added Pd(Quinox)Cl_2 (1.47 g, 3.9 mmol). The resulting mixture was stirred for 15 min and then diluted with CH_2Cl_2 (400 mL). 70% aqueous *tert*-butyl hydrogenperoxide (340 mL, 2.3 mol) was added and the resulting mixture was stirred for 10 min. The reaction mixture was cooled to 0 °C and alkene **S10** (47.1 g, 97.5 mmol) was added, rinsing with minimal CH_2Cl_2. The resulting mixture was warmed to room temperature and stirred for 3 h. The reaction mixture was re-cooled to 0 °C and carefully quenched by the slow addition of saturated aqueous Na_2SO_3 (800 mL). The layers were separated and the aqueous layer was extracted with hexanes (3 x 200 mL). The combined organic layers were washed with H_2O (1 x 500 mL), dried over Na_2SO_4, filtered, and concentrated. The residue was purified by silica gel column chromatography eluting with 3:97 → 8:92 EtOAc:Hexanes to give **S11** as a pale yellow oil (44.6 g, 89 mmol, 92% yield).

OR $[\alpha]_D^{22}$ -2.1° (*c* 1.0, $CHCl_3$)

IR (thin film, cm^{-1}) 2930, 2857, 1716, 1471, 1427, 1254, 1107, 835, 776, 739, 703, 614, 505

^{1}H NMR (400 MHz, $CDCl_3$) δ 7.73 – 7.59 (m, 4H), 7.49 – 7.32 (m, 6H), 3.68 – 3.44 (m, 4H), 2.64 (dd, *J* = 16.5, 6.9 Hz, 1H), 2.41 (dd, *J* = 16.5, 6.3 Hz, 1H), 2.35 – 2.22 (m, 1H), 2.10 (s, 3H), 1.72 – 1.54 (m, 1H), 1.54 – 1.39 (m, 1H), 1.05 (s, 9H), 0.86 (s, 9H), 0.00 (s, 6H)

^{13}C NMR (101 MHz, $CDCl_3$) δ 208.7, 135.7, 135.7, 133.8, 129.8, 127.8, 66.3, 61.2, 46.1, 34.4, 34.1, 30.4, 27.1, 26.1, 19.5, 18.4, -5.2

HRMS (FAB+) calculated for $C_{29}H_{45}O_3Si_2$ $[M-H]^-$: 497.2907; found 497.2917

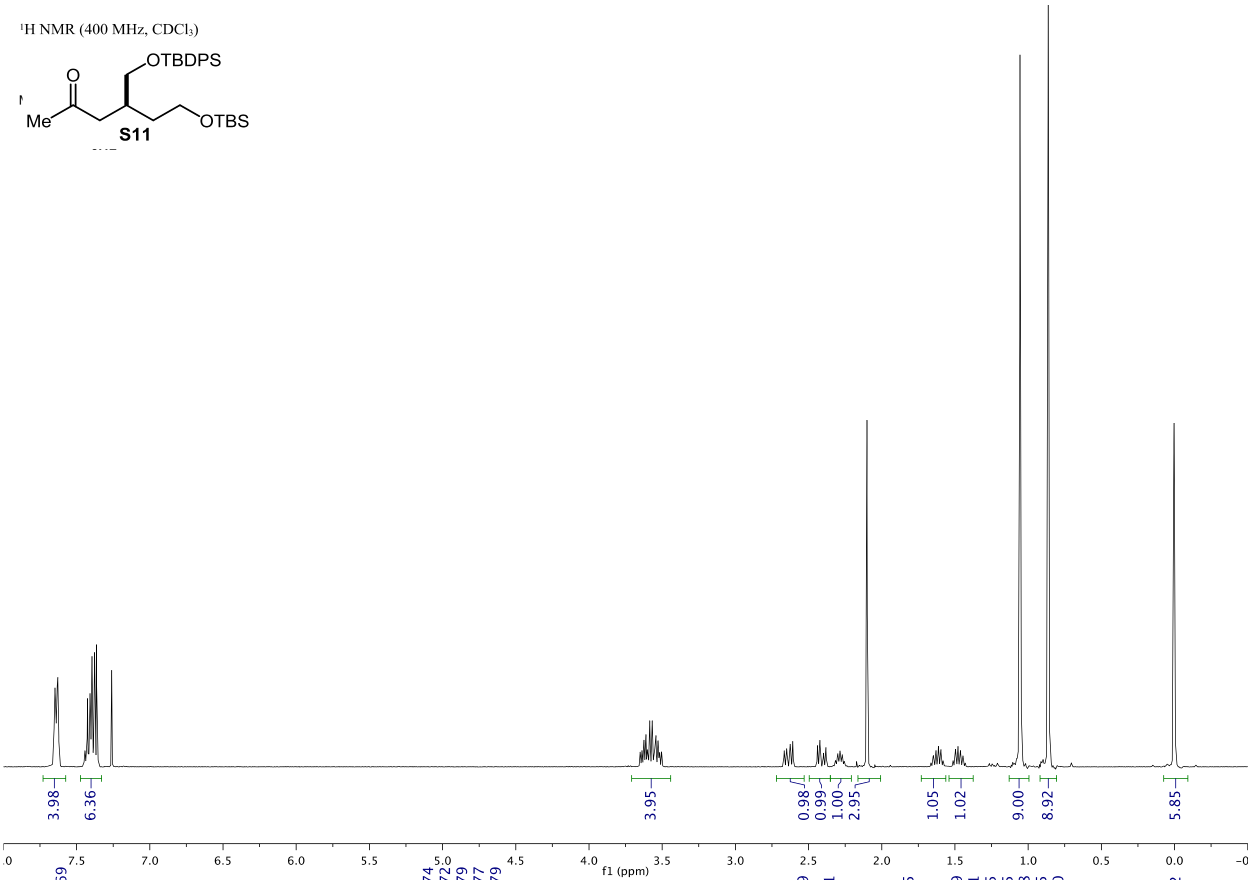

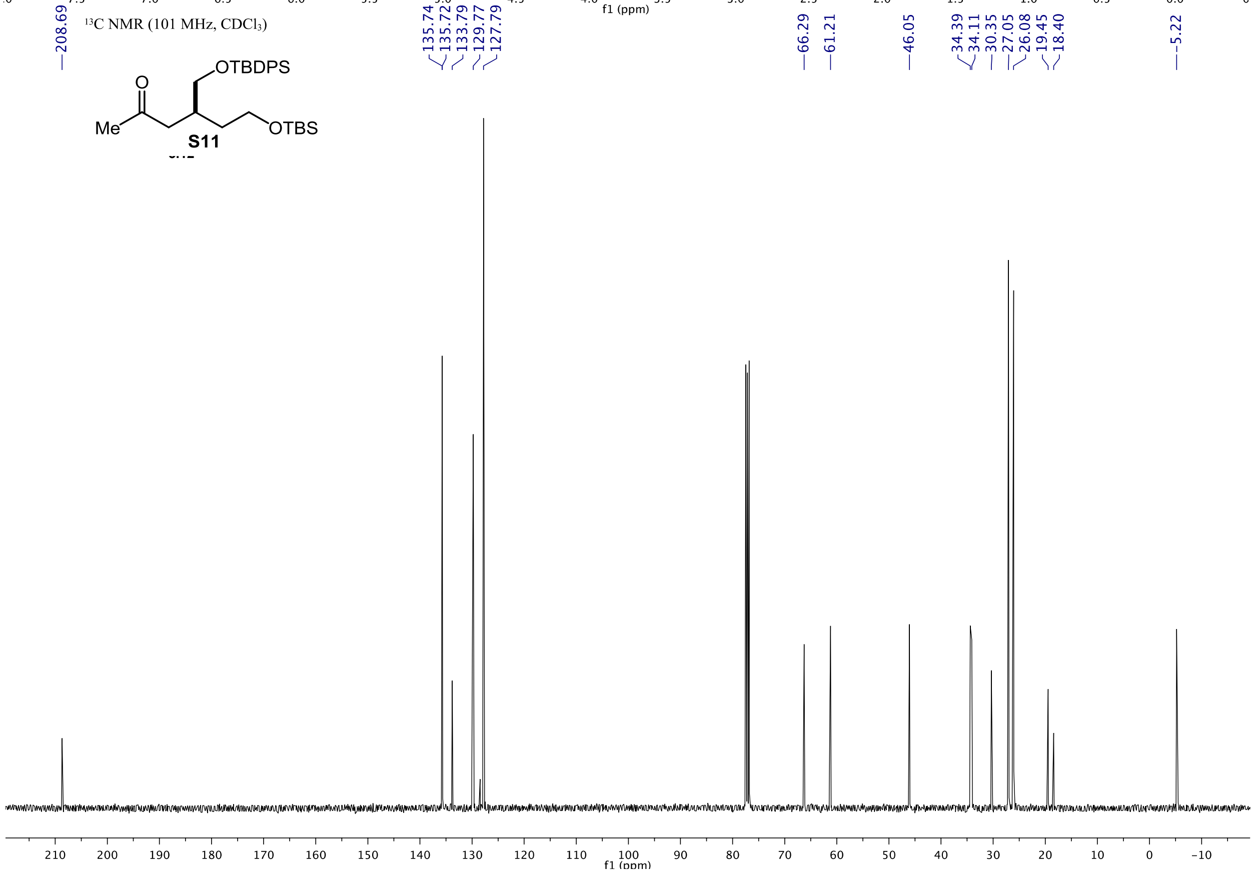

Supplementary Figure 34. ^{1}H NMR and ^{13}C NMR of **S11**.

Synthesis of aldehyde **9b** from **14** and **15**

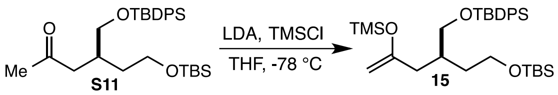

Supplementary Figure 35. Synthesis of **15**.

To a cooled (-78 °C) solution of iPr_2NH (99 µL, 0.70 mmol) in THF (3 mL) was added *n*-BuLi (240 µL, 0.60 mmol, 2.5 M in hexanes). After 5 min the solution was warmed to 0 °C, held at that temperature for 10 min, and then re-cooled to -78 °C. A solution of **S11** (250 mg, 0.50 mmol) in THF (1 mL) was added slowly, with a THF rinse (1 mL). After 20 min, TMSCl (102 µL, 0.80 mmol) was added slowly. After 1 h, the mixture was warmed to room temperature and concentrated to give a thick cloudy oil. [***CAUTION***: over concentration will result in isomerization of the enol ether product]. The residue was resuspended in pentane and the iPr_2NH•HCl salts were removed by filtration. The filtrate was concentrated to give enol ether **15** as a pale yellow oil that was used immediately in the next step without further purification.

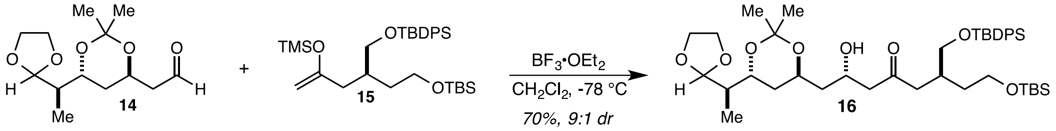

Supplementary Figure 36. Synthesis of **16**.

To a cooled (-78 °C) solution of the enol ether **15** and aldehyde **14** (90 mg, 0.35 mmol) in CH_2Cl_2 (5 mL) was added BF_3•Et_2O (51 µL, 0.39 mmol) over 20 min by syringe pump. After 1 h the reaction was quenched by the addition of saturated aqueous $NaHCO_3$ (2.5 mL). The layers were separated and the aqueous layer was extracted with CH_2Cl_2 (3 x 15 mL). The combined organic layers were dried over Na_2SO_4, filtered, and concentrated. Analysis of the residue by ^1H NMR spectroscopy revealed a 9:1 dr for the reaction. The residue was purified by silica gel column chromatography eluting with 0:100 → 40:60 EtOAc:Hexanes to give **16** as a colorless oil in 9:1 dr (170 mg, 0.25 mmol, 70% yield).

OR $[\alpha]_D^{21}$ +2.9° (*c* 1.0, $CHCl_3$)

IR (thin film, cm^{-1}) 3519, 2930, 2857, 1707, 1471, 1428, 1381, 1224, 1100, 938, 836, 777, 703, 505

^{1}H NMR (500 MHz, $CDCl_3$) δ 7.62 (dd, *J* = 7.5, 3.4 Hz, 4H), 7.39 (dt, *J* = 14.5, 7.2 Hz, 6H), 4.98 (d, *J* = 3.1 Hz, 1H), 4.22 (s, 1H), 4.11 – 4.04 (m, 1H), 4.00 – 3.89 (m, 2H), 3.89 – 3.80 (m, 3H), 3.66 – 3.61 (m, 1H), 3.61 – 3.48 (m, 3H), 3.29 (d, *J* = 3.7 Hz, 1H), 2.66 (dd, *J* = 16.8, 6.7 Hz, 1H), 2.54 – 2.45 (m, 2H), 2.40 (dd, *J* = 16.9, 6.4 Hz, 1H), 2.27 (p, *J* = 6.2 Hz, 1H), 1.94 (tp, *J* = 7.2, 3.5, 3.1 Hz, 1H), 1.71 (ddd, *J* = 12.6, 9.7, 6.1 Hz, 1H), 1.66 – 1.55 (m, 4H), 1.54 – 1.42 (m, 2H), 1.34 (d, *J* = 7.4 Hz, 6H), 1.26 (s, 1H), 1.05 (s, 9H), 0.97 (d, *J* = 6.9 Hz, 1H), 0.85 (s, 12H), -0.02 (d, *J* = 2.2 Hz, 6H)

^{13}C NMR (126 MHz, $CDCl_3$) δ 211.5, 135.7, 135.7, 133.7, 129.8, 127.8, 104.1, 100.7, 67.6, 66.0, 65.3, 65.2, 64.8, 63.9, 61.2, 49.8, 45.8, 41.9, 41.5, 36.3, 34.3, 33.7, 27.1, 26.1, 24.8, 24.6, 19.5, 18.4, 7.8, -5.2

HRMS (ESI+) calculated for $C_{42}H_{68}O_8Si_2Na$ $[M+Na]^+$: 779.4350; found 779.4344

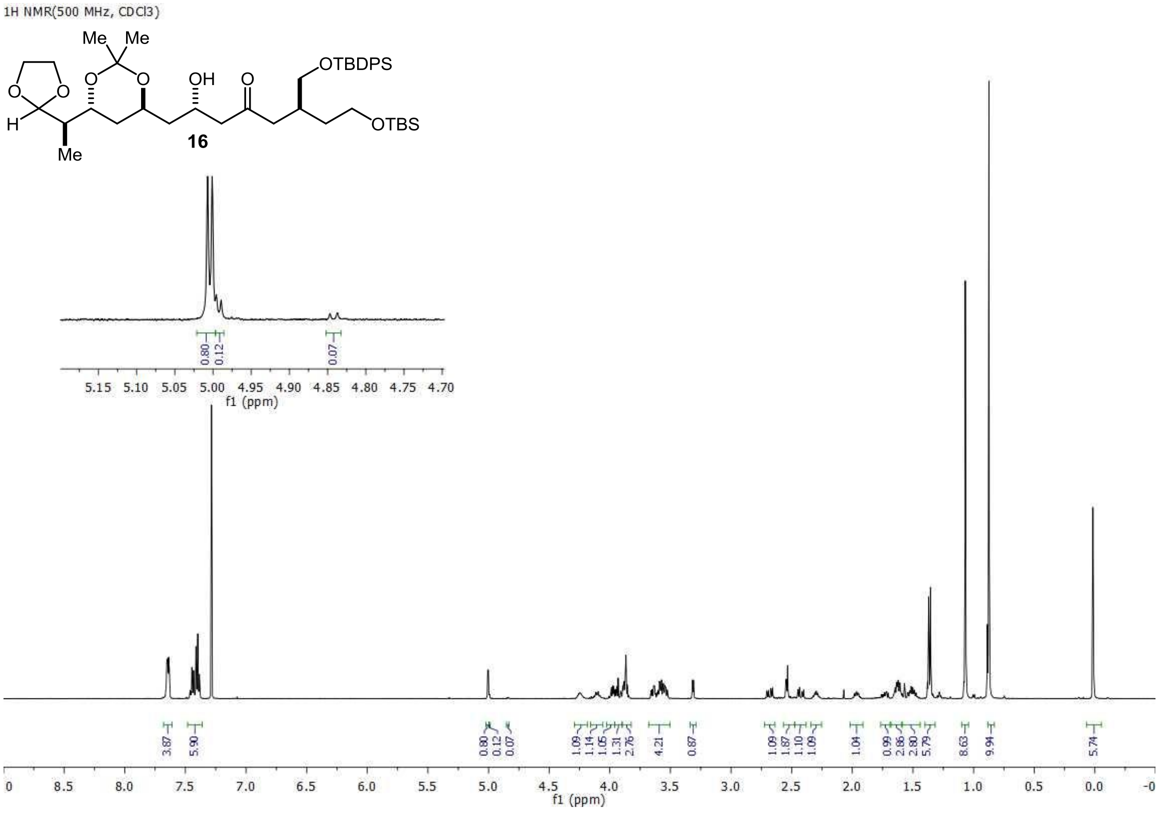

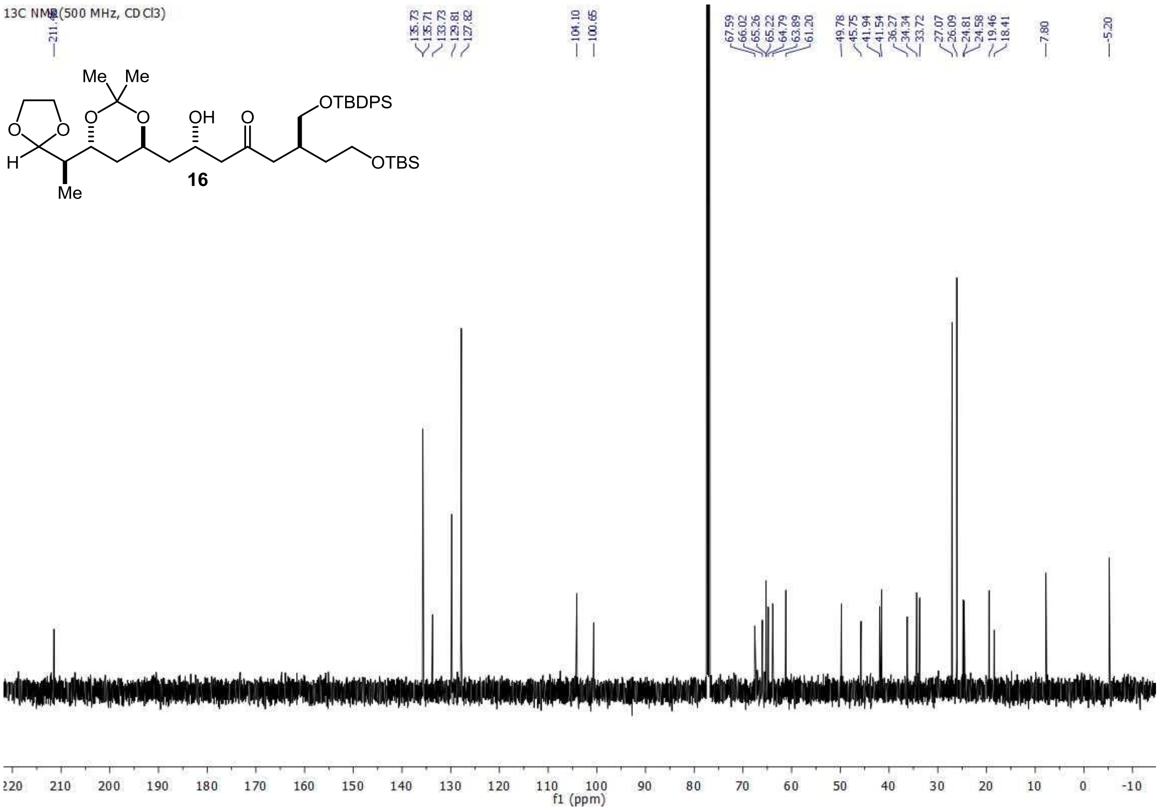

Supplementary Figure 37. ^{1}H NMR and ^{13}C NMR of **16**.

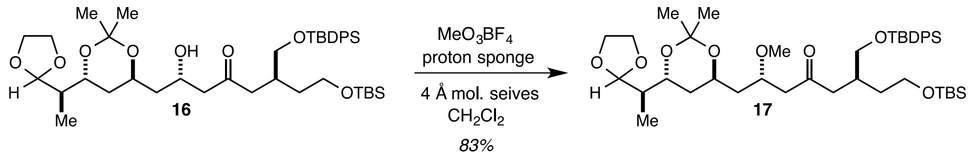

Supplementary Figure 38. Synthesis of **17**.

To a solution of **16** (150 mg, 0.22 mmol) in CH_2Cl_2 (4 mL) was added 4 Å MS (170 mg), proton sponge (330 mg, 1.54 mmol), and $Me_3O{\bullet}BF_4$ (195 mg, 1.32 mmol). After 1.5 h the mixture was filtered through celite, with EtOAc rinses. The filtrate was washed with 1 M AcOH (1 x 75 mL) and saturated aqueous $NaHCO_3$ (1 x 50 mL). The organic layer was dried over $MgSO_4$, filtered, and concentrated. The residue was purified by silica gel column chromatography eluting with 0:100 → 20:80 EtOAc:Hexanes to give **17** as a colorless oil (125 mg, 0.18 mmol, 83% yield).

OR $[\alpha]_D^{22}$ +7.4º (*c* 1.0, $CHCl_3$)

IR (thin film, cm^{-1}) 2929, 2856, 1711, 1464, 1428, 1379, 1253, 1224, 1092, 834, 703, 505

^{1}H NMR (500 MHz, $CDCl_3$) δ 7.66 – 7.59 (m, 4H), 7.41 (d, *J* = 7.0 Hz, 2H), 7.37 (t, *J* = 7.3 Hz, 4H), 4.98 (d, *J* = 3.0 Hz, 1H), 4.00 – 3.87 (m, 3H), 3.87 – 3.77 (m, 4H), 3.63 (dd, *J* = 10.0, 4.7 Hz, 1H), 3.60 – 3.48 (m, 3H), 3.32 (s, 3H), 2.65 (ddd, *J* = 28.2, 16.3, 6.8 Hz, 2H), 2.49 – 2.37 (m, 2H), 2.28 (h, *J* = 6.1 Hz, 1H), 1.93 (pd, *J* = 7.0, 3.1 Hz, 1H), 1.79 – 1.64 (m, 1H), 1.64 – 1.38 (m, 6H), 1.33 (d, *J* = 2.1 Hz, 6H), 1.05 (s, 9H), 0.85 (s, 11H), -0.01 (d, *J* = 2.2 Hz, 6H)

^{13}C NMR (126 MHz, $CDCl_3$) δ 209.1, 135.8, 135.7, 133.8, 129.8, 127.8, 104.1, 100.6, 74.4, 67.5, 66.1, 65.3, 65.2, 63.4, 61.3, 57.9, 48.5, 46.1, 41.7, 41.6, 36.8, 34.4, 33.7, 27.1, 26.1, 24.8, 24.6, 19.5, 18.4, 7.8, -5.2

HRMS (ESI+) calculated for $C_{42}H_{74}O_8Si_2N$ $[M+H]^+$: 788.4953; found 788.4954

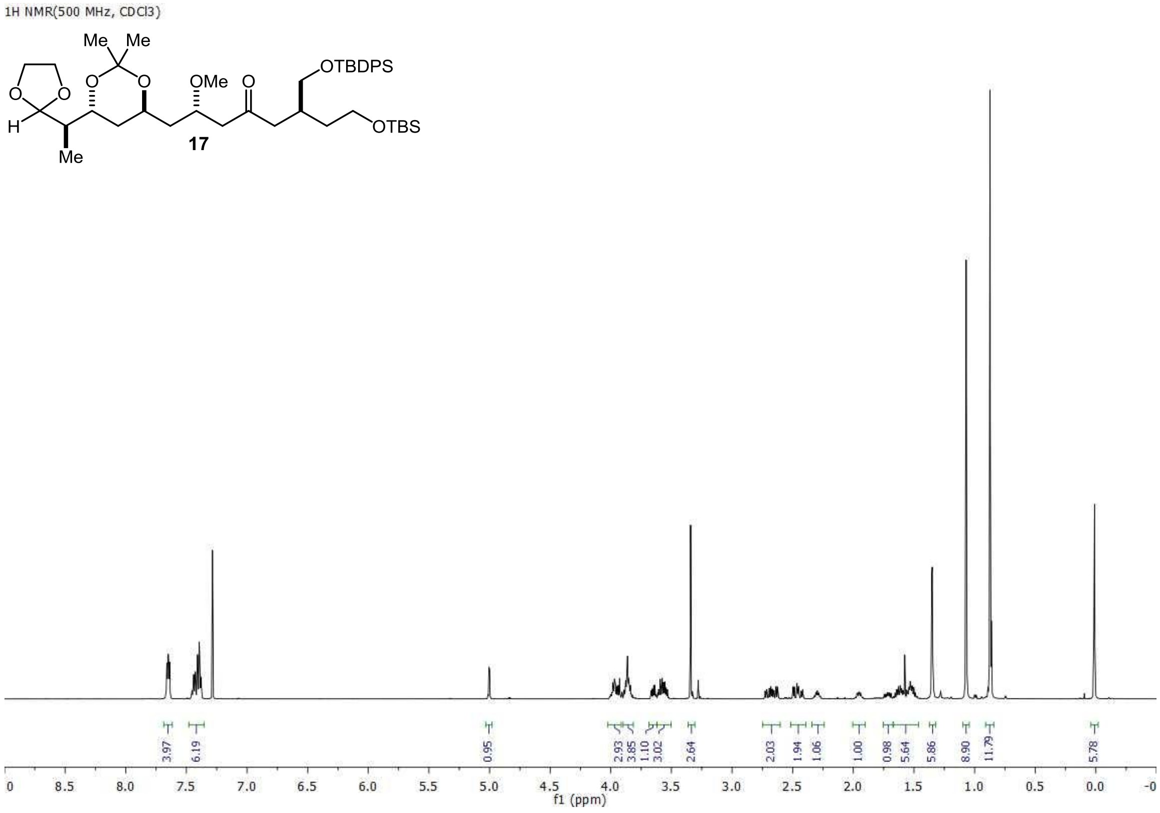

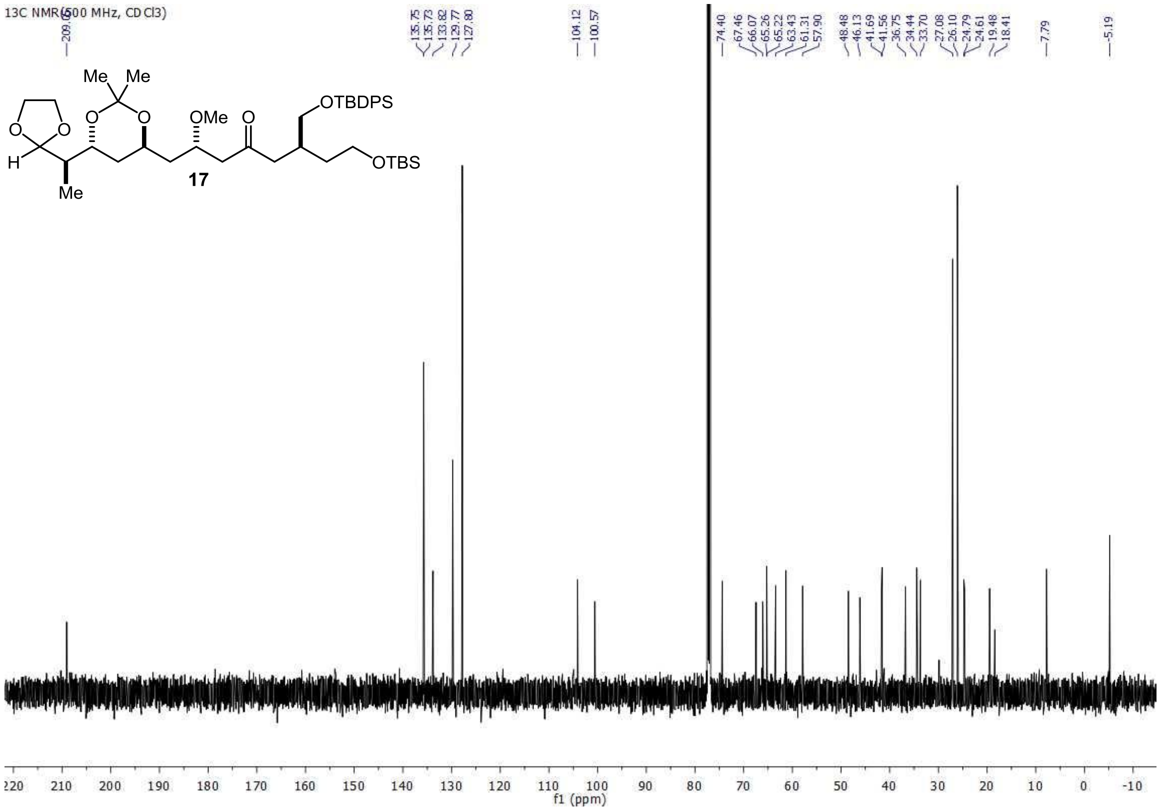

Supplementary Figure 39. ^{1}H NMR and ^{13}C NMR of **17**.

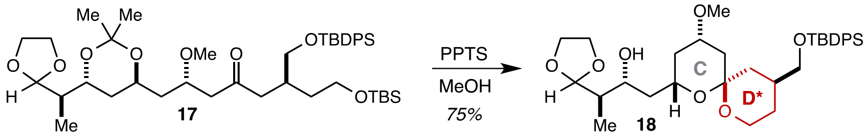

Supplementary Figure 40. Synthesis of **18**.

To a solution of ketone **17** (35 mg, 0.048 mmol) in MeOH (0.55 mL) was added pyridinium *p*-toluene sulfonate (12 mg, 0.048 mmol). After 2 h, the reaction mixture was quenched by the addition of saturated aqueous $NaHCO_3$ (1.0 mL). The mixture was extracted with CH_2Cl_2 (4 x 5 mL). The combined organic layers were dried over $MgSO_4$, filtered, and concentrated. Purification of the residue by silica gel column chromatography eluting with 0:100 → 50:50 EtOAc:Hexanes gave product **18** as a colorless oil (18 mg, 0.036 mmol, 75% yield).

OR $[\alpha]_D^{21}$ +38.3° (*c* 0.5, $CHCl_3$)

IR (thin film, cm^{-1}) 3488, 3070, 2929, 2856, 1673, 1464, 1427, 1386, 1058, 704

^{1}H NMR (500 MHz, $CDCl_3$) δ 7.64 (dt, *J* = 6.6, 1.6 Hz, 4H), 7.44 – 7.34 (m, 7H), 4.94 (d, *J* = 4.5 Hz, 1H), 4.05 – 3.96 (m, 4H), 3.96 – 3.89 (m, 1H), 3.89 – 3.81 (m, 2H), 3.80 – 3.61 (m, 4H), 3.52 (dd, *J* = 9.9, 5.0 Hz, 1H), 3.42 (dd, *J* = 10.0, 6.2 Hz, 1H), 3.33 (s, 3H), 3.29 (d, *J* = 3.8 Hz, 1H), 2.15 (s, 1H), 2.09 (ddd, *J* = 12.5, 4.7, 1.8 Hz, 1H), 2.01 – 1.90 (m, 1H), 1.86 (td, *J* = 7.1, 4.5 Hz, 1H), 1.79 – 1.69 (m, 2H), 1.69 – 1.55 (m, 4H), 1.43 – 1.35 (m, 1H), 1.35 – 1.25 (m, 3H), 1.25 – 1.13 (m, 4H), 1.05 (s, 10H), 0.97 (d, *J* = 7.0 Hz, 3H)

^{13}C NMR (126 MHz, $CDCl_3$) δ 135.8, 135.8, 134.0, 133.9, 129.7, 127.8, 106.5, 97.7, 73.4, 69.5, 68.7, 65.1, 65.0, 65.0, 60.3, 55.7, 42.6, 41.8, 40.7, 38.7, 37.7, 33.0, 28.6, 27.1, 19.5, 11.2

HRMS (ESI+) calculated for $C_{34}H_{51}O_7Si$ $[M+H]^+$: 599.3404; found 599.3400

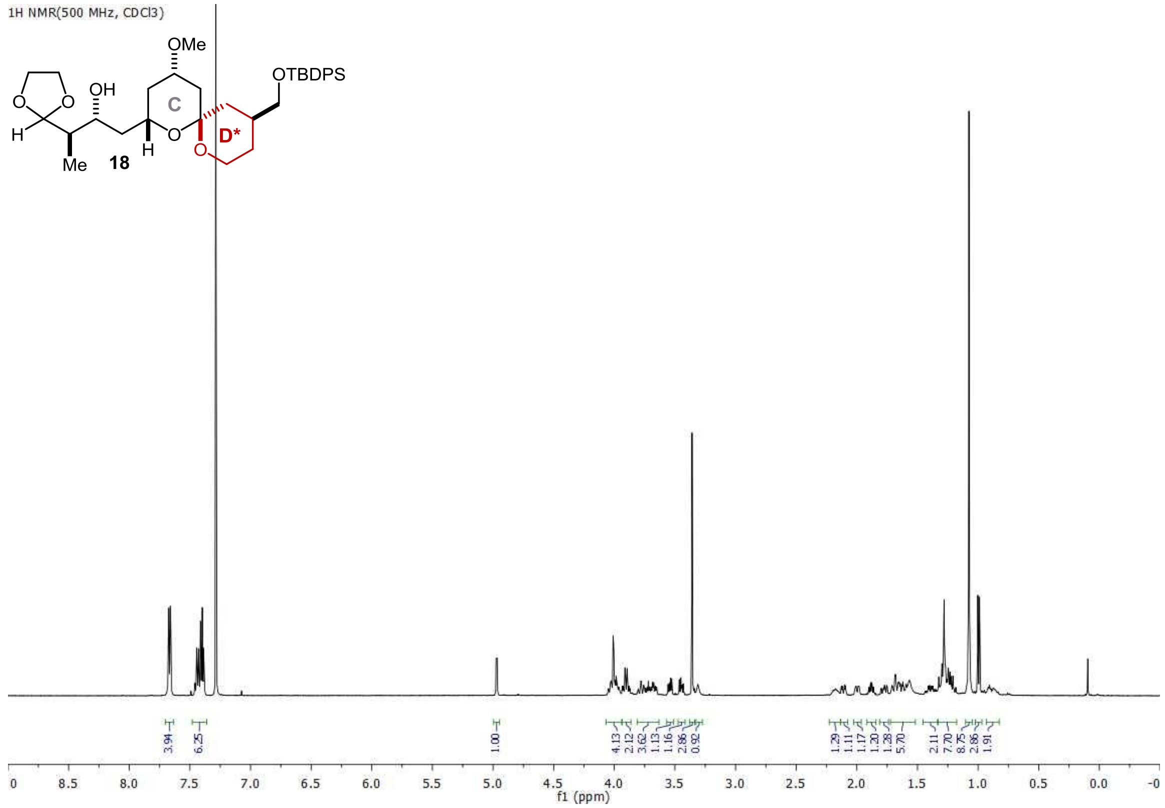

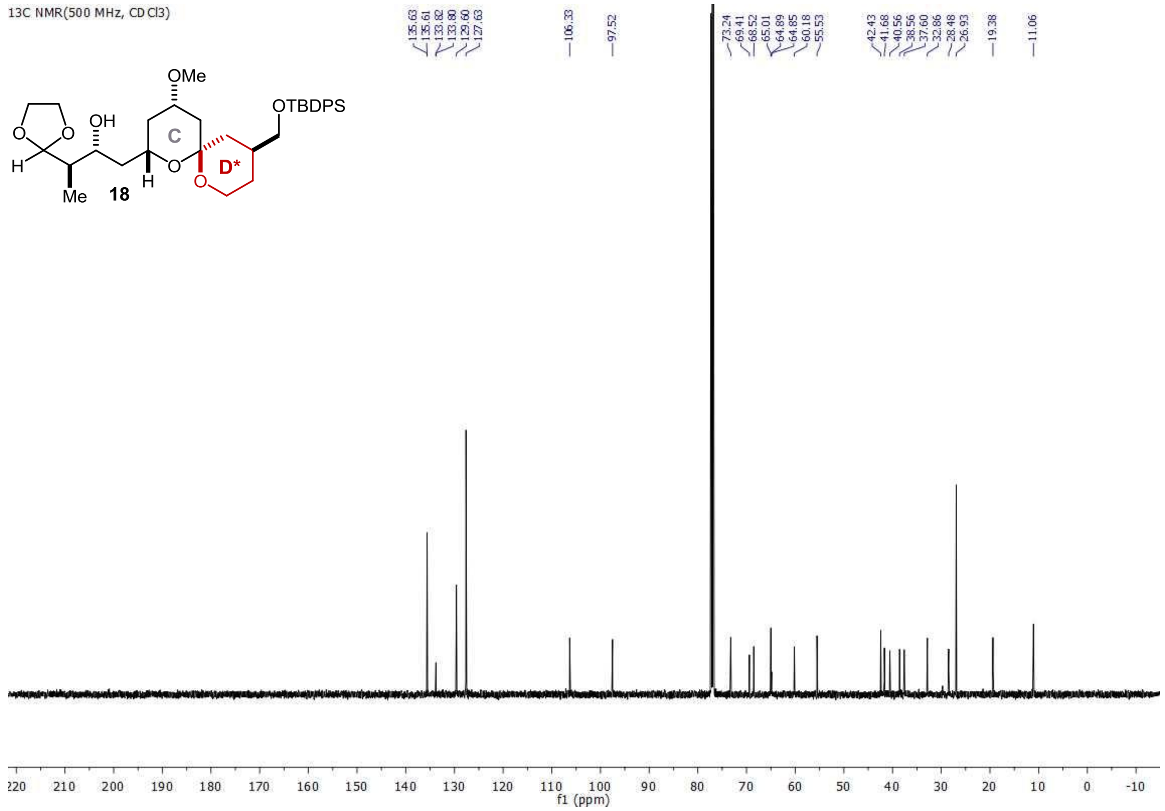

Supplementary Figure 41. ^{1}H NMR and ^{13}C NMR of **18**.

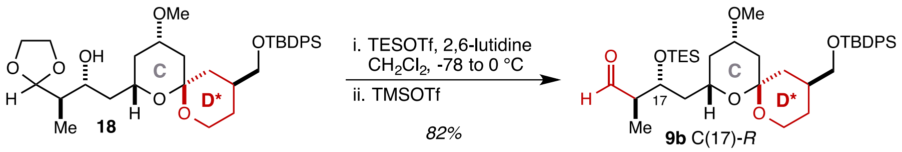

Supplementary Figure 42. Synthesis of **9b**.

To a cooled (-78 °C) solution of alcohol **18** (8 mg, 0.0136 mmol) in CH_2Cl_2 (200 µL) was added 2,6-lutidine (12 µL, 0.136 mmol) and TESOTf (3.2 µL, 0.014 mmol). After 4h, TMSOTf (6 µL, 0.0325 mmol) was added, and the reaction mixture was warmed to 0 °C. After 3h, the reaction mixture was quenched by the addition of water (150 µL). The mixture was stirred vigorously for 2h, and then the layers were separated and the aqueous layer was extracted with CH_2Cl_2 (5 x 0.5 mL). The combined organic layers were dried over Na_2SO4, filtered, and concentrated. The crude product was purified by chromatography with pH 7.0 buffered silica gel eluting with 0:100 → 15:85 EtOAc:Hexanes to provide aldehyde **9b** (8 mg, 0.011 mmol, 82% yield) as a colorless oil.

OR $[\alpha]_D^{25}$ +27.5° (*c* 1.0, $CHCl_3$)

IR (thin film, cm^{-1}) 2954, 2933, 2876, 1724, 1461, 1428, 1385, 1239, 1187, 1107, 1093, 975, 822, 740, 703, 505

^{1}H NMR (500 MHz, $CDCl_3$) δ 9.79 (d, *J* = 1.8 Hz, 1H), 7.72 – 7.57 (m, 4H), 7.49 – 7.32 (m, 6H), 4.19 – 4.18 (m, 1H), 3.81 – 3.71 (m, 1H), 3.71 – 3.56 (m, 3H), 3.52 (dd, *J* = 9.9, 5.1 Hz, 1H), 3.42 (dd, *J* = 9.9, 6.4 Hz, 1H), 3.34 (s, 3H), 2.67 – 2.65 (m, 1H), 2.18 – 2.03 (m, 2H), 2.02 – 1.98 (m, 1H), 1.85 (ddd, *J* = 14.1, 8.1, 5.8 Hz, 1H), 1.74 – 1.59 (m, 3H), 1.45 – 1.31 (m, 1H), 1.29 – 1.20 (m, 2H), 1.20 – 1.10 (m, 4H), 1.05 (s, 9H), 0.97 (t, *J* = 8.0 Hz, 9H), 0.63 (q, *J* = 8.0 Hz, 6H)

^{13}C NMR (126 MHz, $CDCl_3$) δ 204.5, 135.7, 135.7, 133.9, 133.9, 129.7, 127.8, 97.7, 73.1, 71.9, 68.6, 66.1, 60.5, 55.7, 52.3, 42.4, 41.6, 38.6, 38.1, 33.1, 28.6, 27.0, 19.5, 10.6, 7.1, 5.4

HRMS (ESI+) calcd for $C_{38}H_{60}O_6Si_2Na$ $[M+Na]^+$: 691.3826; found 691.3816

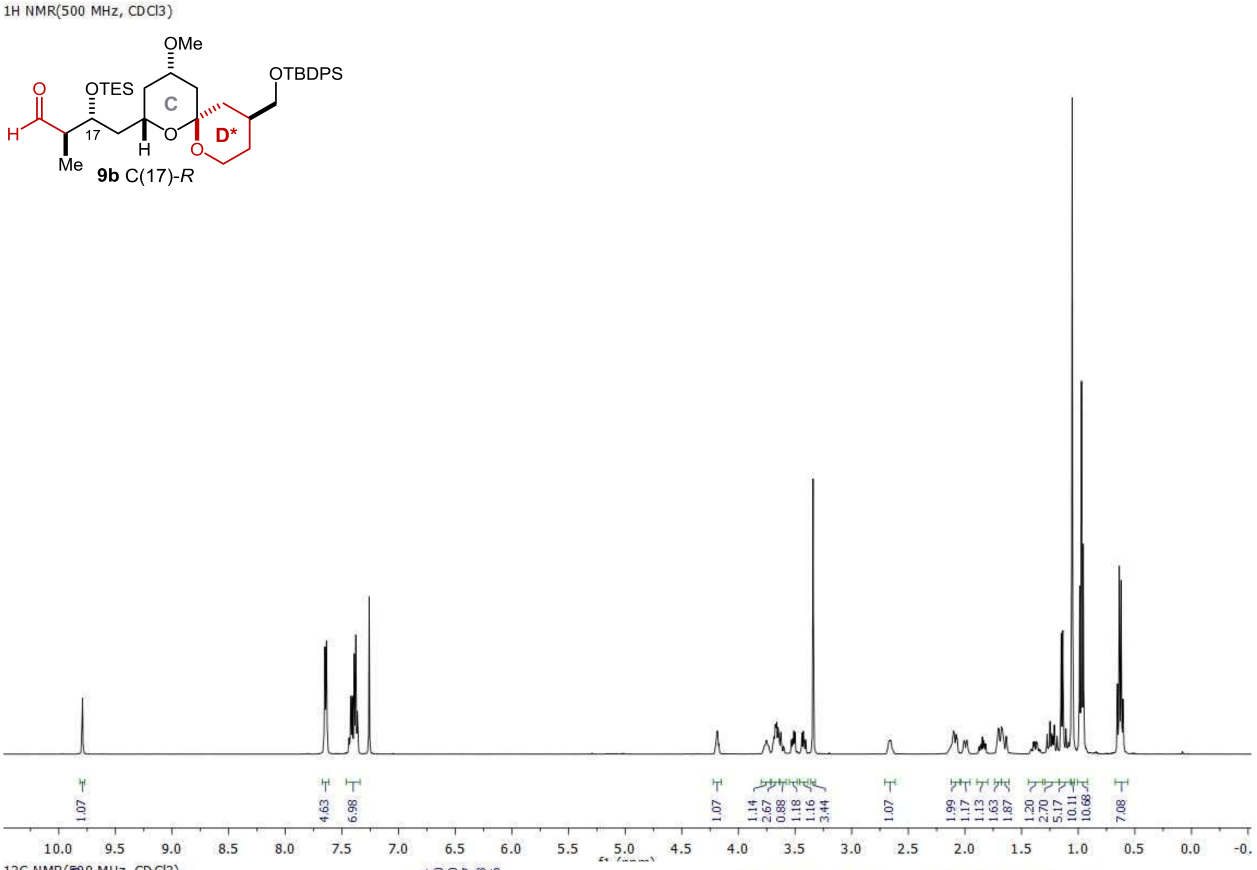

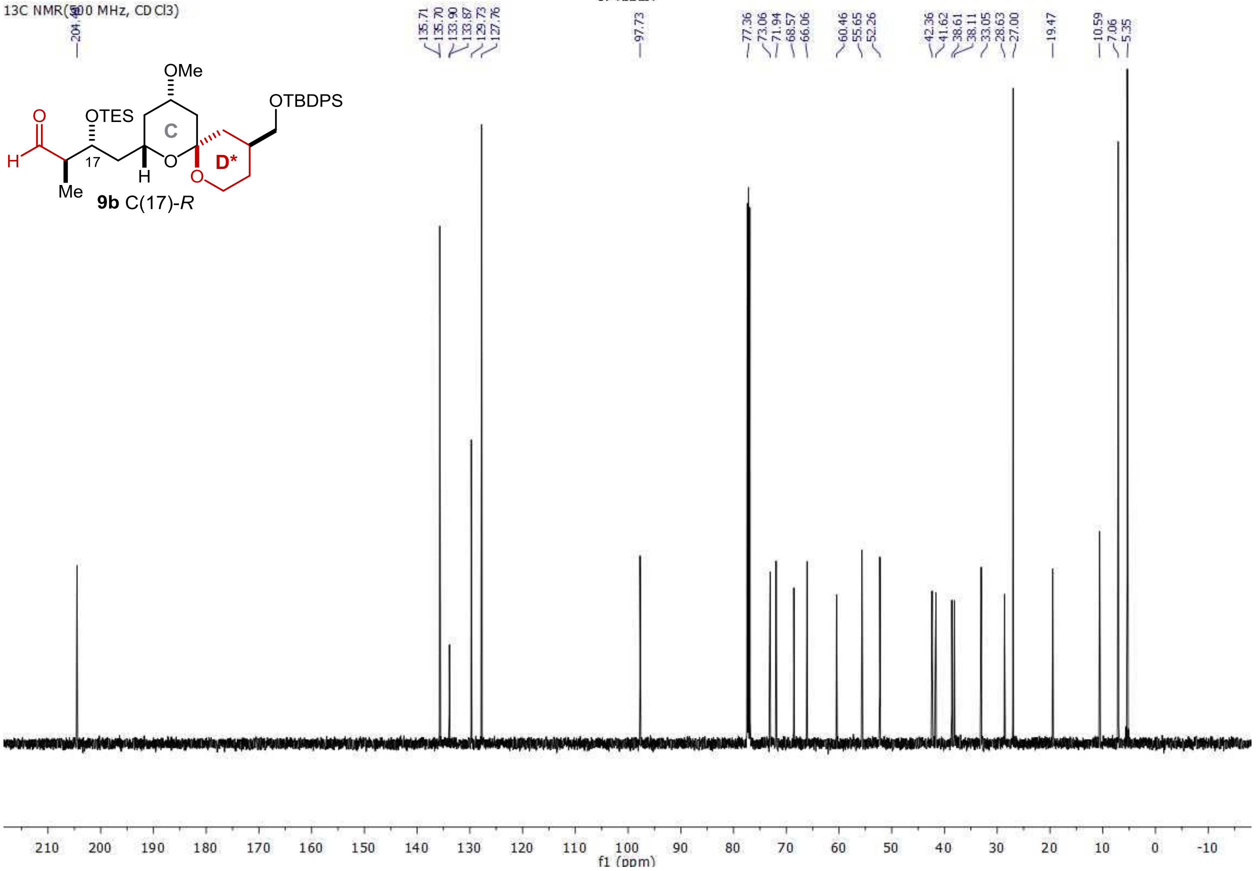

Supplementary Figure 43. ^{1}H NMR and ^{13}C NMR of **9b**.

Synthesis of aldehyde **9a**

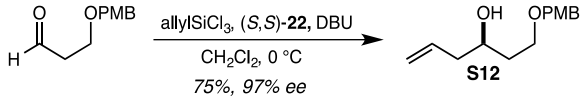

Supplementary Figure 44. Synthesis of **S12**.

Alcohol **S12** was prepared using a modified literature procdure.[5] To a cooled (0 °C) solution of (*S*,*S*)-**22** (20.3 g, 70.0 mmol) in CH_2Cl_2 (250 mL) was added DBU (31.4 mL, 210 mmol). Allyltrichlorosilane (11.2 mL, 77 mmol) was then added slowly. The ice/water bath was removed and after 1 h the mixture was re-cooled to 0 °C. 3-((4-methoxybenzyl)oxy)propanal (13.6 g, 70.0 mmol, prepared as described here[8]) was added and the resulting mixture was stirred at 0 °C for 1 h. The mixture was concentrated and the residue was suspended in Et_2O (250 mL). The mixture was stirred vigorously for 20 min to ensure complete precipitation of the DBU•HCl salts. The mixture was then filtered through a frit, and the filtrate was treated with TBAF (70.0 mL, 70.0 mmol, 1 M in THF). After 2.5 h, 1 M HCl (350 mL, 350 mmol) was added and the mixture was transferred to a separatory funnel. The layers were separated and the aqueous layer was extracted with Et_2O (3 x 100 mL). The combined organic layers were washed with H_2O (2 x 50 mL) and saturated aqueous $NaHCO_3$ (1 x 100 mL), dried over $MgSO_4$, filtered and concentrated. The residue was purified by chromatography on silica gel to provide pure **S12** as a pale yellow oil (12.3 g, 52.2 mmol, 75% yield, 97% ee). **^{1}H NMR** (400 MHz, $CDCl_3$) δ 7.28 (m, 2H), 7.01 – 6.79 (m, 2H), 5.86 (ddt, *J* = 17.4, 10.4, 7.1 Hz, 1H), 5.20 – 5.05 (m, 2H), 4.48 (s, 2H), 3.93 – 3.85 (m, 1H), 3.83 (s, 3H), 3.72 (dt, *J* = 9.3, 5.3 Hz, 1H), 3.65 (ddd, *J* = 9.3, 6.7, 5.4 Hz, 1H), 2.88 (s, 1H), 2.27 (ddt, *J* = 7.4, 6.3, 1.3 Hz, 2H), 1.84 – 1.67 (m, 2H). The ^{1}H NMR spectroscopic data is in agreement with data reported in literature.[9] The enantiomeric excess was determined by chiral HPLC analysis: OD-H column, 98:2 hexanes:*i*PrOH, 1 mL/min, 254 nm, where the desired *R*-enantiomer **S12** has been established to elute second.[9]

To recover (*S*,*S*)-**22**, the combined aqueous layers from above were treated with 3 M NaOH (240 mL) and extracted with CH_2Cl_2 (5 x 100 mL). The combined organic layers were washed with water (2 x 100 mL), dried over $MgSO_4$, filtered, and concentrated. The residue was dissolved in minimal hot 9:1 MeOH:H_2O (during the dissolution process the temperature should not be allowed to exceed 80 °C, as the ligand may start to undergo decomposition at higher temperatures). The hot saturated solution was allowed to cool to room temperature, and distilled water (10.0 mL) was added to ensure complete crystallization of **22**. The white solid was collected by filtration through a frit with a cold 1:1 MeOH:H_2O rinse and then dried *in vacuo* at 45 °C to give recovered diaminophenol **22**.

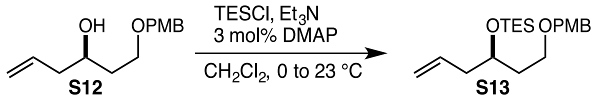

Supplementary Figure 45. Synthesis of **S13**.

To a cooled (0 °C) solution of **S12** (17.8 g, 75.2 mmol) in CH_2Cl_2 (225 mL) was added Et_3N (13.6 mL, 97.7 mmol) followed by the slow addition of TESCl (12.6 mL, 75.2 mmol) and DMAP (0.28 g, 2.2 mmol). The resulting solution was stirred at room temperature for 2 h. MeOH (3.0 mL) was added to quench unreacted TESCl and the resulting mixture was concentrated. The residue was suspended in hexanes and the Et_3N•HCl salts were removed by filtration. The filtrate was concentrated to give crude **S13** as a pale orange oil (26.6 g, 76 mmol), which was used without further purification. **^{1}H NMR** (500 MHz, $CDCl_3$) δ 7.34 – 7.24 (m, 2H), 6.95 – 6.84 (m, 2H), 5.84 (ddt, *J* = 16.3, 11.1, 7.1 Hz, 1H), 5.11 – 5.01 (m, 2H), 3.92 (m, 1H), 3.83 (s, 3H), 3.61 – 3.46 (m, 2H), 2.38 (s, 1H), 2.25 (m, 2H), 1.81 (m, 1H), 1.71 (m, 1H), 0.98 (t, *J* = 8.0 Hz, 9H), 0.62 (q, *J* = 7.7 Hz, 6H).

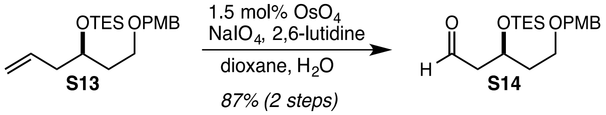

Supplementary Figure 46. Synthesis of **S14**.

To a solution of unpurified **S13** (15.5 g, 44.2 mmol) in dioxane:H_2O (330 mL:110 mL) were sequentially added 2,6-lutidine (6.7 mL, 57.5 mmol), OsO_4 (168 mg, 0.66 mmol), and $NaIO_4$ (21.7 g, 102 mmol). The resulting reaction mixture was stirred at room temperature for 2.5 h. The reaction mixture was cooled to 0 ºC and carefully quenched with saturated aqueous $Na_2S_2O_3$ (250 mL). The layers were separated and the aqueous layer was extracted with Et_2O (3 x 100 mL). The combined organic layers were dried over $MgSO_4$, filtered and concentrated. The residue was purified by silica gel column chromatography eluting with 5:95 → 15:85 EtOAc:Hexanes to give **S14** as a pale yellow oil (13.5 g, 38.3 mmol, 87% yield from **S12** over 2 steps).

OR $[\alpha]_D^{19}$ -6.1° (*c* 1.0, $CHCl_3$)

IR (thin film, cm^{-1}) 2953, 2876, 1724, 1612, 1513, 1461, 1366, 1247, 1174, 1094, 1027, 820, 742

^{1}H NMR (500 MHz, $CDCl_3$) δ 9.78 (t, *J* = 2.3 Hz, 1H), 7.30 – 7.16 (m, 2H), 6.92 – 6.82 (m, 2H), 4.47 – 4.33 (m, 3H), 3.79 (s, 3H), 3.58 – 3.42 (m, 2H), 2.63 – 2.45 (m, 2H), 1.93 – 1.73 (m, 1H), 0.94 (t, *J* = 8.0 Hz, 9H), 0.60 (q, *J* = 8.0 Hz, 6H)

^{13}C NMR (126 MHz, $CDCl_3$) δ 202.0, 159.3, 130.5, 129.4, 113.9, 72.8, 66.2, 65.7, 55.3, 51.3, 37.9, 6.9, 5.0

HRMS (FAB+) calculated for $C_{19}H_{31}O_4Si$ $[M+H]^+$: 351.1992; found 351.1994

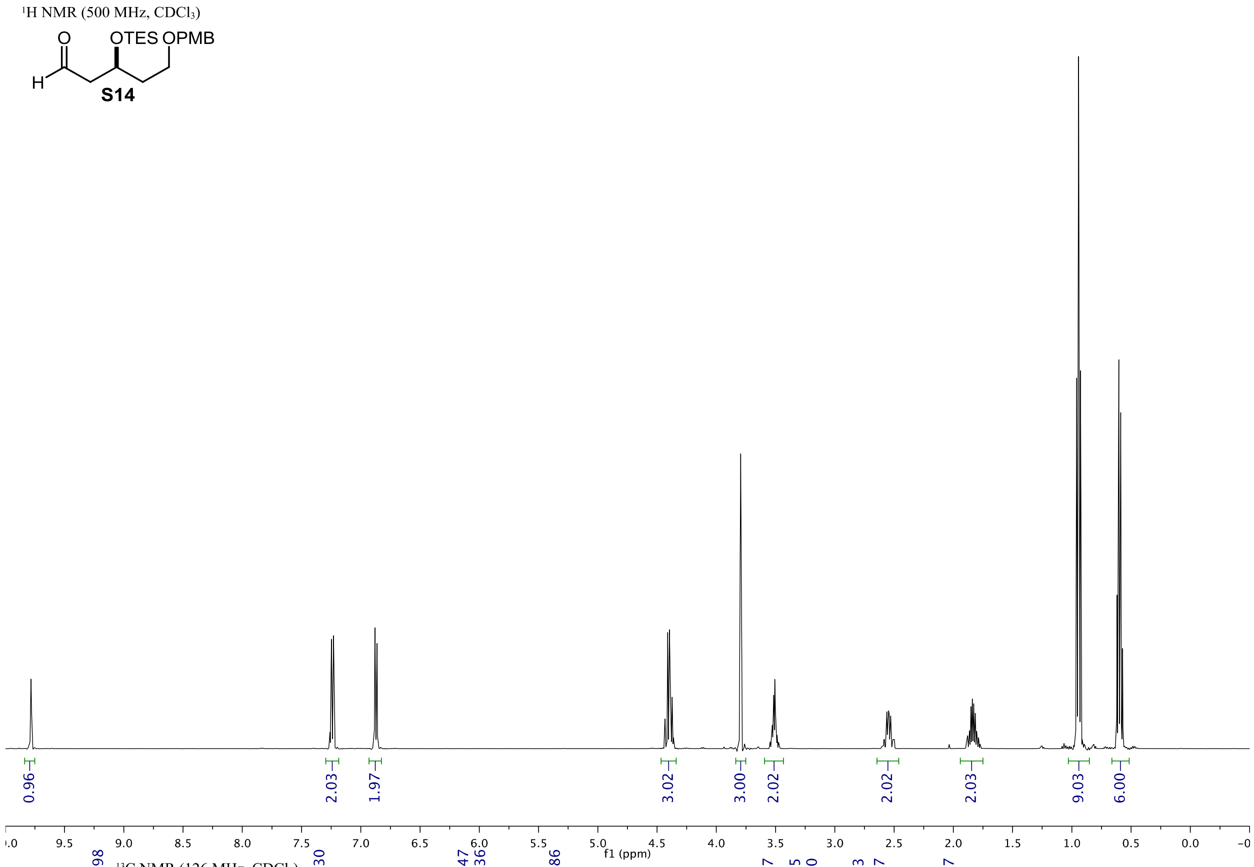

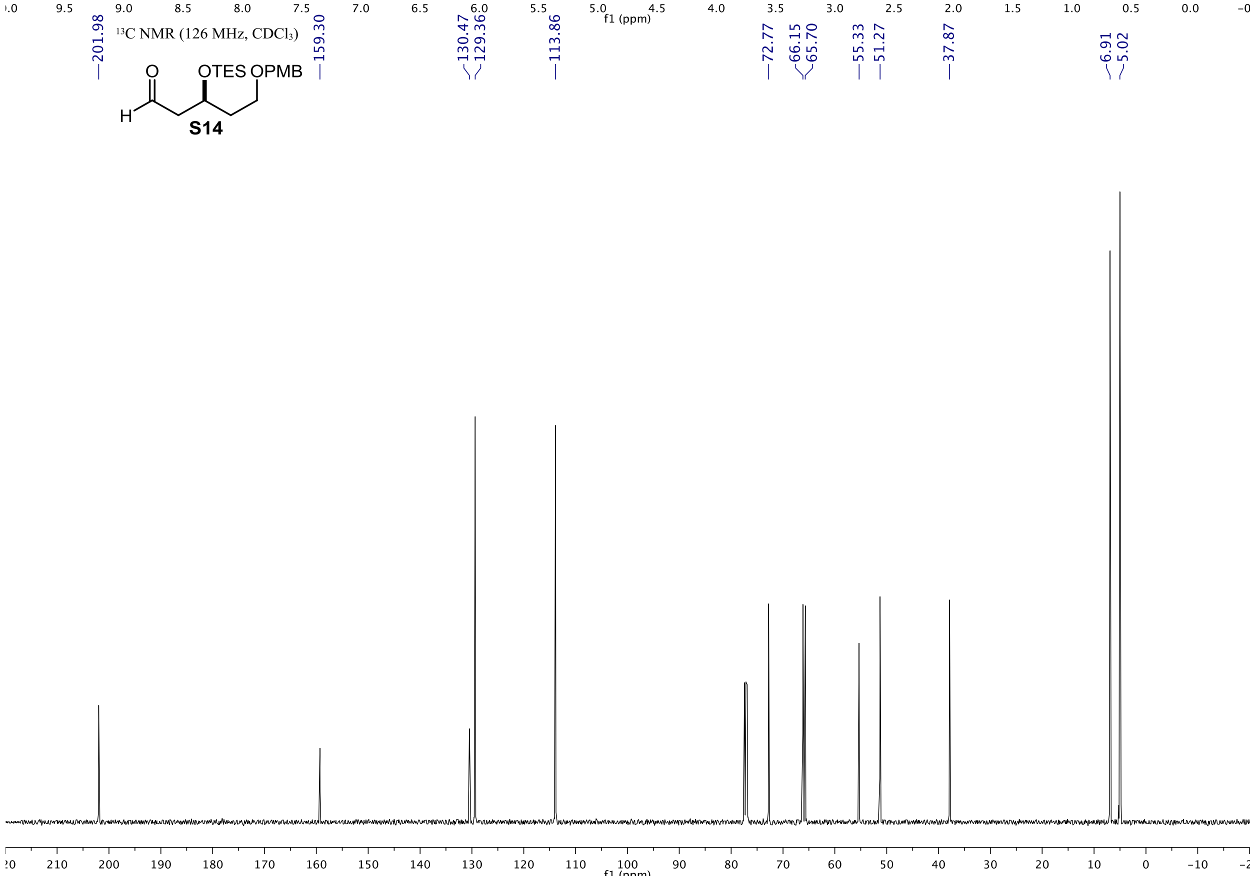

Supplementary Figure 47. ^{1}H NMR and ^{13}C NMR of **S14**.

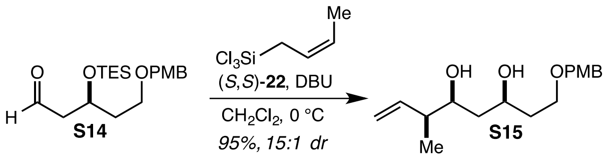

Supplementary Figure 48. Synthesis of **S15**.

Diol **S14** was prepared using a modified literature procdure.[5] To a cooled (0 °C) solution of (*S*,*S*)-**22** (8.02 g, 27.6 mmol) in CH_2Cl_2 (100 mL) was added DBU (12.4 mL, 82.8 mmol). The *cis*-crotyltrichlorosilane (4.6 mL, 30.4 mmol, prepared as described here[5]) was then added slowly. The ice/water bath was removed and after 1 h the mixture was re-cooled to 0 °C. Aldehyde **S14** (9.13 g, 25.9 mmol) was added and the resulting solution was stirred at 0 °C for 1 h. The mixture was concentrated and the residue was resuspended in Et_2O (100 mL). The mixture was stirred vigorously for 20 min to ensure complete precipitation of the DBU•HCl salts. The mixture was then filtered through a frit, and the filtrate was treated with TBAF (52.0 mL, 52.0 mmol, 1 M in THF). After 2.5 h, 1 M HCl (140 mL, 140 mmol) was added and the mixture was transferred to a separatory funnel. The layers were separated and the aqueous layer was extracted with Et_2O (3 x 50 mL). The combined organic layers were washed with H_2O (2 x 25 mL) and saturated aqueous $NaHCO_3$ (1 x 50 mL), dried over $MgSO_4$, filtered, and concentrated. The residue was purified by chromatography on silica gel to provide **S15** as a pale yellow oil (7.24 g, 24.6 mmol, 95% yield, 15:1 dr).

OR $[\alpha]_D^{19}$ -6.1° (*c* 1.0, $CHCl_3$)

IR (thin film, cm^{-1}) 3378, 2938, 2863, 1612, 1513, 1421, 1301, 1246, 1174, 1089, 1035, 915, 820, 570

^{1}H NMR (500 MHz, $CDCl_3$) δ 7.28 – 7.19 (m, 2H), 6.95 – 6.80 (m, 2H), 5.78 (ddd, *J* = 17.1, 10.6, 7.5 Hz, 1H), 5.09 – 4.98 (m, 2H), 4.44 (s, 2H), 4.09 – 3.98 (m, 1H), 3.88 – 3.81 (m, 1H), 3.80 (s, 3H), 3.75 – 3.69 (m, 1H), 3.69 – 3.64 (m, 1H), 3.64 – 3.54 (m, 2H), 2.35 – 2.15 (m, 1H), 1.86 – 1.65 (m, 2H), 1.58 (dt, *J* = 14.3, 2.3 Hz, 1H), 1.48 (dt, *J* = 14.4, 10.1 Hz, 1H), 1.03 (d, *J* = 6.9 Hz, 3H)

^{13}C NMR (126 MHz, $CDCl_3$) δ 159.4, 141.0, 130.0, 129.5, 115.1, 114.0, 75.8, 73.1, 72.7, 68.5, 55.4, 44.2, 40.0, 37.1, 15.0

HRMS (FAB+) calculated for $C_{17}H_{27}O_4$ $[M+H]^+$: 295.1909; found 295.1911

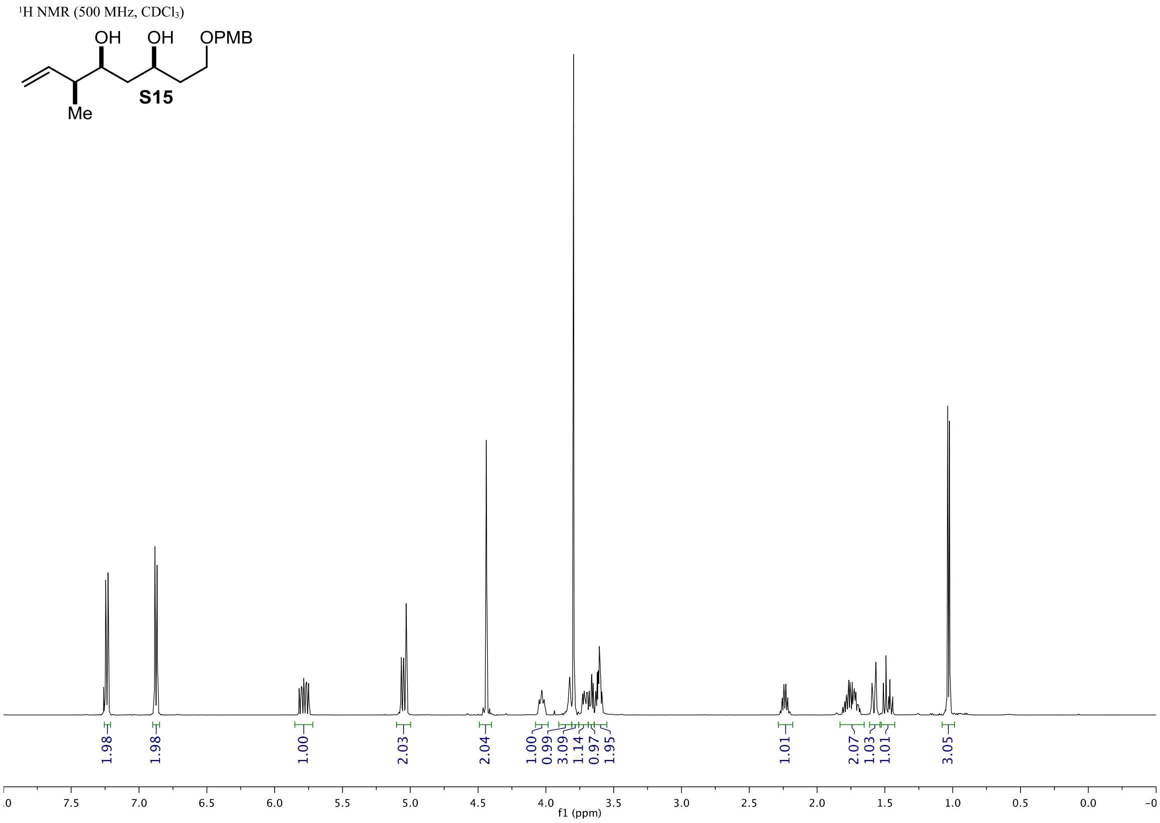

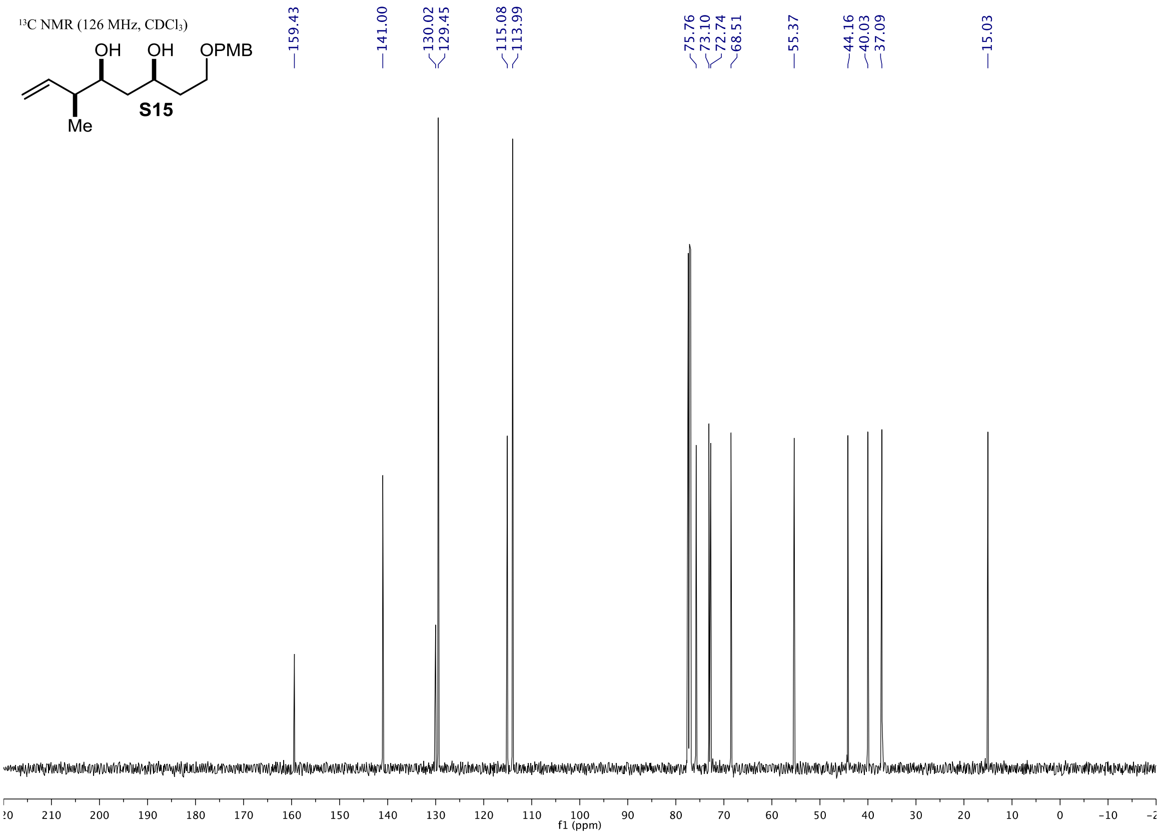

Supplementary Figure 49. ^{1}H NMR and ^{13}C NMR of **S15**.

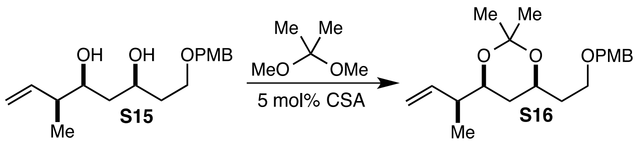

Supplementary Figure 50. Synthesis of **S16**.

To a solution of **S15** (9.27 g, 31.5 mmol) in dimethoxypropane (210 mL) was added camphor sulfonic acid (0.37 g, 1.6 mmol). The resulting solution was stirred at room temperature for 1 h. Saturated aqueous $NaHCO_3$ (75 mL) was added. The layers were separated and the aqueous layer was extracted with EtOAc (3 x 50 mL). The combined organic layers were washed with H_2O (1 x 75 mL), dried over Na_2SO_4, filtered, and concentrated to give crude **S16** as a pale yellow oil (10.4 g, 31.2 mmol) that was used without further purification. Purification for a characterization sample was achieved by silica gel column chromatography eluting with 0:100 → 10:90 EtOAc:Hexanes to give **S16** as a pale yellow oil.

OR $[\alpha]_D^{21}$ -16.3° (*c* 1.0, $CHCl_3$)

IR (thin film, cm^{-1}) 2859, 1612, 1512, 1461, 1378, 1301, 1247, 1200, 1174, 1097, 1035, 915, 872, 817, 522

^{1}H NMR (400 MHz, $CDCl_3$) δ 7.31 – 7.20 (m, 2H), 6.93 – 6.80 (m, 2H), 5.72 (ddd, *J* = 17.1, 10.4, 7.7 Hz, 1H), 5.11 – 4.95 (m, 2H), 4.42 (s, 2H), 4.04 – 3.91 (m, 1H), 3.80 (s, 3H), 3.64 – 3.42 (m, 3H), 2.24 – 2.10 (m, 1H), 1.82 – 1.60 (m, 2H), 1.46 (dt, *J* = 12.9, 2.5 Hz, 1H), 1.41 (s, 3H), 1.37 (s, 3H), 1.11 (dt, *J* = 12.6, 11.5 Hz, 1H), 1.02 (d, *J* = 6.7 Hz, 3H)

^{13}C NMR (101 MHz, $CDCl_3$) δ 159.3, 140.4, 130.8, 129.4, 115.0, 113.9, 98.6, 72.7, 72.6, 66.3, 66.1, 55.4, 43.5, 36.8, 34.8, 30.4, 20.0, 15.9

HRMS (FAB+) calculated for $C_{20}H_{29}O_4$ $[M+H]^+$: 333.2066; found 333.2060

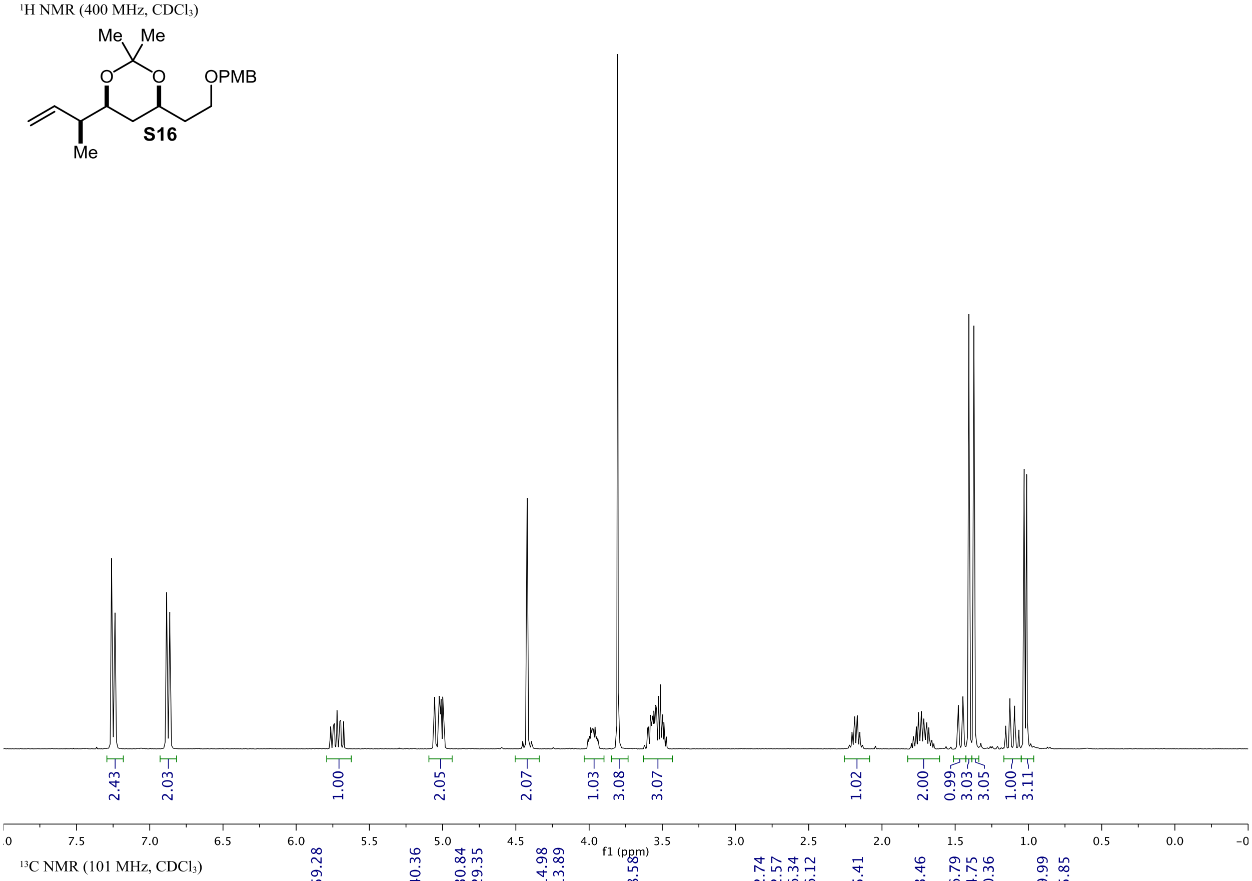

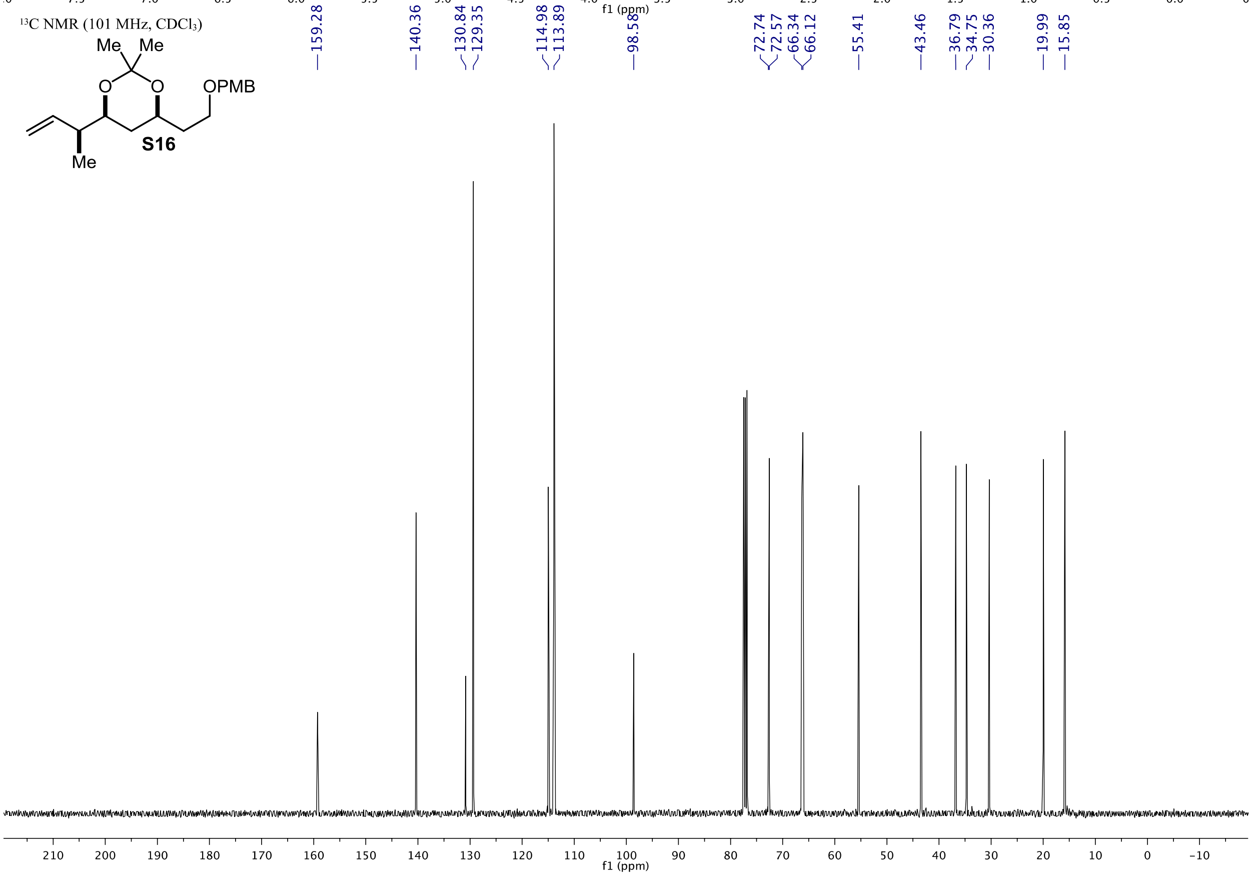

Supplementary Figure 51. ^{1}H NMR and ^{13}C NMR of **S16**.

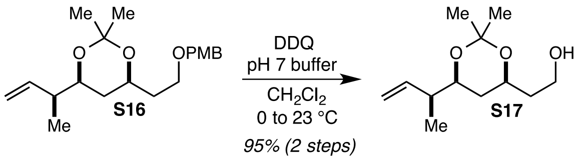

Supplementary Figure 52. Synthesis of **S17**.

To a solution of unpurified PMB ether **S16** (4.00 g, 12.0 mmol) in CH_2Cl_2 (106 mL) was added pH 7 buffer solution (14 mL). The resulting mixture was cooled to 0 °C. DDQ (4.07 g, 17.9 mmol) was added in 3 portions at a rate of 1 portion per 3 min. The resulting mixture was stirred at 0 °C for 5 min and then at room temperature for 1 h. The reaction mixture was filtered through celite, onto pH 7 buffer solution (100 mL), rinsing with CH_2Cl_2. The layers were separated and the aqueous layer was extracted with CH_2Cl_2 (3 x 50 mL). The combined organic layers were washed with saturated $NaHCO_3$ solution (1 x 100 mL) and H_2O (1 x 100 mL), dried over Na_2SO_4, filtered, and concentrated. The residue was purified by chromatography with pH 7 buffered silica gel eluting with 10:90 → 30:70 EtOAc:Hexanes to give **S17** as a pale yellow oil (2.46 g, 11.4 mmol, 95% yield from **S15** over 2 steps).

OR $[\alpha]_D^{21}$ -27.7° (*c* 1.0, $CHCl_3$)

IR (thin film, cm^{-1}) 3418, 2946, 1379, 1257, 1200, 1100, 1053, 998, 965, 916, 874

^{1}H NMR (400 MHz, $CDCl_3$) δ 5.70 (ddd, *J* = 17.2, 10.4, 7.8 Hz, 1H), 5.08 – 4.94 (m, 2H), 4.05 (dddd, *J* = 11.7, 7.3, 4.6, 2.6 Hz, 1H), 3.83 – 3.66 (m, 2H), 3.59 (ddd, *J* = 11.6, 7.3, 2.4 Hz, 1H), 2.60 (s, 1H), 2.24 – 2.08 (m, 1H), 1.80 – 1.62 (m, 2H), 1.48 – 1.40 (m, 1H), 1.43 (s, 3H), 1.37 (s, 3H), 1.23 (dt, *J* = 13.0, 11.5 Hz, 1H), 1.01 (d, *J* = 6.7 Hz, 3H)

^{13}C NMR (101 MHz, $CDCl_3$) δ 140.1, 115.2, 98.7, 72.4, 69.6, 61.0, 43.4, 38.3, 34.5, 30.3, 19.9, 15.8

HRMS (FAB+): calculated for $C_{12}H_{23}O_3$ 215.1647 $[M+H]^+$; found 215.1648

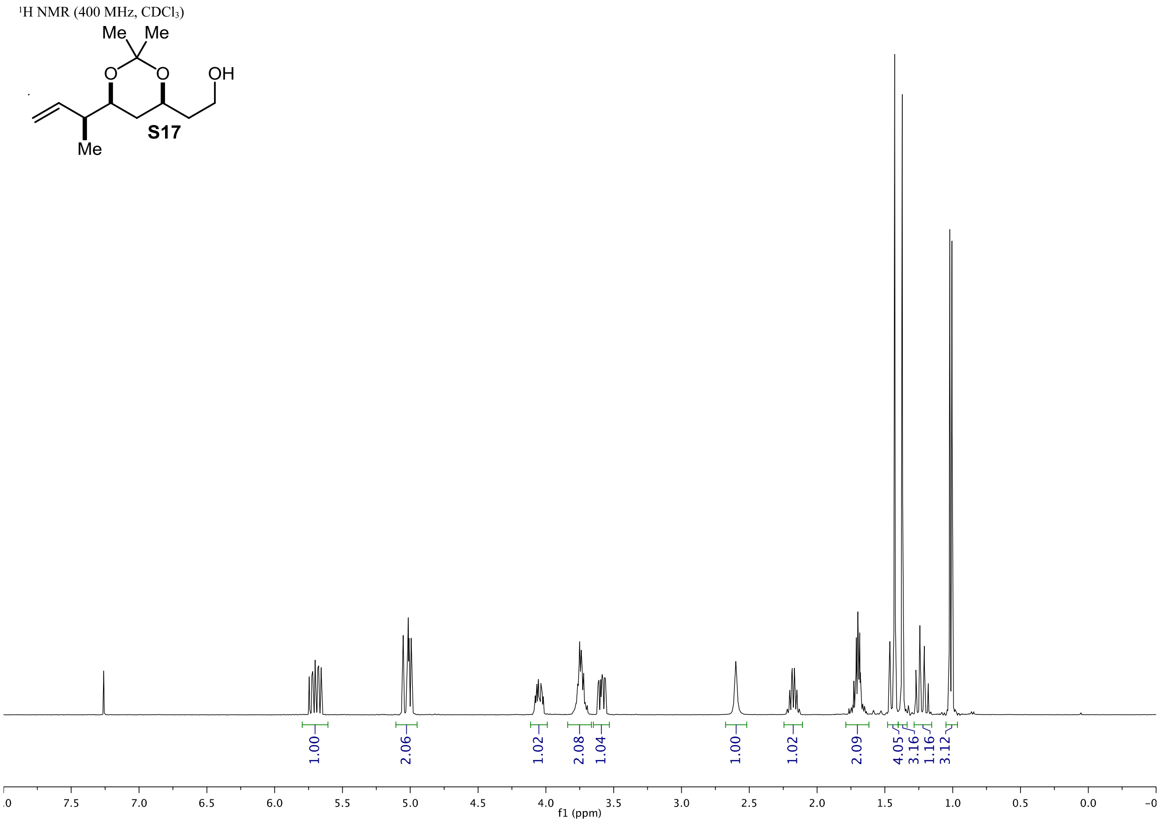

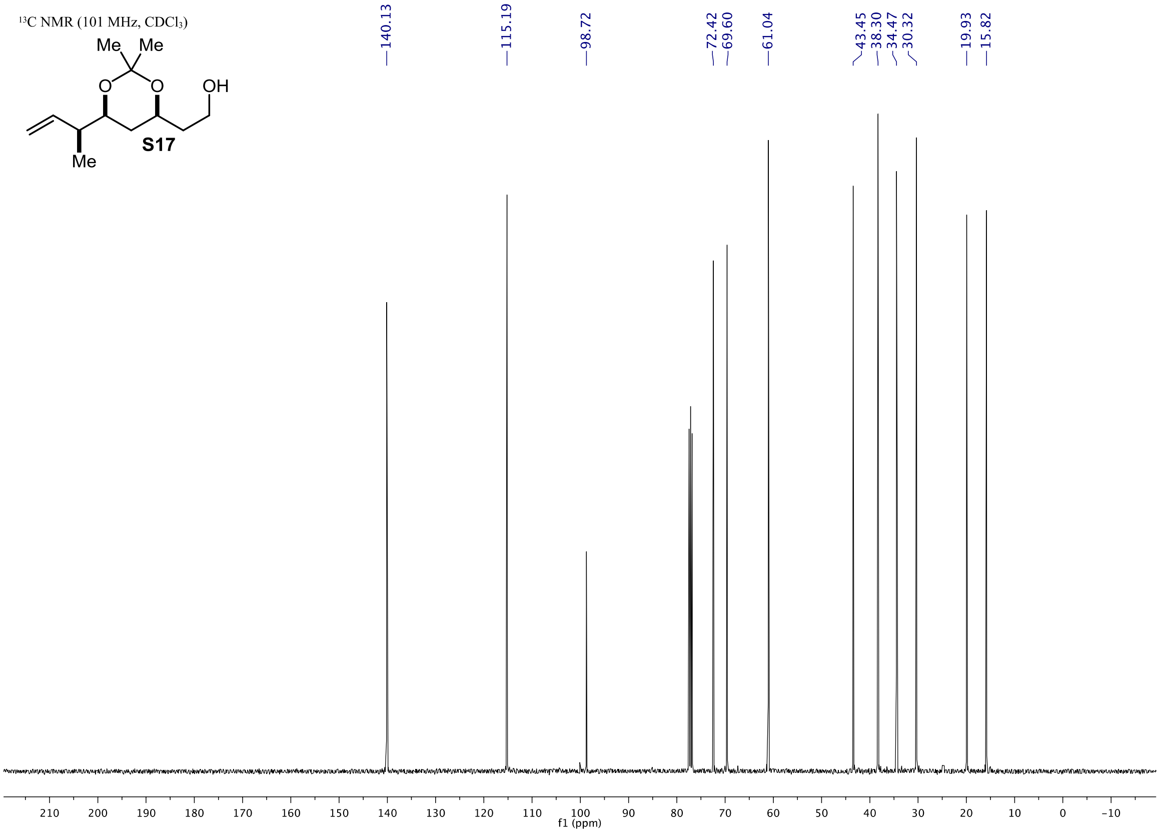

Supplementary Figure 53. ^{1}H NMR and ^{13}C NMR of **S17**.

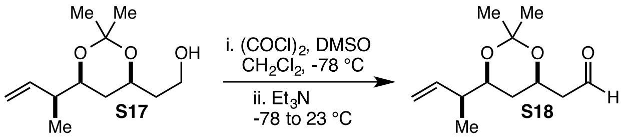

Supplementary Figure 54. Synthesis of **S18**.

To a cooled (-78 °C) solution of oxalyl chloride (4.4 mL, 52.4 mmol) in CH_2Cl_2 (175 mL) was added DMSO (7.4 mL, 105 mmol). After 20 min a solution of **S17** (5.61 g, 26.2 mmol) in CH_2Cl_2 (90 mL) was added slowly. After 20 min Et_3N (24.5 mL, 175 mmol) was added dropwise, and the mixture was then allowed to warm to room temperature. After 30 min the reaction mixture was quenched with water (100 mL) and the mixture was concentrated to remove the bulk of the CH2Cl2, and the residue was extracted with Et_2O (3 x 100 mL). The combined ether layers were washed with water (1 x 100 mL), dried over Na_2SO_4, filtered, and concentrated to give **S18** as a pale yellow oil (5.6 g, 26 mmol) which was used without further purification.

OR $[\alpha]_D^{22}$ -18.1° (*c* 0.8, $CHCl_3$)

IR (thin film, cm^{-1}) 2924, 2854, 1717, 1380, 1259, 1201, 1102, 965, 917, 771

^{1}H NMR (400 MHz, $CDCl_3$) δ 9.77 (dd, *J* = 2.3, 1.7 Hz, 1H), 5.70 (ddd, *J* = 17.1, 10.4, 7.8 Hz, 1H), 5.11 – 4.95 (m, 2H), 4.35 (dddd, *J* = 12.0, 7.4, 5.0, 2.6 Hz, 1H), 3.62 (ddd, *J* = 11.5, 7.3, 2.4 Hz, 1H), 2.60 (ddd, *J* = 16.6, 7.3, 2.3 Hz, 1H), 2.45 (ddd, *J* = 16.6, 5.0, 1.8 Hz, 1H), 2.25 – 2.12 (m, 1H), 1.55 (dt, *J* = 12.9, 2.5 Hz, 1H), 1.44 (s, 3H), 1.37 (s, 3H), 1.18 (dt, *J* = 12.8, 11.5 Hz, 1H), 1.02 (d, *J* = 6.8 Hz, 3H)

^{13}C NMR (101 MHz, $CDCl_3$) δ 201.2, 140.0, 115.4, 98.9, 72.3, 64.9, 50.1, 43.5, 34.5, 30.2, 19.8, 15.8

HRMS (FAB+) calculated for $C_{12}H_{21}O_3$ $[M+H]^+$: 213.1491; found 213.1487

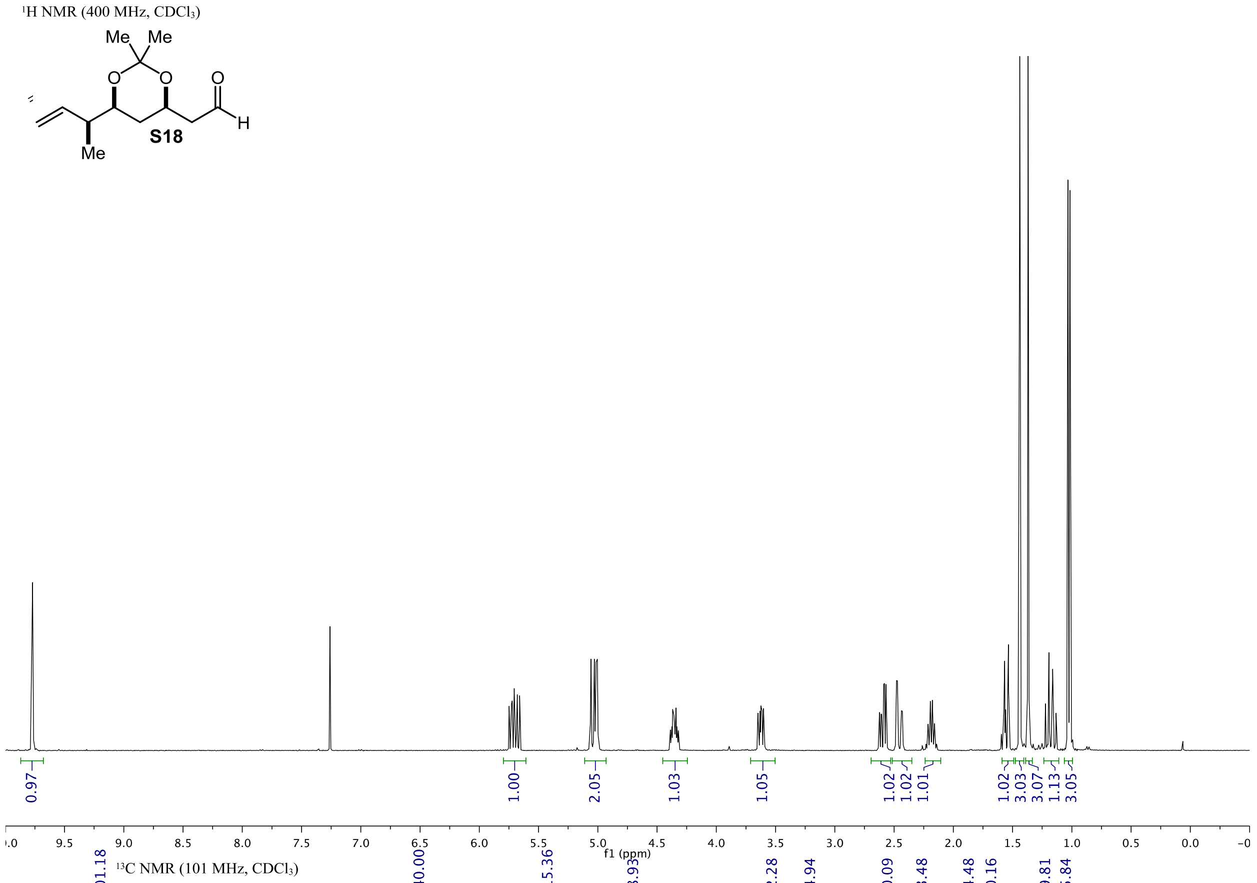

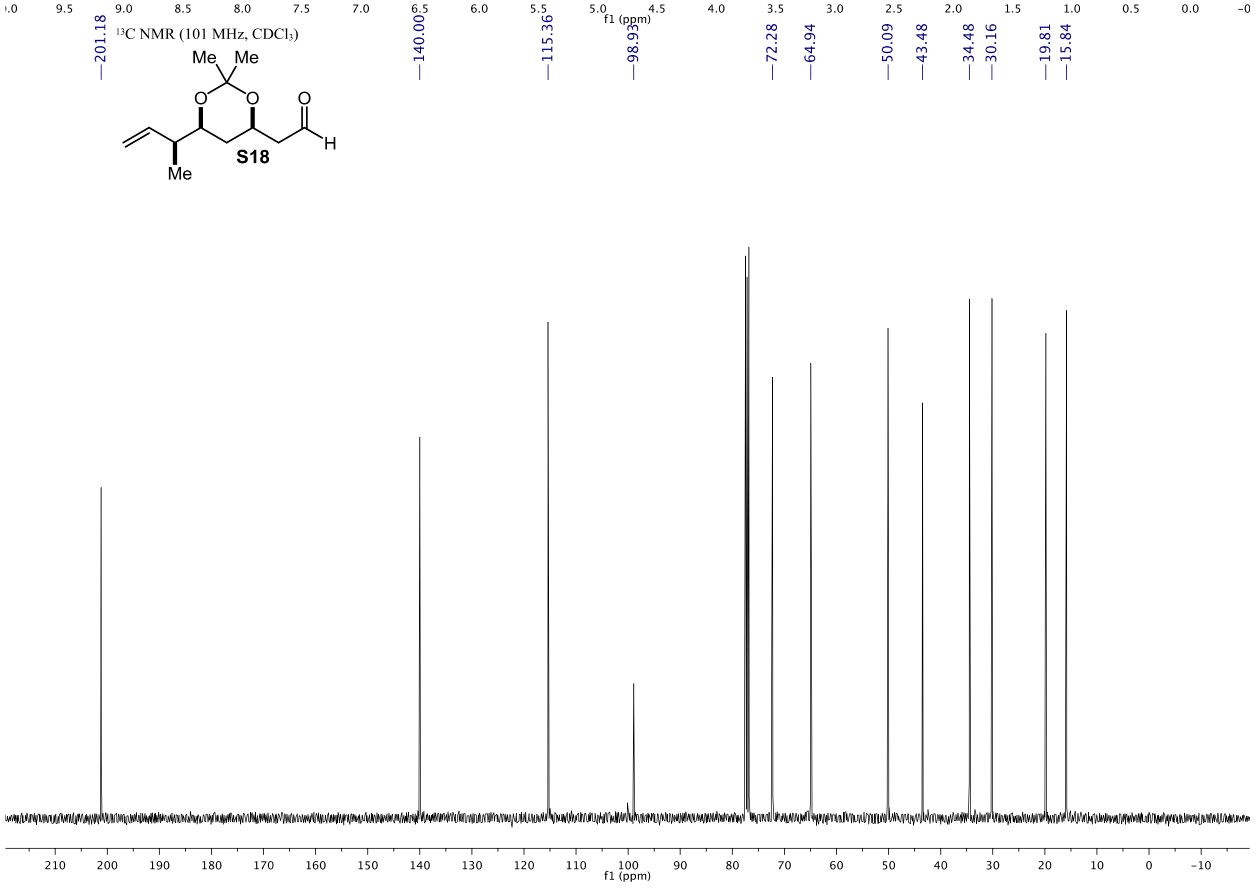

Supplementary Figure 55. ^{1}H NMR and ^{13}C NMR of **S18**.

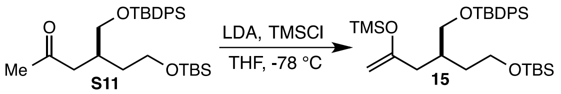

Supplementary Figure 56. Synthesis of **15**.

To a cooled (-78 °C) solution of *i*-Pr_2NH (4.5 mL, 32.1 mmol) in THF (170 mL) was added *n*-BuLi (11.1 mL, 27.8 mmol, 2.5 M in hexanes). After 5 min the solution was warmed to 0 °C, held at that temperature for 10 min, and then re-cooled to -78 °C. A solution of **S11** (10.7 g, 21.4 mmol) in THF (25 mL) was added slowly, with a THF rinse (15 mL). After 20 min TMSCl (4.6 mL, 36.4 mmol) was added slowly. After 1 h the reaction mixture was warmed to room temperature and concentrated to give a thick cloudy oil. [***CAUTION***: over concentration will result in isomerization of the enol ether product]. The residue was resuspended in pentane and the iPr_2NH•HCl salts were removed by filtration. The filtrate was concentrated to give the enol ether as a pale yellow oil that was used immediately in the next step without further purification.

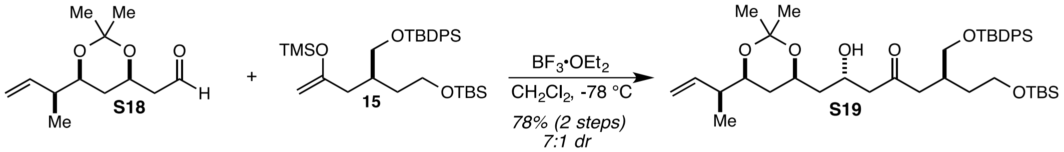

Supplementary Figure 57. Synthesis of **S19**.

To a cooled (-78 °C) solution of the enol ether **15** and unpurifed aldehyde **S18** (4.13 g, 19.5 mmol) in CH_2Cl_2 (200 mL) was added BF_3•Et_2O (2.52 mL, 20.4 mmol) over 20 min by syringe pump. After 1 h the reaction was quenched by the addition of saturated aqueous $NaHCO_3$ (100 mL). The layers were separated and the aqueous layer was extracted with CH_2Cl_2 (3 x 50 mL). The combined organic layers were dried over Na_2SO_4, filtered, and concentrated. Analysis of the residue by ^{1}H NMR spectroscopy revealed a 7:1 dr for the reaction. The residue was purified by silica gel column chromatography eluting with 5:95 → 20:80 EtOAc:Hexanes to give **S19** as a colorless oil (10.8 g, 15.2 mmol, 78% yield from **S17** over 2 steps, 7:1 dr).

OR $[\alpha]_D^{19}$ -4.7° (*c* 1.0, $CHCl_3$)

IR (thin film, cm^{-1}) 2952, 2930, 2857, 1706, 1470, 1427, 1380, 1254, 1200, 1103, 999, 917, 834, 776, 703, 505

^{1}H NMR (400 MHz, $CDCl_3$) δ 7.71 – 7.54 (m, 4H), 7.48 – 7.29 (m, 6H), 5.72 (ddd, *J* = 17.7, 10.4, 7.7 Hz, 1H), 5.13 – 4.94 (m, 2H), 4.35 – 4.19 (m, 1H), 4.16 – 4.00 (m, 1H), 3.73 – 3.42 (m, 5H), 3.32 (d, *J* = 3.5 Hz, 1H), 2.66 (dd, *J* = 16.8, 6.8 Hz, 1H), 2.58 – 2.45 (m, 2H), 2.40 (dd, *J* = 16.8, 6.3 Hz, 1H), 2.34 – 2.23 (m, 1H), 2.18 (hept, *J* = 6.8, 6.4 Hz, 1H), 1.68 – 1.44 (m, 5H), 1.43 (s, 3H), 1.38 (s, 3H), 1.27 – 1.13 (m, 1H), 1.05 (s, 9H), 1.03 (d, *J* = 6.7 Hz, 3H), 0.85 (s, 9H), -0.01 (d, *J* = 1.6 Hz, 6H)

^{13}C NMR (101 MHz, $CDCl_3$) δ 211.4, 140.3, 135.7, 135.7, 133.7, 129.8, 127.8, 115.1, 98.8, 72.6, 66.4, 66.0, 64.5, 61.2, 49.9, 45.7, 43.5, 42.7, 34.7, 34.4, 33.7, 30.4, 27.1, 26.1, 19.9, 19.45, 18.4, 15.9, -5.2

HRMS (FAB+): calculated for $C_{41}H_{66}O_6Si_2Na$ $[M+Na]^+$ 733.4296; found 733.4267

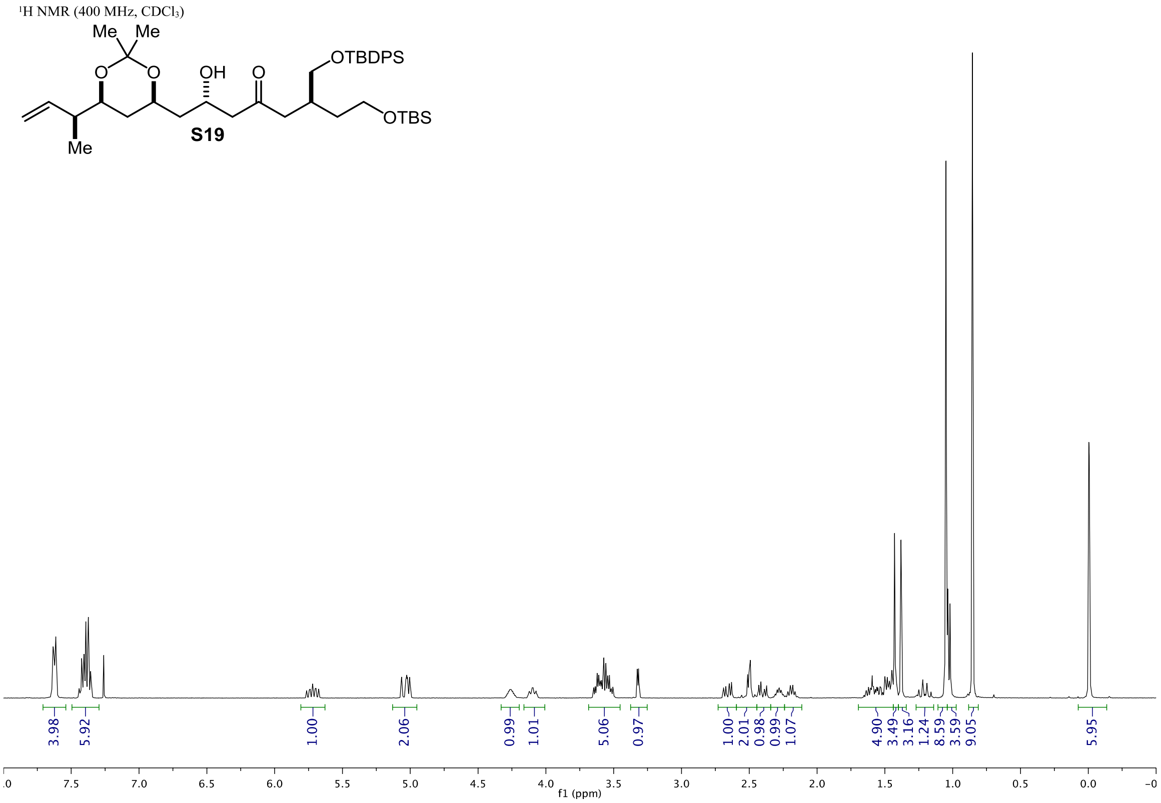

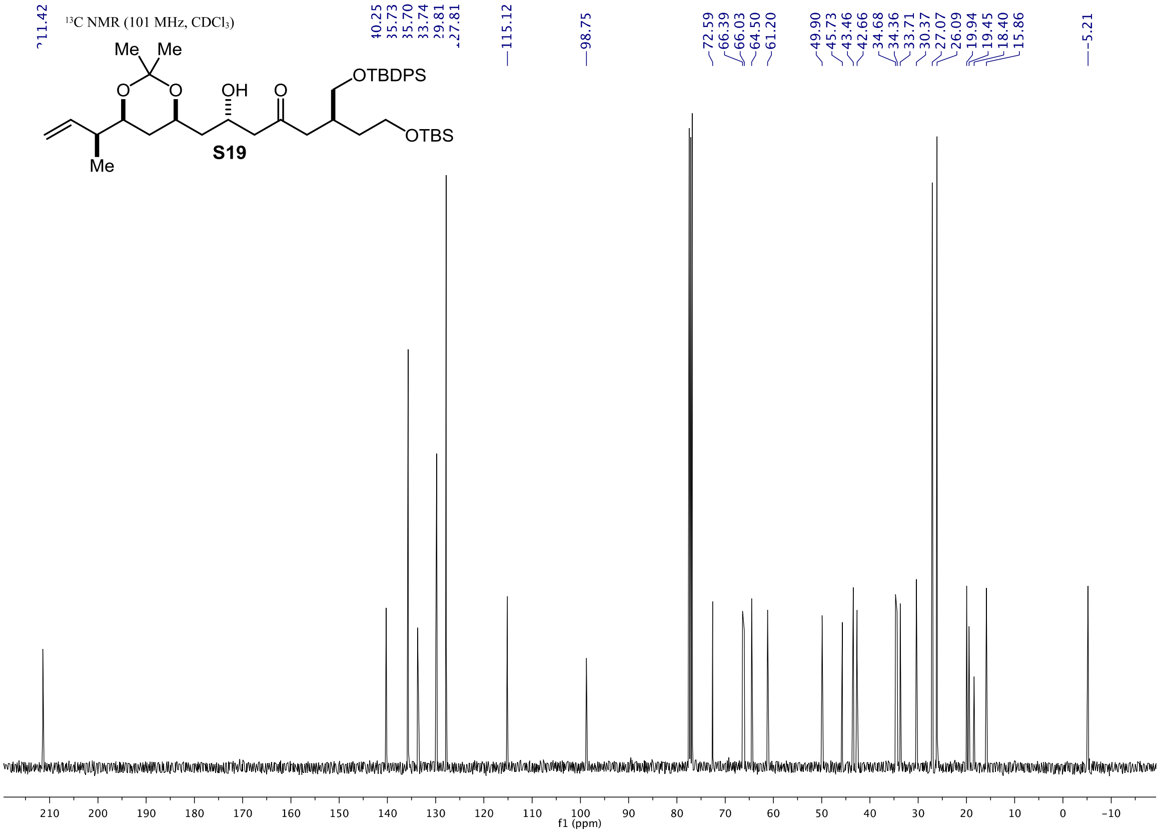

Supplementary Figure 58. ^{1}H NMR and ^{13}C NMR of **S19**.

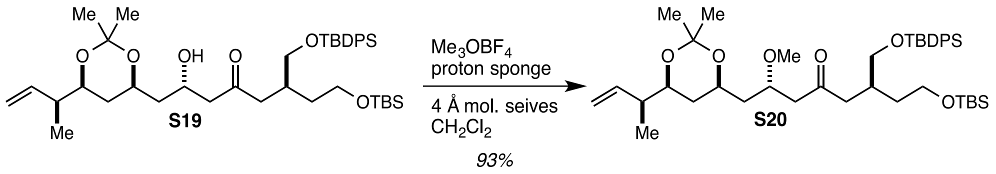

Supplementary Figure 59. Synthesis of **S20**.

To a solution of **S19** (10.8 g, 15.2 mmol) in CH_2Cl_2 (150 mL) was added 4 Å MS (11.0 g), proton sponge (32.6 g, 152 mmol) and Me_3O•BF_4 (18.1 g, 122 mmol). After 5 h the mixture was filtered through celite, with EtOAc rinses. The filtrate was washed with 1 M AcOH (1 x 500 mL) and saturated aqueous $NaHCO_3$ (1 x 300 mL), dried over $MgSO_4$, filtered, and concentrated. The residue was purified by silica gel column chromatography eluting with 5:95 → 20:80 EtOAc:Hexanes to give **S20** (7:1 dr) as a colorless oil (10.20 g, 14.1 mmol, 93% yield).

OR $[\alpha]_D^{19}$ -8.4° (*c* 1.0, $CHCl_3$)

IR (thin film, cm^{-1}) 2930, 2855, 1713, 1470, 1378, 1253, 1199, 1090, 835, 703, 505

^{1}H NMR (400 MHz, $CDCl_3$) δ 7.71 – 7.52 (m, 4H), 7.49 – 7.31 (m, 6H), 5.72 (ddd, *J* = 17.3, 10.4, 7.7 Hz, 1H), 5.12 – 4.93 (m, 2H), 4.02 – 3.91 (m, 1H), 3.91 – 3.81 (m, 1H), 3.68 – 3.47 (m, 5H), 3.32 (s, 3H), 2.64 (ddd, *J* = 24.3, 16.4, 7.0 Hz, 2H), 2.49 – 2.35 (m, 2H), 2.28 (h, *J* = 6.1 Hz, 1H), 2.18 (h, *J* = 7.0 Hz, 1H), 1.68 – 1.45 (m, 4H), 1.45 – 1.39 (m, 1H), 1.41 (s, 3H), 1.37 (s, 3H), 1.17 – 1.07 (m, 1H), 1.06 (s, 9H), 1.02 (d, *J* = 6.7 Hz, 3H), 0.85 (s, 9H), -0.01 (d, *J* = 1.7 Hz, 6H)

^{13}C NMR (101 MHz, $CDCl_3$) δ 209.0, 140.3, 135.7, 135.7, 133.8, 129.8, 127.8, 115.0, 98.6, 74.0, 72.6, 66.1, 65.7, 61.3, 58.2, 48.8, 46.1, 43.5, 42.6, 35.1, 34.5, 33.7, 30.4, 27.1, 26.1, 20.0, 19.5, 18.4, 15.8, -5.2

HRMS (FAB+) calculated for $C_{42}H_{68}O_6Si_2Na$ $[M+Na]^+$: 747.4452; found 747.4445

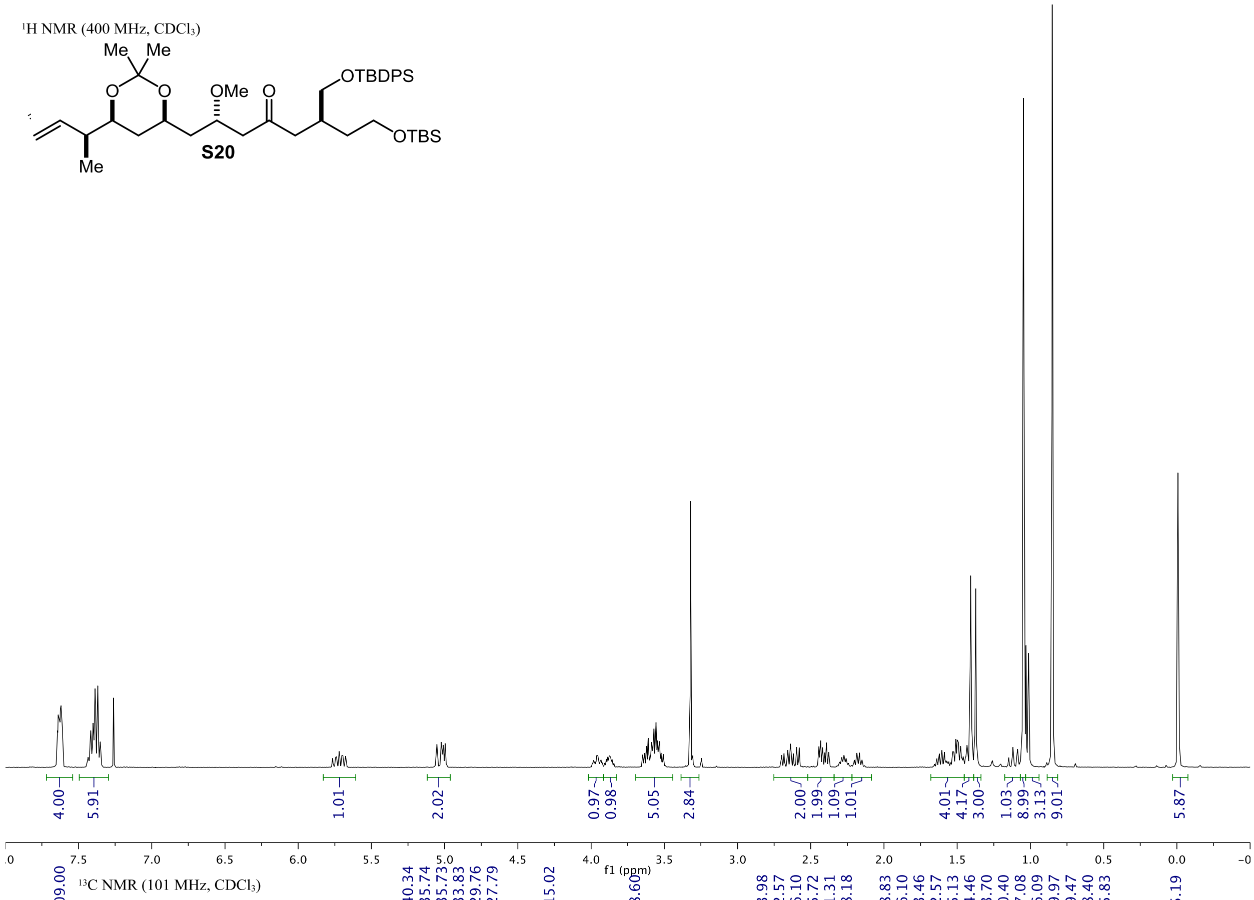

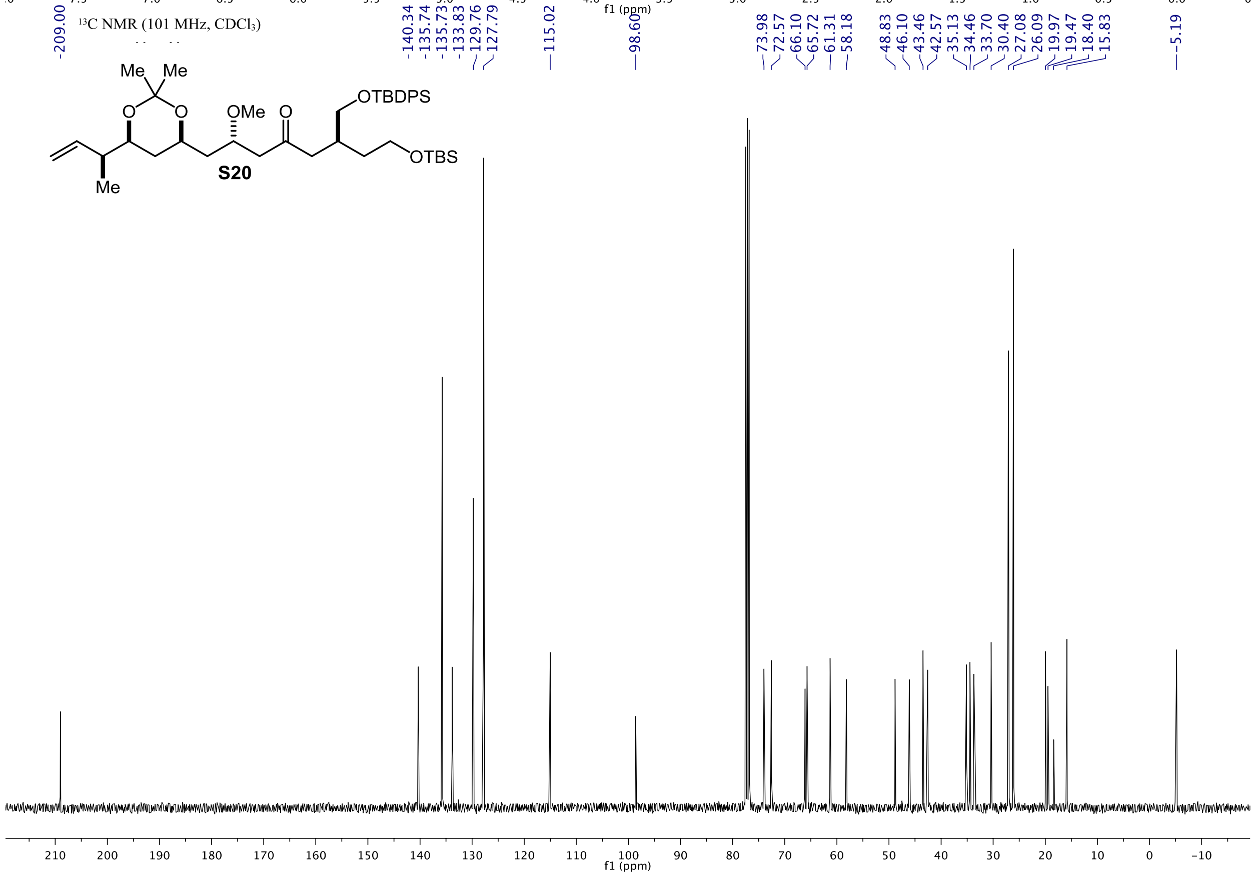

Supplementary Figure 60. ^{1}H NMR and ^{13}C NMR of **S20**.

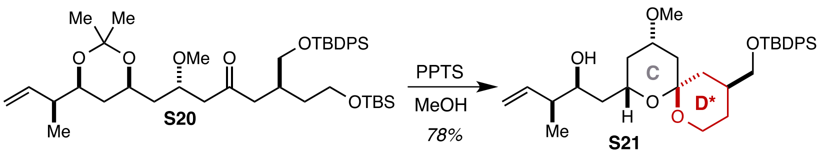

Supplementary Figure 61. Synthesis of **S21**.

To a solution of ketone **S20** (70 mg, 0.1 mmol) in MeOH (1.0 mL) was added pyridinium *p*-toluene sulfonate (PPTS) (25 mg, 0.1 mmol). After 20 h additional PPTS (25 mg, 0.1 mmol) was added. After 2 h the reaction mixture was quenched by the addition of saturated aqueous $NaHCO_3$ (3.0 mL). The mixture was extracted with CH_2Cl_2 (3 x 2.0 mL). The combined organic layers were dried over $MgSO_4$, filtered, and concentrated. The residue was purified by silica gel column chromatography eluting with 5:95 → 20:80 EtOAc:Hexanes. The product **S21** was isolated as a colorless oil and was diastereomerically pure (42 mg, 0.075 mmol, 78% yield).

OR $[\alpha]_D^{22}$ +32.4° (*c* 1.8, $CHCl_3$)

IR (thin film, cm^{-1}) 3501, 3061, 2926, 2858, 1636, 1420, 1380, 1106, 710

^{1}H NMR (500 MHz, $CDCl_3$) δ 7.74 – 7.57 (m, 4H), 7.48 – 7.29 (m, 6H), 5.84 (ddd, *J* = 17.6, 10.4, 7.6 Hz, 1H), 5.12 – 5.02 (m, 2H), 3.92 – 3.80 (m, 1H), 3.76 – 3.59 (m, 5H), 3.48 (dd, *J* = 10.0, 5.2 Hz, 1H), 3.42 (dd, *J* = 10.0, 5.7 Hz, 1H), 3.33 (s, 3H), 2.28 (h, *J* = 6.9 Hz, 1H), 2.14 – 2.07 (m, 1H), 2.07 – 1.96 (m, 2H), 1.74 – 1.55 (m, 4H), 1.45 – 1.35 (m, 1H), 1.35 – 1.22 (m, 2H), 1.18 (q, *J* = 11.7 Hz, 1H), 1.08 (d, *J* = 6.8 Hz, 3H), 1.05 (s, 9H)

^{13}C NMR (126 MHz, $CDCl_3$) δ 141.2, 135.8, 133.8, 129.7, 127.8, 115.0, 98.3, 75.5, 72.7, 70.3, 68.3, 60.4, 55.6, 44.1, 41.7, 39.6, 38.2, 38.0, 33.0, 28.1, 27.1, 19.5, 15.3

HRMS (FAB+) calculated for $C_{33}H_{48}O_5SiNa$ $[M+Na]^+$: 575.3169; found 575.3161

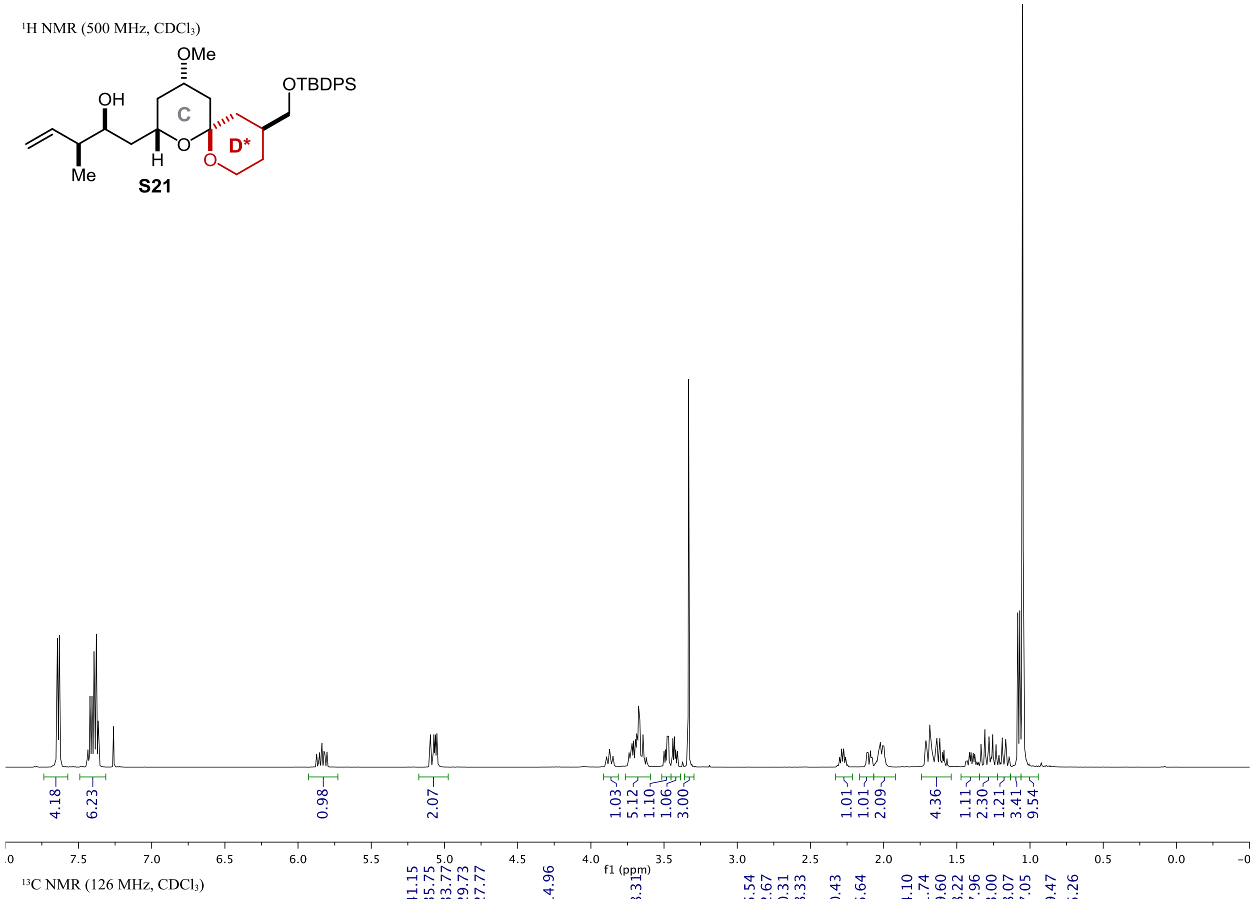

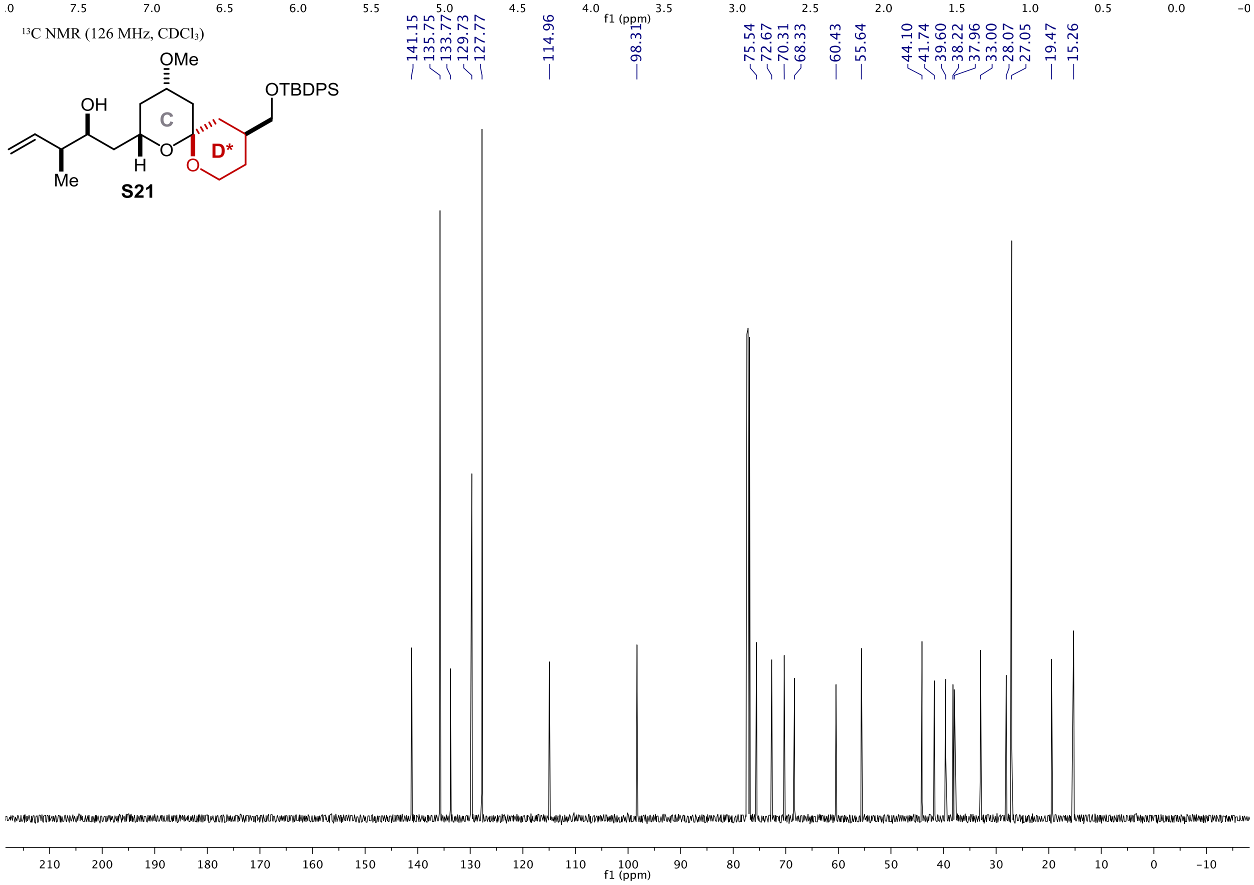

Supplementary Figure 62. ^{1}H NMR and ^{13}C NMR of **S21**.

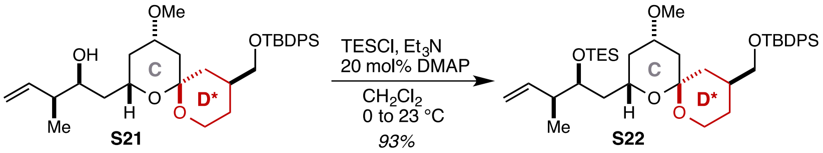

Supplementary Figure 63, Synthesis of **S22**.

To a cooled (0 °C) solution of **S21** (4.79 g, 8.7 mmol) in CH_2Cl_2 (90 mL) was sequentially added Et_3N (2.66 mL, 19.1 mmol), TESCl (2.91 mL, 17.3 mmol), and DMAP (0.21 g, 1.7 mmol). The resulting mixture was warmed to room temperature and stirred 15 h. Excess TESCl was quenched by the addition of MeOH (0.80 mL) and the reaction mixture was concentrated. The residue was resuspended in hexanes and the Et_3N•HCl salts were removed by filtration. The filtrate was concentrated and the residue purified by silica gel column chromatography eluting with 5:95 EtOAc:Hexanes to give **S22** as a colorless oil (5.38 g, 8.1 mmol, 93% yield).

OR $[\alpha]_D^{18}$ +21.5° (*c* 1.0, $CHCl_3$)

IR (thin film, cm^{-1}) 3071, 2953, 2932, 2876, 1639, 1461, 1385, 1239, 1092, 1004, 824, 739, 703, 614, 505

^{1}H NMR (400 MHz, $CDCl_3$) δ 7.72 – 7.56 (m, 4H), 7.51 – 7.28 (m, 6H), 6.02 – 5.80 (m, 1H), 5.13 – 4.92 (m, 2H), 3.88 – 3.71 (m, 2H), 3.70 – 3.58 (m, 3H), 3.50 (dd, *J* = 9.9, 5.3 Hz, 1H), 3.42 (dd, *J* = 9.8, 6.3 Hz, 1H), 3.35 (s, 3H), 2.50 – 2.35 (m, 1H), 2.28 – 2.14 (m, 1H), 2.13 – 1.99 (m, 2H), 1.81 – 1.53 (m, 4H), 1.34 (qd, *J* = 12.2, 6.3 Hz, 1H), 1.24 (dd, *J* = 12.9, 11.6 Hz, 2H), 1.06 (s, 9H), 1.10 – 1.02 (m, 1H), 1.02 – 0.92 (m, 12H), 0.62 (q, *J* = 7.9 Hz, 6H)

^{13}C NMR (101 MHz, $CDCl_3$) δ 141.5, 135.7, 134.0, 133.9, 129.7, 127.8, 114.4, 97.7, 73.4, 72.8, 68.7, 65.4, 60.3, 55.6, 43.1, 41.9, 40.6, 38.8, 37.5, 33.0, 28.7, 27.0, 19.5, 14.6, 7.1, 5.4

HRMS (ESI+) calculated for $C_{39}H_{62}O_5Si_2Na$ $[M+Na]^+$: 689.4033; found 689.4031

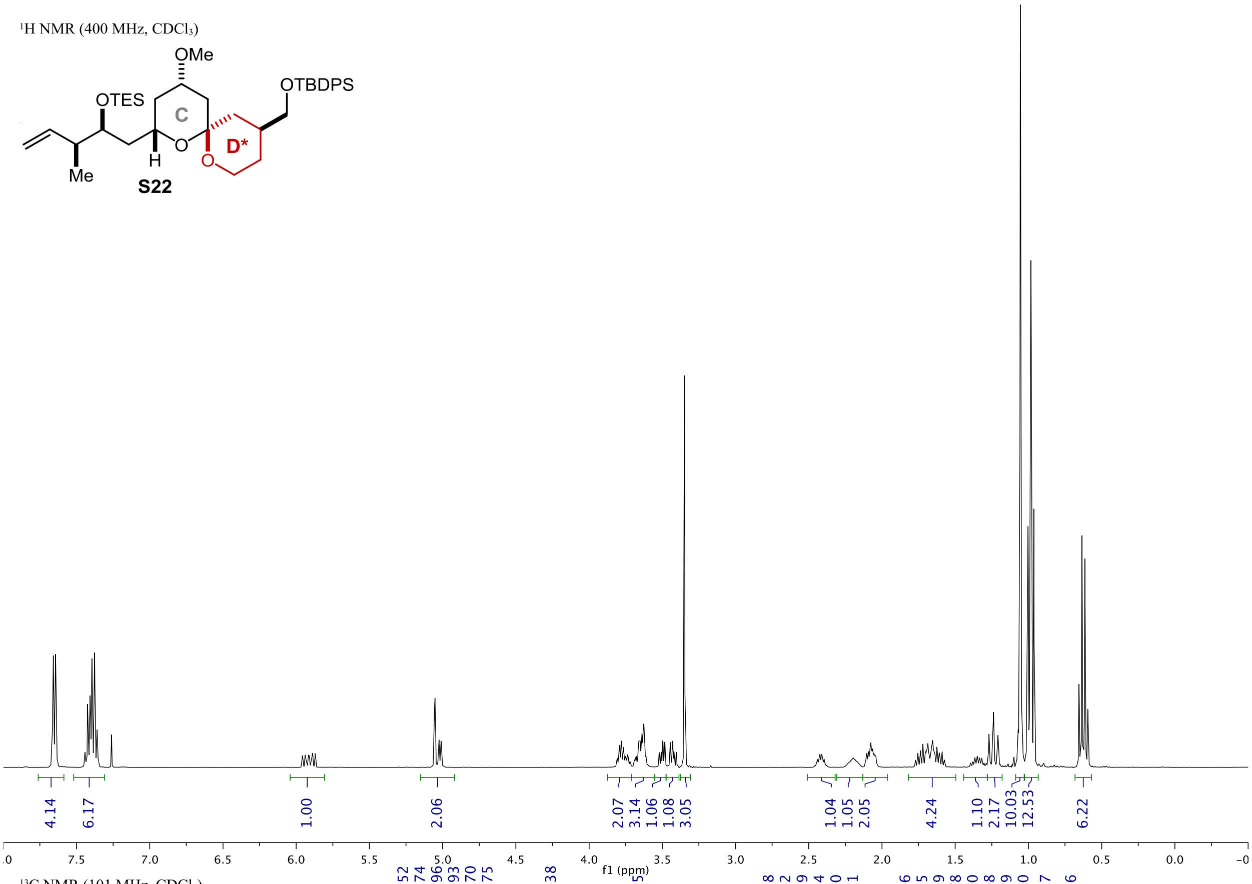

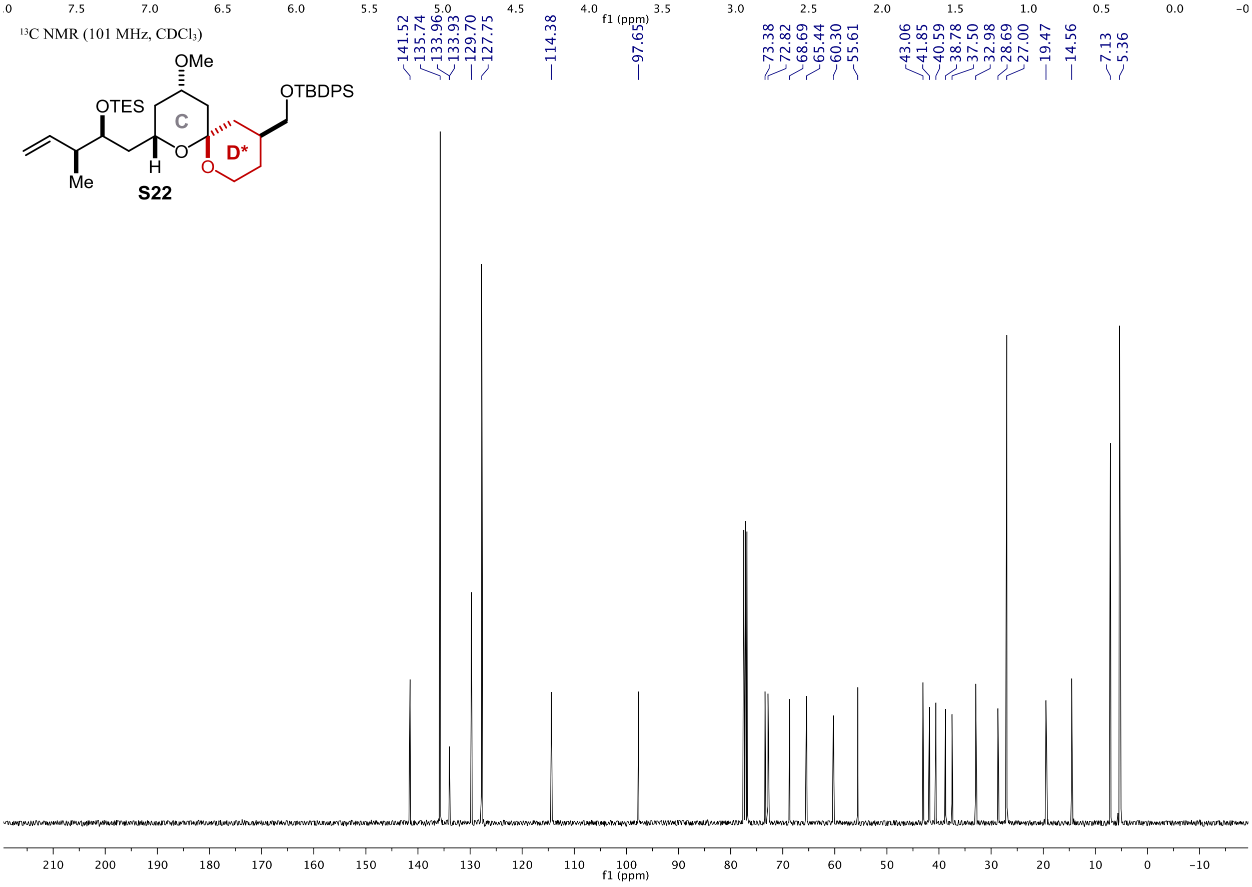

Supplementary Figure 64. ^{1}H NMR and ^{13}C NMR of **S22**.

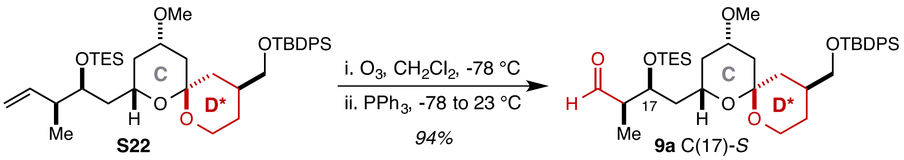

Supplementary Figure 65. Synthesis of **9a** C(17)-*S*.

A cooled (-78 °C) solution of **S22** (1.15 g, 1.7 mmol) in CH_2Cl_2 (17 mL) was sparged with O_2 for 5 min. Ozone was then bubbled through the solution until it turned blue (~8 min.). The solution was sparged with O_2 for 5 min resulting in a cloudy white mixture. PPh_3 (0.54 g, 2.1 mmol) was added and the resulting mixture was warmed to room temperature. After 12h, the mixture was concentrated [**CAUTION!!!! It is imperative to make sure that the ozonides have been completely reduced before concentration**]. The residue was resuspended in hexanes and residual PPh_3O was removed by filtration. The filtrate was concentrated and the residue was purified by silica gel column chromatography eluting with 5:95 → 15:85 EtOAc:Hexanes to give **9a** as a colorless oil (1.07 g, 1.6 mmol, 94% yield).

OR $[\alpha]_D^{16}$ 14.1° (*c* 1.0, $CHCl_3$)

IR (thin film, cm^{-1}) 2930, 2876, 1727, 1589, 1461, 1385, 1240, 1104, 822, 703, 614, 505

^{1}H NMR (400 MHz, $CDCl_3$) δ 9.78 (d, *J* = 1.0 Hz, 1H), 7.75 – 7.56 (m, 4H), 7.51 – 7.30 (m, 6H), 4.52 – 4.40 (m, 1H), 3.72 – 3.52 (m, 4H), 3.52 – 3.39 (m, 2H), 3.34 (s, 3H), 2.61 – 2.51 (m, 1H), 2.25 – 2.12 (m, 1H), 2.09 (ddd, *J* = 12.5, 4.7, 1.7 Hz, 1H), 2.05 – 1.95 (m, 1H), 1.85 – 1.57 (m, 4H), 1.42 – 1.29 (m, 1H), 1.29 – 1.18 (m, 2H), 1.11 (d, *J* = 6.9 Hz, 3H), 1.16 – 1.08 (m, 1H) 1.05 (s, 9H), 0.95 (t, *J* = 7.9 Hz, 9H), 0.67 – 0.53 (m, 6H)

^{13}C NMR (101 MHz, $CDCl_3$) δ 205.1, 135.7, 133.9, 129.7, 127.8, 97.8, 73.1, 68.6, 68.5, 65.2, 60.5, 55.7, 51.2, 41.8, 41.2, 38.9, 37.5, 33.1, 28.6, 27.0, 19.5, 7.6, 7.0, 5.2

HRMS (FAB+) calculated for $C_{38}H_{60}O_6Si_2Na$ $[M+Na]^+$: 691.3826; found 691.3836

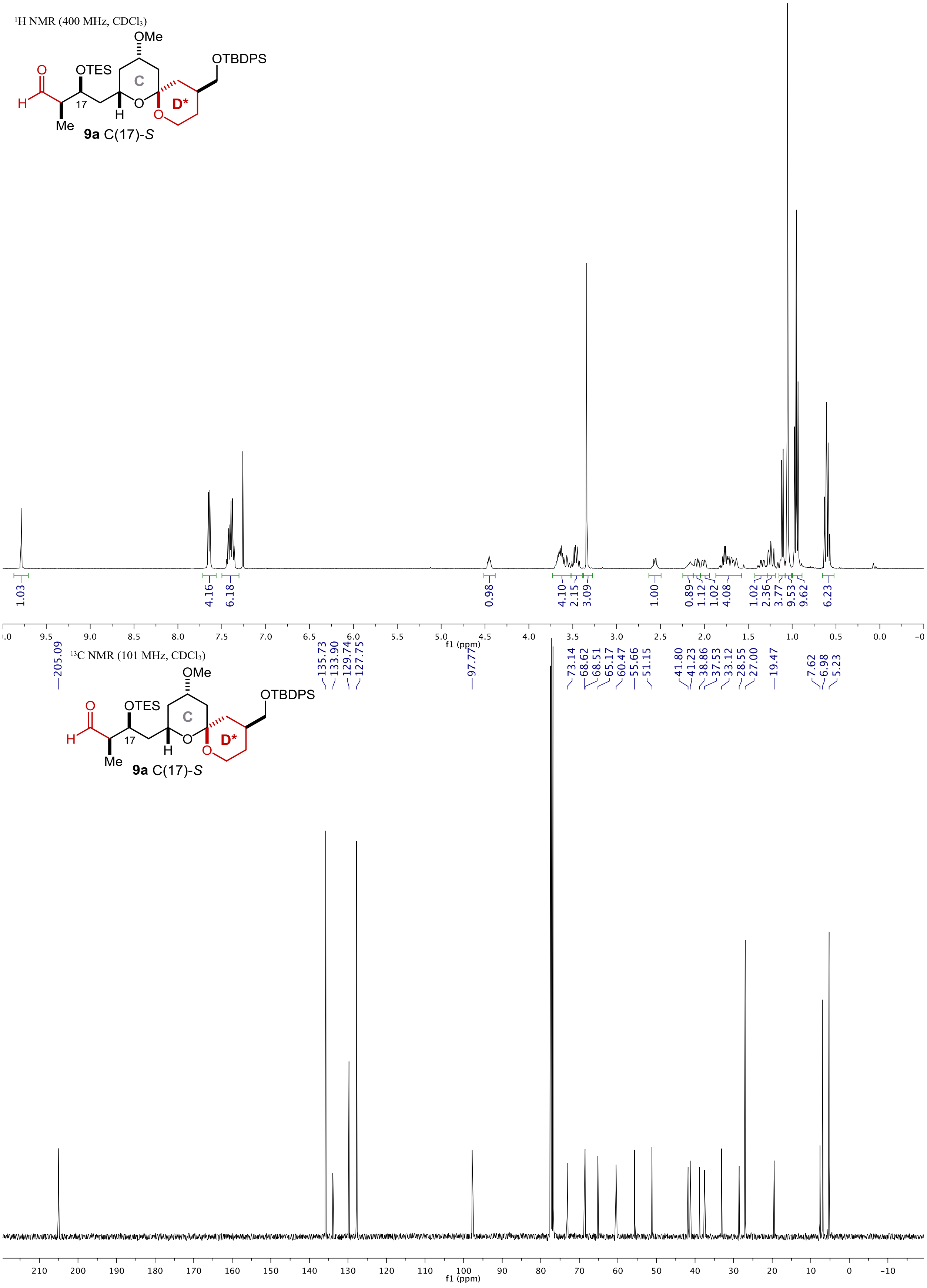

Supplementary Figure 66. ^{1}H NMR and ^{13}C NMR of **9a** C(17)-*S*.

Synthesis of diaminophenol **24**

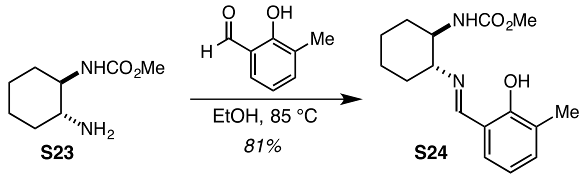

Supplementary Figure 67. Synthesis of **S24**.

To a solution of compound **S23**[6] (4.89 g, 28.4 mmol) in EtOH (82 mL), was added 2-hydroxy-3-methylbenzaldehyde (4.13 mL, 34.1 mmol). After 12 h the mixture was concentrated and the residue was recrystallized from minimal boiling EtOH to give imine **S24** as yellow crystals (6.70 g, 23.0 mmol, 81% yield).

OR $[\alpha]_D^{23}$ -79.5° (*c* 1.0, $CHCl_3$)

IR (thin film, cm^{-1}) 3312, 2932, 2857, 1695, 1626, 1534, 1447, 1361, 1313, 1261, 1194, 1142, 1031, 845, 748

^{1}H NMR (500 MHz, $CDCl_3$) δ 13.42 (s, 1H), 8.32 (s, 1H), 7.17 (ddd, *J* = 7.4, 1.8, 0.9 Hz, 1H), 7.08 (dd, *J* = 7.6, 1.6 Hz, 1H), 6.78 (t, *J* = 7.5 Hz, 1H), 4.51 (s, 1H), 3.62 (dtd, *J* = 10.6, 9.0, 4.1 Hz, 1H), 3.56 (s, 3H), 3.11 (s, 1H), 2.27 (s, 3H), 2.12 (d, *J* = 12.2 Hz, 1H), 1.90 – 1.72 (m, 3H), 1.71 – 1.61 (m, 1H), 1.40 (dt, *J* = 31.6, 12.1 Hz, 3H)

^{13}C NMR (126 MHz, $CDCl_3$) δ 164.4, 159.4, 133.3, 129.0, 126.0, 118.0, 117.9, 54.9, 52.0, 33.4, 24.5, 23.9, 15.5

HRMS (ESI+) calculated for $C_{16}H_{23}N_2O_3$ $[M+H]^+$: 291.1709; found 291.1705

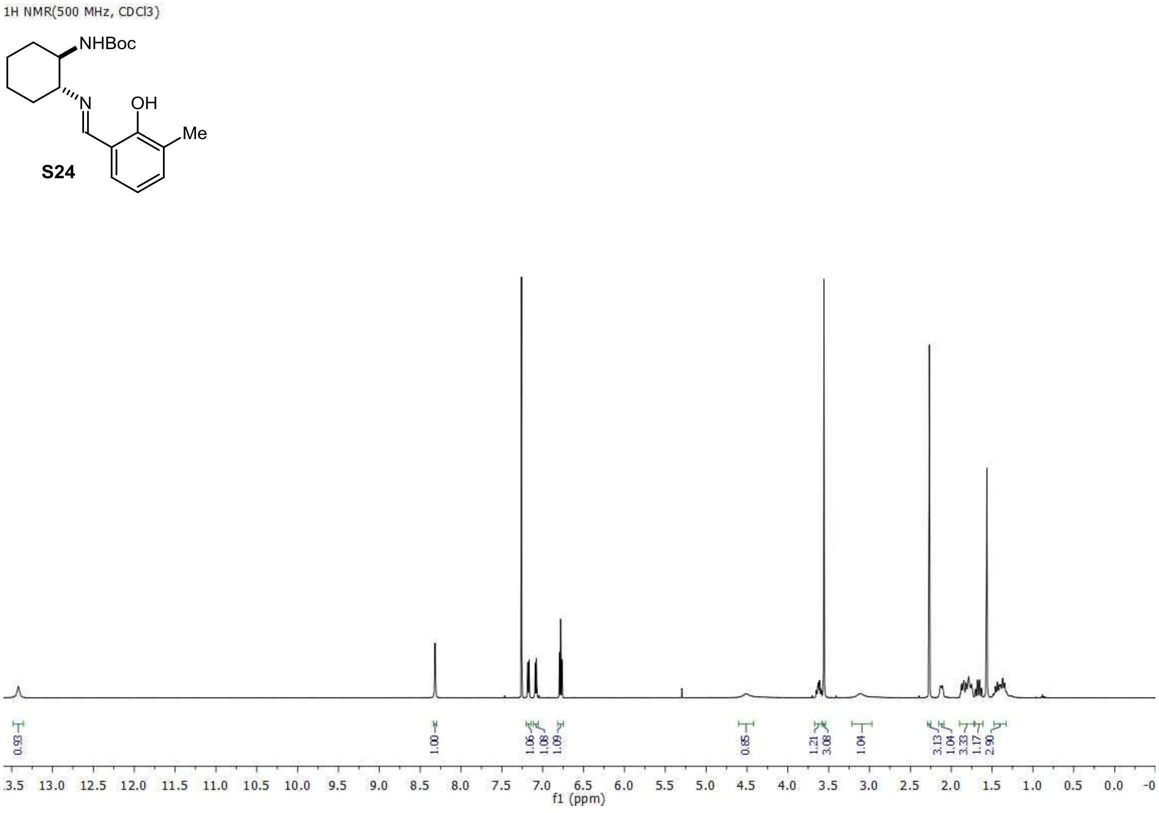

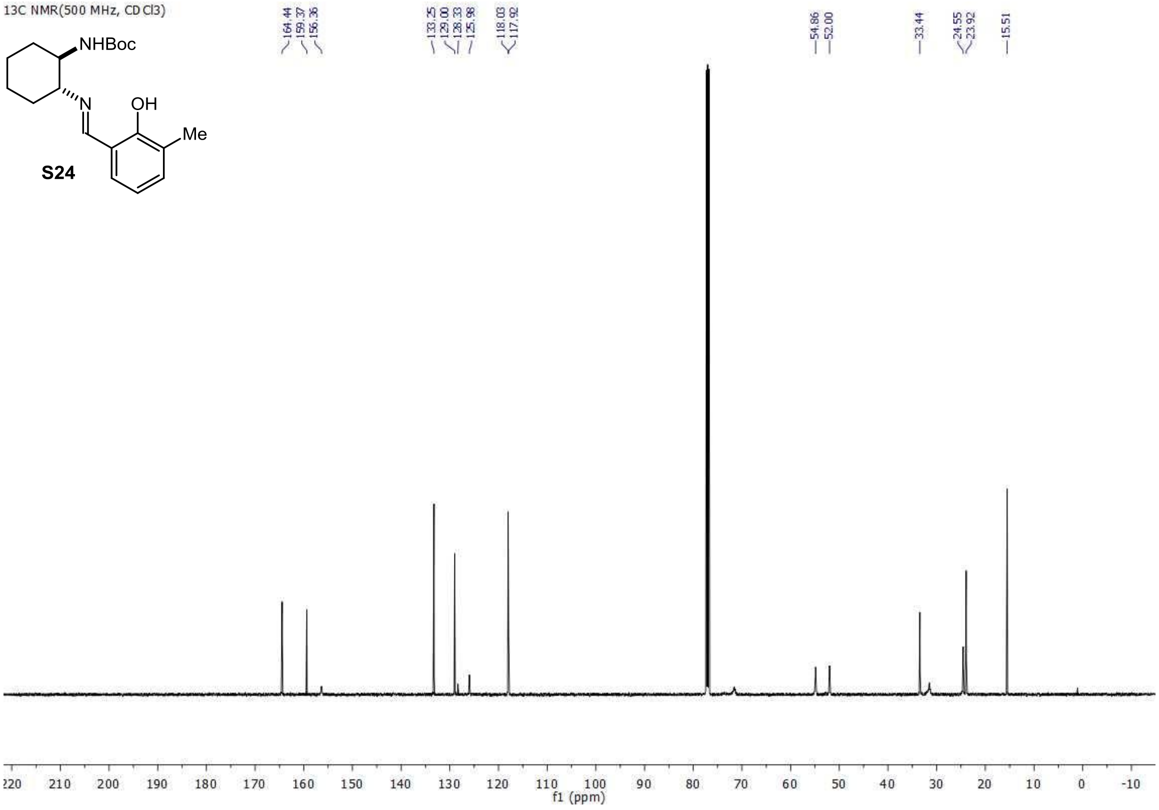

Supplementary Figure 68. ^{1}H NMR and ^{13}C NMR of **S24**.

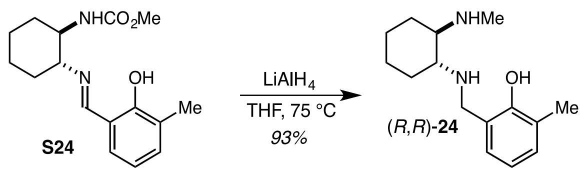

Supplementary Figure 69. Synthesis of (*R*,*R*)-**24**.

A 500 mL roundbottom flask equipped with an addition funnel was charged with $LiAlH_4$ (4.10 g, 108 mmol) and THF (110 mL) and the resulting mixture was cooled to 0 °C. A solution of **S24** (6.70 g, 27.0 mmol) in THF (110 mL) was added slowly *via* the addition funnel, with THF rinses (3 x 20.0 mL). The mixture was warmed to room temperature and stirred for 2 h. The addition funnel was replaced with a reflux condenser and the mixture was heated at reflux for 12 h. The mixture was cooled to 0 °C, and the reaction was quenched by the ***slow*** sequential addition of water (4.1 mL), 15% aq. NaOH (4.1 mL) and water (12.3 mL). The resulting mixture was stirred for 10 min at room temperature. The mixture was dried by stirring with excess $MgSO_4$ for an additional 20 min. The fine white solids were removed filtration and the filtrate was concentrated. The resulting beige solid was purified by recrystallization from minimal boiling hexanes to give diaminophenol (*R*,*R*)-**24** as colorless crystals (6.24 g, 25.1 mmol, 93% yield).

OR $[\alpha]_D^{24}$ -137.3° (*c* 1.0, $CHCl_3$)

IR (thin film, cm^{-1}) 3312, 3235, 2924, 2851, 2796, 1595, 1467, 1449, 1359, 1268, 1230, 1111, 1082, 975, 854, 766, 742

^{1}H NMR (400 MHz, $CDCl_3$) δ 7.08 – 6.96 (m, 1H), 6.84 (ddd, *J* = 7.5, 1.7, 0.9 Hz, 1H), 6.68 (t, *J* = 7.4 Hz, 1H), 4.02 (d, *J* = 13.6 Hz, 1H), 3.82 (d, *J* = 13.6 Hz, 1H), 2.40 (s, 3H), 2.24 (s, 3H), 2.22 – 2.09 (m, 4H), 1.82 – 1.65 (m, 2H), 1.31 – 1.13 (m, 3H), 1.02 – 0.88 (m, 1H)

^{13}C NMR (101 MHz, $CDCl_3$) δ 156.5, 129.7, 125.6, 125.3, 123.3, 118.4, 62.5, 62.2, 50.5, 33.5, 31.2, 31.1, 25.3, 24.8, 15.9

HRMS (ESI+) calculated for $C_{15}H_{25}N_2O$ $[M+H]^+$: 249.1967; found 249.1967.

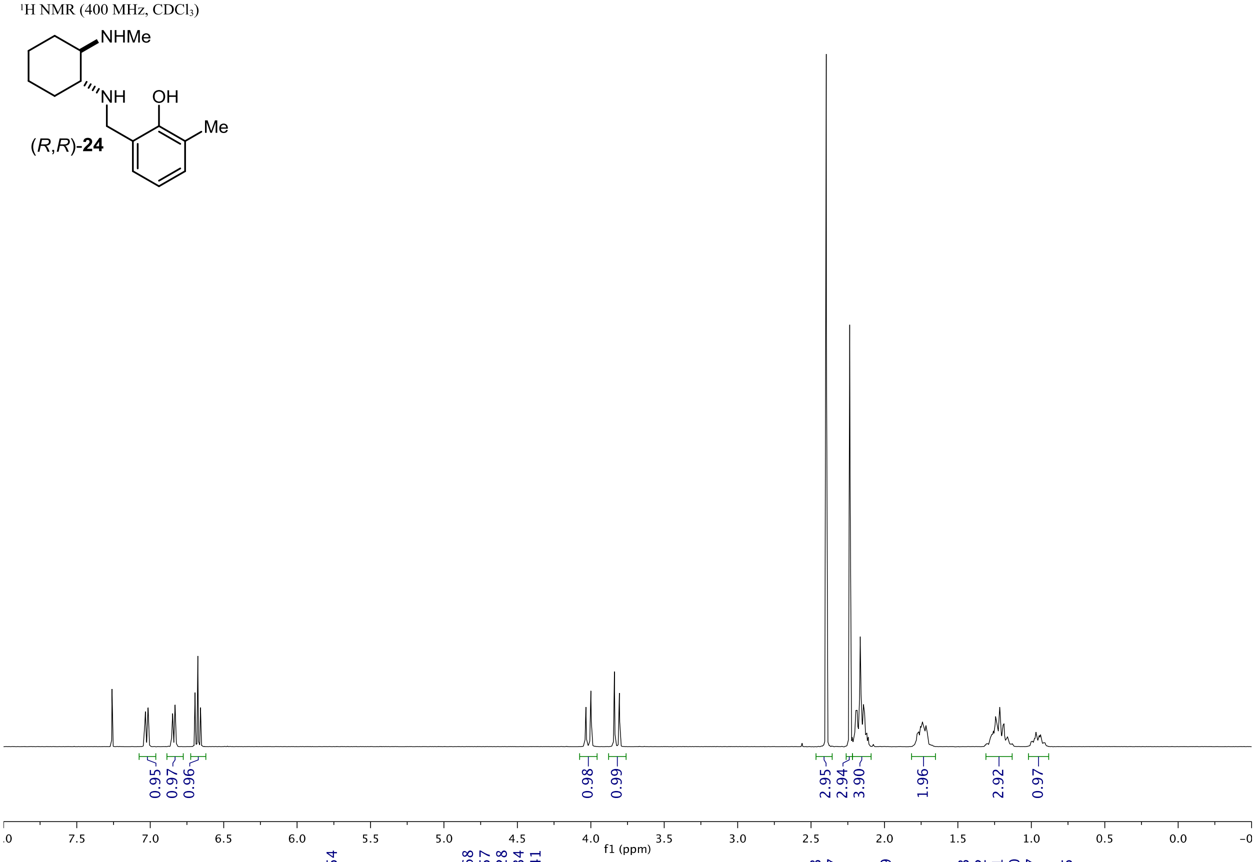

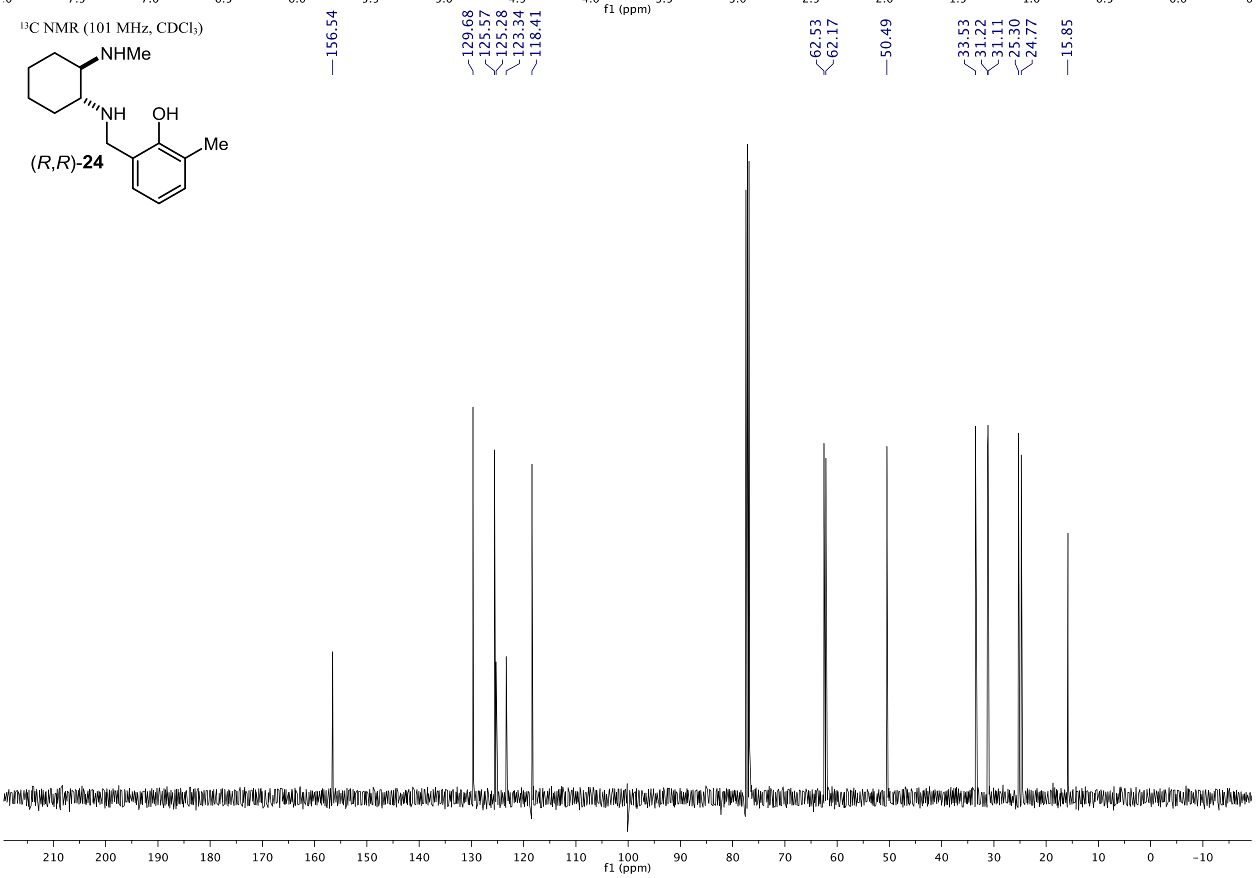

Supplementary Figure 70. ^{1}H NMR and ^{13}C NMR of (*R*,*R*)-**24**.

Synthesis of **21** from **12** and **9a**

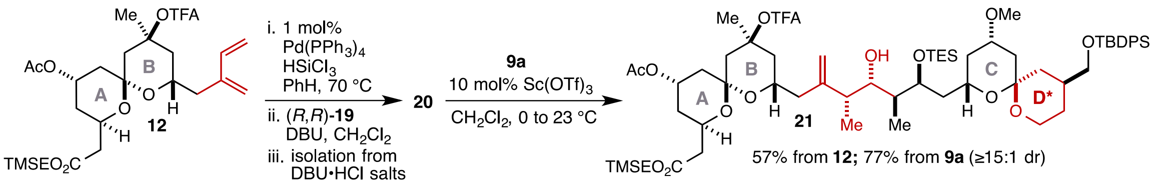

Supplementary Figure 71. Synthesis of **21**.

A 100 mL-capacity, septum-adapted pressure tube was charged with $Pd(PPh_3)_4$ (24 mg, 0.02 mmol), evacuated, and then back-filled with N_2. A solution of diene **12**[10] (1.19 g, 2.1 mmol) in benzene (24 mL) was added followed by trichlorosilane (0.43 mL, 4.2 mmol) and the tube was tightly sealed. The tube was heated with an oil bath set at 70 °C. After 15 h the oil bath was removed and the sealed tube was allowed to cool to room temperature. The resulting light brown solution was transferred by cannula into a 250 mL round bottom flask, rinsing with benzene (3 x 5 mL). The flask was attached to a vacuum line equipped with a manometer and a -78 °C cold finger, and then placed in a warm (~35 °C) water bath. All volatiles were evaporated by application of vacuum until a viscous oil remained.

The residue was dissolved in CH_2Cl_2 (21.0 mL) and (*R*,*R*)-**19**[11] (1.11 g, 2.11 mmol) was added. The resulting white slurry was cooled to 0 °C and DBU (1.26 mL, 8.4 mmol) was added over 10 min. After 5 min, the mixture was warmed to room temperature. After 2 h, the flask was attached to a vacuum line equipped with a manometer and a -78 °C coldfinger, and then placed in a room temperature water bath. All volatiles were removed by careful application of vacuum, and the residue was treated with Et_2O (25.0 mL). The mixture was stirred vigorously for 3 h during which time to precipitate the DBU•HCl. The DBU•HCl salts were removed by syringe transfer on to an oven-dried, air-free filter frit and filtering into a 250 mL round bottom flask. The residual DBU•HCl salts were rinsed with Et_2O (3 x 5.0 mL) and the resulting supernatants were again filtered through the air-free frit into the flask. The flask was attached to a vacuum line equipped with a manometer and a -78 °C coldfinger, and then placed in a room temperature water bath. All volatiles were removed by careful application of vacuum until the resulting yellow oil became opaque and foamy. The residue was dissolved in CH_2Cl_2 (11.0 mL) and used without further purification.

To the above solution (cooled to 0 °C) was added aldehyde **9a** (1.06 g, 1.58 mmol) as a solution in CH_2Cl_2 (5.0 mL) rinsing with CH_2Cl_2 (2 x 2.5 mL). $Sc(OTf)_3$ (78 mg, 0.16 mmol) was added in one portion. The N_2 inlet was removed and the septum was parafilmed. The reaction solution was stirred for 15 h during which time the ice/water bath was allowed to warm to room temperature. The reaction mixture was re-cooled to 0 ºC and TBAF•$3H_2O$ (0.50 g, 1.6 mmol) was added. After 45 min the mixture was concentrated. Analysis of the residue by ^1H NMR spectroscopy revealed that the product was formed with > 15:1 diastereoselectivity. The residue was purified by silica gel column chromatography eluting with 5:95 → 100:0 EtOAc:Hexanes to give pure **21** as a beige foam (1.50 g, 1.21 mmol, 57% from **12**, 77% from **9a**).

OR $[\alpha]_D^{19}$ -13.5° (*c* 1.0, $CHCl_3$)

IR (thin film, cm^{-1}) 2953, 2932, 1778, 1736, 1379, 1247, 1216, 1165, 1107, 1000, 859, 741, 704

^{1}H NMR (400 MHz, $CDCl_3$) δ 7.74 – 7.56 (m, 4H), 7.51 – 7.31 (m, 6H), 5.17 (s, 1H), 5.08 – 4.98 (m, 2H), 4.38 – 4.27 (m, 2H), 4.27 – 4.20 (m, 1H), 4.20 – 4.06 (m, 2H), 3.78 – 3.54 (m, 5H), 3.49 (dd, J = 9.8, 5.3 Hz, 1H), 3.42 (dd, J = 9.8, 6.2 Hz, 1H), 3.34 (s, 3H), 2.99 – 2.78 (m, 2H), 2.56 – 2.32 (m, 3H), 2.32 – 2.16 (m, 3H), 2.13 – 1.99 (m, 2H), 2.03 (s, 3H), 1.99 – 1.74 (m, 4H), 1.74 –

1.56 (m, 4H), 1.56 – 1.43 (m, 1H), 1.53 (s, 3H), 1.43 – 1.17 (m, 6H), 1.05 (s, 9H), 1.08-1.01 (m, 3H), 0.98 (t, *J* = 7.8 Hz, 11H), 0.81 (d, *J* = 6.9 Hz, 3H), 0.63 (q, *J* = 7.9 Hz, 6H), 0.04 (s, 10H)

^{13}C NMR (101 MHz, $CDCl_3$) δ 171.1, 170.6, 156.6, 156.2, 148.1, 135.8, 134.0, 133.9, 129.7, 127.8, 115.9, 113.7, 113.0, 97.7, 96.8, 84.3, 73.4, 71.7, 71.2, 68.7, 66.5, 65.4, 63.5, 62.6, 61.5, 60.2, 55.6, 42.7, 41.9, 41.6, 41.1, 40.1, 40.0, 39.9, 39.5, 38.8, 37.7, 37.4, 33.8, 32.8, 28.7, 27.0, 26.5, 21.6, 19.5, 17.4, 10.9, 10.2, 7.1, 5.2, -1.4

HRMS (ESI+) calculated for $C_{64}H_{101}O_{14}Si_3F_3Na$ $[M+Na]^+$: 1257.6349; found 1257.6316

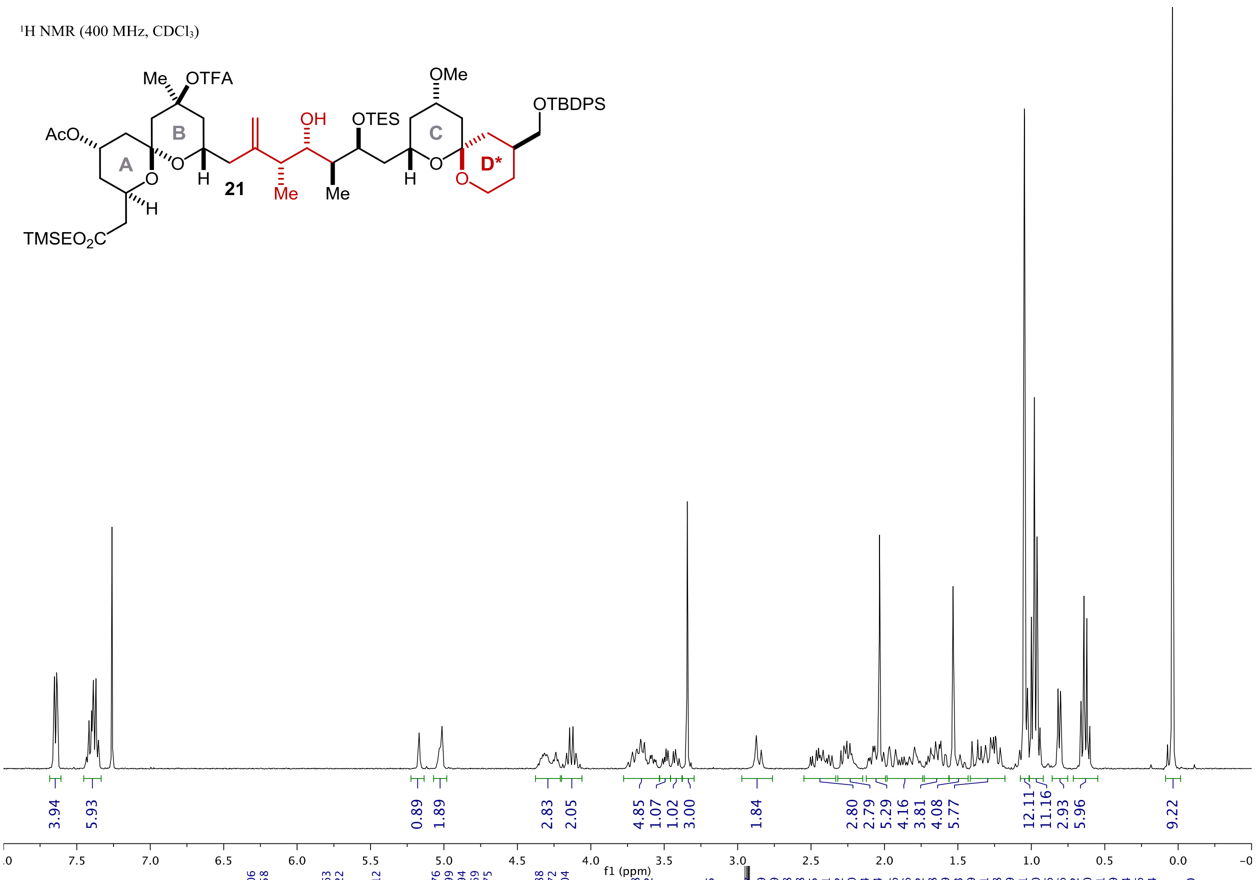

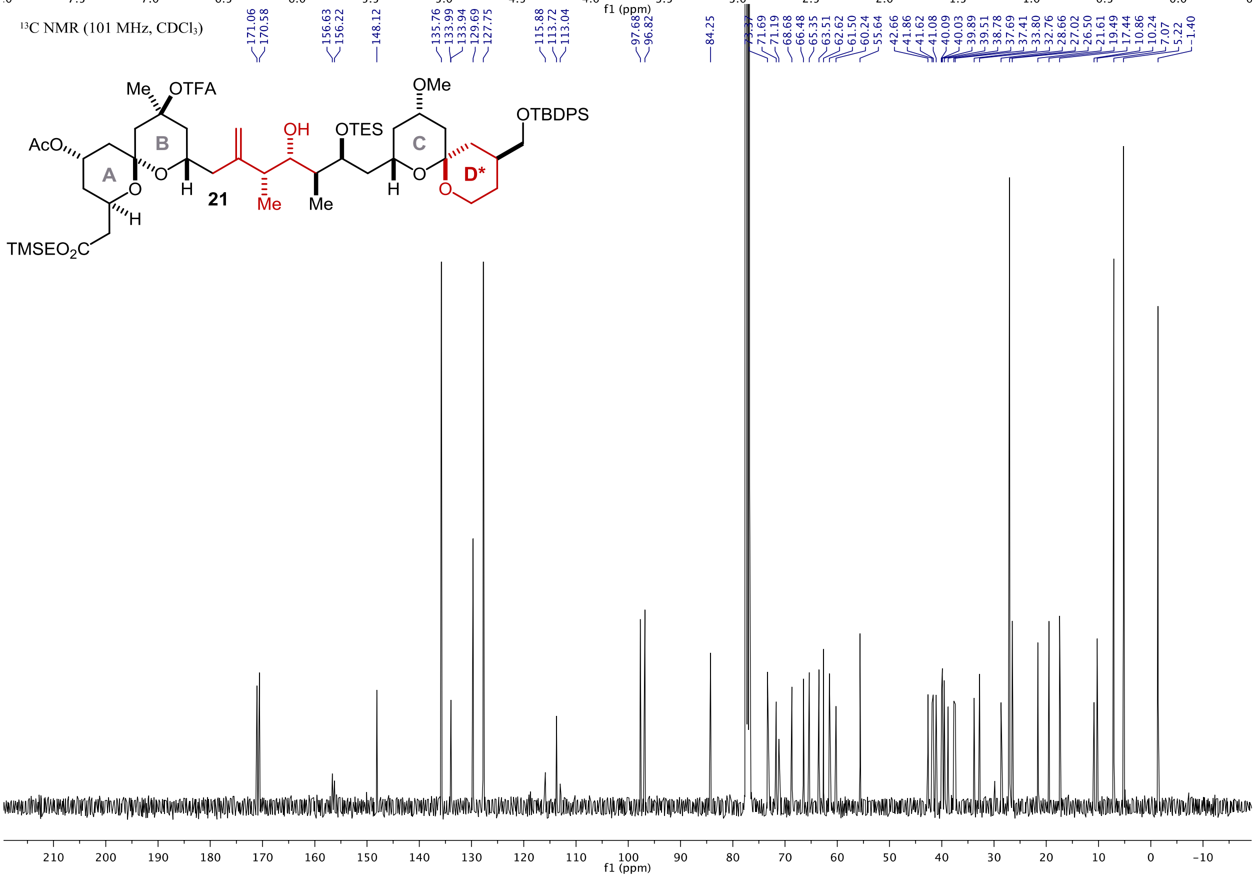

Supplementary Figure 72. ^{1}H NMR and ^{13}C NMR of **21**.

Synthesis of **25** from **12** and **9b**

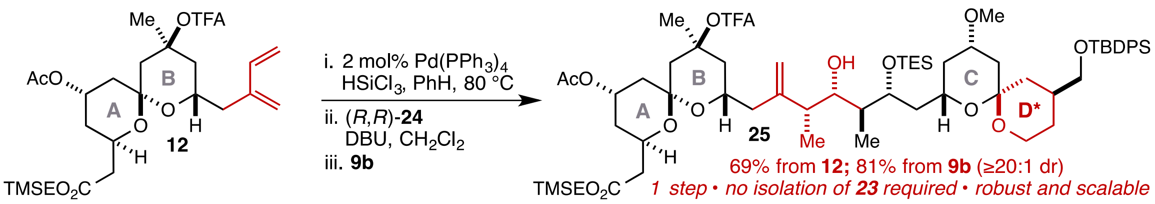

Supplementary Figure 73. Synthesis of **25**.

To a solution of diene **12** (838 mg, 1.49 mmol) in C_6H_6 (15 mL) in a 100 mL-capacity, septum-adapted pressure tube was added $Pd(PPh_3)_4$ (34 mg, 0.029 mmol). The tube was evacuated and back-filled with N_2 (3x). $HSiCl_3$ (0.40 mL, 3.0 mmol) was added and the reaction vessel was sealed with a teflon lined screw cap and heated at 80 °C for 12 h. The reaction mixture was cooled to room temperature. The reaction vessel was attached to a vacuum line equipped with a manometer and a -78 °C cold finger, and then placed in a warm (~35 °C) water bath. All volatiles were evaporated by application of vacuum until a viscous oil remained.

The resulting hydrosilylated product was dissolved in CH_2Cl_2 (11.0 mL) and the solution was cooled to 0 °C. A solution of diaminophenol (*R*,*R*)-**24** (351 mg, 1.41 mmol) and DBU (0.63 mL, 4.2 mmol) in CH_2Cl_2 (2.0 mL) was added, rinsing with CH_2Cl_2 (2 x 1 mL). The mixture was warmed to room temperature and stirred for 1 h. The reaction mixture was cooled back to 0 °C and aldehyde **9b** (850 mg, 1.27 mmol) was added as a solution in CH_2Cl_2 (2.0 mL), rinsing with CH_2Cl_2 (3 x 0.5 mL). The N_2 inlet was removed and the septum was parafilmed. The mixture was stirred for 12 h during which time the ice/water bath was allowed to warm to room temperature. The reaction mixture was cooled to 0 °C and treated with TBAF•$3H_2O$ (400 mg, 1.3 mmol). After 1.5 h the reaction mixture was filtered through a plug of silica gel, rinsing with EtOAc. The filtrate was concentrated, and analysis of the residue by ^1H NMR spectroscopy revealed that the product was formed with ≥20:1 diastereoselectivity. The residue was purified by silica gel column chromatography eluting with 0:100 → 60:40 EtOAc:Hexanes to give **25** as a beige foam (1.27 g, 1.03 mmol, ≥20:1 dr, 69% from **12**, 81% from **9b**).

OR $[\alpha]_D^{26}$ -4.5° (*c* 1.0, $CHCl_3$)

IR (thin film, cm^{-1}) 2954, 1777, 1734, 1379, 1247, 1215, 1163, 1134, 1094, 1060, 998, 837, 753, 704, 505

^{1}H NMR (500 MHz, $CDCl_3$) δ 7.68 – 7.57 (m, 4H), 7.45 – 7.33 (m, 6H), 5.15 (d, *J* = 1.9 Hz, 1H), 5.07 – 5.01 (m, 1H), 5.00 (s, 1H), 4.41 (dt, *J* = 9.2, 2.8 Hz, 1H), 4.35 – 4.29 (m, 2H), 4.20 – 4.04 (m, 2H), 3.83 – 3.75 (m, 1H), 3.75 – 3.59 (m, 3H), 3.50 (dd, *J* = 9.9, 4.9 Hz, 1H), 3.41 (dd, *J* = 9.9, 6.1 Hz, 1H), 3.34 (s, 4H), 2.84 (dd, *J* = 15.2, 2.2 Hz, 1H), 2.54 – 2.42 (m, 2H), 2.33 (dd, *J* = 14.3, 8.9 Hz, 1H), 2.29 – 2.15 (m, 3H), 2.14 – 2.04 (m, 2H), 2.02 (s, 3H), 2.02 – 1.98 (m, 1H), 1.96 – 1.84 (m, 3H), 1.81 – 1.78 (m, 1H), 1.73 – 1.56 (m, 4H), 1.55 – 1.43 (m, 5H), 1.42 – 1.35 (m, 2H), 1.34 – 1.14 (m, 5H), 1.13- 1.04 (s, 4H), 1.04 (s, 9H), 1.02 – 0.91 (m, 11H), 0.85 (d, *J* = 6.9 Hz, 3H), 0.64 (q, *J* = 7.7 Hz, 6H)

^{13}C NMR (126 MHz, $CDCl_3$) δ 171.0, 170.5, 156.6, 156.2, 148.3, 135.7, 133.9, 129.7, 127.7, 115.6, 113.9, 113.3, 97.6, 96.8, 84.2, 73.4, 72.0, 71.0, 68.6, 66.5, 66.4, 63.5, 62.6, 61.4, 60.5, 55.6, 42.7, 42.1, 41.9, 41.1, 39.9, 39.9, 38.9, 38.8, 38.6, 37.7, 33.8, 32.8, 28.6, 27.0, 26.5, 21.6, 19.5, 17.4, 10.5, 10.0, 7.2, 5.6, -1.4

HRMS (ESI+) calculated for $C_{64}H_{101}O_{14}Si_3F_3Na$ $[M+Na]^+$: 1257.6349; found 1257.6335

¹H NMR (500 MHz, CDCl₃)

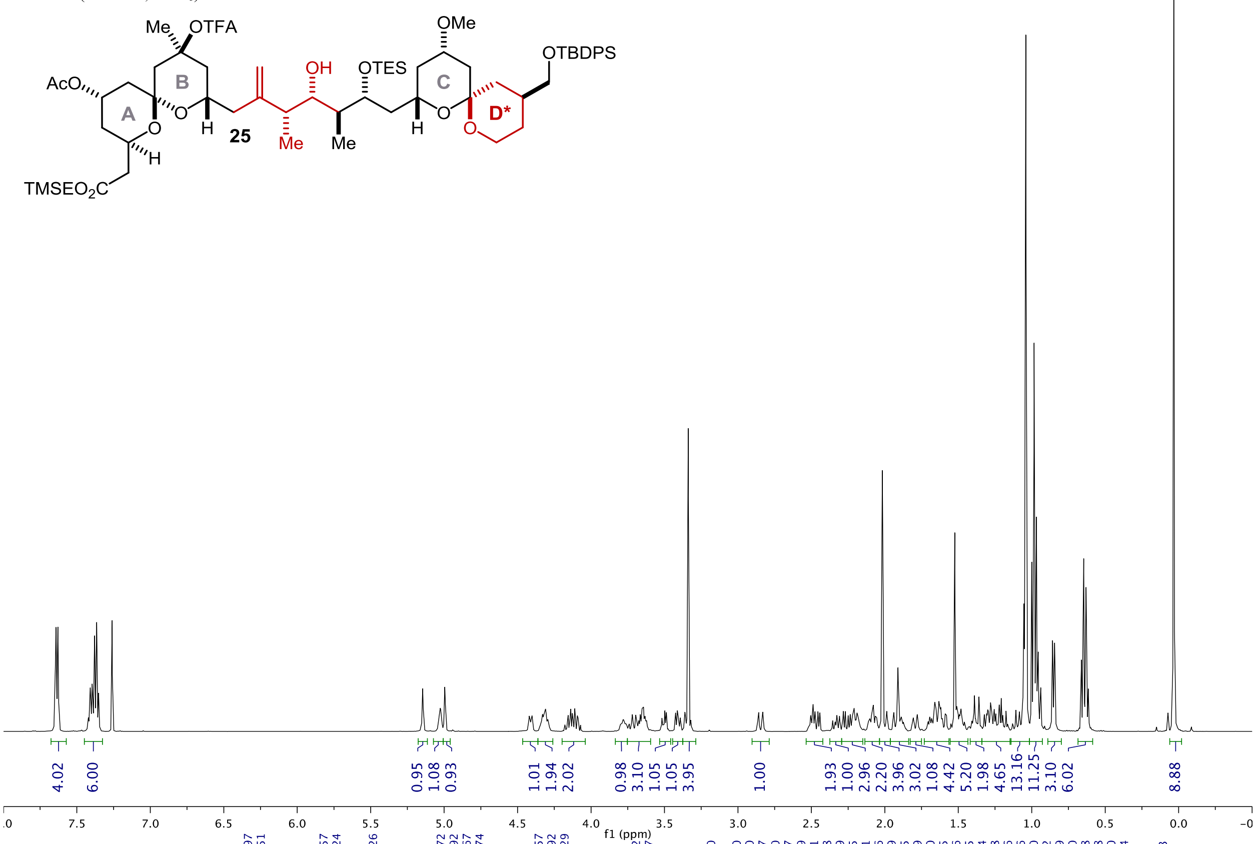

¹³C NMR (126 MHz, CDCl₃)

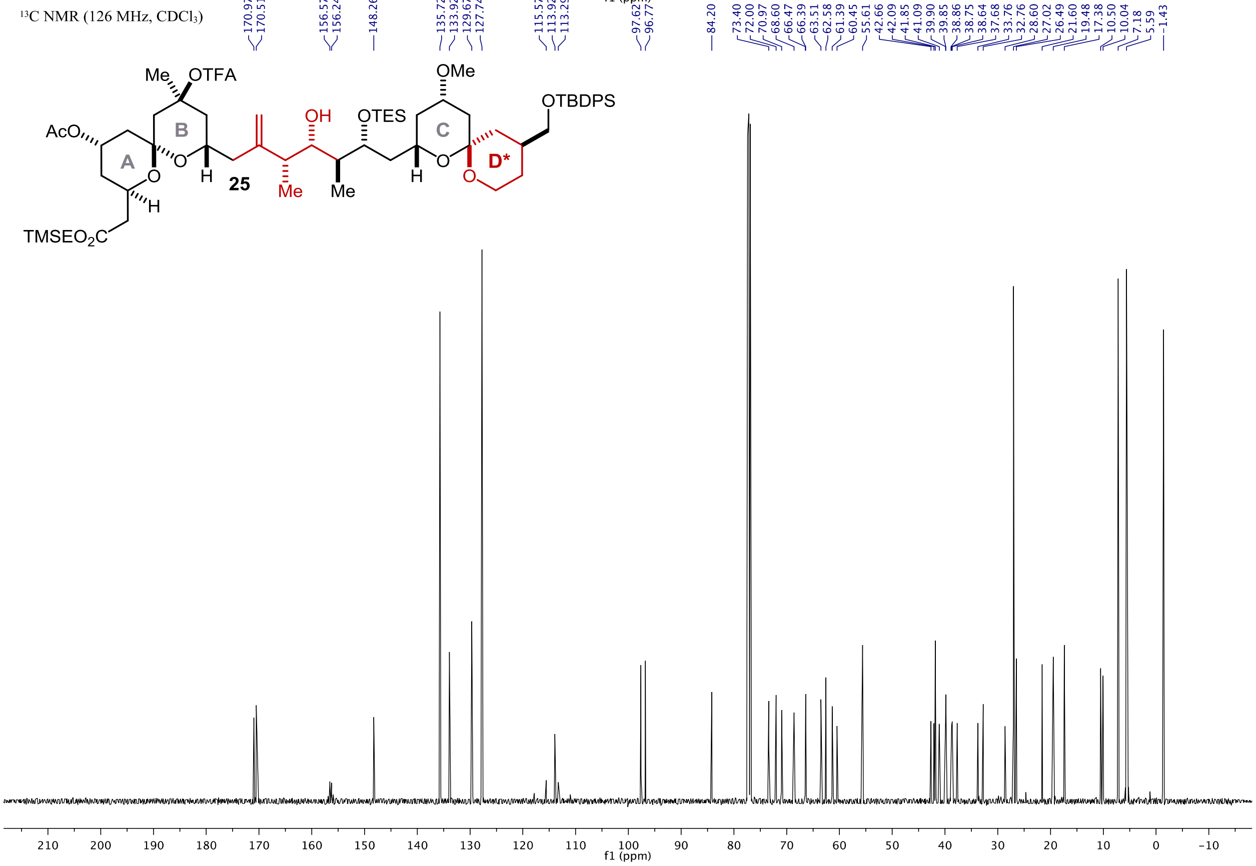

Supplementary Figure 74. ^{1}H NMR and ^{13}C NMR of **25**.

Synthesis of **6a** from **21**

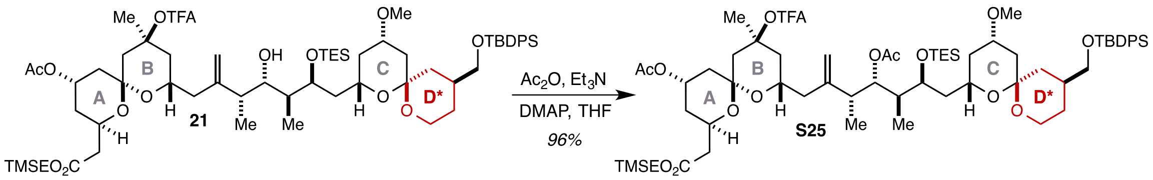

Supplementary Figure 75. Synthesis of **S25**.

To a cooled (0 °C) solution of **21** (1.08 g, 0.874 mmol) in THF (9.0 mL) at 0 ºC was added Et_3N (1.22 mL, 8.73 mmol), Ac_2O (0.50 mL, 5.24 mmol), and DMAP (0.32 g, 2.62 mmol), sequentially. The resulting mixture was warmed to room temperature and stirred for 15 h. Saturated aqueous $NaHCO_3$ (20 mL) was added and the aqueous layer was extracted with EtOAc (3 x 10 mL). The combined organic layers were washed with H_2O (1 x 20 mL), dried over Na_2SO_4, filtered, and concentrated in vacuo. The residue was purified by silica gel column chromatography eluting with 5:95 → 30:70 EtOAc:Hexanes to give **S25** as a beige foam (1.07 g, 0.837 mmol, 96% yield).

OR $[\alpha]_D^{20}$ -2.1° (*c* 1.0, $CHCl_3$)

IR (thin film, cm^{-1}) 2953, 2882, 1779, 1735, 1379, 1246, 1216, 1165, 1100, 999, 859, 838, 704

^{1}H NMR (400 MHz, $CDCl_3$) δ 7.69 – 7.56 (m, 4H), 7.49 – 7.29 (m, 6H), 5.20 – 5.12 (m, 1H), 5.08 – 4.99 (m, 1H), 4.97 (s, 1H), 4.82 (s, 1H), 4.30 – 4.18 (m, 2H), 4.18 – 4.10 (m, 2H), 4.10 – 4.03 (m, 1H), 3.76 – 3.51 (m, 4H), 3.46 (dd, *J* = 9.9, 5.7 Hz, 1H), 3.37 (dd, *J* = 9.9, 6.6 Hz, 1H), 3.33 (s, 3H), 2.85 (dd, *J* = 15.2, 2.2 Hz, 1H), 2.68 – 2.56 (m, 1H), 2.56 – 2.40 (m, 2H), 2.34 – 2.22 (m, 2H), 2.22 – 2.11 (m, 1H), 2.11 – 2.03 (m, 1H), 2.02 (s, 3H), 1.99 – 1.86 (m, 3H), 1.89 (s, 3H), 1.85 – 1.55 (m, 6H), 1.53 (s, 3H), 1.51 – 1.41 (m, 1H), 1.41 – 1.11 (m, 6H), 1.08 (d, *J* = 6.8 Hz, 3H), 1.03 (s, 9H), 0.97 (t, *J* = 8.0 Hz, 14H), 0.89 (d, *J* = 6.8 Hz, 3H), 0.60 (q, *J* = 7.9 Hz, 6H), 0.04 (s, 9H)

^{13}C NMR (101 MHz, $CDCl_3$) δ 171.1, 170.6, 170.0, 156.6, 156.2, 147.8, 135.7, 135.7, 133.8, 133.7, 129.8, 129.8, 127.8, 115.9, 113.5, 113.1, 97.6, 96.8, 84.3, 73.8, 73.1, 68.7, 67.6, 66.3, 65.3, 63.3, 62.6, 61.6, 60.1, 55.6, 42.9, 42.3, 41.9, 41.9, 41.0, 40.1, 39.0, 38.2, 37.7, 37.4, 33.8, 33.0, 28.9, 27.0, 26.5, 21.6, 21.0, 19.4, 17.5, 10.8, 8.4, 7.2, 5.6, -1.4

HRMS (ESI+) calculated for $C_{66}H_{103}O_{15}SiF_3Na$ $[M+Na]^+$: 1299.6455; found 1299.6426

^{1}H NMR (400 MHz, CDCl$_3$)

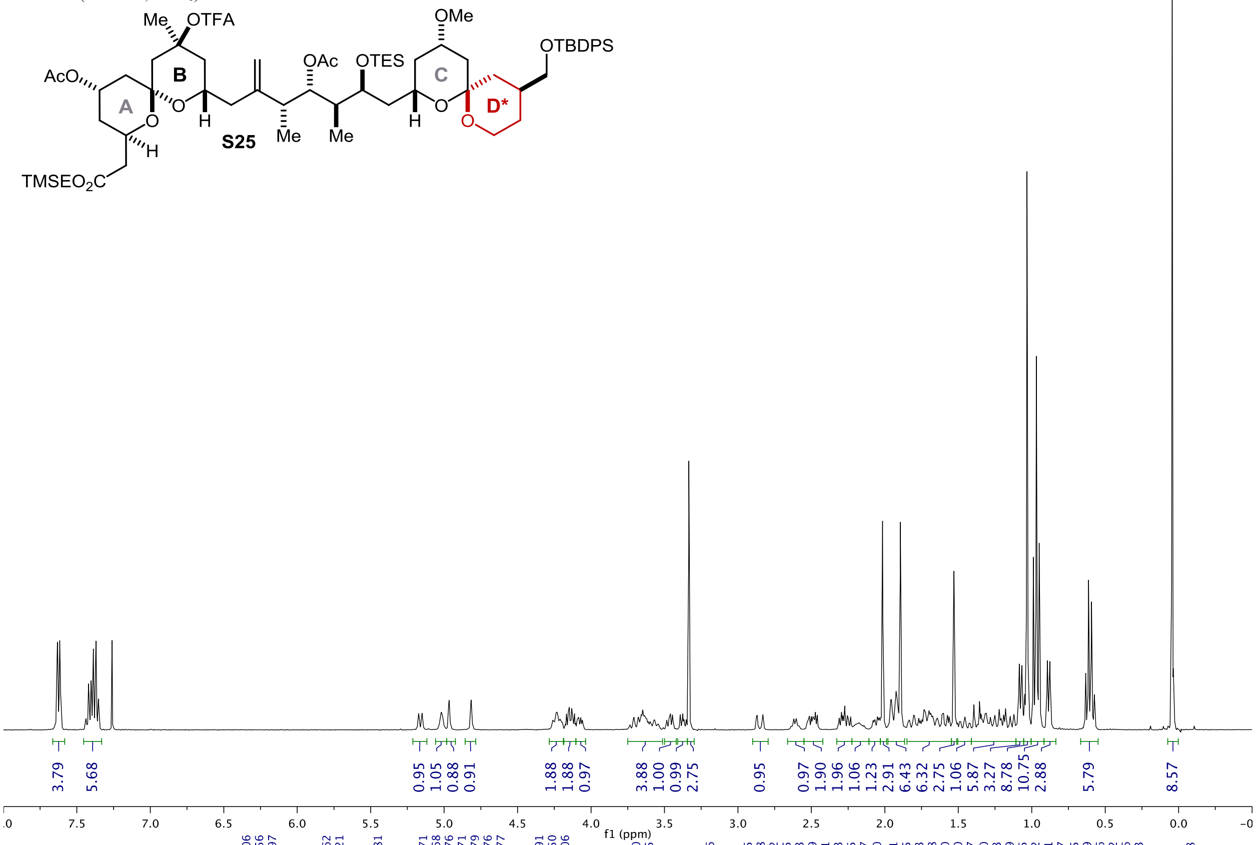

^{13}C NMR (101 MHz, CDCl$_3$)

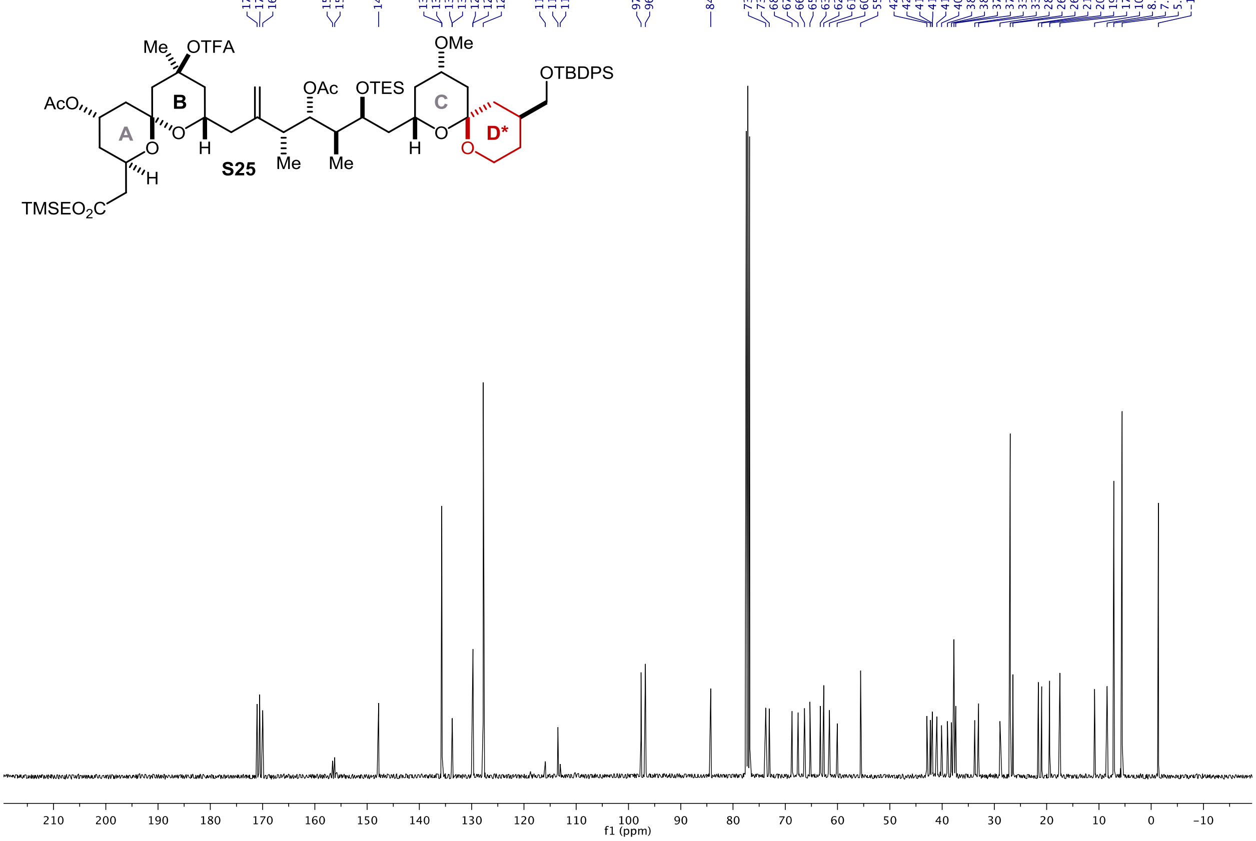

Supplementary Figure 76. ^{1}H NMR and ^{13}C NMR of **S25**.

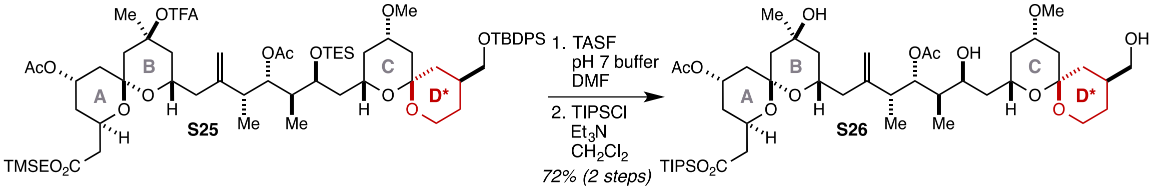

Supplementary Figure 77. Synthesis of **S26**.

To a solution of **S25** (426 mg, 0.333 mmol) in DMF (4.0 mL) was added distilled H_2O (192 µL) and tris(dimethylamino)sulfonium difluorotrimethylsilicate (TASF) (1.01 g, 3.7 mmol), and the vial containing the TASF was rinsed with DMF (2.0 mL). After 15 min the reaction mixture was heated to 50 °C (oil bath, external temperature). After 10 h, TLC analysis indicated complete conversion to product. The reaction mixture was cooled to room temperature and the excess TASF was quenched by the addition of pH 7 buffer (8.0 mL). The mixture was extracted with EtOAc (5 x 3 mL) and the combined organic layers were washed with H_2O (1 x 3 mL), dried over Na_2SO_4, filtered, and concentrated. Filtration through a plug of silica gel eluting with 0:100 → 10:90 MeOH:CH_2Cl_2 gave a beige foam, which was azeotroped with toluene (4 x) before being used immediately in the next step.

To a solution of this material in CH_2Cl_2 (3.0 mL) was added Et_3N (60 µL, 0.43 mmol) and TIPSCl (68 µL, 0.32 mmol). After 20 min saturated aqueous $NaHCO_3$ (3 mL) was added. The layers were separated and the aqueous layer was extracted with CH_2Cl_2 (3 x 1 mL). The combined organic layers were dried over Na_2SO_4, filtered, and concentrated. The residue was purified by silica gel column chromatography eluting with 0:100 → 5:95 MeOH:CH_2Cl_2 to give **S26** as a beige foam (212 mg, 0.239 mmol, 72% yield over 2 steps from **S25**).

OR $[\alpha]_D^{20}$ +16.4° (*c* 1.0, $CHCl_3$)

IR (thin film, cm^{-1}) 3530, 2945, 2870, 1718, 1462, 1370, 1245, 1183, 1092, 1020, 991, 958, 885, 753

^{1}H NMR (400 MHz, $CDCl_3$) δ 5.09 – 5.02 (m, 2H), 5.00 (dd, *J* = 9.8, 2.6 Hz, 1H), 4.86 (s, 1H), 4.41 – 4.22 (m, 2H), 4.03 (s, 1H), 3.87 – 3.72 (m, 2H), 3.72 – 3.54 (m, 3H), 3.52 – 3.36 (m, 2H), 3.33 (s, 3H), 2.97 (d, *J* = 2.5 Hz, 1H), 2.66 – 2.52 (m, 2H), 2.46 (dd, *J* = 17.2, 4.7 Hz, 1H), 2.35 (dd, *J* = 14.4, 8.1 Hz, 1H), 2.21 – 2.07 (m, 2H), 2.07 – 1.98 (m, 2H), 2.05 (s, 3H), 2.01 (s, 3H), 1.98 – 1.91 (m, 1H), 1.90 – 1.69 (m, 5H), 1.69 – 1.61 (m, 3H), 1.61 – 1.56 (m, 1H), 1.56 – 1.46 (m, 3H), 1.46 – 1.38 (m, 1H), 1.35 – 1.16 (m, 8H), 1.14 (s, 3H), 1.09-1.01 (m, 3H), 1.05 (d, *J* = 7.5 Hz, 18H), 0.99 – 0.85 (m, 1H), 0.95 (d, 3H).

^{13}C NMR (101 MHz, $CDCl_3$) δ 171.4, 171.1, 170.4, 147.5, 113.3, 98.3, 97.9, 75.2, 73.0, 68.7, 68.1, 67.8, 66.2, 65.0, 61.4, 60.2, 55.6, 46.3, 43.9, 42.3, 41.6, 41.5, 40.7, 39.7, 38.8, 38.5, 37.7, 34.1, 33.2, 30.1, 28.3, 21.7, 20.9, 17.9, 17.9, 12.0, 11.9, 9.4

HRMS (FAB+) calculated for $C_{46}H_{81}O_{14}Si$ $[M+H]^+$: 885.5396; found 885.5422

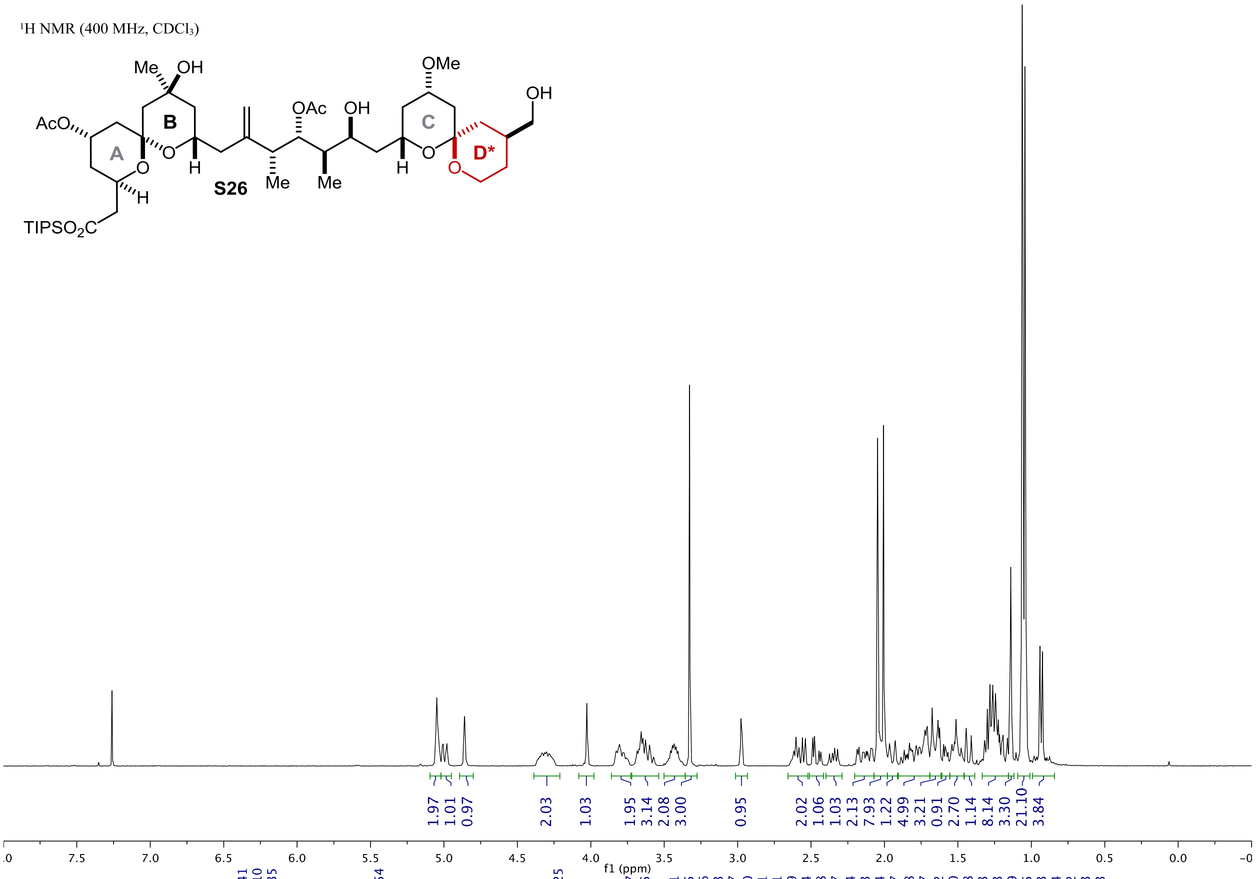

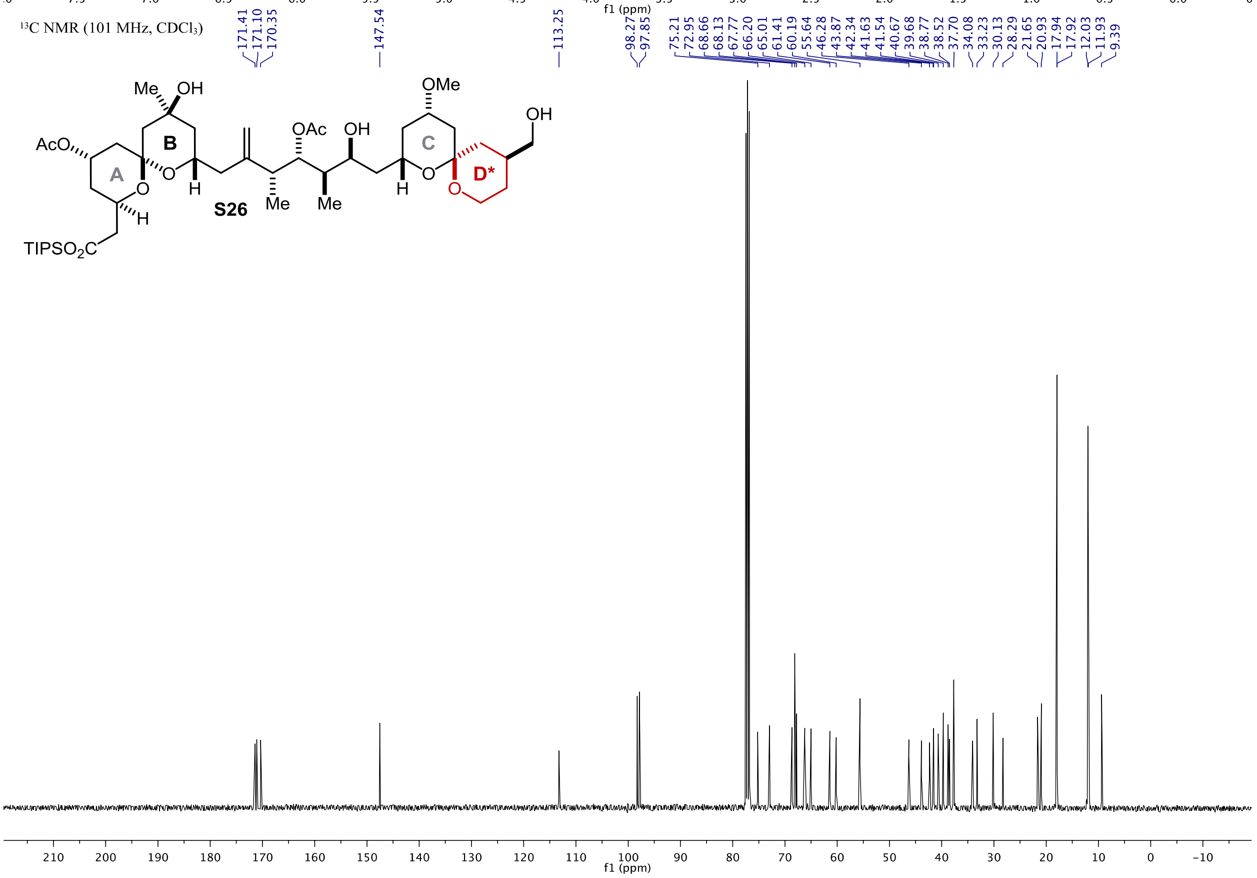

Supplementary Figure 78. ^{1}H NMR and ^{13}C NMR of **S26**.

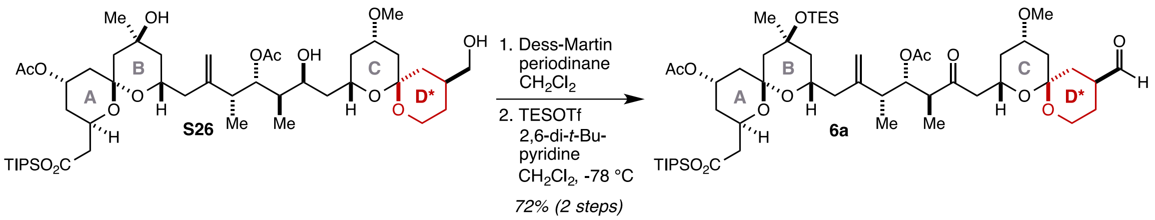

Supplementary Figure 79. Synthesis of **6a**.

To a solution of **S26** (102 mg, 0.115 mmol) in CH_2Cl_2 (7.7 mL) was added Dess-Martin periodinane (147 mg, 0.35 mmol). After 1.5 h the reaction mixture was cooled to 0 °C and 10 mL of a 1:1 solution of saturated aqueous $NaHCO_3$ and saturated aqueous $Na_2S_2O_3$ was added slowly. The mixture was warmed to room temperature and after 30 min the layers were separated and the aqueous layer was extracted with CH_2Cl_2 (3 x 5.0 mL). The combined organic layers were dried over MgSO4, filtered, and concentrated. The residue was filtered through a plug of pH 7 buffered silica gel with 0:100 → 70:30 EtOAc:Hexanes to give a beige foam (88 mg), which was used immediately in the next step without further purification.

To a solution of 61 mg of this product in CH_2Cl_2 (1.4 mL) was added 2,6-di-*tert*-butylpyridine (155 μL, 0.69 mmol). The mixture was cooled to -78 °C and TESOTf (78 μL, 0.35 mmol) was added slowly. After 2h, the reaction mixture was quenched by the addition of saturated aqueous $NaHCO_3$ (3.0 mL) and the mixture was warmed to room temperature over 10 minutes. The layers were separated and the aqueous layer was extracted with CH_2Cl_2 (3 x 2 mL). The combined organic layers were dried over $MgSO_4$, filtered, and concentrated. The residue was purified by silica gel column chromatography with pH 7 buffered silica gel eluting with 0:100 → 60:40 EtOAc:Hexanes to give **6a** as a beige foam (58 mg, 0.058 mmol, 72% yield over 2 steps from **S26**).

OR $[\alpha]_D^{20}$ +13.3° (*c* 1.0, $CHCl_3$)

IR (thin film, cm^{-1}) 2946, 2872, 1725, 1460, 1371, 1235, 1182, 1146, 1062, 1005, 885, 740, 673, 492

^{1}H NMR (400 MHz, $CDCl_3$) δ 9.57 (s, 1H), 5.29 (dd, *J* = 9.7, 2.9 Hz, 1H), 5.06 – 5.01 (m, 1H), 4.99 (s, 1H), 4.84 (s, 1H), 4.29 – 4.09 (m, 3H), 3.88 – 3.76 (m, 1H), 3.74 – 3.62 (m, 2H), 3.33 (s, 3H), 2.94 – 2.76 (m, 2H), 2.76 – 2.61 (m, 2H), 2.53 – 2.34 (m, 3H), 2.33 – 2.24 (m, 2H), 2.16 – 2.07 (m, 1H), 2.06 – 1.94 (m, 2H), 2.01 (s, 3H), 1.92 (s, 3H), 1.90 – 1.79 (m, 3H), 1.79 – 1.69 (m, 1H), 1.63 – 1.37 (m, 56H), 1.37 – 1.15 (m, 8H), 1.20 (s, 3H), 1.13 – 1.00 (m, 25H), 0.93 (t, *J* = 7.9 Hz, 9H), 0.55 (qd, *J* = 7.9, 2.1 Hz, 6H)

^{13}C NMR (101 MHz, $CDCl_3$) δ 209.2, 203.0, 171.1, 170.7, 169.6, 147.2, 113.9, 97.1, 97.0, 73.8, 72.8, 70.6, 66.8, 64.4, 64.2, 61.3, 59.6, 55.8, 48.7, 48.5, 47.8, 45.4, 43.4, 42.4, 42.2, 41.3, 38.7, 38.0, 37.0, 35.0, 34.2, 32.1, 24.9, 21.7, 20.9, 17.9, 12.9, 12.1, 12.1, 7.5, 7.5, 7.1

HRMS (ESI+) calculated for $C_{52}H_{90}O_{14}Si_2Na$ $[M+Na]^+$: 1017.5767; found 1017.5761

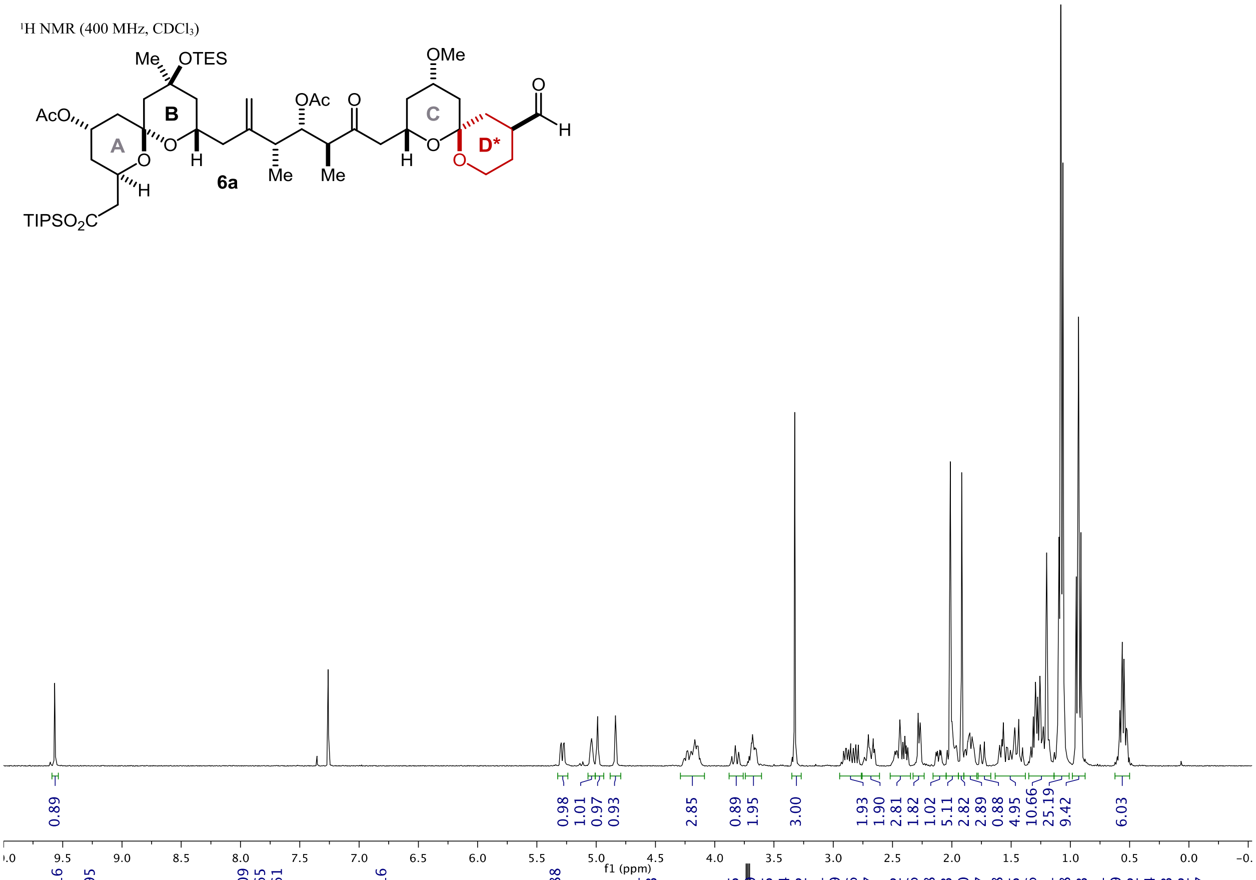

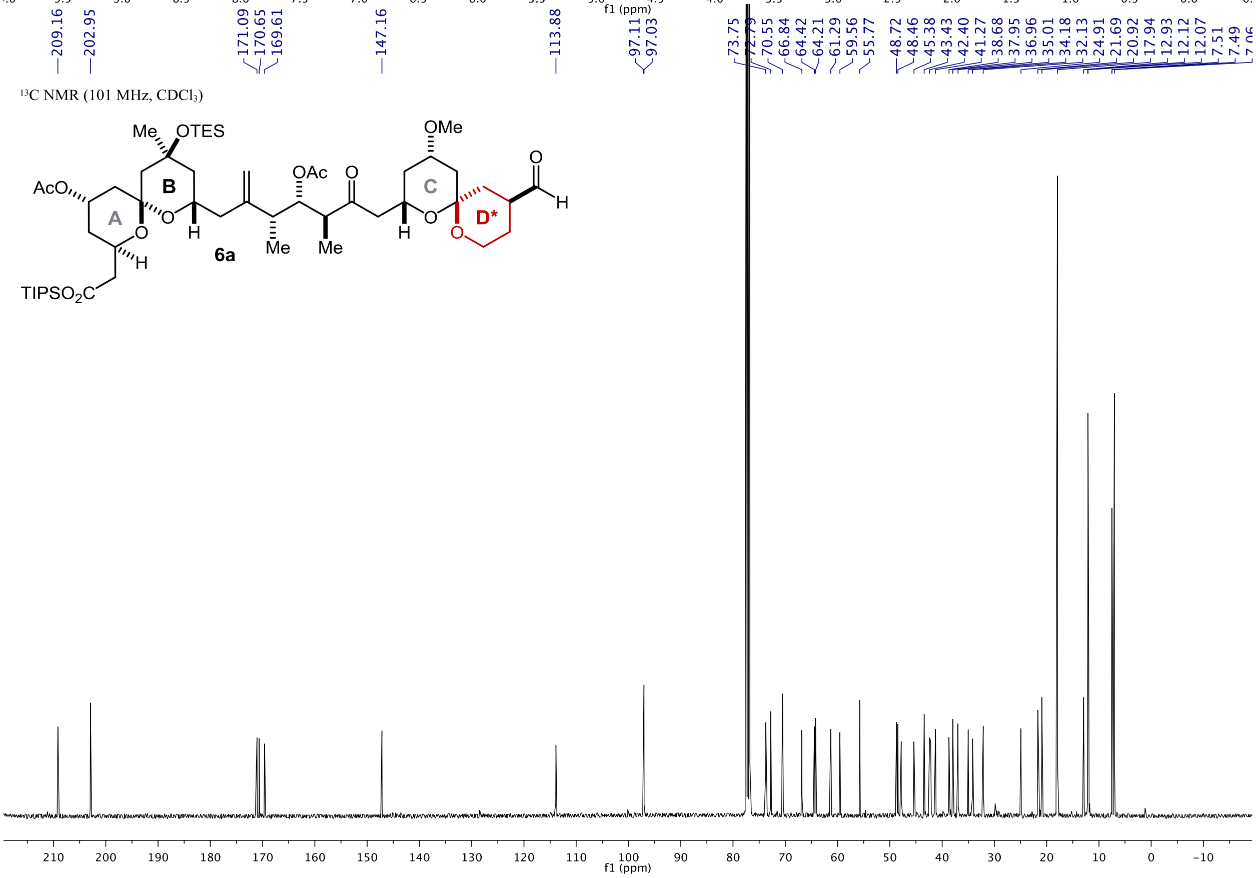

Supplementary Figure 80. ^{1}H NMR and ^{13}C NMR of **6a**.

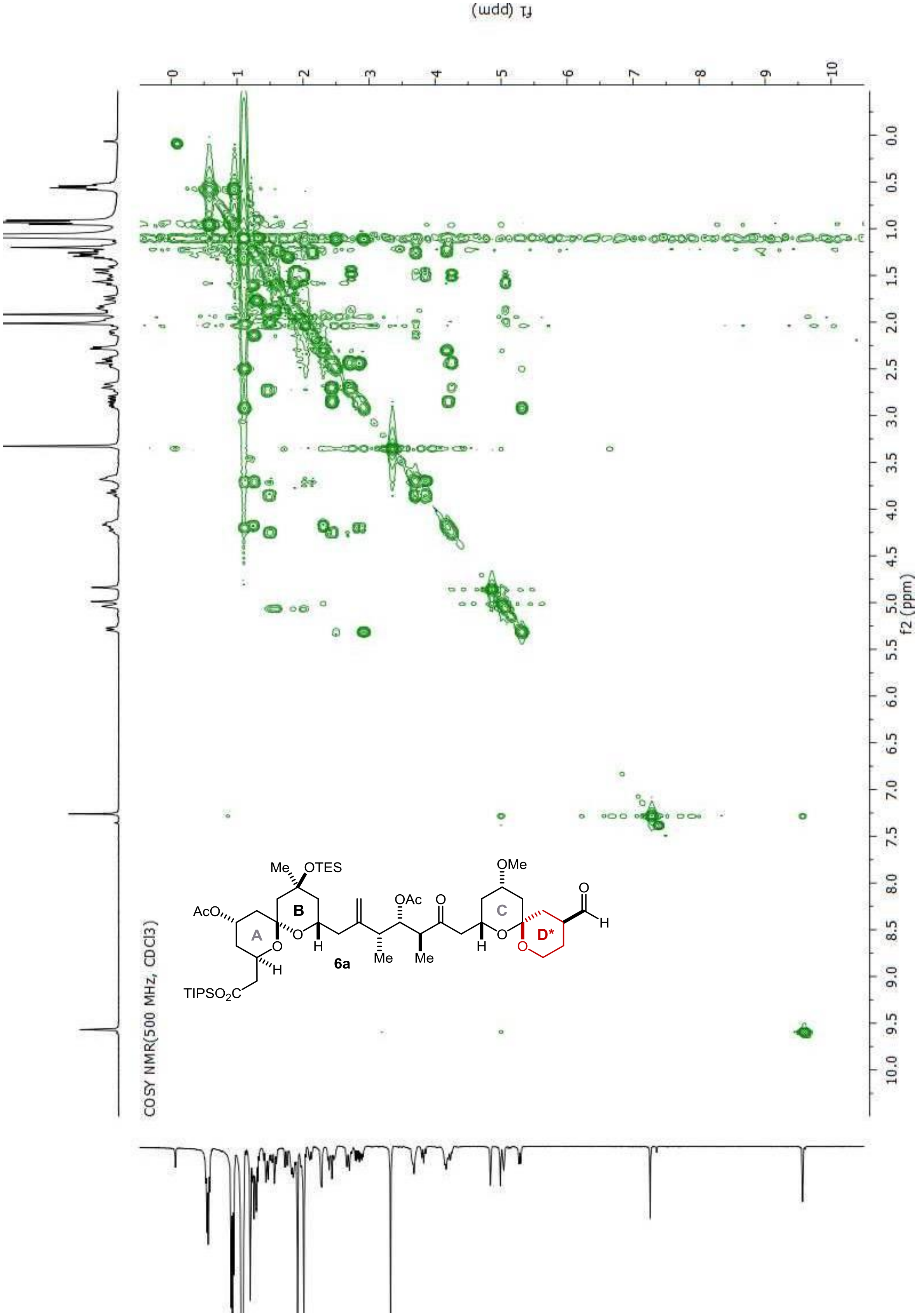

Supplementary Figure 81. COSY NMR of **6a**.

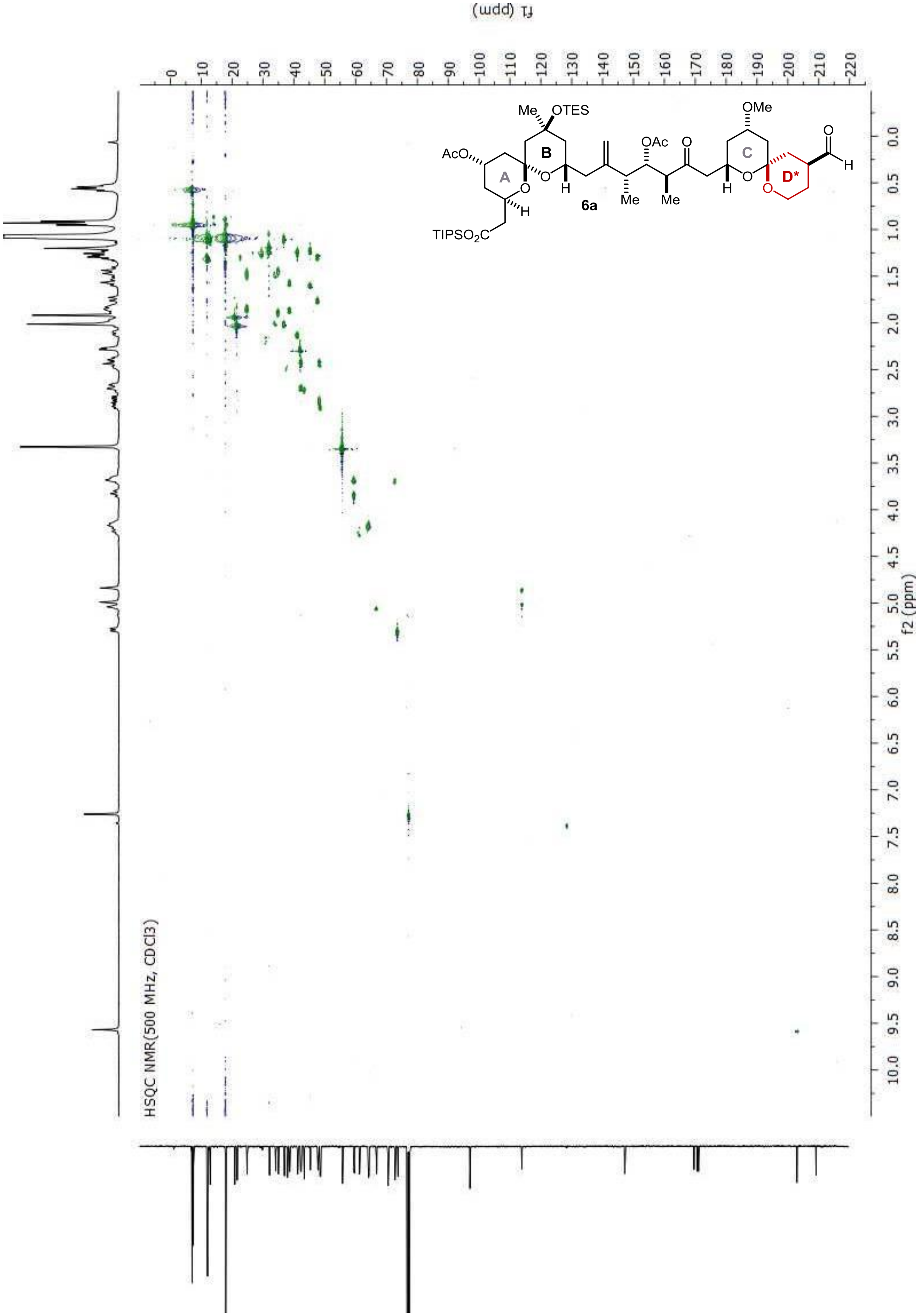

Supplementary Figure 82. HSQC NMR of **6a**.

Synthesis of azido acids **S28** and **S33**

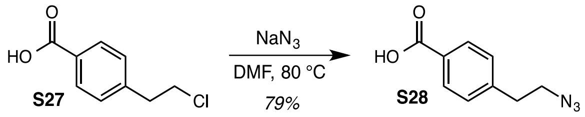

Supplementary Figure 83. Synthesis of **S28**.

To a solution of **S27** (3.00 g, 16.3 mmol) in DMF (50 mL) was added NaN_3 (2.11 g, 32.5 mmol). The mixture was heated at 80 °C for 12 h and the cooled to room temperature. Water (30 mL) was added to the reaction vessel and the mixture was extracted with EtOAc (3 x 20 mL). The combined organic layers were dried over $MgSO_4$, filtered, and concentrated. The crude product was purified by silica gel column chromatography eluting with 0:100 → 70:30 EtOAc:Hexanes to afford the desired product **S28** as a pale yellow solid (2.37 g, 79% yield). The ^{1}H NMR spectroscopic data is in agreement with data reported in literature.[12]

^{1}H NMR (300 MHz, $CDCl_3$) δ 8.15 – 7.99 (m, 2H), 7.41 – 7.28 (m, 2H), 3.56 (t, *J* = 7.1 Hz, 2H), 2.97 (t, *J* = 7.1 Hz, 2H).

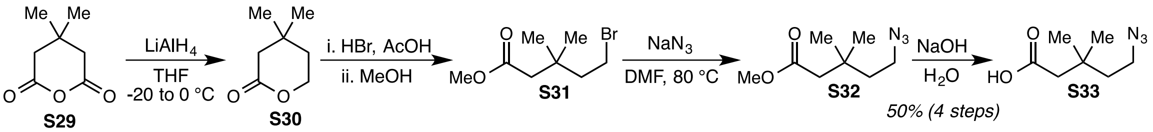

Supplementary Figure 84. Synthesis of **S33**.

To a cooled (-20 °C) solution of 3,3-dimethylglutaric anhydride **S29** (5.00 g, 35.0 mmol) in THF (175 mL) was added a $LiAlH_4$ (0.80 g, 21 mmol) in 3 equal portions in 5 min increments. The reaction mixture was slowly warmed to 0 °C and stirred for 1 h. Excess $LiAlH_4$ was quenched by the ***slow*** addition of 6 M HCl (45.0 mL), and the mixture was warmed to room temperature. After 20 min the mixture was extracted with EtOAc (3 x 50 mL) and the combined organic layers were dried over $MgSO_4$, filtered, and concentrated to provide crude **S30**, which was used without additional purification.

^{1}H NMR (500 MHz, $CDCl_3$) δ 4.40 – 4.32 (m, 2H), 2.32 (s, 2H), 1.69 (t, J = 6.1 Hz, 2H), 1.08 (s, 6H).

A mixture of lactone **S30** (205 mg, 1.6 mmol) and 33% HBr in AcOH (0.46 mL) was stirred at 75 °C for 1 h. Upon cooling to room temperature, MeOH (0.60 mL) was added and the resulting mixture was stirred for 48 h. All volatiles were removed *in vacuo* and the resulting residue was dissolved in EtOAc (5.0 mL) and washed with saturated $NaHCO_3$ (3 x 2.0 mL). The organic layer was dried over Mg_2SO_4, filtered, and concentrate to afford crude **S31** as a colorless oil. NMR analysis of the crude indicated an 85:15 mixture of product **S31** to side products (**S30** and bromoacid) that was used without further purification.

^{1}H NMR (400 MHz, $CDCl_3$) δ 3.67 (s, 3H), 3.46 – 3.36 (m, 2H), 2.22 (s, 2H), 2.01 – 1.91 (m, 2H), 1.03 (s, 6H).

To a solution of crude **S31** in DMF (4 mL) was added NaN_3 (210 mg, 3.2 mmol) and the resulting mixture was stirred at 70 °C for 2 h. The reaction mixture was quenched by the addition of H_2O (4 mL) and extracted with EtOAc (3 x 2 mL). The combined organic layers were dried over $MgSO_4$, filtered, and concentrated to provide the crude product **S32**, which was used without further purification.

^{1}H NMR (400 MHz, $CDCl_3$) δ 3.65 (s, 3H), 3.35 – 3.23 (m, 2H), 2.23 (s, 2H), 1.71 – 1.61 (m, 2H), 1.02 (s, 6H).

To a solution of crude **S32** in MeOH (0.4 mL) was added 1 M NaOH (2.4 mL, 2.4 mmol). The resulting reaction mixture was stirred at room temperature for 12 h. The reaction mixture was concentrated *in vacuo* and the resulting residue was treated with 1 M HCl until pH 1. The product was extracted with EtOAc (3 x 2 mL) and the combined organic layers were dried over $MgSO_4$, filtered, and concentrated. The crude product was purified by silica gel column chromatography eluting with 0:100 → 50:50 EtOAc:Hexanes to afford the desired product **S33** as a colorless oil (138 mg, 50% yield over 4 steps).

IR (thin film, cm^{-1}) 2963, 2092, 1702, 1472, 1409, 1249, 898, 670

^{1}H NMR (400 MHz, $CDCl_3$) δ 3.40 – 3.24 (m, 2H), 2.28 (s, 2H), 1.78 – 1.66 (m, 2H), 1.08 (s, 6H)

^{13}C NMR (101 MHz, $CDCl_3$) δ 177.7, 47.7, 45.8, 39.8, 32.6, 27.6

HRMS (APCI-) calculated for $C_7H_{12}N_3O_2$ $[M-H]^-$: 170.0930; found 170.0923

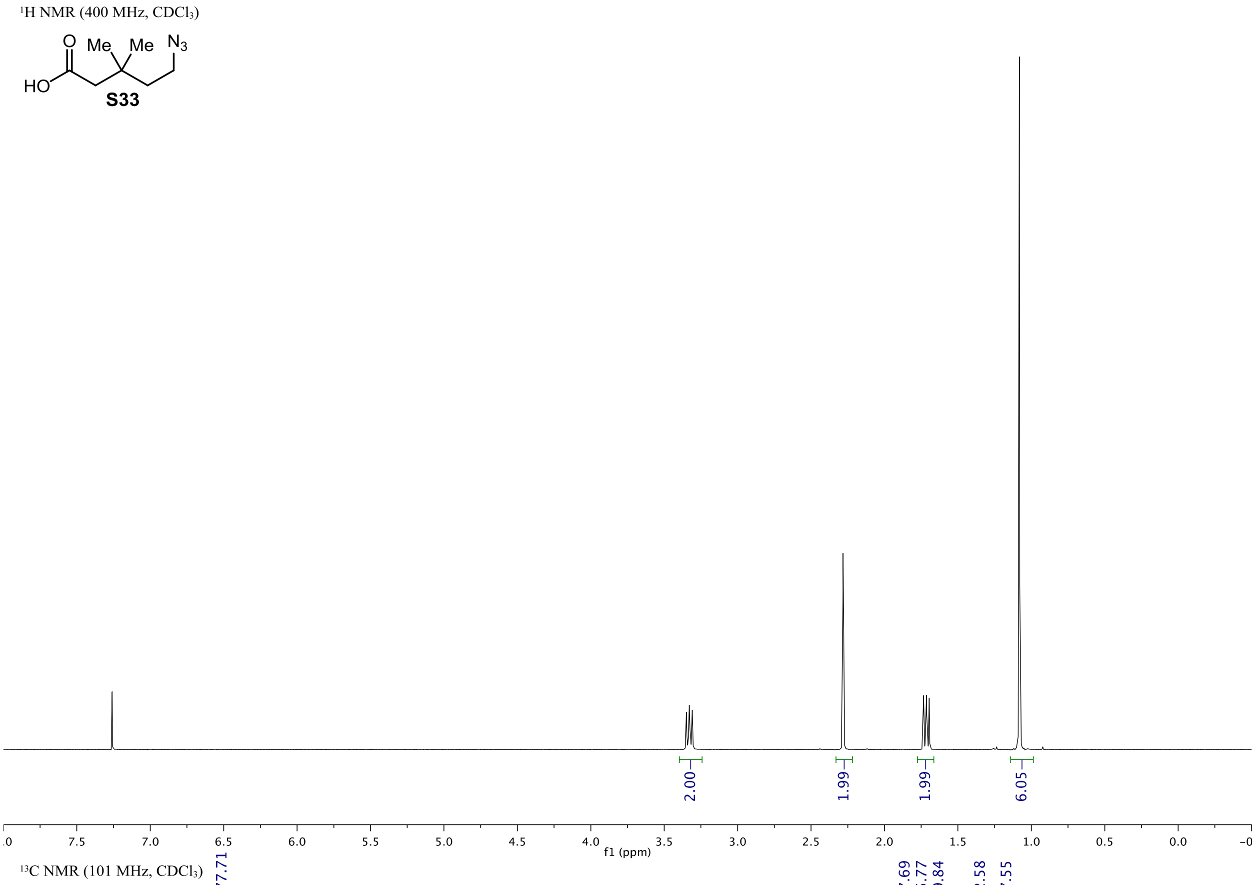

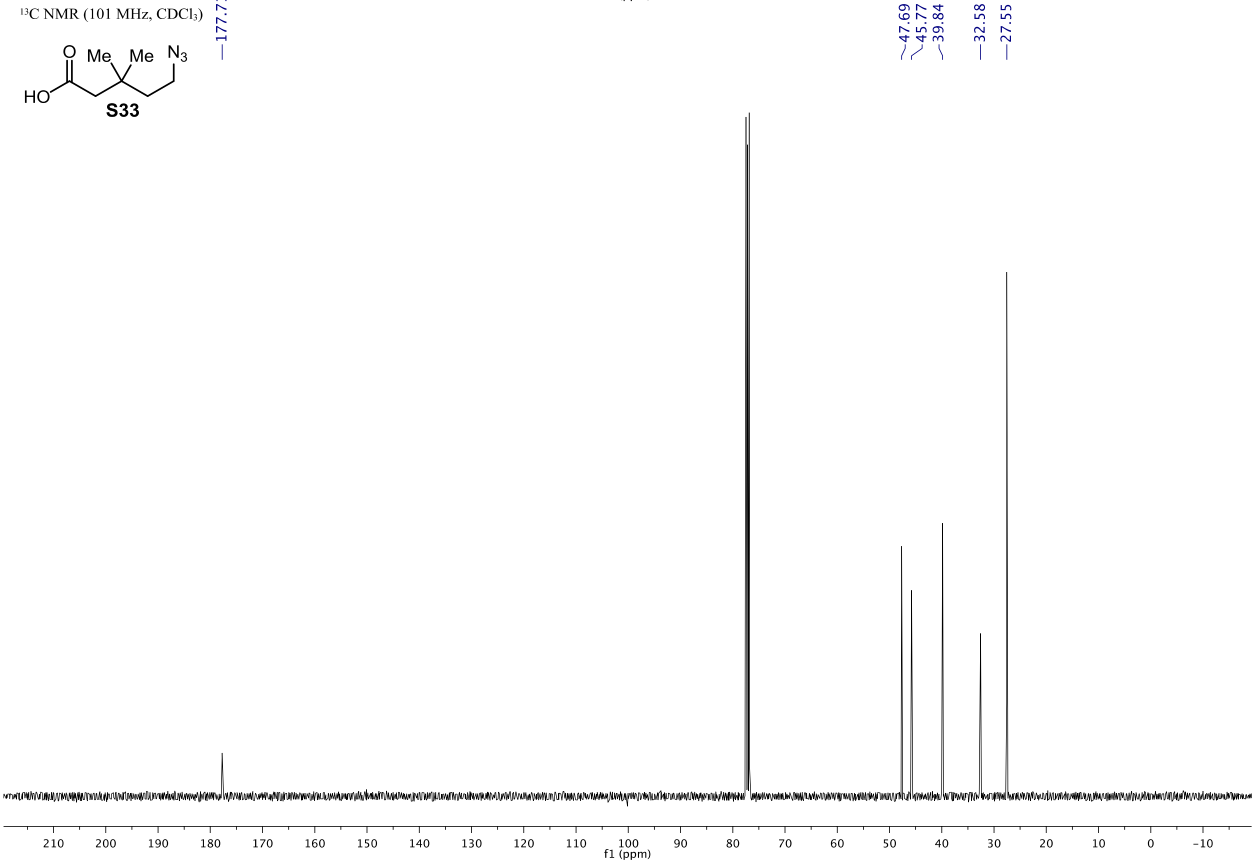

Supplementary Figure 85. ^{1}H NMR and ^{13}C NMR of **S33**.

Synthesis of **6b** from **25**

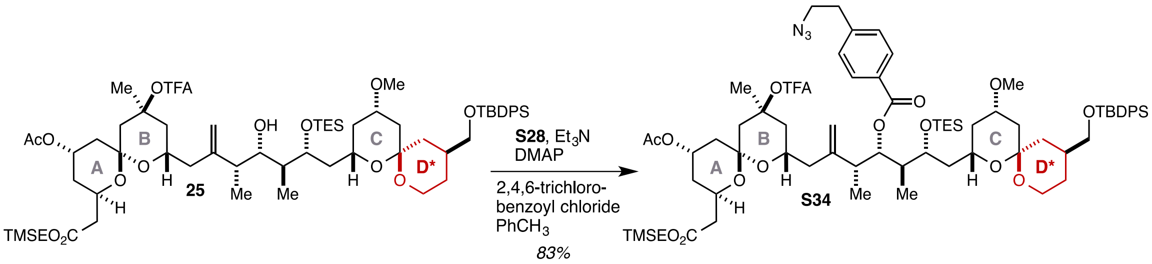

Supplementary Figure 86. Synthesis of **S34**.

To a cooled (0 °C) solution of alcohol **25** (400 mg, 0.324 mmol) in toluene (6.5 mL) was sequentially added benzoic acid **S28** (309 mg, 1.6 mmol), Et_3N (0.27 mL, 1.9 mmol), DMAP (198 mg, 1.6 mmol), and 2,4,6-trichlorobenzoyl chloride (278 µL, 1.8 mmol). The reaction mixture immediately became thick and cloudy upon addition of the 2,4,6-trichlorobenzoyl chloride. The mixture was warmed to room temperature. After 12 h the reaction mixture was quenched by the addition of saturated aqueous $NaHCO_3$ (10 mL). The mixture was extracted with EtOAc (3 x 3 mL) and the combined organic layers were dried over $MgSO_4$, filtered, and concentrated. The residue was purified by silica gel column chromatography eluting with 0:100 → 50:50 EtOAc:Hexanes to afford the desired product **S34** as a beige foam (378 mg, 0.268 mmol, 83% yield).

OR $[\alpha]_D^{24}$ +1.6° (*c* 1.0, $CHCl_3$)

IR (thin film, cm^{-1}) 2954, 2097, 1777, 1733, 1428, 1378, 1248, 1215, 1114, 1098, 999, 752, 667, 504

^{1}H NMR (400 MHz, $CDCl_3$) δ 7.91 – 7.78 (m, 2H), 7.73 – 7.59 (m, 4H), 7.52 – 7.34 (m, 6H), 7.00 (d, *J* = 8.1 Hz, 2H), 5.19 – 5.07 (m, 1H), 5.07 – 4.96 (m, 1H), 4.87 (s, 1H), 4.72 (s, 1H), 4.34 – 4.21 (m, 2H), 4.19 – 4.06 (m, 2H), 4.03 (dd, *J* = 10.3, 2.5 Hz, 1H), 3.67 – 3.52 (m, 2H), 3.44 – 3.37 (m, 2H), 3.35 – 3.26 (m, 2H), 3.31 (s, 3H), 3.21 (d, *J* = 4.2 Hz, 2H), 2.90 – 2.77 (m, 1H), 2.72 (q, *J* = 7.6 Hz, 1H), 2.65 (td, *J* = 7.1, 2.7 Hz, 2H), 2.53 – 2.38 (m, 2H), 2.34 – 2.18 (m, 3H), 2.13 – 1.95 (m, 4H), 1.95 – 1.84 (m, 1H), 1.91 (s, 3H), 1.83 – 1.67 (m, 3H), 1.58 (dd, *J* = 15.0, 4.2 Hz, 2H), 1.53 (s, 3H), 1.50 – 1.14 (m, 14H), 1.14 – 1.06 (m, 1H), 1.06 – 1.01 (m, 1H), 1.04 (s, 9H), 1.00 – 0.83 (m, 16H), 0.64 – 0.55 (m, 6H), 0.04 (s, 9H)

^{13}C NMR (101 MHz, $CDCl_3$) δ 171.0, 170.5, 164.9, 156.6, 156.2, 146.4, 143.1, 135.8, 135.8, 135.7, 134.0, 133.9, 129.8, 129.8, 129.7, 129.0, 128.8, 127.8, 127.8, 115.9, 114.4, 113.0, 97.5, 96.7, 84.3, 77.4, 74.5, 73.5, 70.5, 68.1, 66.4, 66.4, 63.3, 62.6, 61.4, 60.1, 55.5, 51.9, 42.9, 42.2, 41.8, 41.6, 41.0, 40.0, 38.6, 38.4, 38.1, 37.9, 37.7, 35.2, 33.8, 32.2, 28.1, 27.0, 26.5, 21.5, 19.5, 17.4, 11.5, 9.7, 7.2, 5.6, -1.4

HRMS (ESI+) calculated for $C_{73}H_{108}N_3O_{15}F_3Si_3Na$ $[M+Na]^+$: 1430.6938; found 1430.6936

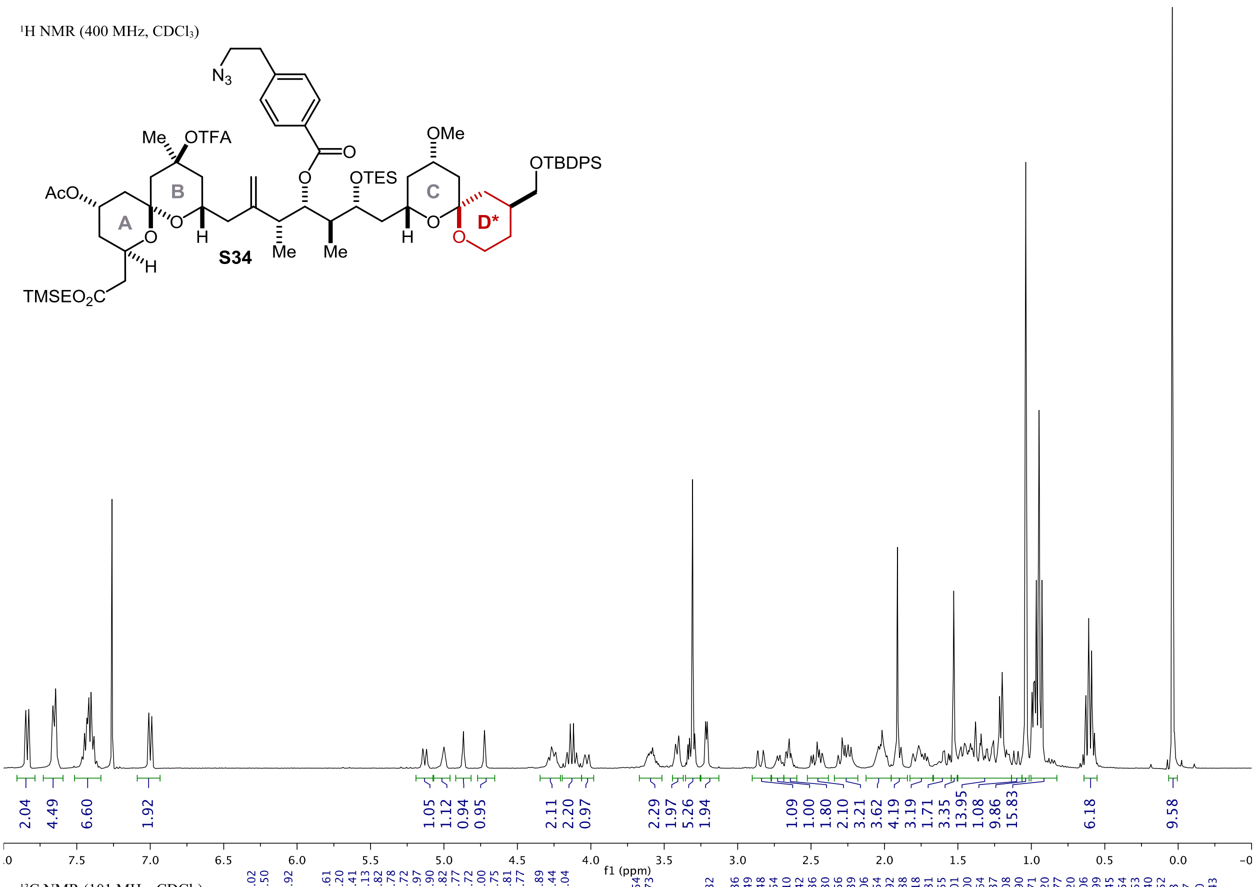

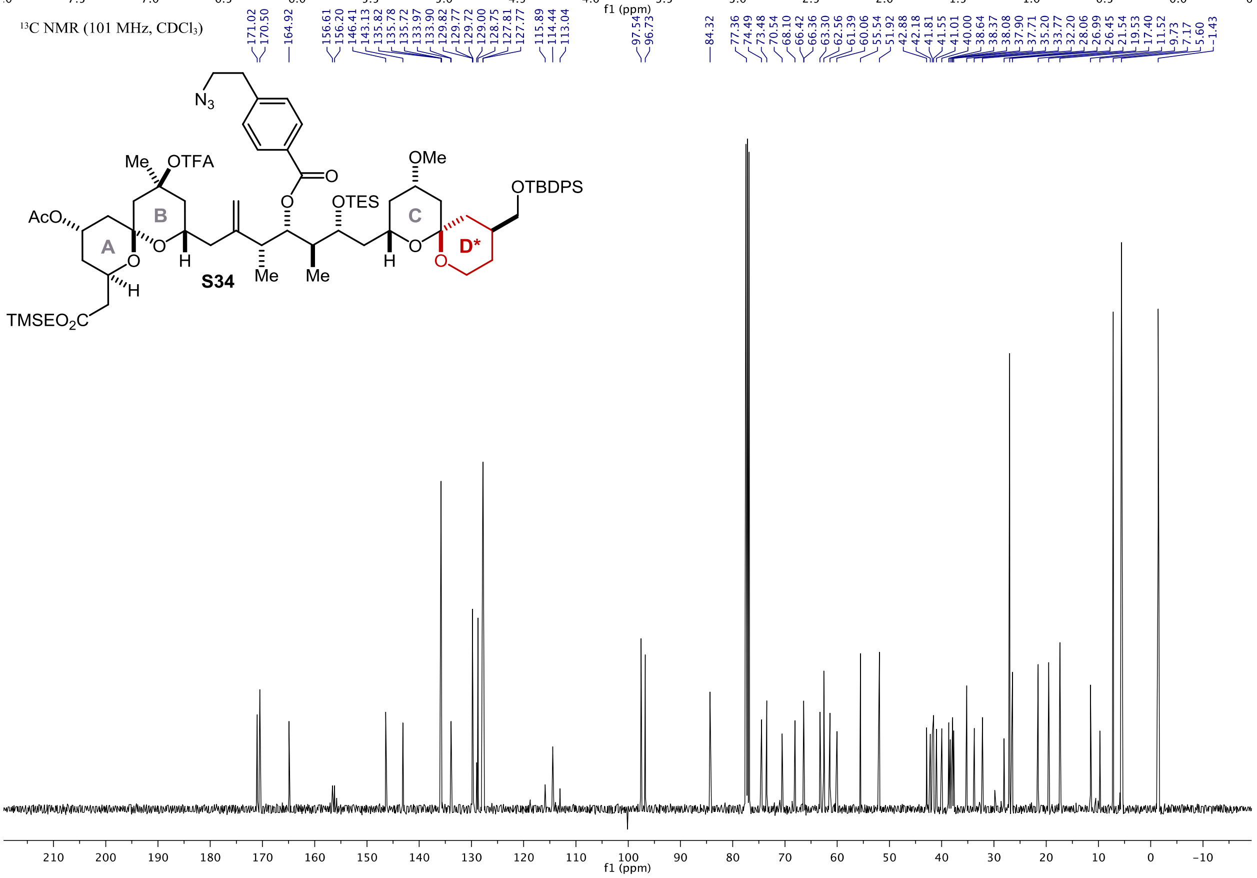

Supplementary Figure 87. ^{1}H NMR and ^{13}C NMR of **S34**.

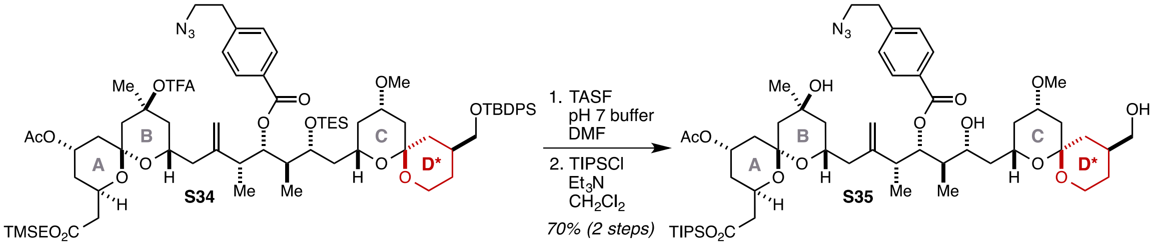

Supplementary Figure 88. Synthesis of **S35**.

To a solution of **S34** (378 mg, 0.268 mmol) in DMF (5.4 mL) was added pH 7 buffer (150 μL) followed by tris(dimethylamino)sulfonium difluorotrimethylsilicate (TASF) (740 mg, 2.69 mmol). After 23 h the reaction mixture was quenched by the addition of pH 7 buffer (10 mL). The mixture was extracted with EtOAc (5 x 5 mL) and the combined organic layers were dried over Na_2SO_4, filtered, and concentrated. Filtration through a plug of silica gel eluting with 0:100 → 10:90 MeOH:CH_2Cl_2 gave a beige foam (166 mg), which was used immediately in the next step.

To a solution of this material in CH_2Cl_2 (3.9 mL) was added Et_3N (50 μL, 0.36 mmol) and TIPSCl (45 μL, 0.21 mmol). After 45 min the reaction mixture was quenched by the addition of saturated aqueous $NaHCO_3$ (5 mL). The product was extracted with CH_2Cl_2 (3 x 2 mL) and the combined organic layers were dried over $MgSO_4$, filtered, and concentrated. The residue was purified by silica gel column chromatography eluting with 0:100 → 10:90 MeOH:CH_2Cl_2 to afford the desired product **S35** as a beige foam (190 mg, 0.187 mmol, 70% yield over 2 steps from **S34**).

OR $[\alpha]_D^{23}$ +3.8° (*c* 1.0, $CHCl_3$)

IR (thin film, cm^{-1}) 3529, 2945, 2869, 2098, 1718, 1383, 1271, 1184, 1097, 993, 757

^{1}H NMR (400 MHz, $CDCl_3$) δ 7.96 (d, *J* = 8.2 Hz, 2H), 7.29 (d, *J* = 8.2 Hz, 2H), 5.23 (dd, *J* = 8.7, 3.1 Hz, 1H), 5.06 – 4.99 (m, 1H), 4.96 (s, 1H), 4.82 (s, 1H), 4.39 – 4.27 (m, 2H), 4.09 (s, 1H), 4.07 – 3.99 (m, 1H), 3.96 – 3.84 (m, 1H), 3.67 – 3.48 (m, 5H), 3.29 – 3.19 (m, 2H), 3.25 (s, 3H), 2.94 (t, *J* = 7.0 Hz, 2H), 2.81 – 2.75 (m, 1H), 2.73 (d, *J* = 4.1 Hz, 1H), 2.65 (dd, *J* = 16.9, 7.7 Hz, 1H), 2.48 (dd, *J* = 17.0, 5.3 Hz, 1H), 2.37 (dd, *J* = 14.4, 8.2 Hz, 1H), 2.30 – 2.18 (m, 2H), 2.01 (ddd, *J* = 12.1, 4.4, 1.7 Hz, 1H), 1.98 – 1.91 (m, 1H), 1.97 (s, 3H), 1.90 – 1.50 (m, 12H), 1.47 – 1.37 (m, 1H), 1.33 – 1.08 (m, 15H), 1.05 (dd, *J* = 7.6, 1.2 Hz, 18H), 1.00 (d, *J* = 7.0 Hz, 3H)

^{13}C NMR (101 MHz, $CDCl_3$) δ 171.1, 170.4, 165.7, 147.2, 143.5, 130.0, 129.3, 129.0, 113.9, 98.3, 97.7, 77.4, 75.7, 73.1, 69.0, 68.2, 67.8, 66.2, 66.2, 64.8, 61.6, 60.2, 55.5, 52.1, 46.3, 44.1, 42.5, 41.7, 41.6, 40.7, 39.3, 38.6, 37.7, 36.9, 36.6, 35.4, 34.1, 33.2, 30.1, 28.4, 21.6, 18.0, 17.9, 13.1, 12.0, 11.7

HRMS (ESI+) calculated for $C_{53}H_{85}N_3O_{14}SiNa$ $[M+Na]^+$: 1038.5699; found 1038.5691

¹H NMR (400 MHz, CDCl₃)

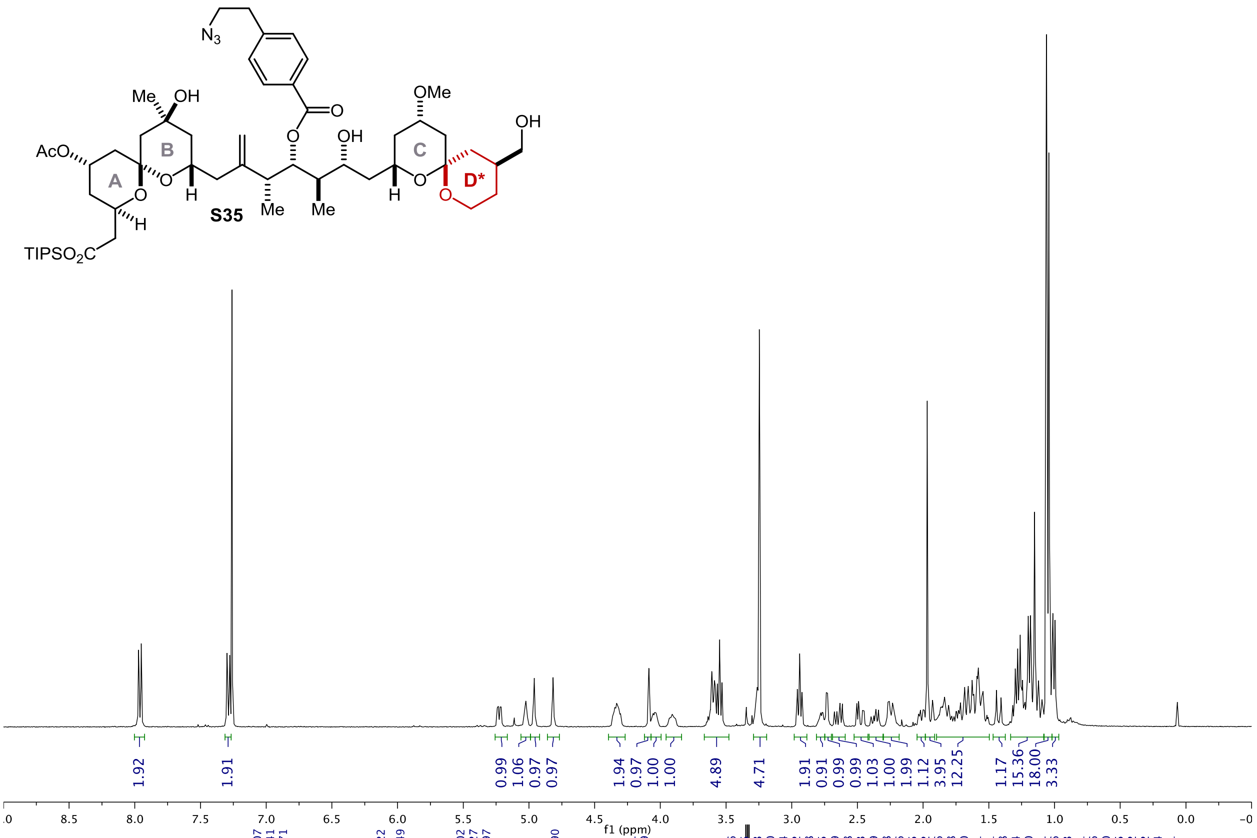

¹³C NMR (101 MHz, CDCl₃)

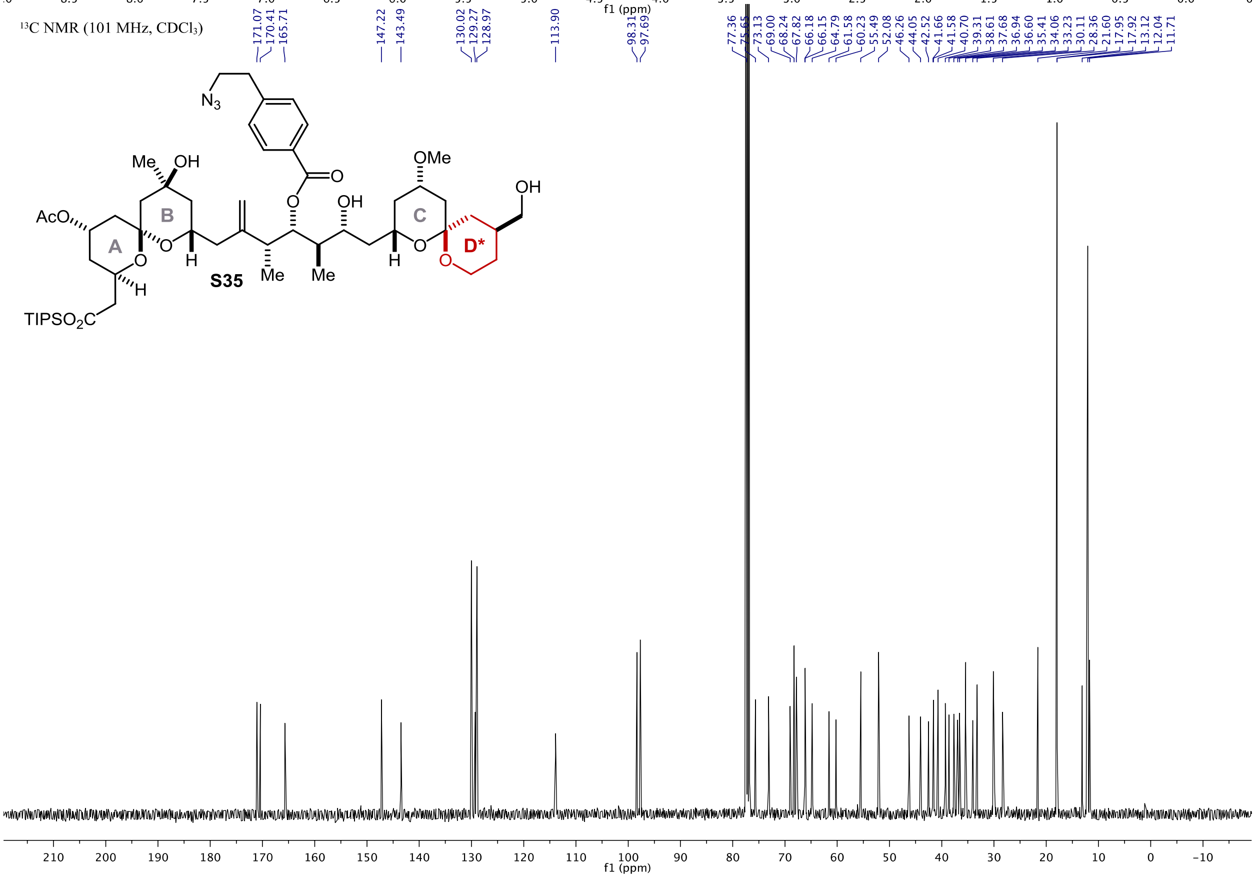

Supplementary Figure 89. ^{1}H NMR and ^{13}C NMR of **S35**.

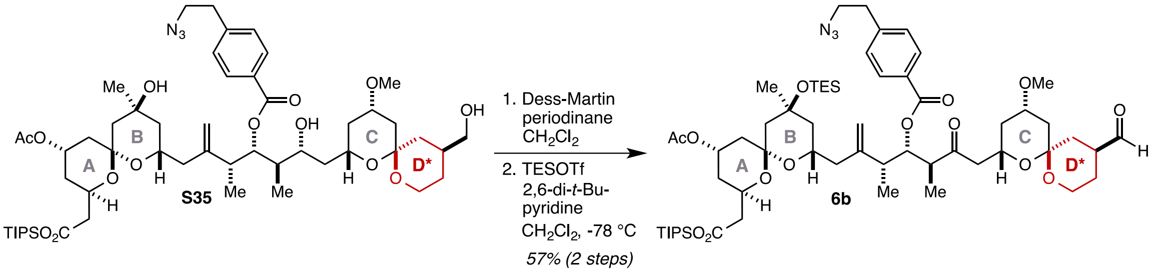

Supplementary Figure 90. Synthesis of **6b**.

To a solution of **S35** (190 mg, 0.187 mmol) in CH_2Cl_2 (12 mL) was added Dess-Martin periodinane (229 mg, 0.540 mmol). After 1.5 h the reaction mixture was cooled to 0 °C and 40 mL of a 1:1 solution of saturated aqueous $NaHCO_3$ and saturated aqueous $Na_2S_2O_3$ was added slowly. The mixture was warmed to room temperature and after 30 min the layers were separated and the aqueous layer was extracted with CH_2Cl_2 (3 x 10 mL). The combined organic layers were dried over MgSO4, filtered, and concentrated. The residue (189 mg) was used immediately in the next step without further purification.

To a solution of this product (189 mg) in CH_2Cl_2 (3.7 mL) was added 2,6-di-*tert*-butylpyridine (356 µL, 1.86 mmol). The mixture was cooled to -78 °C and TESOTf (210 µL, 0.93 mmol) was added dropwise. After 1 h the mixture was warmed to -45 °C. After 1 h excess TESOTf was quenched by the addition of saturated aqueous $NaHCO_3$ (10 mL). The mixture was warmed to room temperature and extracted with CH_2Cl_2 (3 x 10 mL). The combined organic layers were washed with saturated $CuSO_4$ (10 mL), dried over Na_2SO_4, filtered, and concentrated. The residue was purified by column chromatography using pH 7 buffered silica gel and eluting with 0:100 → 5:95 acetone:CH_2Cl_2 to afford the desired product **6b** as a beige foam (119 mg, 0.106 mmol, 57% yield over 2 steps from **S35**).

OR $[\alpha]_D^{23}$ +12.2° (*c* 1.0, $CHCl_3$)

IR (thin film, cm^{-1}) 2947, 2871, 2098, 1720, 1463, 1269, 1180, 1019, 754, 667

^{1}H NMR (500 MHz, $CDCl_3$) δ 9.48 (s, 1H), 7.91 (d, *J* = 7.8 Hz, 2H), 7.26 (m, 2H), 5.58 – 5.48 (m, 1H), 5.03 (s, 1H), 4.90 (s, 1H), 4.79 (s, 1H), 4.31 – 4.14 (m, 2H), 4.14 – 4.04 (m, 1H), 3.80 (t, *J* = 12.1 Hz, 1H), 3.68 – 3.56 (m, 2H), 3.53 (t, *J* = 7.1 Hz, 2H), 3.26 (s, 3H), 3.10 – 2.99 (m, 1H), 2.96 – 2.89 (m, 2H), 2.89 – 2.82 (m, 1H), 2.75 – 2.66 (m, 1H), 2.65 – 2.56 (m, 2H), 2.46 – 2.28 (m, 4H), 2.06 (dd, *J* = 12.6, 4.5 Hz, 1H), 2.01 – 1.92 (m, 1H), 1.95 (s, 3H) 1.90 – 1.81 (m, 2H), 1.81 – 1.70 (m, 3H), 1.65 – 1.23 (m, 15H), 1.23 – 1.11 (m, 11H), 1.07 (dd, *J* = 7.8, 2.3 Hz, 18H), 1.07 – 0.99 (m, 1H), 0.94 (t, *J* = 7.9 Hz, 9H), 0.62 – 0.50 (m, 6H)

^{13}C NMR (126 MHz, $CDCl_3$) δ 209.1, 203.0, 171.1, 170.7, 165.0, 146.6, 143.6, 130.1, 129.0, 128.9, 114.5, 97.1, 74.7, 72.7, 70.5, 66.8, 64.4, 64.1, 61.4, 59.5, 55.7, 52.1, 49.1, 48.1, 47.8, 45.4, 43.4, 42.5, 42.1, 41.3, 38.6, 38.5, 36.8, 35.4, 34.9, 34.2, 32.2, 24.9, 21.7, 17.9, 13.2, 12.5, 12.1, 7.5, 7.1

HRMS (ESI+) calculated for $C_{59}H_{95}N_3O_{14}Si_2Na$ $[M+H]^+$: 1148.6250; found 1148.6245.

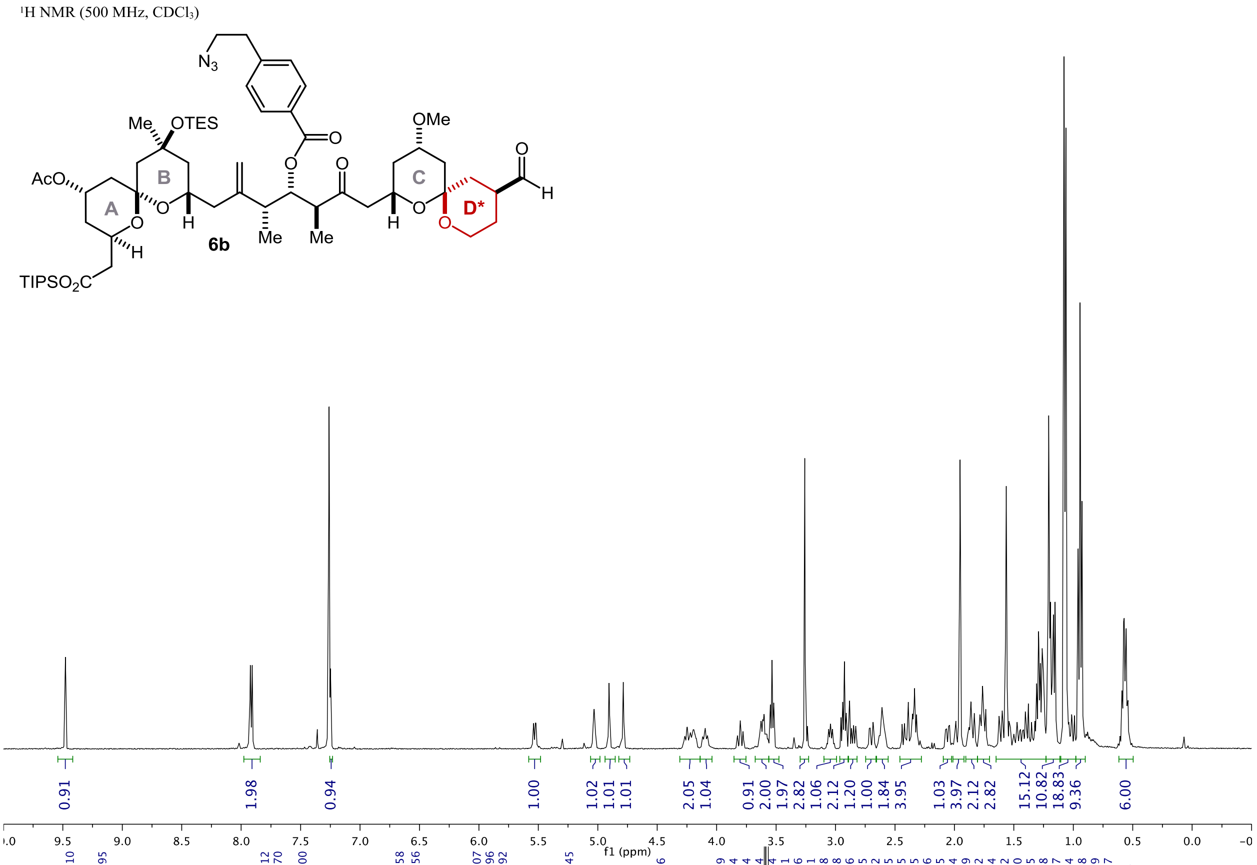

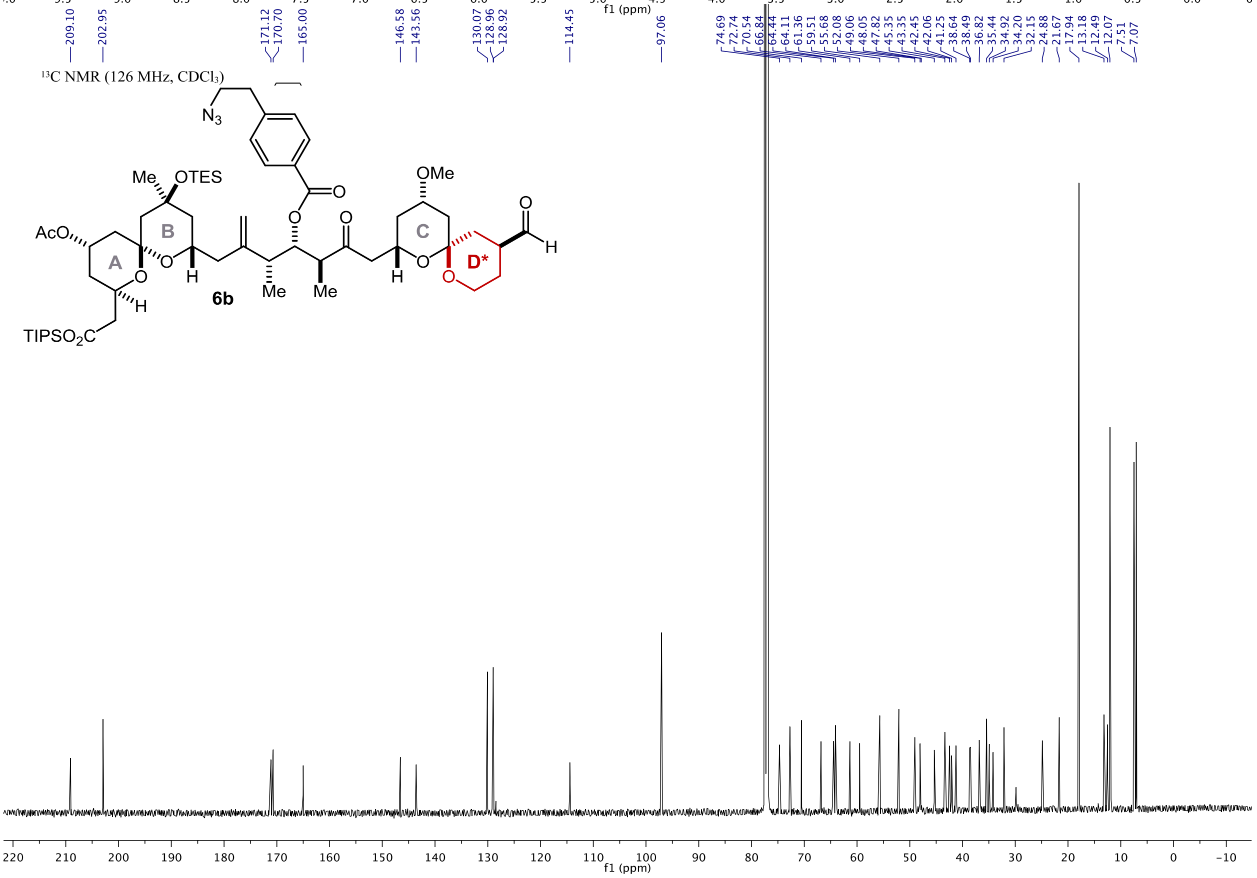

Supplementary Figure 91. ^{1}H NMR and ^{13}C NMR of **6b**.

Synthesis of 6c from 25

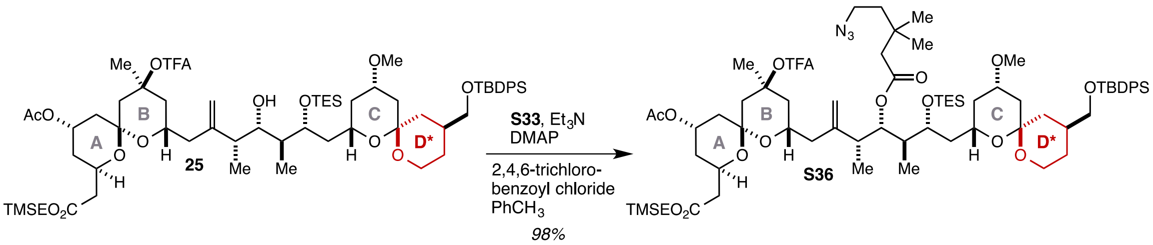

Supplemntary Figure 92. Synthesis of **S36**.

To a cooled (0 °C) solution of alcohol **25** (400 mg, 0.324 mmol) in toluene (6.5 mL) was added acid **S33** (166 mg, 0.97 mmol), Et_3N (180 µL, 1.3 mmol), DMAP (119 mg, 0.97 mmol), and 2,4,6-trichlorobenzoyl chloride (167 µL, 1.1 mmol). The reaction mixture immediately became thick and cloudy upon addition of 2,4,6-trichlorobenzoyl chloride. The mixture was warmed to room temperature. After 1.5 h the reaction mixture was quenched by the addition of saturated aqueous $NaHCO_3$ (10 mL). The mixture was extracted with EtOAc (3 x 3 mL) and the combined organic layers were dried over $MgSO_4$, filtered, and concentrated. The residue was purified by silica gel column chromatography eluting with 0:100 → 50:50 EtOAc:Hexanes to afford the desired product **S36** as a beige foam (439 mg, 0.316 mmol, 98% yield).

OR $[\alpha]_D^{23}$ +14.4° (*c* 1.0, $CHCl_3$)

IR (thin film, cm^{-1}) 2655, 2095, 1777, 1734, 1378, 1248, 1215, 1163, 1106, 1074, 999, 977, 838, 742, 702, 505

^{1}H NMR (500 MHz, $CDCl_3$) δ 7.68 – 7.58 (m, 4H), 7.46 – 7.31 (m, 6H), 5.06 – 4.97 (m, 2H), 4.97 – 4.90 (m, 1H), 4.86 (s, 1H), 4.30 – 4.19 (m, 2H), 4.18 – 4.04 (m, 2H), 3.97 (d, *J* = 10.2 Hz, 1H), 3.72 – 3.57 (m, 4H), 3.54 (dd, *J* = 10.0, 4.2 Hz, 1H), 3.39 (dd, *J* = 9.9, 6.5 Hz, 1H), 3.32 (s, 3H), 3.25 – 3.12 (m, 2H), 2.90 – 2.80 (m, 1H), 2.63 (q, *J* = 7.1 Hz, 1H), 2.43 (ddd, *J* = 19.6, 15.8, 4.2 Hz, 2H), 2.33 – 2.22 (m, 2H), 2.21 – 1.85 (m, 12H), 1.83 – 1.76 (m, 1H), 1.73 – 1.16 (m, 20H), 1.11 (d, *J* = 6.9 Hz, 3H), 1.08 – 1.03 (m, 2H), 1.05 (s, 9H), 1.02 – 0.89 (m, 21H), 0.62 (q, *J* = 7.9 Hz, 6H), 0.04 (s, 9H)

^{13}C NMR (126 MHz, $CDCl_3$) δ 171.0, 170.5, 170.5, 156.6, 156.3, 146.7, 135.8, 135.8, 133.9, 133.8, 129.8, 129.7, 127.8, 115.6, 114.3, 113.3, 97.5, 96.7, 84.3, 74.0, 73.3, 70.3, 68.5, 66.6, 66.4, 63.2, 62.6, 61.4, 60.3, 55.6, 47.7, 45.9, 42.9, 42.0, 41.9, 41.2, 41.0, 40.0, 39.6, 38.6, 38.5, 37.9, 37.7, 37.6, 36.8, 33.8, 32.8, 32.0, 28.7, 27.7, 27.3, 27.1, 26.4, 24.8, 23.5, 21.6, 19.5, 17.4, 11.3, 10.2, 7.2, 5.6, -1.4

HRMS (ESI+) calculated for $C_{71}H_{112}N_3O_{15}F_3Si_3Na$ $[M+Na]^+$: 1410.7251; found 1410.7235

^{1}H NMR (500 MHz, $CDCl_3$)

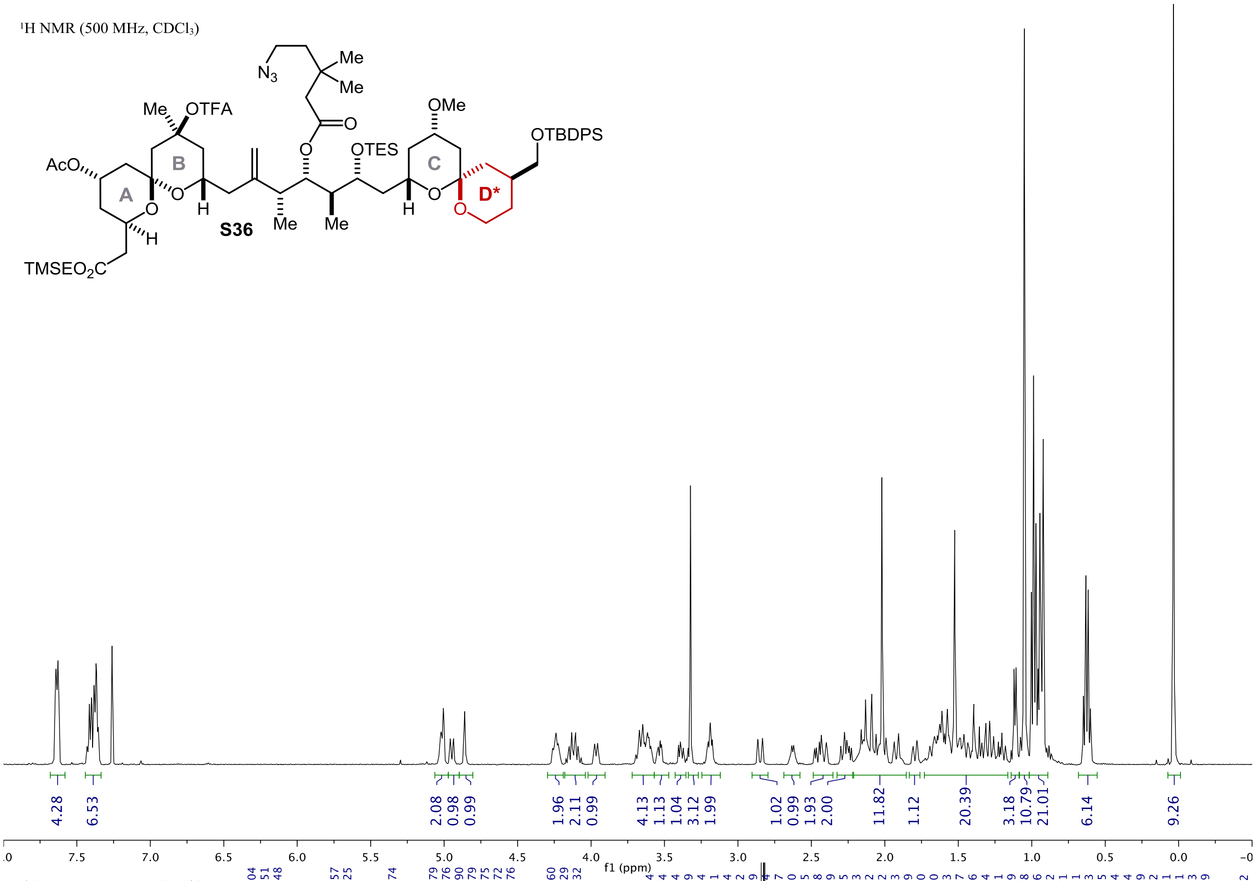

^{13}C NMR (126 MHz, $CDCl_3$)

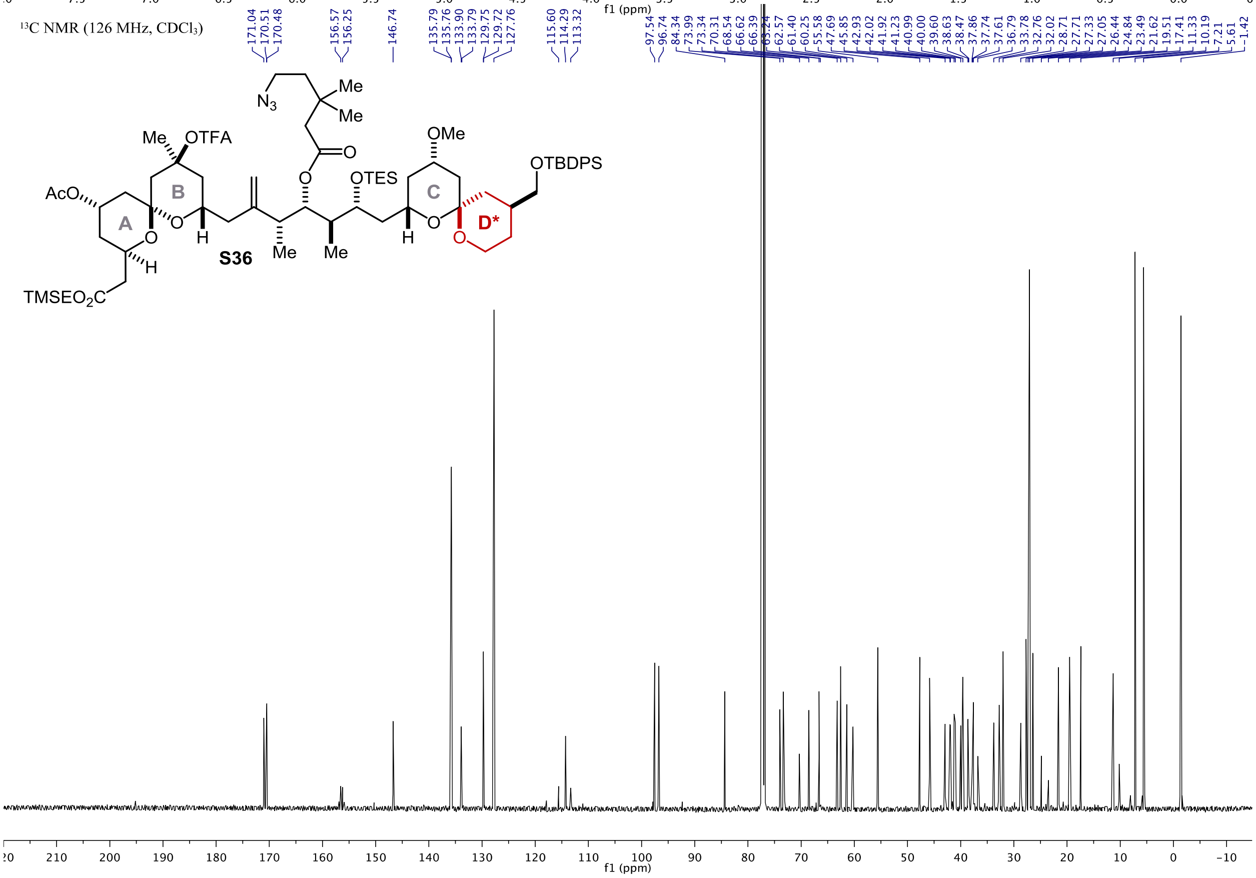

Supplementary Figure 93. ^{1}H NMR and ^{13}C NMR of **S36**.

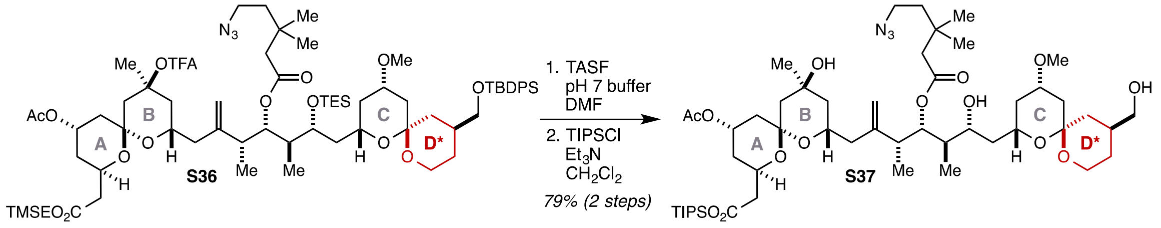

Supplementary Figure 94. Synthesis of **S37**.

To a solution of **S36** (439 mg, 0.316 mmol) in DMF (6.3 mL) was added pH 7 buffer (0.17 mL) followed by tris(dimethylamino)sulfonium difluorotrimethylsilicate (TASF) (0.87 g, 3.2 mmol). After 23 h the reaction mixture was quenched by the addition of pH 7 buffer (10 mL). The mixture was extracted with EtOAc (5 x 5 mL) and the combined organic layers were dried over Na_2SO_4, filtered, and concentrated. Filtration through a plug of silica gel eluting with 0:100 → 10:90 MeOH:CH_2Cl_2 gave a beige foam (228 mg), which was used immediately in the next step.

To a solution of this material in CH_2Cl_2 (5.4 mL) was added Et_3N (70 μL, 0.50 mmol) and TIPSCl (64 μL, 0.30 mmol). After 45 min the reaction mixture was quenched by the addition of saturated aqueous $NaHCO_3$ (6 mL). The product was extracted with CH_2Cl_2 (3 x 3 mL) and the combined organic layers were dried over $MgSO_4$, filtered, and concentrated. The residue was purified by silica gel column chromatography eluting with 0:100 → 6:94 MeOH:CH_2Cl_2 to afford the desired product **S37** as a beige foam (250 mg, 0.251 mmol, 79% yield over 2 steps from **S34**).

OR $[\alpha]_D^{22}$ +18.1° (*c* 1.0, $CHCl_3$)

IR (thin film, cm^{-1}) 3485, 2945, 2869, 2095, 1718, 1552, 1464, 1370, 1324, 1184, 1091, 1053, 993, 884, 754, 667

^{1}H NMR (500 MHz, $CDCl_3$) δ 5.08 – 5.01 (m, 3H), 4.87 (s, 1H), 4.39 – 4.26 (m, 2H), 4.12 (s, 1H), 4.02 – 3.88 (m, 2H), 3.71 – 3.59 (m, 3H), 3.41 (s, 2H), 3.36 – 3.30 (m, 4H), 3.28 (t, *J* = 8.0 Hz, 2H), 3.08 – 2.99 (m, 1H), 2.68 (q, *J* = 7.7 Hz, 2H), 2.48 (dd, *J* = 17.1, 5.5 Hz, 1H), 2.33 (dd, *J* = 14.4, 8.5 Hz, 1H), 2.26 – 2.17 (m, 1H), 2.20 (s, 2H), 2.14 – 1.87 (m, 7H), 2.04 (s, 3H), 1.87 – 1.74 (m, 2H), 1.74 – 1.48 (m, 11H), 1.42 (d, *J* = 14.0 Hz, 1H), 1.34 – 1.16 (m, 10H), 1.15 (s, 3H), 1.10 – 0.99 (m, 30H), 0.92 (d, *J* = 7.0 Hz, 3H)

^{13}C NMR (126 MHz, $CDCl_3$) δ 171.2, 171.1, 170.5, 147.6, 113.7, 98.3, 97.9, 74.9, 73.1, 69.3, 68.3, 67.8, 66.5, 66.2, 64.6, 61.6, 60.4, 55.7, 47.7, 46.2, 45.8, 44.0, 42.5, 41.7, 41.4, 40.4, 39.7, 38.8, 38.7, 37.6, 36.7, 34.0, 33.3, 32.2, 30.0, 28.3, 27.6, 27.5, 21.7, 18.0, 17.9, 13.2, 12.0, 11.9

HRMS (ESI+) calculated for $C_{51}H_{89}N_3O_{14}SiNa$ $[M+Na]^+$: 1018.6012; found 1018.6011

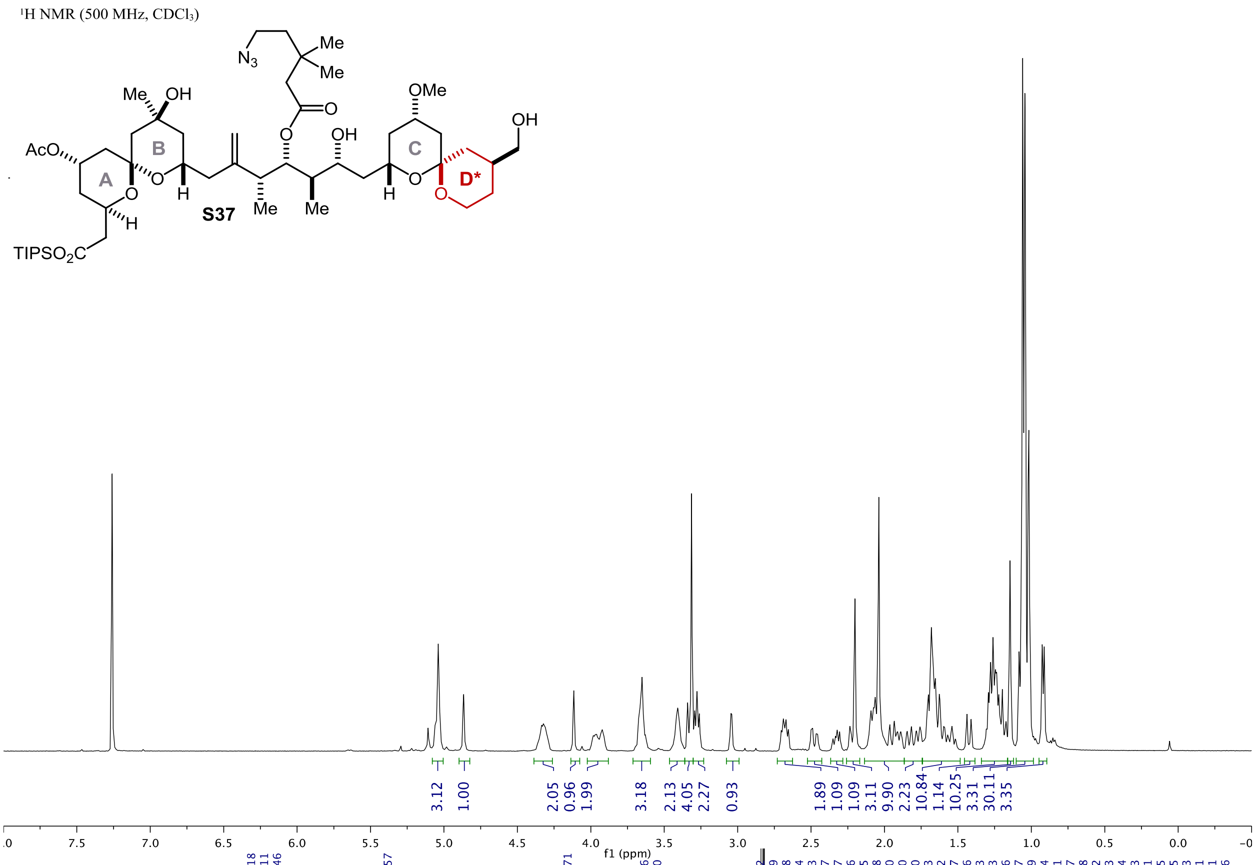

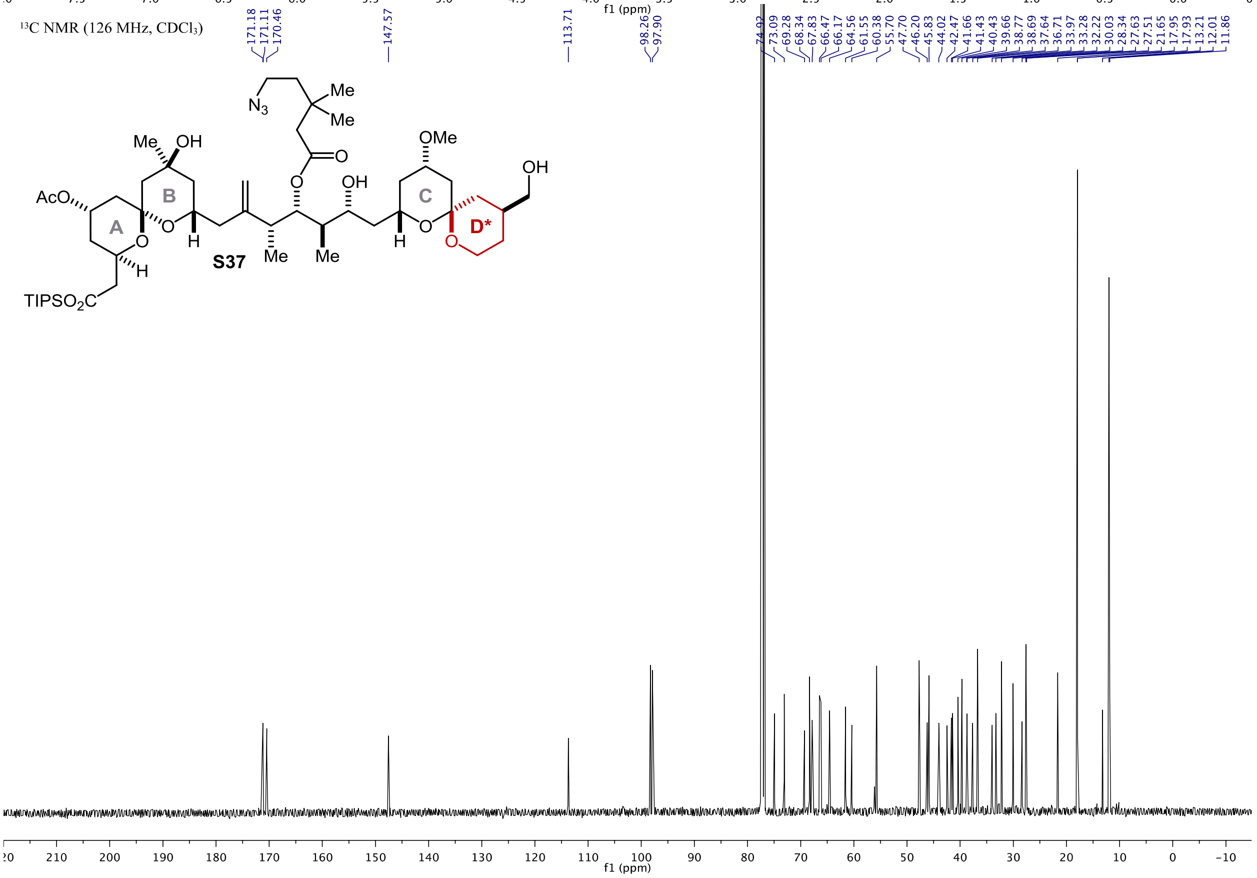

Supplementary Figure 95. ^{1}H NMR and ^{13}C NMR of **S37**.

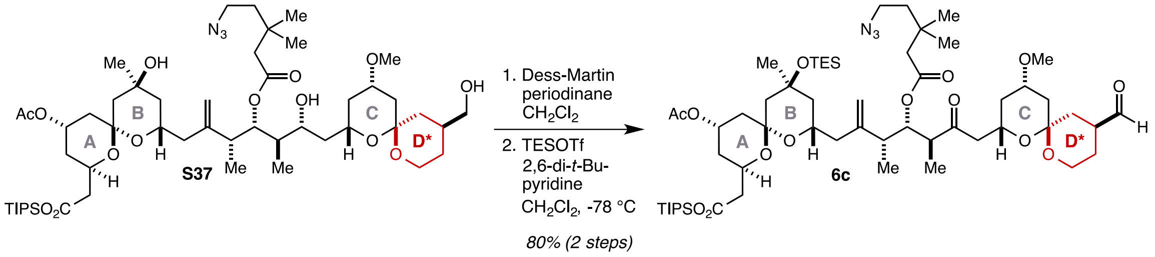

Supplementary Figure 96. Synthesis of **6c**.

To a solution of **S37** (154 mg, 0.154 mmol) in CH_2Cl_2 (10 mL) was added Dess-Martin periodinane (190 mg, 0.447 mmol). After 1.5 h the reaction mixture was cooled to 0 °C and 35 mL of a 1:1 solution of saturated aqueous $NaHCO_3$ and saturated aqueous $Na_2S_2O_3$ was added slowly. The mixture was warmed to room temperature and after 30 min the layers were separated and the aqueous layer was extracted with CH_2Cl_2 (3 x 10 mL). The combined organic layers were dried over MgSO4, filtered, and concentrated. The residue (152 mg) was used immediately in the next step without further purification.

To a solution of this product (152 mg) in CH_2Cl_2 (3.1 mL) was added 2,6-di-*tert*-butylpyridine (333 µL, 1.54 mmol). The mixture was cooled to -78 °C and TESOTf (174 µL, 0.769 mmol) was added dropwise. After 1 h the mixture was warmed to -45 °C. After 1 h excess TESOTf was quenched by the addition of saturated aqueous $NaHCO_3$ (10 mL). The mixture was warmed to room temperature and extracted with CH_2Cl_2 (3 x 10 mL). The combined organic layers were washed with saturated aqueous $CuSO_4$ (10 mL), dried over Na_2SO_4, filtered, and concentrated. The residue was purified by column chromatorgraphy using pH 7 buffered silica gel and eluting with 0:100 → 8:92 acetone:CH_2Cl_2 to afford the desired product **6c** as a beige foam (136 mg, 0.123 mmol, 80% yield over 2 steps from **S37**).

OR $[\alpha]_D^{22}$ +11.4° (*c* 0.5, $CHCl_3$)

IR (thin film, cm^{-1}) 2947, 2871, 2095, 1725, 1463, 1370, 1214, 1181, 1145, 1065, 994, 755, 667

^{1}H NMR (500 MHz, $CDCl_3$) δ 9.56 (s, 1H), 5.42 – 5.31 (m, 1H), 5.04 (s, 1H), 4.98 (s, 1H), 4.82 (s, 1H), 4.27 – 4.09 (m, 3H), 3.83 (t, *J* = 12.0 Hz, 1H), 3.74 – 3.62 (m, 2H), 3.33 (s, 3H), 3.26 (t, *J* = 8.0 Hz, 2H), 2.92 – 2.77 (m, 2H), 2.74 – 2.61 (m, 2H), 2.52 – 2.44 (m, 1H), 2.44 – 2.21 (m, 4H), 2.16 – 2.06 (m, 3H), 2.04 – 1.95 (m, 5H), 1.90 – 1.79 (m, 3H), 1.78 – 1.70 (m, 1H), 1.65 – 1.52 (m, 7H), 1.51 – 1.39 (m, 3H), 1.36 – 1.17 (m, 11H), 1.08 (t, *J* = 6.0 Hz, 25H), 0.97 (s, 6H), 0.92 (t, *J* = 7.8 Hz, 9H), 0.59 – 0.49 (m, 6H).

^{13}C NMR (126 MHz, $CDCl_3$) δ 208.8, 203.0, 171.1, 170.7, 170.2, 147.0, 114.1, 97.1, 97.0, 73.3, 72.8, 70.5, 66.8, 64.3, 64.2, 61.3, 59.6, 55.8, 48.9, 48.4, 47.8, 47.7, 45.8, 45.3, 43.4, 42.4, 42.1, 41.2, 39.7, 38.6, 37.6, 37.0, 35.0, 34.1, 32.3, 32.1, 27.5, 27.5, 24.9, 21.7, 17.9, 12.9, 12.1, 12.0, 7.5, 7.0.

HRMS (ESI+) calculated for $C_{57}H_{99}N_3O_{14}Si_2Na$ $[M+Na]^+$: 1128.6563; found 1128.6573

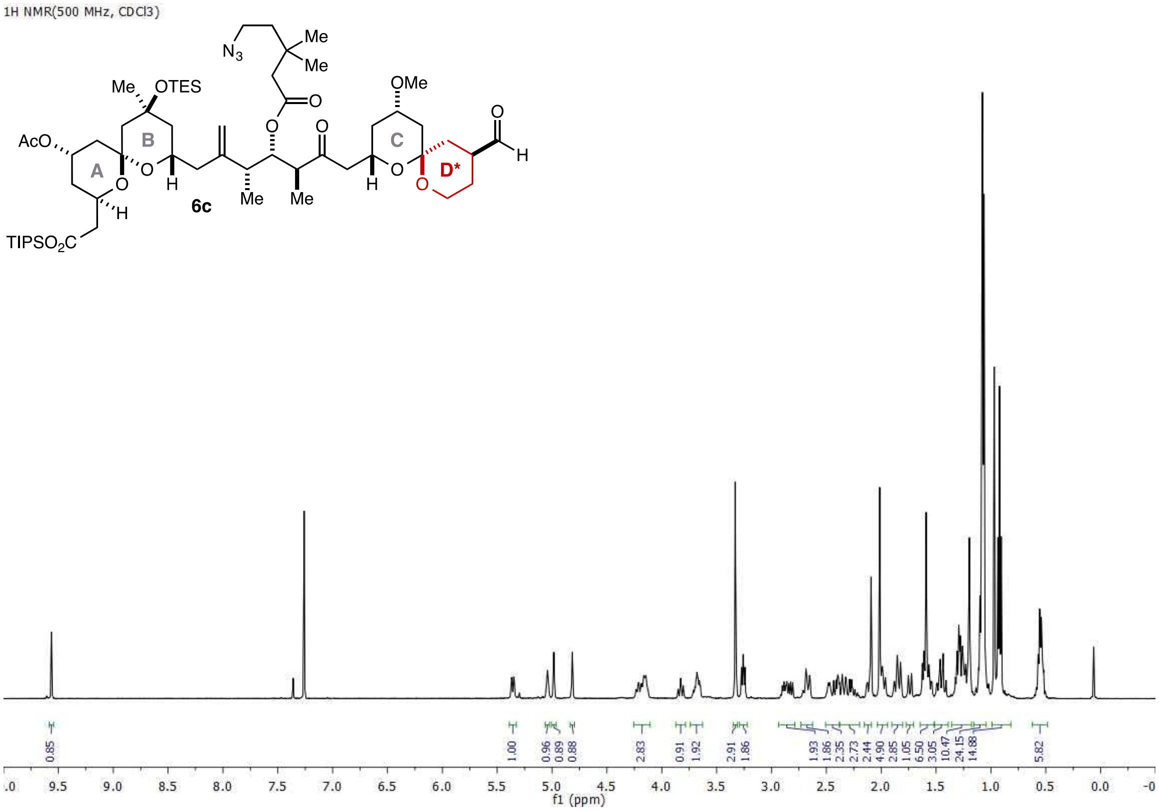

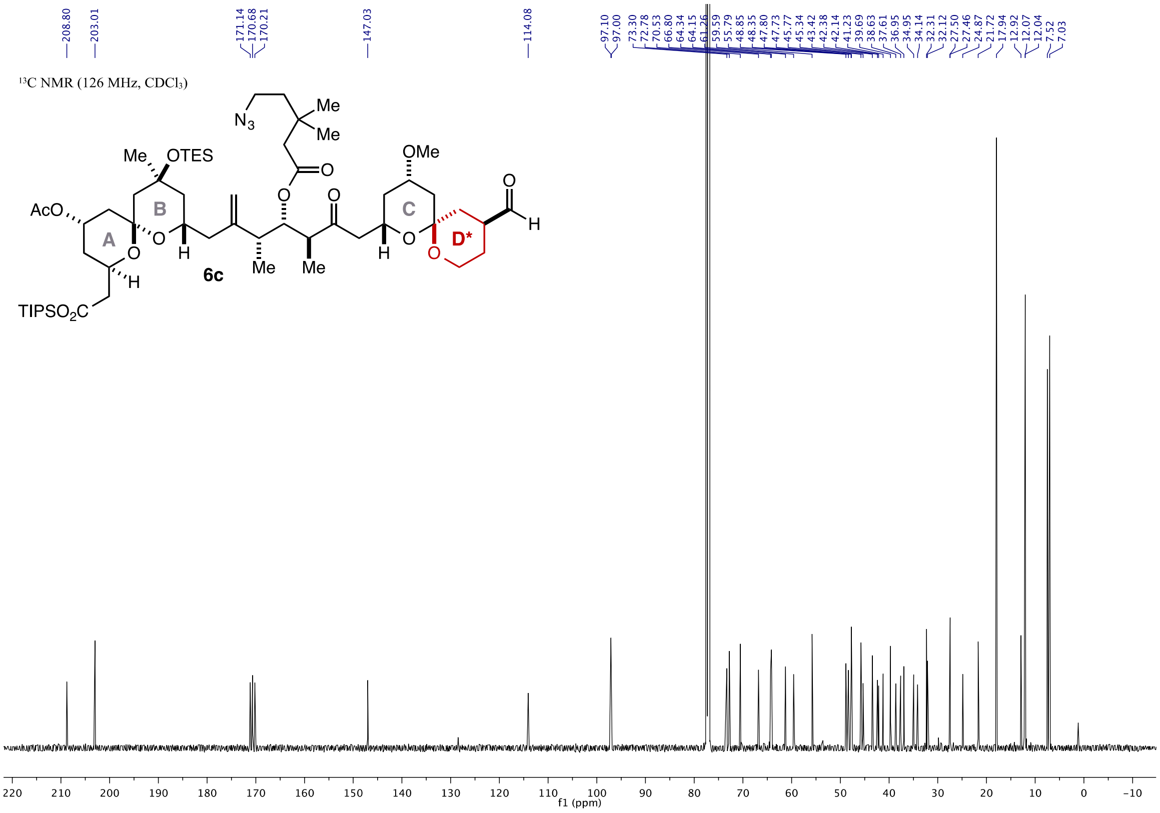

Supplementary Figure 97. ^{1}H NMR and ^{13}C NMR of **6c**.

Synthesis of **33**

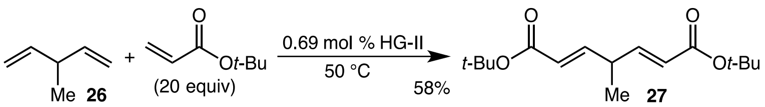

Supplementary Figure 98. Synthesis of **27**.

To a solution of 3-methyl-1,4-pentadiene **26** (5.62 mL, 45 mmol, 1 equiv) in *t*-butyl acrylate (135 ml, 0.9 mol, 20 equiv) was added Hoveyda-Grubbs 2nd Generation catalyst (65.6 mg, 0.105 mmol, 0.23 mol %). The flask was fitted with a dry-ice condenser under constant nitrogen flow and the reaction mixture was heated to 50 °C. After 1.5 h, an additional portion of the HG-II catalyst (65.6 mg, 0.105 mmol, 0.23 mol %) was added. After 1.5 h, an additional portion of the HG-II catalyst (65.6 mg, 0.105 mmol, 0.23 mol %) was added. After 2 h, the reaction mixture was cooled to ambient temperature and concentrated. The residue was treated with toluene (100 mL) and the mixture was concentrated. The residue was purified by silica gel flash column chromatography (4% EtOAc/Hexanes) to yield dienoate **27** (7.55 g, 26.7 mmol, 58%) as a pale yellow oil. Dienoate **27** was previously characterized,[13] and the data are reproduced here.

IR (thin film) 2977, 2933, 1713, 1614, 1392, 1318, 1153 cm^{-1}

^{1}H NMR (500 MHz, $CDCl_3$) δ 6.76 (dd, J = 15.7, 7.0 Hz, 2H), 5.73 (dd, J = 15.7, 1.3 Hz, 2H), 3.17-3.08 (m, 1H), 1.47 (s, 18H), 1.20 (d, J = 6.9 Hz, 3H)

^{13}C NMR (126 MHz, $CDCl_3$) δ 165.9, 148.4, 123.2, 80.6, 38.5, 28.3, 18.6

HRMS (FAB+) calculated for $C_{16}H_{27}O_4$ $[M+H]^+$: 283.1904, found 283.1910.

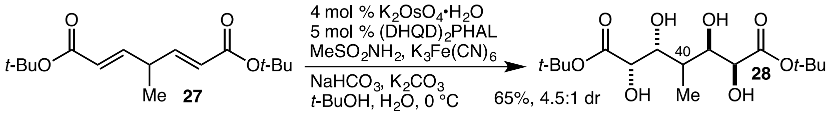

Supplementary Figure 99. Synthesis of **28**.

To a mechanically-stirred solution of $K_3Fe(CN)_6$ (148 g, 450 mmol, 6 equiv), K_2CO_3 (62.1 g, 450 mmol, 6 equiv), $NaHCO_3$ (37.8 g, 450 mmol, 6 equiv) and $CH_3SO_2NH_2$ (14.3 g, 150 mmol, 2 equiv) in H2 O (750 mL) was added a solution of $(DHQD)_2PHAL$ (3.00 g, 3.85 mmol, 0.05 equiv) in *t*-BuOH (500 mL). The mixture was cooled to 0 °C and K_2OsO_4 •$2H_2O$ (1.10 g, 3.00 mmol, 0.04 equiv) was added, followed 10 minutes later by a solution of dienoate **27** (21.2 g, 74.9 mmol) in *t*-BuOH (250 mL). The bright orange suspension was stirred vigorously at 0 °C for 18 h. Solid Na_2SO_3 (94 g, 749 mmol, 10 equiv) was added and the reaction mixture was stirred for a further 45 min. The layers of the cold reaction mixture were separated, and the aqueous layer was extracted with EtOAc (3 x 150 mL). The combined organic layers were stirred with solid NaCl, the resulting brine layer was removed. The organic phase was washed with additional brine (350 mL), dried over Na_2SO_4, filtered and concentrated. The residue was purified by silica gel flash column chromatography (50% EtOAc/Hexanes) to yield tetraol **28** (16.4 g, 46.7 mmol, 62%) as the major product of a 4.5:1 mixture of diastereomers as a colorless gum that solidified to a colorless solid on standing. This mixture was used as is in the next step, but for the purposes of characterization, an analytically pure sample was obtained by careful flash chromatography. Tetraol **28** was previously characterized,[13] and the data are reproduced here.

OR $[\alpha]_D^{21}$ -12.7° (*c* 0.56, $CHCl_3$)

IR (thin film) 3473, 2978, 2934, 1716, 1394, 1288 cm^{-1}

^{1}H NMR (400 MHz, $CDCl_3$) δ 4.15 (dd, J = 5.7, 3.0 Hz, 1H), 4.12 (dd, J = 5.7, 1.6 Hz, 1H), 4.06 (dt, J = 8.4, 3.3 Hz, 1H), 3.99 (td, J = 8.9, 1.3 Hz, 1H), 3.55 (d, J = 8.5 Hz, 1H), 3.52 (d, J = 5.7 Hz, 1H), 3.34 (d, J = 5.7 Hz, 1H), 3.22 (d, J = 9.1 Hz, 1H), 2.26-2.14 (m, 1H), 1.50 (s, 18H), 1.07 (d, J = 7.0 Hz, 3H)

^{13}C NMR (75 MHz, $CDCl_3$) δ 173.0, 83.5, 83.3, 75.0, 74.2, 72.2, 71.9, 40.0, 28.2, 13.1

HRMS (FAB+) calcd for $[C_{16}H_{30}O_8H+]$ requires *m/z* 351.2013, found 351.2018

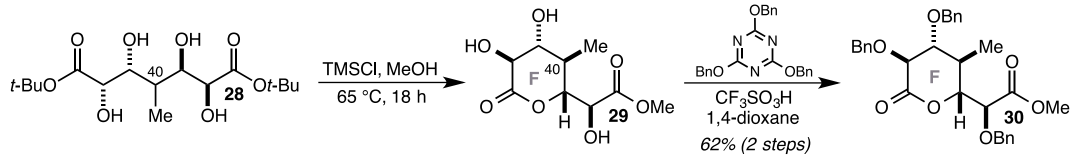

Supplementary Figure 100. Synthesis of **30**.

To a solution of tetraol **28** (20.83 g, 59.40 mmol) in methanol (300 mL) in a sealed-tube pressure apparatus was added TMS-Cl (37.7 mL, 297 mmol). The vessel was sealed and the reaction mixture was heated at 65 °C (oil bath, external temperature) for 18 h. [***CAUTION!!! Always use a blast shield and proper personal protective equipment when running reactions in sealed tubes***.] The reaction mixture was cooled and concentrated. The residue was re-dissolved in toluene (150 mL) and then concentrated.

To the residue was added TriBOT[14] (18.96 g, 47.52 mmol), flame-dried 5Å mol. sieves (14.36 g), 1,4-dioxane (585 mL), and triflic acid (3.93 mL, 44.55 mmol). After 1 h an additional portion of TriBOT (14.22 g, 35.64 mmol) was added. After 4 h an additional portion of TriBOT (14.22 g, 35.64 mmol) was added. After 18 h 1M NaOH (45 mL) was added and the mixture was stirred for 10 minutes. The reaction mixture was diluted with CH_2Cl_2 (450 mL) and filtered through Celite. The filtrate was washed with 1M NaOH (2 x 100 mL) and brine (150 mL), dried over Na_2SO_4, filtered, and concentrated. Purification of the residue by flash column chromatography (5% → 10% → 20% → 30% EtOAc/Hexanes) on silica gel gave lactone **30** as a pale yellow oil (18.6 g, 36.8 mmol, 62% yield over two steps) as well as a mix of doubly benzylated lactones (1.9 g, 4.58 mmol, 9% yield).

OR $[\alpha]_D^{23}$ –43.6° (*c* 1.00, CH_2Cl_2)

IR (ATR) 3029, 2876, 1754, 1496, 1453, 1286, 1210 cm^{-1}

^{1}H NMR (500 MHz, $CDCl_3$) δ 7.56 – 7.17 (m, 20H), 5.06 (d, J = 11.2 Hz, 1H), 4.94 (d, J = 11.7 Hz, 1H), 4.80 (d, J = 11.1 Hz, 1H), 4.66 (d, J = 11.2 Hz, 1H), 4.52 (d, J = 11.2 Hz, 1H), 4.42 (d, J = 11.7 Hz, 1H), 4.38 (dd, J = 10.4, 2.2 Hz, 1H), 4.05 (d, J = 7.9 Hz, 1H), 4.03 (d, J = 2.2 Hz, 1H), 3.85 (s, 3H), 3.44 (dd, J = 9.8, 7.8 Hz, 1H), 2.33 – 2.22 (m, 1H), 0.78 (d, J = 6.6 Hz, 3H)

^{13}C NMR (126 MHz, $CDCl_3$) δ 170.1, 169.7, 137.8, 137.3, 136.5, 128.9, 128.7, 128.7, 128.6, 128.6, 128.5, 128.5, 128.3, 128.2, 128.1, 81.9, 80.4, 79.9, 75.4, 74.5, 74.4, 73.2, 52.7, 35.7, 13.5

HRMS (ESI+) calculated for $C_{62}H_{82}O_9SiNa$ $[M+Na]^+$: 527.2046, found 527.2041

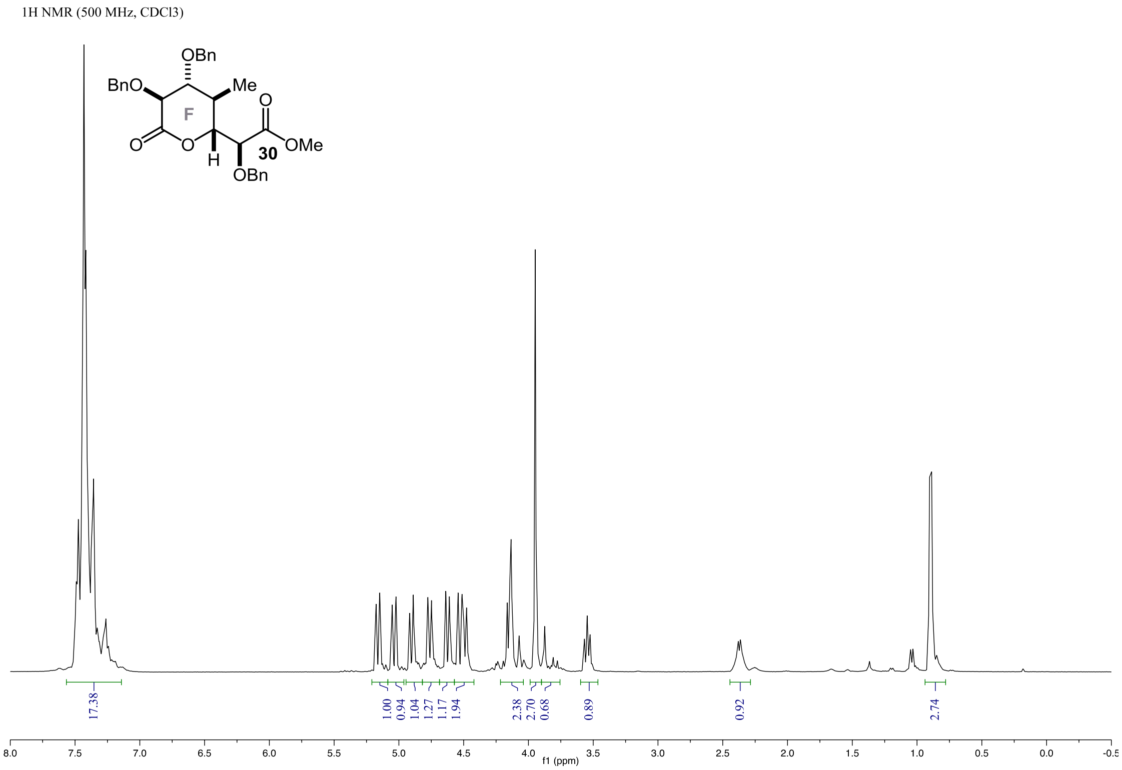

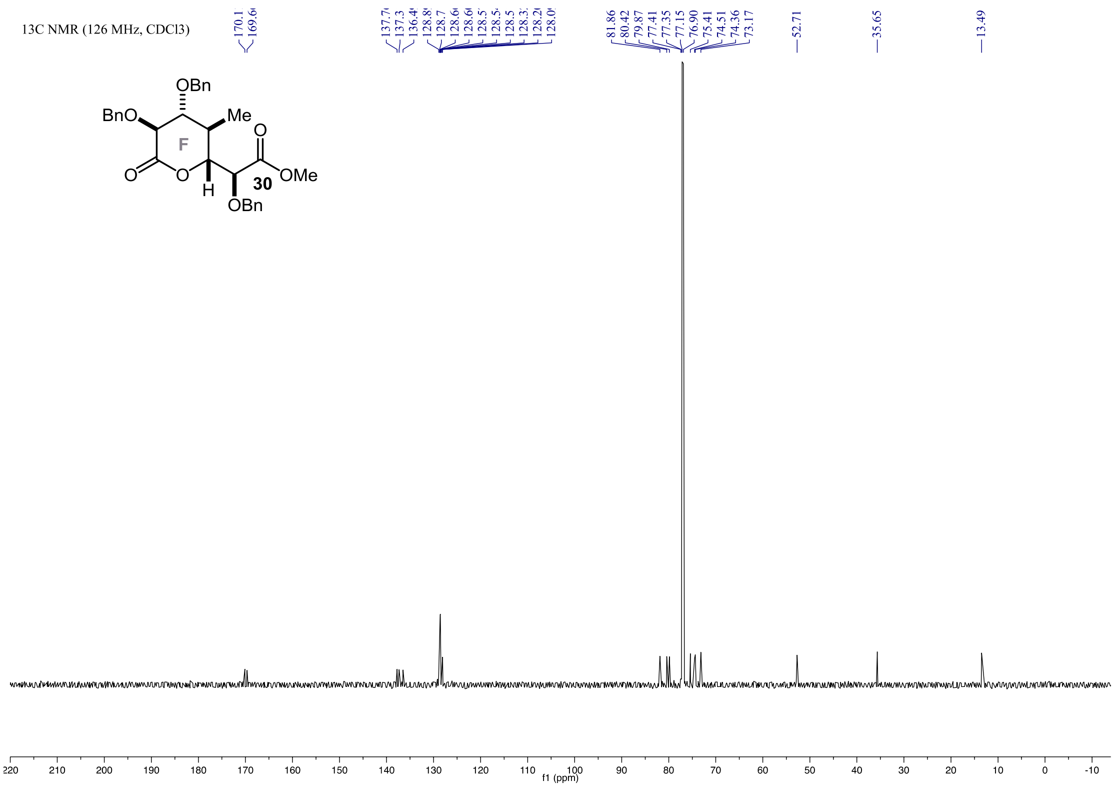

Supplementary Figure 101. ^{1}H NMR and ^{13}C NMR of **30**.

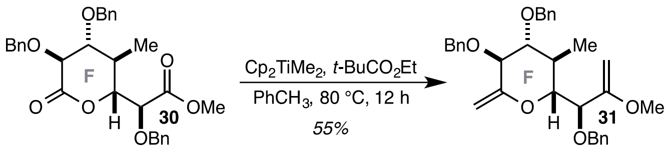

Supplementary Figure 102. Synthesis of **31**.

To a solution of lactone **30** (918 mg, 1.82 mmol) in toluene (15 mL) in a sealed-tube pressure apparatus was added Cp_2TiMe_2 (5.77 g of a 24% w/w solution in toluene, 6.38 mmol) and ethyl pivalate (0.14 mL, 0.91 mmol). The pressure apparatus was sealed and heated at 80 °C (oil bath, external temperature) for 12 h. [***CAUTION!!! Always use a blast shield and proper personal protective equipment when running reactions in sealed tubes***.] The reaction mixture was cooled to room temperature and hexane (100 mL) was added to precipitate the titanocene oxide byproduct. The reaction mixture was filtered through a pad of Celite and the filtrate was concentrated. Purification of the residue by silica gel flash column chromatography 0% → 10% EtOAc/Hexanes gave enol ether **31** as a yellow oil (501 mg, 1.00 mmol, 55% yield).

OR $[\alpha]_D^{23}$ –17.7° (*c* 1.00, CH_2Cl_2)

IR (ATR) 3437, 3029, 2924, 2874, 1722, 1659, 1599, 1495, 1453, 1355, 1263, 1101, 1066, 814, 736, 699 cm^{-1}

^{1}H NMR (500 MHz, $CDCl_3$) δ 7.41 – 7.27 (m, 15H), 4.91 (d, J = 11.1 Hz, 1H), 4.78 (dd, J = 14.0, 11.6 Hz, 2H), 4.65 (s, 2H), 4.58 (d, J = 11.1 Hz, 1H), 4.43 (d, J = 2.5 Hz, 1H), 4.38 (d, J = 11.9 Hz, 1H), 4.28 (d, J = 2.5 Hz, 1H), 3.98 (d, J = 8.1 Hz, 1H), 3.86 (d, J = 2.1 Hz, 1H), 3.64 (s, 3H), 3.45 (dd, J = 10.4, 2.1 Hz, 1H), 3.19 (dd, J = 9.9, 8.1 Hz, 1H), 2.24 – 2.09 (m, 1H), 0.70 (d, J = 6.6 Hz, 3H)

^{13}C NMR (126 MHz, $CDCl_3$) δ 159.4, 158.2, 138.6, 138.3, 137.8, 128.7, 128.5, 128.5, 128.4, 128.3, 128.1, 128.0, 127.8, 127.8, 94.3, 84.7 82.6, 82.3, 81.4, 76.5, 75.0, 73.1, 72.2, 55.1, 37.6, 12.9

HRMS (ESI+) calculated for $C_{32}H_{37}O_5$ $[M+H]^+$: 501.2641, found 501.2642

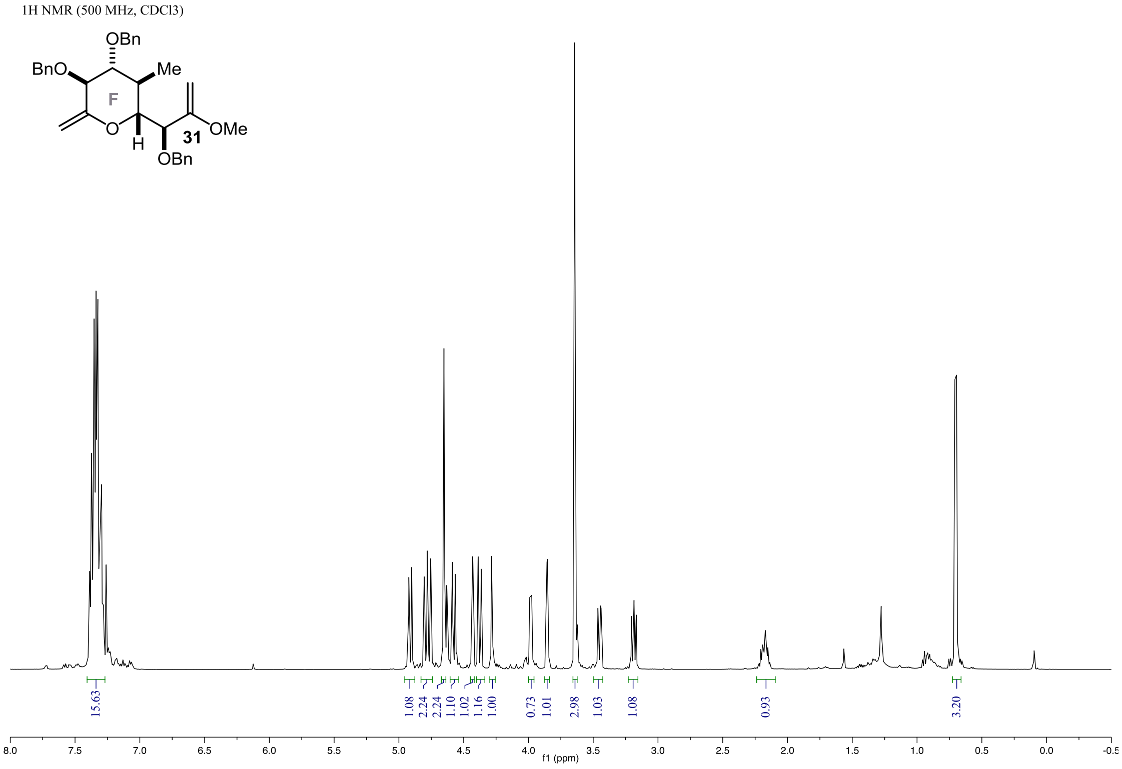

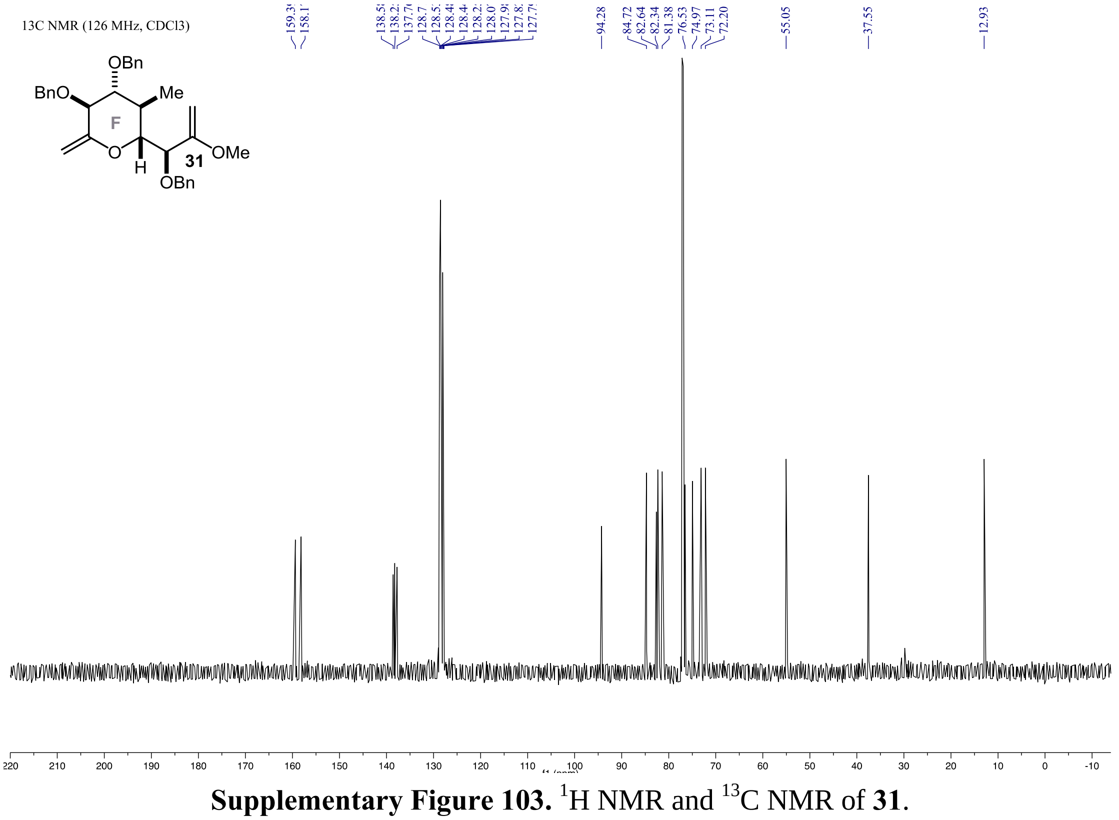

Supplementary Figure 103. ^{1}H NMR and ^{13}C NMR of **31**.

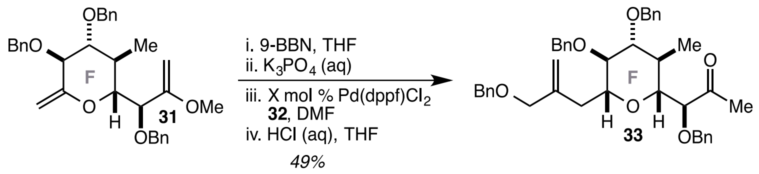

Supplementary Figure 104. Synthesis of **33**.

To a cooled (0 °C) solution of enol ether **31** (1.70 g, 3.40 mmol) in THF (19 mL) was added 9-BBN (13.6 mL, 0.5 M in THF). After 6 h 3 M K_3PO_4 (3.4 mL, degassed) was added. After 20 min, a pre-prepared separate solution of $Pd(dppf)Cl_2•CH_2Cl_2$ (277 mg, 0.34 mmol) and vinyl bromide **32**[15] (2.32 g, 10.2 mmol) in degassed DMF (34 mL) was added dropwise. After 12 h, saturated aqueous $NaHCO_3$ (15 mL) and H_2O (150 mL) were added. The resultant mixture was extracted with Et_2O (3 x 50 mL). The combined organic layers were concentrated. The residue was dissolved in THF (85 mL) and 1M HCl (5 mL) was added. After 1 h saturated aqueous $NaHCO_3$ (80 mL) was added. The layers were separated and the aqueous layer was extracted with EtOAc (3 x 50 mL). The combined organic layers were dried over Na_2SO_4, filtered and concentrated. Purification of the residue by flash column chromatography (5% → 10% EtOAc/Hexanes) on silica gel gave methyl ketone **33** as a clear oil (1.06 g, 1.67 mmol, 49% yield).

OR $[\alpha]_D^{22}$ –11.7° (*c* 1.00, CH_2Cl_2)

IR (ATR) 3029, 2853, 1711, 1495, 1453, 1352, 1072 cm^{-1}

^{1}H NMR (500 MHz, $CDCl_3$) δ 7.41 – 7.20 (m, 20H), 5.07 (s, 1H), 4.94 – 4.83 (m, 3H), 4.78 (d, J = 11.9 Hz, 1H), 4.65 (d, J = 11.0 Hz, 1H), 4.60 (d, J = 10.9 Hz, 1H), 4.48 (d, J = 11.9 Hz, 1H), 4.42 (d, J = 11.9 Hz, 1H), 4.31 (d, J = 11.8 Hz, 1H), 3.99 – 3.85 (m, 2H), 3.75 (s, 1H), 3.37 – 3.23 (m, 3H), 3.17 (t, J = 9.5 Hz, 1H), 2.69 (d, J = 15.0 Hz, 1H), 2.19 (s, 3H), 2.12 – 2.03 (m, 1H), 0.64 (d, J = 6.5 Hz, 3H)

^{13}C NMR (126 MHz, $CDCl_3$) δ 212.9, 142.9, 138.5, 138.4, 138.2, 136.7, 128.9, 128.7, 128.6, 128.6, 128.5, 128.5, 128.2, 128.0, 127.9, 127.9, 127.9, 127.6, 113.9, 86.5, 83.3, 83.1, 83.0, 78.2, 75.6, 75.1, 73.7, 73.0, 71.9, 38.1, 35.5, 27.9, 12.4

HRMS (ESI+) calculated for $C_{41}H_{46}O_6Na$ $[M+Na]^+$: 657.3192, found 657.3195

1H NMR (500 MHz, CDCl3)

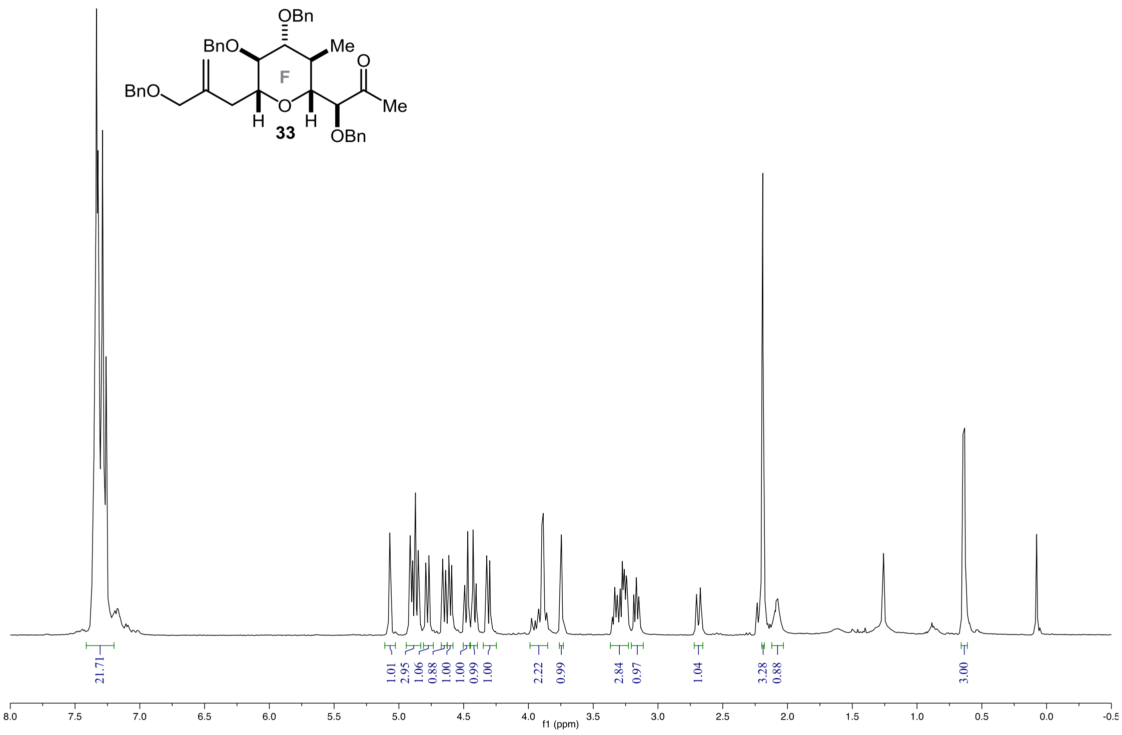

13C NMR (126 MHz, CDCl3)

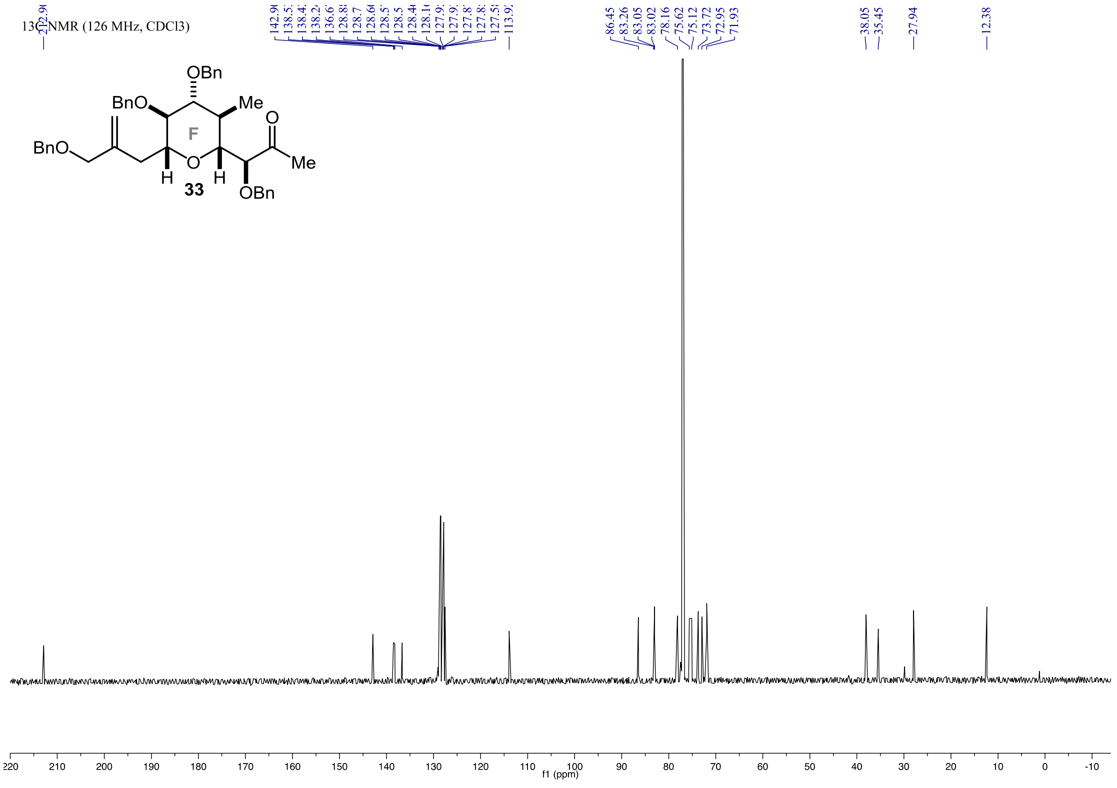

Supplementary Figure 105. ^{1}H NMR and ^{13}C NMR of **33**.

Synthesis of aldehyde **34**

A synthesis of aldehyde **34** has been reported by Crimmins.[16] We devised our own route involving crotylation[5] of aldehyde **S41**[16] as the key step:

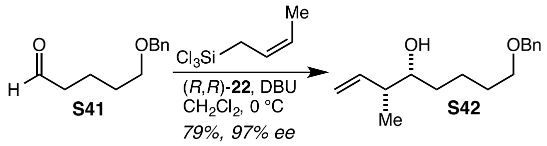

Supplementary Figure 106. Synthesis of **S42**.

To a cooled (0 °C) solution of (*R*,*R*)-**22** (25.24 g, 86.90 mmol) in CH_2Cl_2 (278 mL) was added DBU (39.00 mL, 260.7 mmol), followed by the dropwise addition of *cis*-crotyl trichlorosilane[5] (14.6 mL, 95.60 mmol). The reaction mixture was warmed to room temperature. After 1 h the reaction mixture was re-cooled to 0 °C and aldehyde **S41**[16] (15.87 g, 82.60 mmol) was added dropwise. After 3 h the reaction mixture was concentrated, the residue was resuspended in Et_2O (40 mL), and the resulting mixture was stirred for 1 h to precipitate the DBU•HCl salts. The reaction mixture was filtered, and the filtrate was treated with *n*-Bu_4NF (95 mL, 1M in THF). After 2 h 1 M HCl (400 mL) was added and the mixture was extracted with Et_2O (3 x 400 mL). The combined organic layers were washed with H_2O (2 x 100 mL) and saturated aqueous $NaHCO_3$ (100 mL). The organic layer was dried over Na_2SO_4, filtered, and concentrated. The residue was purified by flash column chromatography (0% → 30% EtOAc/Hexanes) on silica gel to give alcohol **S42** as a clear oil (16.2 g, 65.3 mmol, 79% yield, 97% ee).

The aqueous layers were combined and treated with 1 M NaOH (400 mL) and the mixture was then extracted with CH_2Cl_2 (3 x 400 mL). The combined organic layers were washed with H_2O (100 mL) and brine (100 mL), dried over Na_2SO_4, filtered and concentrated to give recovered ligand (*R*,*R*)-**22** which could be recrystallized according to the literature procedure.[5]

OR $[\alpha]_D^{25}$ +17.7° (*c* 1.00, CH_2Cl_2)

IR (ATR) 3420, 2934, 2859, 1738, 1638, 1453, 1363, 1098 cm^{-1}

^{1}H NMR (500 MHz, $CDCl_3$) δ 7.38 – 7.26 (m, 5H), 5.92 – 5.65 (m, 1H), 5.09 (d, J = 5.1 Hz, 1H), 5.06 (s, 1H), 4.50 (s, 2H), 3.53 – 3.44 (m, 3H), 2.27 (q, J = 6.6 Hz, 1H), 1.72 – 1.35 (m, 6H), 1.02 (d, J = 6.8 Hz, 3H)

^{13}C NMR (126 MHz, $CDCl_3$) δ 141.1, 138.7, 128.5, 127.8, 127.6, 115.4, 74.7, 73.0, 70.4, 43.6, 33.8, 29.8, 22.9, 14.2

HRMS (ESI+) calculated for $C_{16}H_{24}O_2Na$ $[M+Na]^+$: 271.1674, found 271.1667

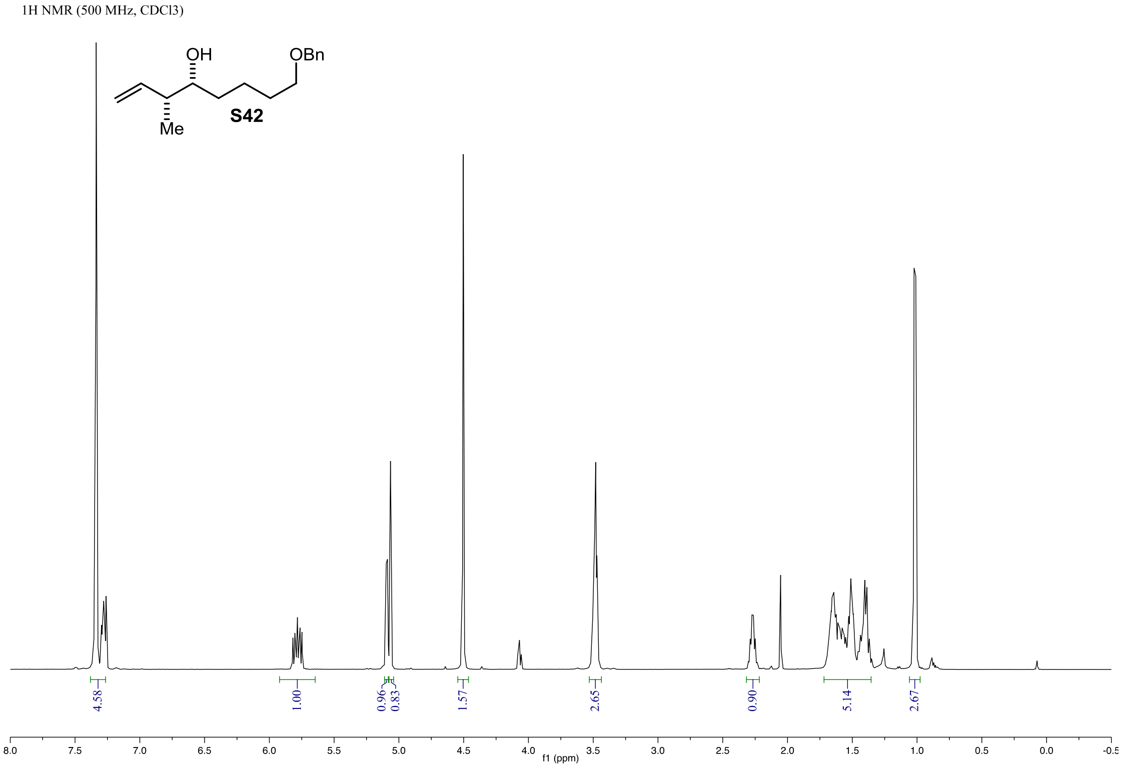

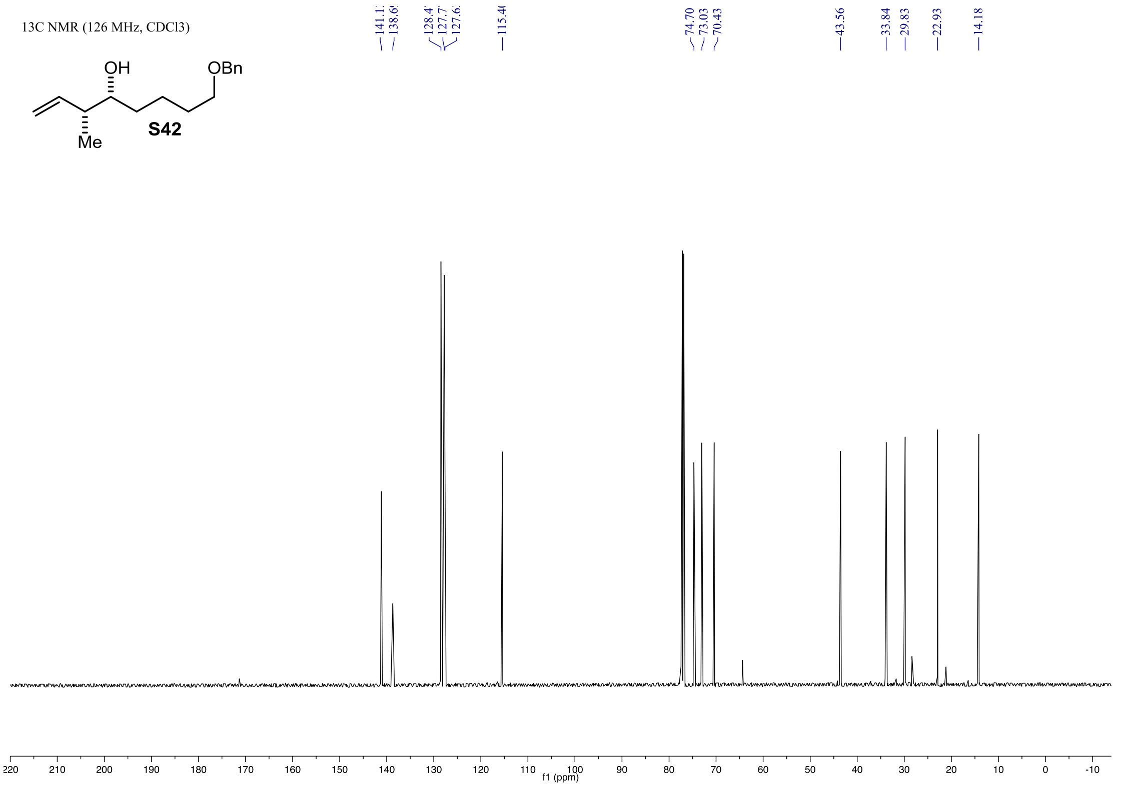

Supplementary Figure 107. ^{1}H NMR and ^{13}C NMR of **S42**.

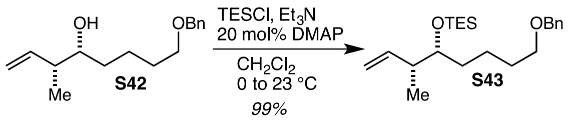

Supplementary Figure 108. Synthesis of **S43**.

To a cooled (0 °C) solution of alcohol **S42** (16.16 g, 65.06 mmol) in CH_2Cl_2 (325 mL) was added DMAP (794 mg, 6.50 mmol), Et_3N (13.6 mL, 97.5 mmol) and TES-Cl (13.1 mL, 78.1 mmol). The reaction mixture was warmed to room temperature. After 3 h, the reaction mixture was quenched by the addition of MeOH (2.62 mL, 65 mmol) and the mixture was concentrated. The residue was suspended in hexane and the mixture was stirred for 20 min. The mixture was filtered and the filtrate was concentrated. Purification of the residue by flash column chromatography (0 → 5% EtOAc/Hexanes) on silica gel afforded TES ether **S43** as a colorless oil (23.6 g, 64.35 mmol, 99%).

OR $[\alpha]_D^{23}$ +15.8° (*c* 1.00, CH_2Cl_2)

IR (ATR) 2951, 2910, 2874, 1639, 1454, 1413, 1360, 1237, 1100, 1006 cm^{-1}

^{1}H NMR (500 MHz, $CDCl_3$) δ 7.38 – 7.27 (m, 5H), 5.93 – 5.76 (m, 1H), 5.00 (d, J = 6.9 Hz, 1H), 4.98 (s, 1H), 4.50 (s, 2H), 3.55 (q, J = 7.5 Hz, 1H), 3.46 (td, J = 6.6, 1.0 Hz, 2H), 2.41 – 2.16 (m, 1H), 1.67 – 1.24 (m, 6H), 0.96 (t, J = 7.8 Hz, 9H), 0.60 (q, J = 7.9 Hz, 6H)

^{13}C NMR (126 MHz, $CDCl_3$) δ 141.6, 138.8, 128.5, 127.7, 127.6, 114.1, 76.2, 73.0, 70.6, 43.2, 33.9, 30.2, 22.2, 15.2, 7.2, 5.4

HRMS (ESI+) calculated for $C_{22}H_{39}O_2SiNa$ $[M+Na]^+$: 363.2719, found 363.2715

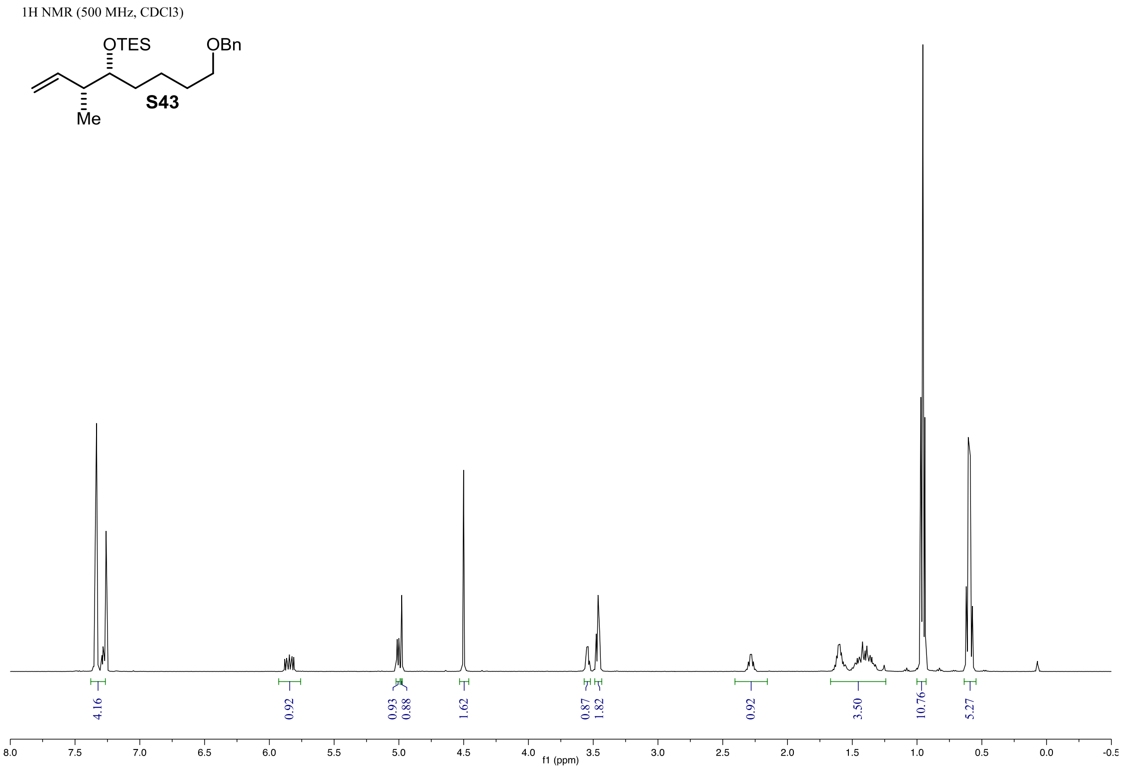

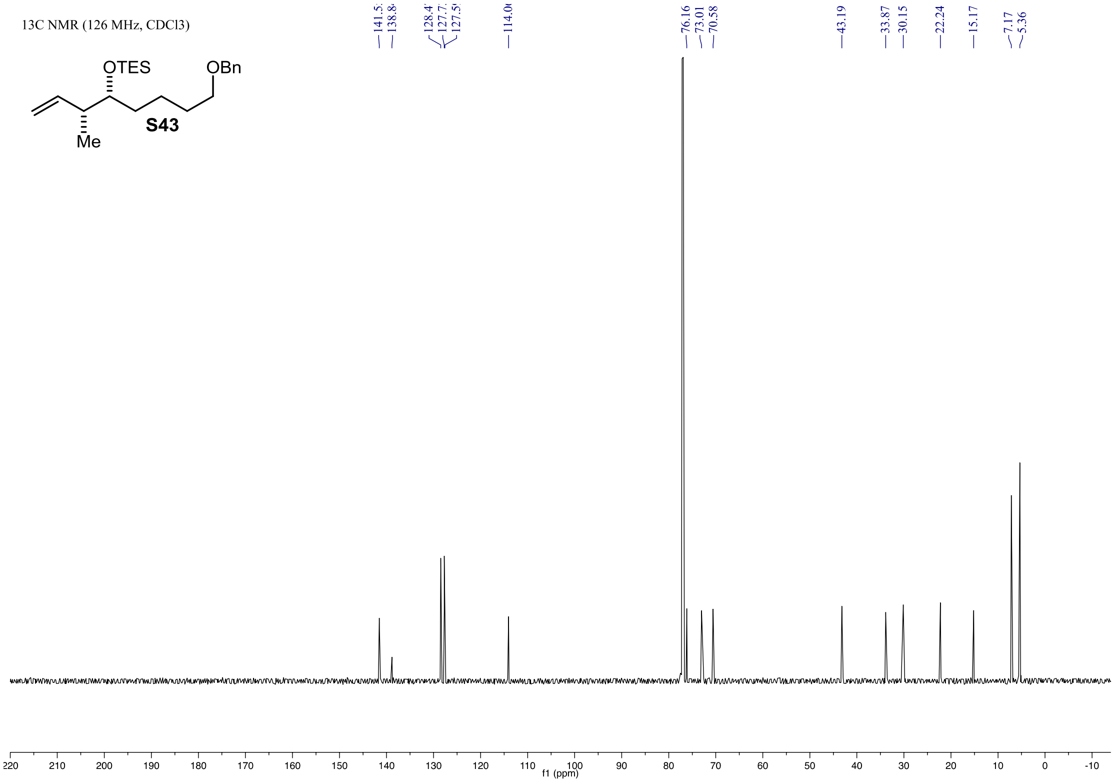

Supplementary Figure 109. ^{1}H NMR and ^{13}C NMR of **S43**.

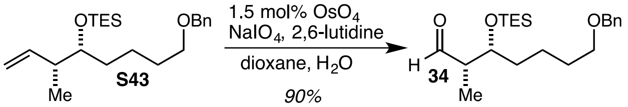

Supplementary Figure 110. Synthesis of **34**.

To a solution of alkene **S43** (500 mg, 1.38 mmol) in dioxane:H_2O (9:3 mL) were sequentially added 2,6-lutidine (207 μL, 1.79 mmol), OsO_4 (5.3 mg, 0.021 mmol), and $NaIO_4$ (678 mg, 3.17 mmol). The mixture was rapidly stirred for 2.5 h, and then cooled to 0 °C and quenched by the addition of saturated aqueous Na_2SO_3 (10 ml). The mixture was diluted with 25 mL H_2O and extracted with Et_2O (3 x 50 mL). The combined organic layers were washed with H_2O (1 x 50 mL) and brine (1 x 50 mL), dried over $MgSO_4$, filtered, and concentrated. Purification of the residue by flash column chromatography 0 → 5% EtOAc/Hexanes on pH 7 buffered silica gel afforded aldehyde **34** as a colorless oil (452 mg, 1.24 mmol, 90% yield).

OR $[\alpha]_D^{24}$ +35.93° (*c* 1.00, CH_2Cl_2)

IR (ATR) 2938, 2874, 1724, 1454, 1360, 1238, 1101, 1030 cm^{-1}

^{1}H NMR (500 MHz, C_6D_6) δ 9.62 (s, 1H), 7.32 (d, J = 7.0 Hz, 2H), 7.20 (t, J = 7.6 Hz, 2H), 7.11 (t, J = 7.3 Hz, 1H), 4.34 (s, 2H), 4.01 – 3.94 (m, 1H), 3.29 (t, J = 6.2 Hz, 2H), 2.10 – 2.01 (m, 1H), 1.55 – 1.17 (m, 6H), 1.03 – 0.89 (m, 12H), 0.55 (q, J = 8.0 Hz, 6H)

^{13}C NMR (126 MHz, C_6D_6) δ 203.3, 139.4, 128.6, 127.8, 127.7, 73.1, 72.1, 70.2, 51.4, 35.0, 30.2, 23.0, 7.6, 7.2, 5.5

HRMS (ESI+) calculated for $C_{21}H_{36}O_3SiNH_4$ $[M+NH_4]^+$: 382.2777, found 382.2781

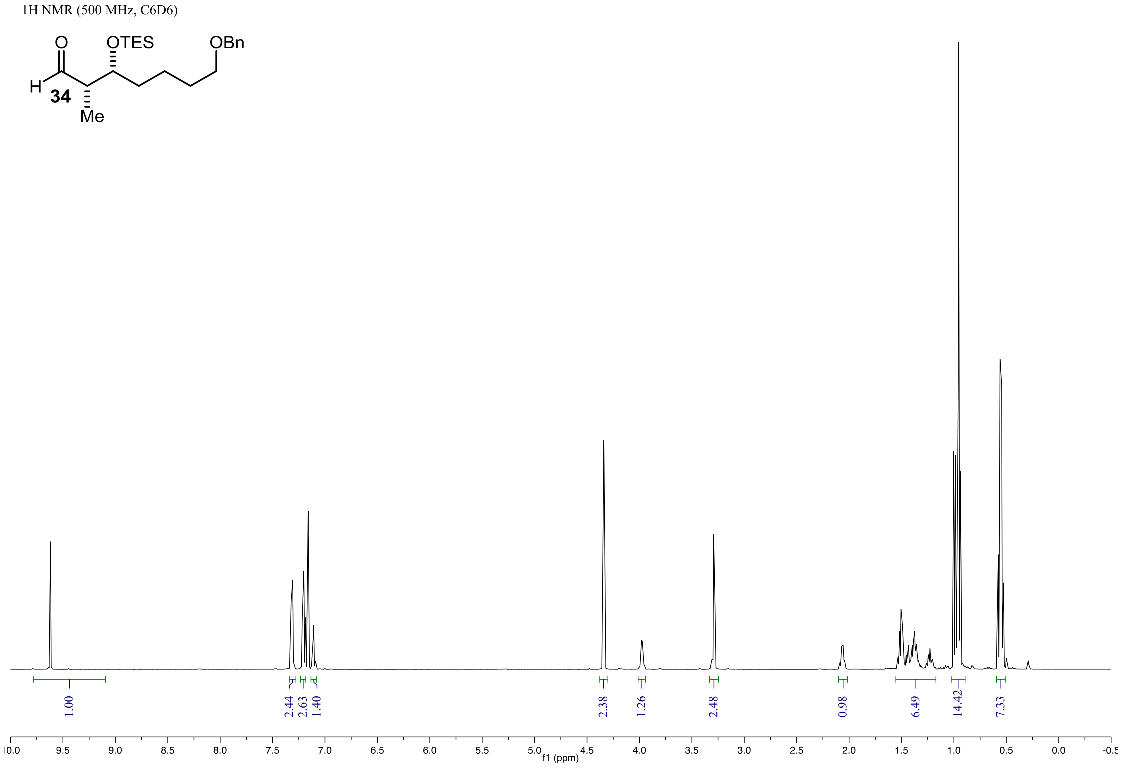

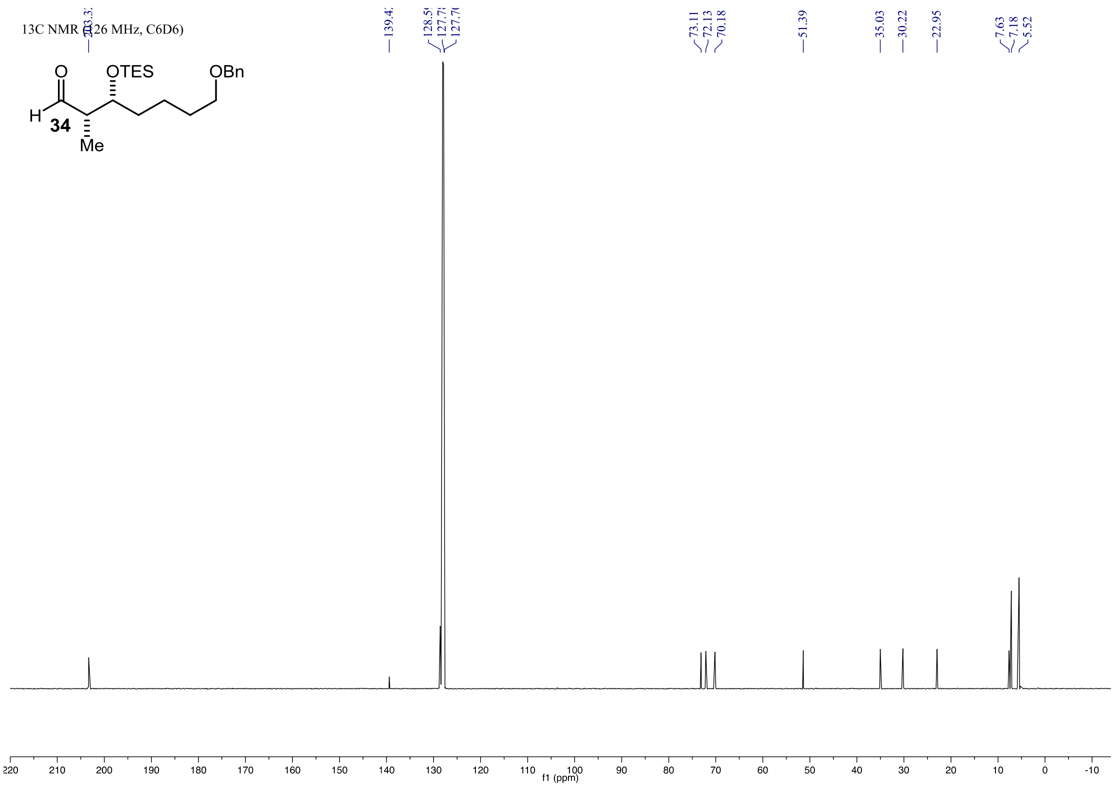

Supplementary Figure 111. ^{1}H NMR and ^{13}C NMR of **34**.

Synthesis of **13** from **33** and **34**

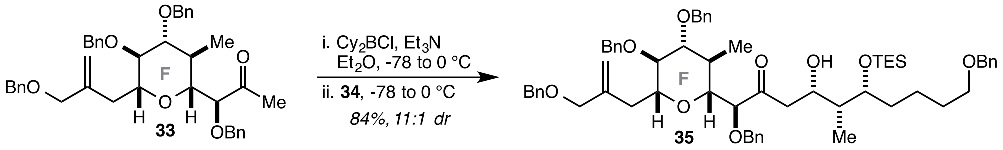

Supplementary Figure 112. Synthesis of **35**.

To a cooled (-78 °C) solution of methyl ketone **33** (5.23 g, 8.24 mmol) in Et_2O (33 mL) was added Cy_2BCl (3.6 mL, 16.48 mmol), followed by Et_3N (3.42 mL, 24.72 mmol). The reaction mixture was warmed to 0 °C and stirred for 3 h to give an off-white slurry. The mixture was re-cooled to -78 °C and a solution of aldehyde **34** (9.01 g, 24.72 mmol) in Et_2O (9 mL) was added. After 4 h the reaction mixture was warmed to -60 °C. After 14 h the reaction mixture was warmed to 0 °C. After 1 h the reaction mixture was quenched by the addition of saturated aqueous NH_4Cl (150 mL). The layers were separated and the aqueous layer was extracted with EtOAc (3 x 100 mL). The combined organic layers were dried over Na_2SO_4, filtered, and concentrated down to a volume of ~100 mL. pH 7 buffered silica gel was added and the yellow mixture was stirred at room temperature for 30 min, then filtered with EtOAc rinses (3 x 100 mL) of the filter cake. The filtrate was concentrated. Purification of the residue by flash column chromatography (5% → 20% EtOAc/Hexanes) on pH 7 buffered silica gel gave **35** as a pale yellow oil (6.89 g, 6.88 mmol, 84% yield, 11:1 *dr*).

OR $[\alpha]_D^{20}$ –28.5° (*c* 2.00, CH_2Cl_2)

IR (ATR) 3511, 3029, 2873, 1711, 1494, 1453, 1360, 1091, 1070 cm^{-1}

^{1}H NMR (500 MHz, C_6D_6) δ 7.37 (d, *J* = 7.5 Hz, 2H), 7.28 – 7.35 (m, 5H), 7.14 – 7.24 (m, 8H), 7.00 – 7.13 (m, 10H), 5.29 (s, 1H), 5.05 (s, 1H), 4.85 (d, *J* = 11.0 Hz, 1H), 4.75 – 4.79 (m, 1H), 4.76 (d, *J* = 10.6 Hz, 1H), 4.73 (d, *J* = 11.0 Hz, 1H), 4.49 (d, *J* = 11.6 Hz, 1H), 4.44 (ABq, 2H, $\Delta\nu_{AB}$ = 18.1 Hz, J_{AB} = 11.9 Hz), 4.43 (d, *J* = 11.5 Hz, 1H), 4.36 (s, 2H), 4.22 (d, *J* = 11.5 Hz, 1H), 4.01 (ABq, 2H, $\Delta\nu_{AB}$ = 37.3 Hz, J_{AB} = 13.2 Hz), 4.00 – 4.03 (m, 1H), 3.84 (d, *J* = 2.5 Hz, 1H), 3.45 (dd, *J* = 17.2, 9.8 Hz, 1H), 3.43 (td, *J* = 9.4, 2.5 Hz, 1H), 3.37 (dd, *J* = 10.2, 2.5 Hz, 1H), 3.34 (t, *J* = 6.4 Hz, 2H), 3.28 (d, *J* = 1.4 Hz, 1H), 3.21 (t, *J* = 9.3 Hz, 1H), 3.01 (dd, *J* = 10.4, 8.9 Hz, 1H), 2.75 (d, *J* = 14.5 Hz, 1H), 2.69 (dd, *J* = 17.3, 2.3 Hz, 1H), 2.35 (qq, *J* = 10.1, 6.4 Hz, 1H), 2.20 (dd, *J* = 15.0, 9.7 Hz, 1H), 1.54 – 1.75 (m, 5H), 1.42 (p, *J* = 7.7 Hz, 2H), 1.12 (d, *J* = 7.0 Hz, 3H), 1.01 (t, *J* = 7.9 Hz, 9H), 0.73 (t, *J* = 7.9 Hz, 3H), 0.63 (q, *J* = 7.9 Hz, 6H)

^{13}C NMR (126 MHz, C_6D_6) δ 213.8, 144.8, 140.0, 140.0, 139.8, 139.7, 138.5, 129.3, 129.3, 129.2, 129.2, 129.1, 129.1, 128.9, 128.8 128.7, 128.6, 128.5, 128.4, 128.3, 128.3, 128.3, 128.2, 128.2, 128.2, 114.0, 87.2, 84.7, 84.2, 83.4, 78.9, 78.0, 75.9, 75.4, 74.1, 73.9, 73.7, 72.8, 71.2, 70.9, 45.9, 42.0, 38.9, 36.8, 35.7, 31.1, 23.2, 13.1, 8.1, 7.9, 6.4

HRMS (ESI+) calculated for $C_{62}H_{82}O_9SiNa$ $[M+Na]^+$: 1021.5626, found 1021.5626

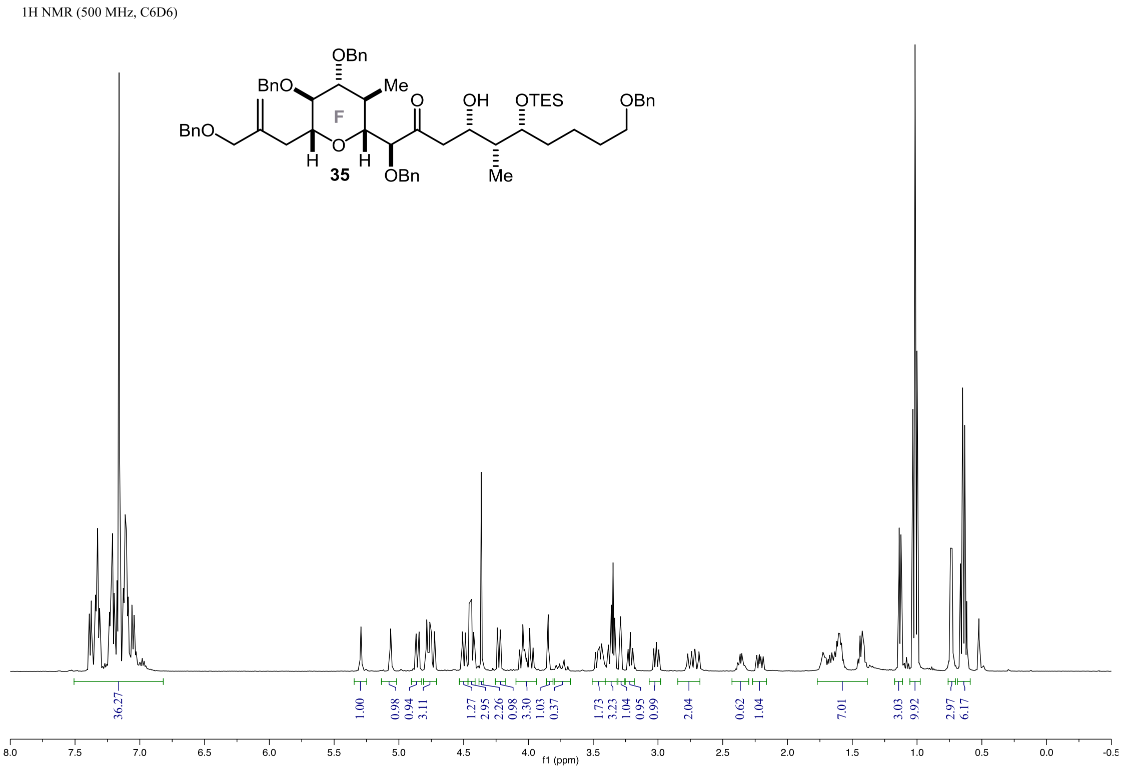

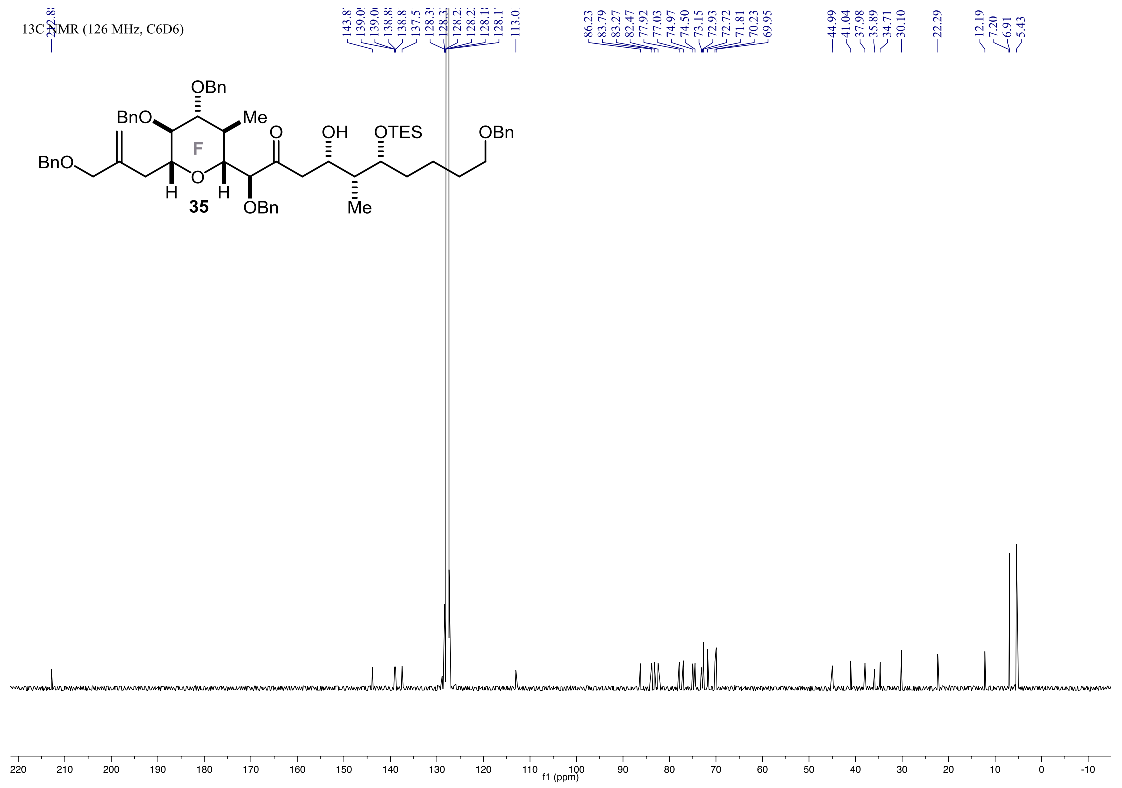

Supplementary Figure 113. ^{1}H NMR and ^{13}C NMR of **35**.

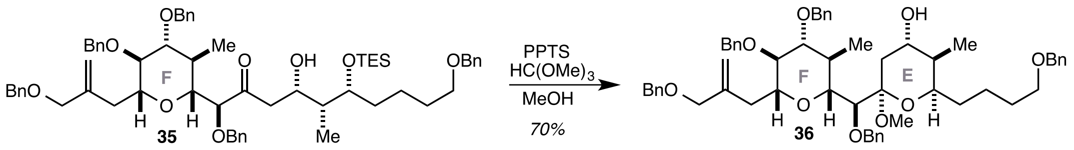

Supplementary Figure 114. Synthesis of **36**.

To a solution of alcohol **35** (6.89 g, 6.90 mmol) in dry MeOH (138 mL) was added trimethylorthoformate (13.8 mL, 124 mmol) and pyridinium *p*-toluene sulfonate (PPTS) (607 mg, 2.42 mmol). After 2 h the reaction mixture was quenched by the slow addition of saturated aqueous $NaHCO_3$ (175 mL) and the mixture was extracted with EtOAc (3 x 125 mL). The combined organic layers were washed with brine (100 mL), dried over Na_2SO_4, filtered, and concentrated. Purification of the residue by flash column chromatography (10 → 25% EtOAc/Hexanes with 1% Et_3N) on silica gel afforded hemiketal **36** as a clear oil (4.47 g, 5.00 mmol, 70% yield).

OR $[\alpha]_D^{20}$ +19.0° (*c* 2.0, CH_2Cl_2)

IR (ATR) 3527, 3029, 2858, 1495, 1454, 1356, 1213, 1090, 1062, 1025 cm^{-1}

^{1}H NMR (500 MHz, C_6D_6) δ 7.40 (t, *J* = 7.0 Hz, 4H), 7.35 (d, *J* = 7.0 Hz, 2H), 7.31 (d, *J* = 7.4 Hz, 2H), 7.24 (d, *J* = 7.0 Hz, 2H), 7.00 – 7.21 (m, 15 H), 5.39 (s, 1H), 5.17 (s, 1H), 4.99 (d, *J* = 11.2 Hz, 1H), 4.92 (d, *J* = 11.4 Hz, 1H), 4.74 (d, *J* = 11.4 Hz, 1H), 4.58 (d, *J* = 11.1 Hz, 1H), 4.55 (d, *J* = 11.6 Hz, 1H), 4.47 (d, *J* = 11.7 Hz, 2H), 4.37 (d, *J* = 12.1 Hz, 1H), 4.36 (s, 2H), 4.22 (d, *J* = 12.7 Hz, 1H), 4.12 – 4.16 (m, 1H), 4.10 (d, *J* = 12.7 Hz, 1H), 3.90 – 3.96 (m, 1H), 3.87 (d, *J* = 9.0 Hz, 1H), 3.58 (td, *J* = 9.0, 2.6 Hz, 1H), 3.56 (s, 1H), 3.38 (t, *J* = 6.1 Hz, 2H), 3.27 (t, *J* = 9.1 Hz, 1H), 3.14 (dd, *J* = 10.2, 1.3 Hz, 1H), 3.06 (dd, *J* = 10.5, 8.7 Hz, 1H), 2.95 (s, 3H), 2.83 (d, *J* = 14.1 Hz, 1H), 2.49 (ABX, 2H, $\Delta\nu_{AB}$ = 22.8 Hz, J_{AB} = 15.3 Hz, J_{BX} = 3.3 Hz, J_{AX} = 2.1 Hz), 2.32 (dd, *J* = 14.9, 9.4 Hz, 1H), 2.22 (ddt, *J* = 16.7, 10.4, 6.4 Hz, 1H), 1.76 – 1.83 (m, 1H), 1.67 – 1.75 (m, 2H), 1.65 (p, *J* = 6.2 Hz, 2H), 1.40 – 1.50 (m, 1H), 1.23 – 1.33 (m, 1H), 0.80 (d, *J* = 7.1 Hz, 3H), 0.71 (d, *J* = 6.5 Hz, 3H)

^{13}C NMR (126 MHz, C_6D_6) δ 144.6, 140.2, 140.0, 140.0, 139.9, 139.7, 129.4, 129.2, 129.2, 129.1, 129.1, 128.9, 128.8, 128.7, 128.6, 128.5, 128.5, 128.4, 128.3, 128.3, 128.3, 128.2, 128.2, 128.1, 115.1, 105.1, 87.7, 84.1, 80.9, 78.8, 77.6, 75.8, 75.5, 75.3, 74.1, 73.7, 72.6, 71.3, 70.9, 68.6, 48.0, 39.4, 38.5, 37.0, 33.8, 31.1, 30.8, 24.0, 13.8, 11.5

HRMS (ESI+) calculated for $C_{57}H_{70}O_9Na$ $[M+Na]^+$: 921.4918, found 921.4922

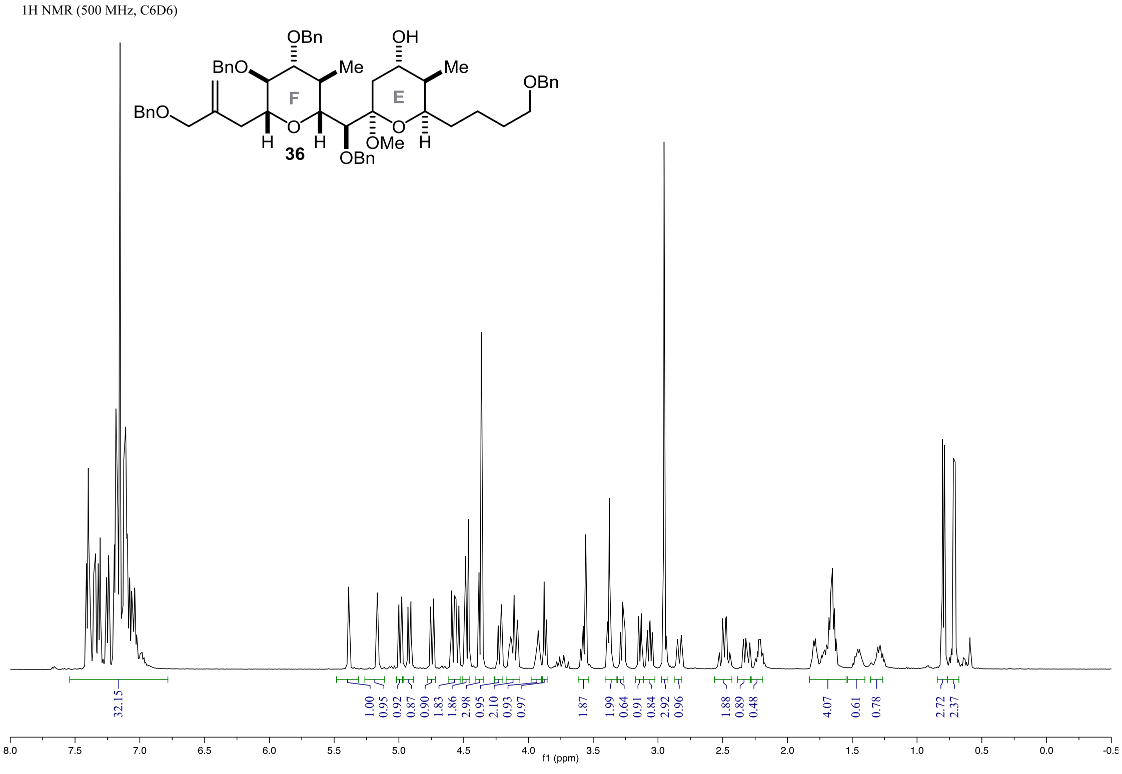

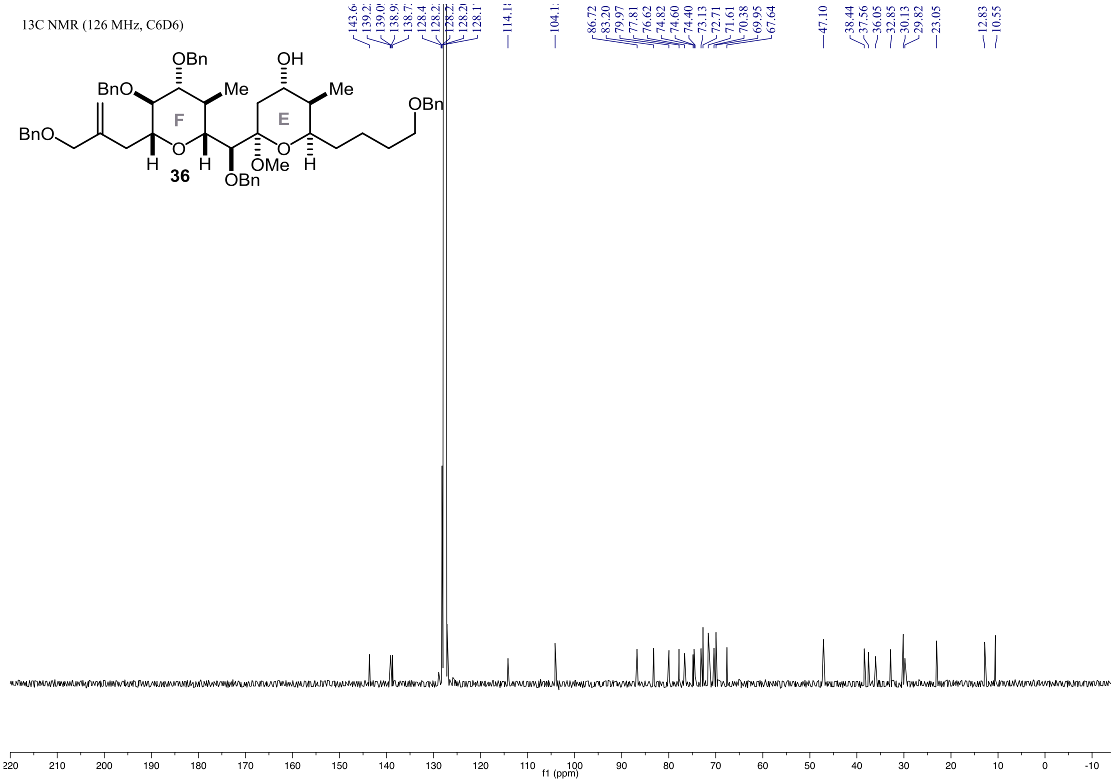

Supplementary Figure 115. ^{1}H NMR and ^{13}C NMR of **36**.

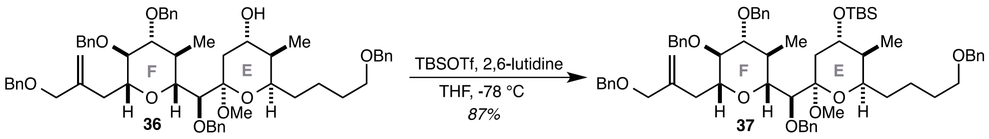

Supplementary Figure 116. Synthesis of **37**.

To a solution of 2,6-lutidine (4.23 mL, 36.48 mmol) in THF (24 mL) was added TBSOTf (2.51 mL, 10.94 mmol). The mixture was cooled to -78 °C. After 5 min the mixture was added dropwise to a cooled (-78 °C) solution of hemiketal **36** (3.28 g, 3.65 mmol) in THF (180 mL). After 1 h the reaction mixture was quenched by the addition of saturated aqueous $NaHCO_3$ (150 mL) and the mixture was diluted with EtOAc (150 mL). The layers were mixed well and then separated and the aqueous layer was extracted with EtOAc (3 x 150 mL). The combined organic layers were washed with brine (100 mL), dried over Na_2SO_4, filtered, and concentrated. Purification of the residue by flash column chromatography (10% EtOAc/Hexanes) on pH 7 buffered silica gel afforded TBS ether **37** as a pale yellow oil (3.15 g, 3.19 mmol, 87% yield).

OR $[\alpha]_D^{21}$ +18.0° (*c* 2.00, CH_2Cl_2)

IR (ATR) 3029, 2931, 2853, 1494, 1454, 1360, 1250, 1209, 1095, 1070, 1025 cm^{-1}

^{1}H NMR (500 MHz, C_6D_6) δ 7.47 (d, *J* = 7.1 Hz, 2H), 7.35 (t, *J* = 7.0 Hz, 4H), 7.31 (d, *J* = 7.0 Hz, 2H), 7.27 (d, *J* = 7.5 Hz, 2H), 7.02 – 7.22 (m, 15 H), 5.38 (s, 1H), 5.16 (s, 1H), 5.07 (d, *J* = 11.3 Hz, 1H), 4.88 (d, *J* = 11.4 Hz, 1H), 4.80 (d, *J* = 11.5 Hz, 1H), 4.67 (d, *J* = 11.2 Hz, 1H), 4.53 (d, *J* = 11.2 Hz, 2H), 4.44 (ABq, 2H, $\Delta\nu_{AB}$ = 24.5 Hz, J_{AB} = 12.5 Hz), 4.36 (s, 2H), 4.31 – 4.36 (m, 1H), 4.19 (ABq, 2H, $\Delta\nu_{AB}$ = 50.5 Hz, J_{AB} = 13.2 Hz), 3.86 (q, *J* = 3.1 Hz, 1H), 3.65 (s, 1H), 3.55 (td, *J* = 7.7, 2.6 Hz, 1H), 3.38 (t, *J* = 5.9 Hz, 2H), 3.33 (t, *J* = 9.1 Hz, 1H), 3.28 (d, *J* = 10.4 Hz, 1H), 3.20 (s, 3H), 3.05 (dd, *J* = 10.4, 8.7 Hz, 1H), 2.83 (d, *J* = 14.4 Hz, 1H), 2.51 (dd, *J* = 15.5, 3.9 Hz, 1H), 2.42 (dd, *J* = 14.7, 7.7 Hz, 1H), 2.14 (tq, *J* = 10.2, 6.4 Hz, 1H), 1.97 (dd, *J* = 15.0, 1.6 Hz, 1H), 1.64 – 1.79 (m, 4H), 1.49 – 1.60 (m, 2H), 1.33 – 1.44 (m, 1H), 1.09 (s, 9H), 0.89 (d, *J* = 7.1 Hz, 3H), 0.76 (d, *J* = 6.5 Hz, 3H), 0.20 (s, 3H), 0.10 (s, 3H)

^{13}C NMR (126 MHz, C_6D_6) δ 144.7, 140.2, 140.2, 140.1, 140.1, 129.2, 129.1, 129.1, 129.1, 128.9, 128.8, 128.7, 128.6, 128.5, 128.4, 128.4, 128.3, 128.3, 128.2, 128.1, 128.1, 128.1, 114.7, 103.5, 87.9, 83.6, 80.4, 78.9, 77.6, 75.8, 75.7, 75.1, 74.6, 73.6, 72.8, 71.7, 71.0, 67.9, 47.9, 39.7, 39.2, 36.9, 33.8, 32.1, 31.1, 26.8, 24.0, 19.0, 13.9, 11.1, -3.6, -3.9

HRMS (ESI+) calculated for $C_{63}H_{84}O_9SiNa$ $[M+Na]^+$: 1035.5782, found 1035.5789

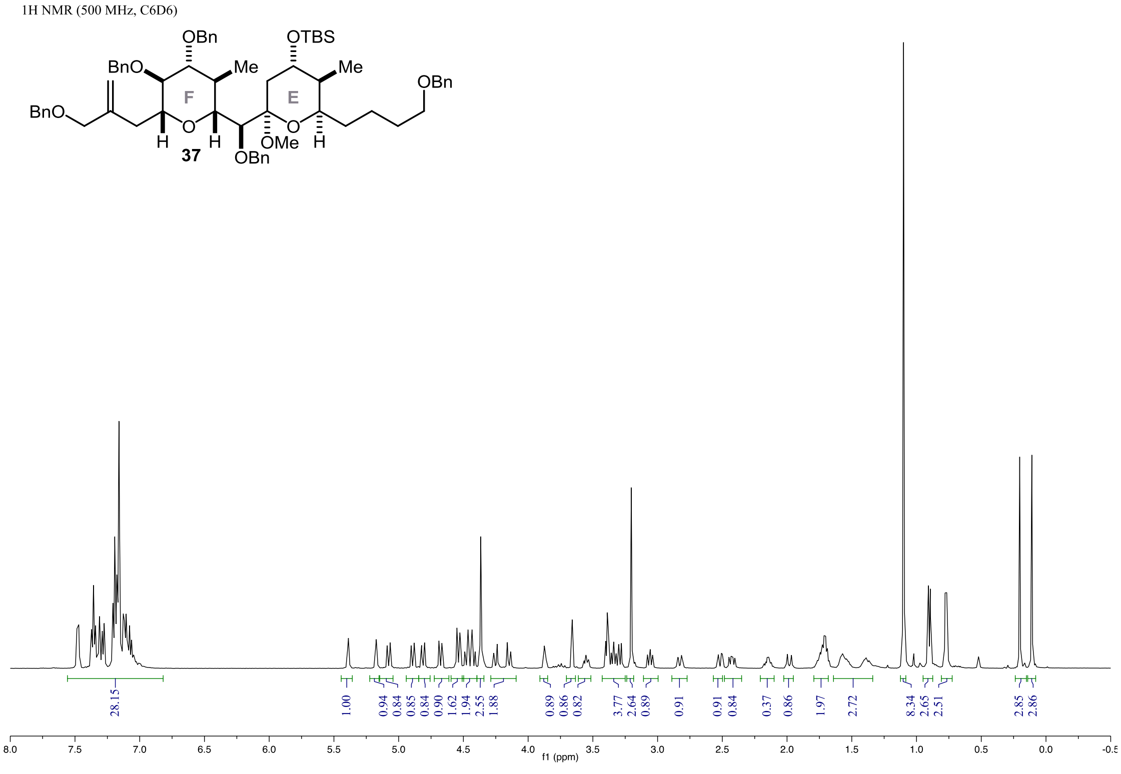

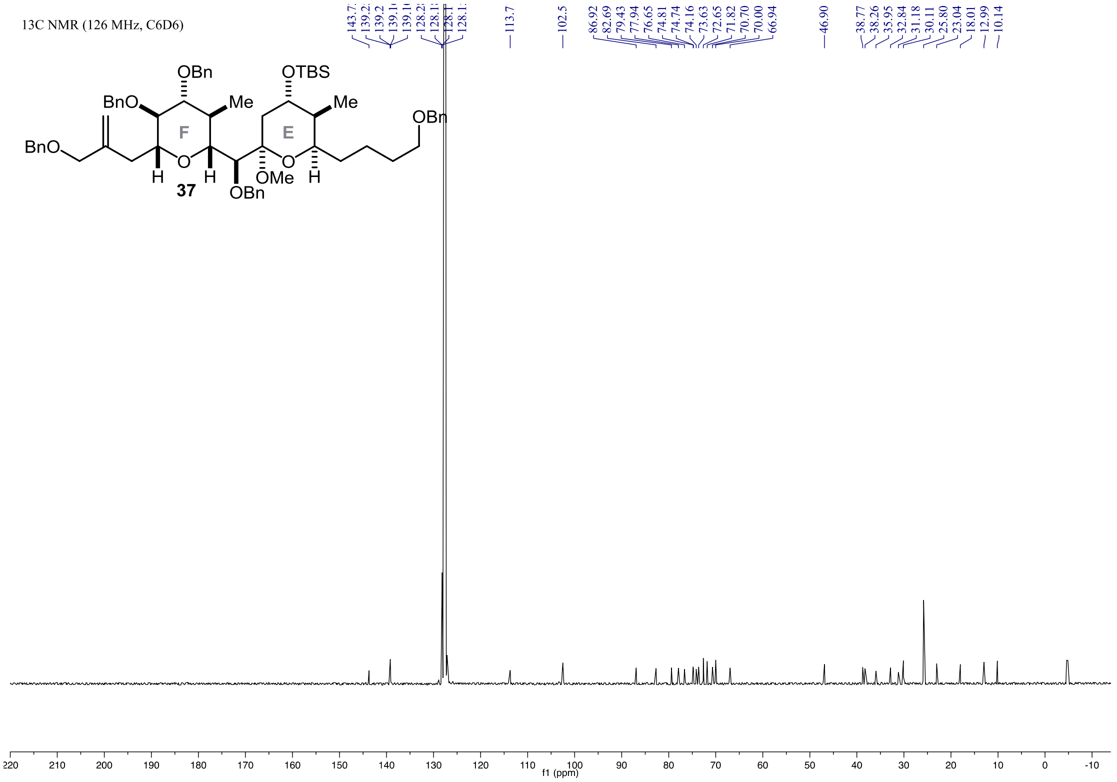

Supplementary Figure 117. ^{1}H NMR and ^{13}C NMR of **37**.

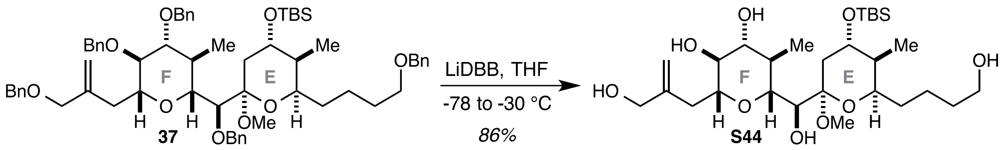

Supplementary Figure 118. Synthesis of **S44**.

Lithium di-*tert*-butylbiphenylide (LiDBB) was prepared as follows: a cooled (0 °C) solution of Li granules (415 mg, 29.61 mmol) and 4,4-di-*tert*-butyl-biphenyl (7.28 g, 27.33 mmol) in THF (57 mL) was sonicated for 3.5 h, and then used as is.

To a cooled (-78 °C) solution of TBS ether **37** (960 mg, 0.949 mmol) in THF (57 mL) was added the LiDBB solution slowly by syringe. After 2 h the reaction mixture was warmed to -30 °C. After 16 h the reaction mixture was quenched by the addition of saturated aqueous NH_4Cl (150 mL). The mixture was diluted with ethyl acetate (150 ml) and warmed to room temperature. The layers were separated and the aqueous layer was extracted with ethyl acetate (75 ml) and methylene chloride (2 x 75 mL). The combined organic layers were dried over Na_2SO_4, filtered, and concentrated. Purification of the residue by flash column chromatography (1% → 10% $MeOH/CH_2Cl_2$) on silica gel afforded pentaol **S44** as an amorphous white solid (460 mg, 0.819 mmol, 86% yield).

OR $[\alpha]_D^{25}$ +22.2° (*c* 1.00, MeOH)

IR (ATR) 3316, 2942, 2831, 1448, 1401, 1402, 1113, 1023 cm^{-1}

^{1}H NMR (500 MHz, MeOH-d_4) δ 5.15 (d, J = 1.9 Hz, 1H), 5.02 (s, 1H), 4.25 – 4.17 (m, 1H), 4.08 (s, 2H), 3.86 (d, J = 2.8 Hz, 1H), 3.75 (s, 1H), 3.61 (t, J = 6.2 Hz, 2H), 3.41 – 3.34 (m, 4H), 3.32 (d, J = 10.3 Hz, 1H), 3.22 (s, 3H), 3.12 (d, J = 17.1 Hz, 2H), 2.70 (d, J = 14.5 Hz, 1H), 2.29 (dd, J = 14.6, 8.4 Hz, 1H), 2.10 (dd, J = 15.4, 3.9 Hz, 1H), 1.86 – 1.79 (m, 1H), 1.72 – 1.41 (m, 5H), 1.07 (d, J = 6.5 Hz, 3H), 0.96 (s, 9H), 0.93 (d, J = 7.1 Hz, 3H), 0.13 (s, 3H), 0.09 (s, 3H)

^{13}C NMR (126 MHz, MeOH-d_4) δ 147.3, 113.5, 102.3, 80.0, 79.7, 79.6, 76.0, 71.9, 71.2, 68.0, 66.7, 62.9, 48.0, 47.8, 40.1, 39.5, 36.6, 33.8, 33.6, 31.4, 26.4, 23.6, 18.9, 13.5, 10.4, 9.2, -4.2, -4.7

HRMS (ESI+) calculated for $C_{28}H_{54}O_9SiNa$ $[M+Na]^+$: 585.3435, found 585.3443

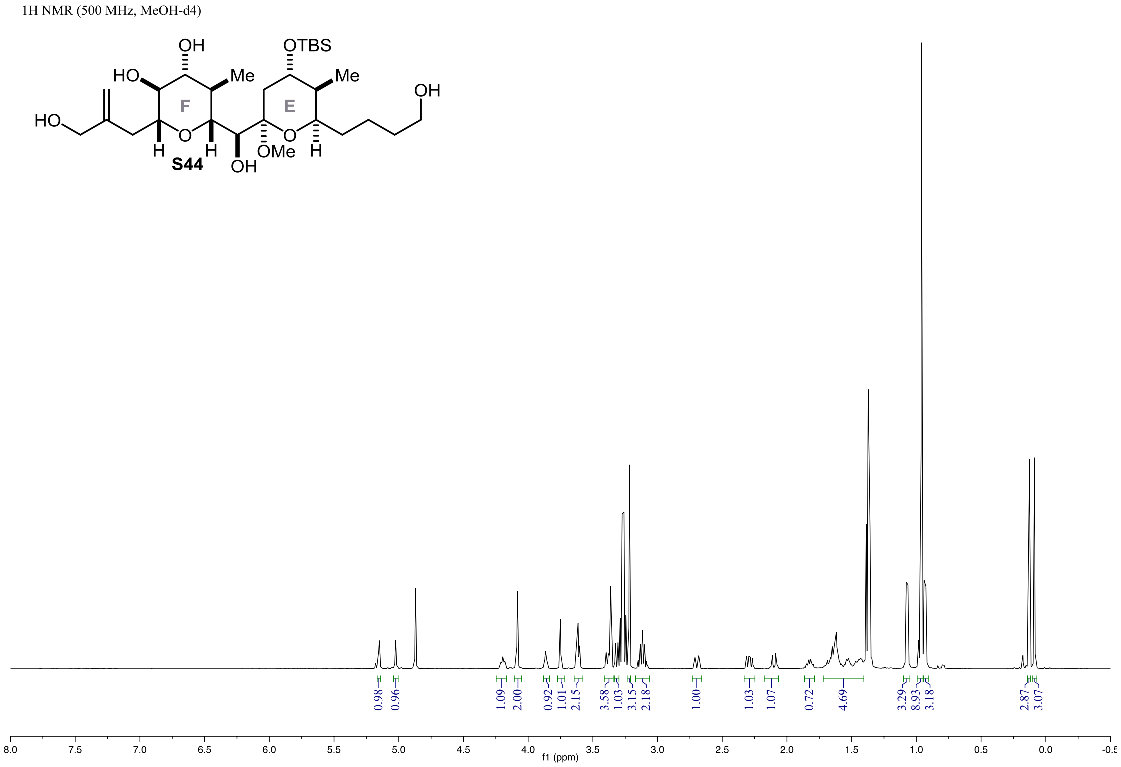

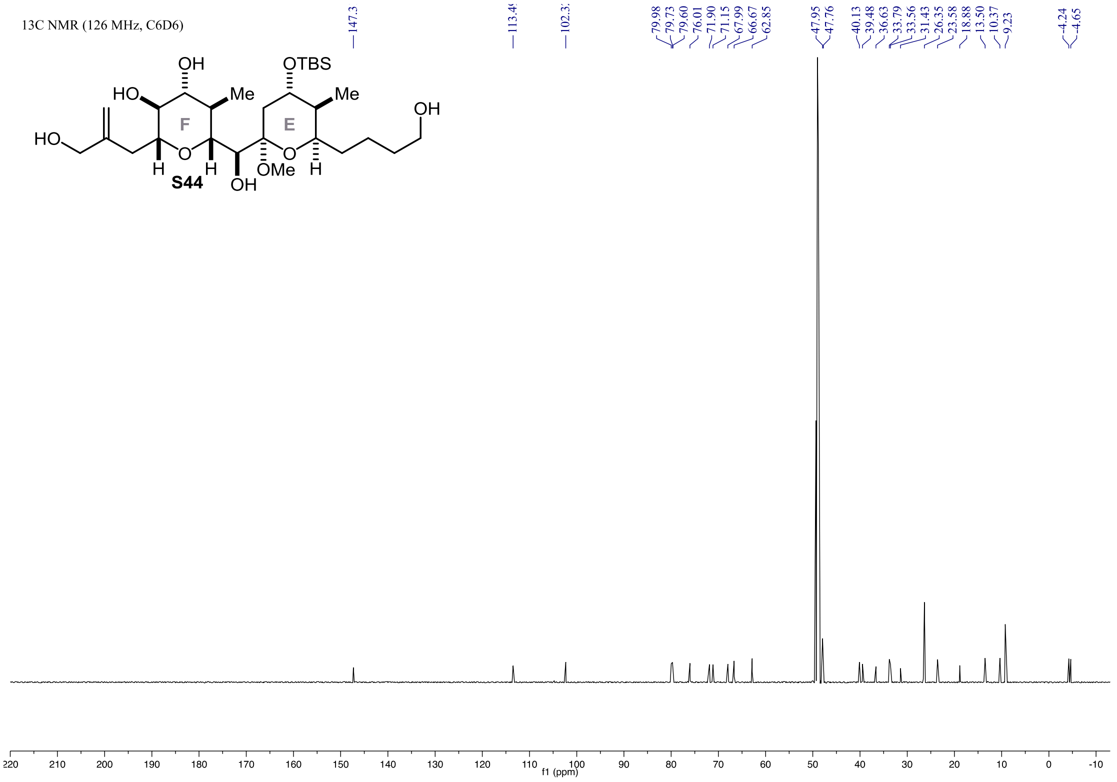

Supplementary Figure 119. ^{1}H NMR and ^{13}C NMR of **S44**.

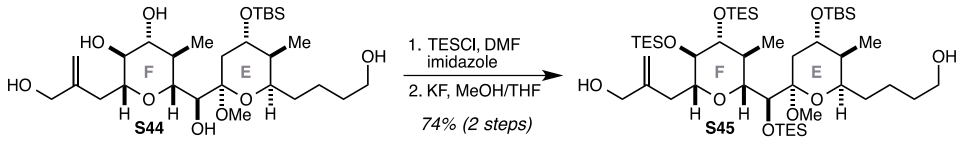

Supplementary Figure 120. Synthesis of **S45**.

To a cooled (0 °C) solution of pentaol **S44** (370 mg, 0.658 mmol) in DMF (36 mL) was added imidazole (1.57 g, 23.04 mmol) and TESCl (2.76 ml, 16.45 mmol). The reaction mixture was heated at 35 °C (oil bath, external temperature). After 14 h, the reaction mixture was diluted with Et_2O (100 mL), cooled to 0 °C and then poured into a stirred solution of saturated aqueous $NaHCO_3$ (150 mL). The layers were separated and the aqueous layer was extracted with Et_2O (3 x 50 mL). The combined organic layers were washed with water (2 x 50 mL) and brine (50 mL). The combined aqueous layers were extracted with Et_2O (75 mL). The combined organic layers were dried over Na_2SO_4, filtered, and concentrated, and the residue was left on a high vacuum line overnight. To a solution of this material in THF (27 mL) and MeOH (82 ml) was added KF (2.86 g, 49.35 mmol). After 8 h the reaction mixture was diluted with CH_2Cl_2 (200 mL) and quenched by the addition of saturated aqueous $NaHCO_3$ (300 mL). The layers were separated and the aqueous layer was extracted with CH_2Cl_2 (3 x 75 mL). The combined organic layers were dried over Na_2SO_4, filtered, and concentrated. Purification of the residue by flash column chromatography (5% EtOAc/Hexanes with 1% Et_3N) on silica gel afforded diol **S45** as a colorless oil (440 mg, 0.487 mmol, 74% yield over 2 steps from **S44**).

OR $[\alpha]_D^{22}$ +35.1° (*c* 1.00, CH_2Cl_2)

IR (ATR) 3421, 2952, 2876, 1459, 1414, 1378, 1237, 1079, 1006 cm^{-1}

^{1}H NMR (500 MHz, C_6D_6) δ 5.11 (d, J = 1.8 Hz, 1H), 5.02 (s, 1H), 4.32 – 4.21 (m, 3H), 3.97 (d, J = 2.9 Hz, 1H), 3.88 (s, 1H), 3.64 (t, J = 6.5 Hz, 1H), 3.60 – 3.49 (m, 3H), 3.44 – 3.38 (m, 3H), 3.13 (s, 3H), 2.74 – 2.65 (m, 2H), 2.34 (dd, J = 15.1, 3.7 Hz, 1H), 2.02 – 1.94 (m, 1H), 1.74 – 1.54 (m, 3H), 1.41 – 1.28 (m, 1H), 1.18 (t, J = 8.0 Hz, 9H), 1.13 – 1.03 (m, 27H), 1.00 (d, J = 7.2 Hz, 3H), 0.92 – 0.66 (m, 18H), 0.22 (s, 3H), 0.14 (s, 3H)

^{13}C NMR (126 MHz, C_6D_6) δ 148.7, 128.6, 111.9, 101.5, 82.1, 80.4, 77.7, 76.9, 71.6, 71.4, 67.7, 67.1, 62.6, 47.0, 40.3, 38.9, 37.6, 33.4, 33.1, 30.2, 26.2, 23.0, 18.4, 16.2, 10.8, 7.6, 7.5, 7.4, 6.2, 6.1, -4.1, -4.6

HRMS (ESI+) calculated for $C_{46}H_{96}O_9Si_4Na$ $[M+Na]^+$: 927.6029, found 927.6010

1H NMR (500 MHz, C6D6)

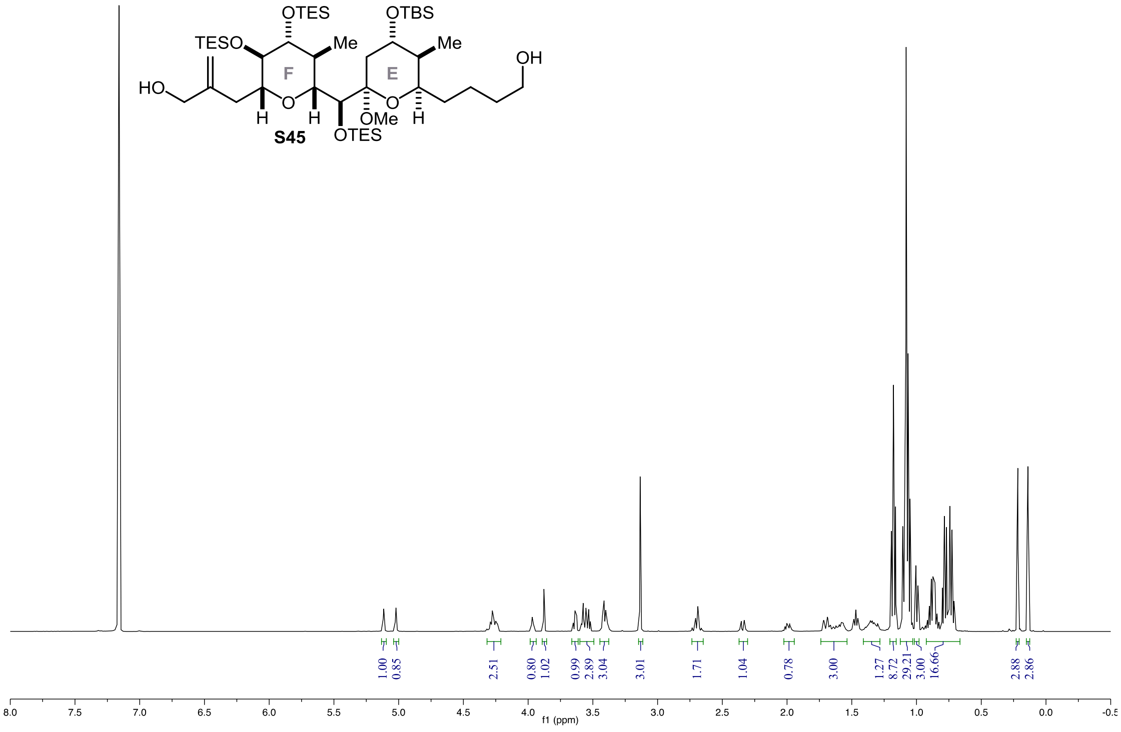

13C NMR (126 MHz, C6D6)

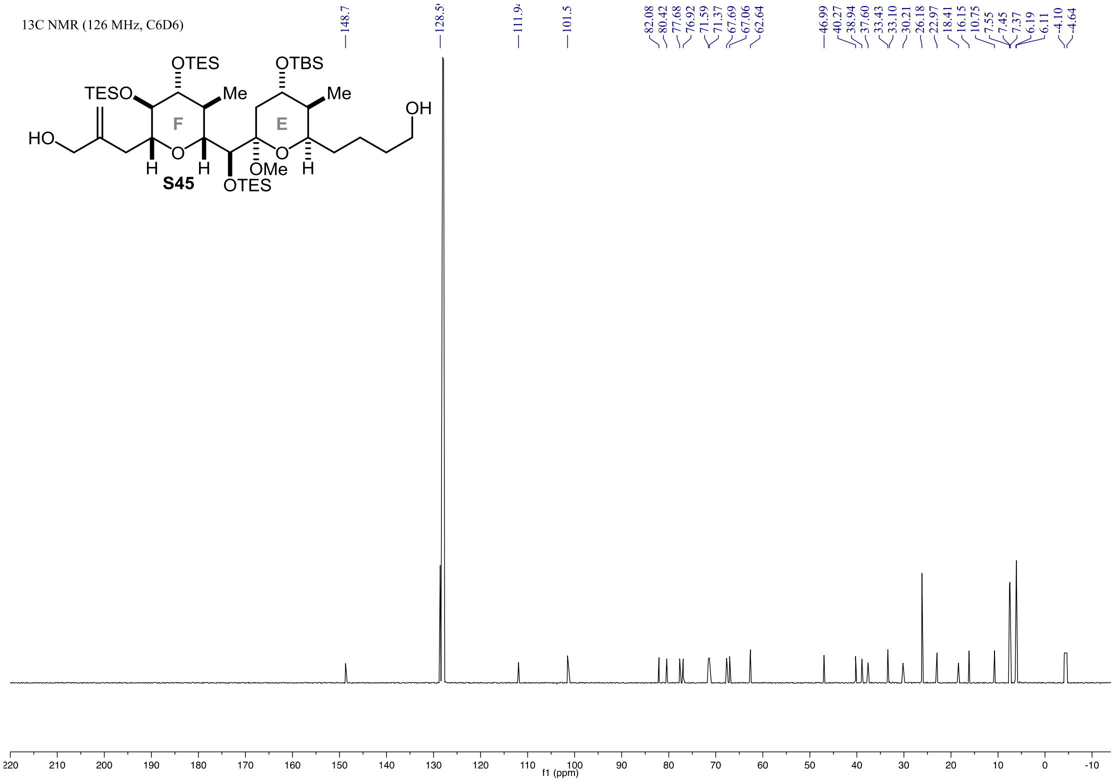

Supplementary Figure 121. ^{1}H NMR and ^{13}C NMR of **S45**.

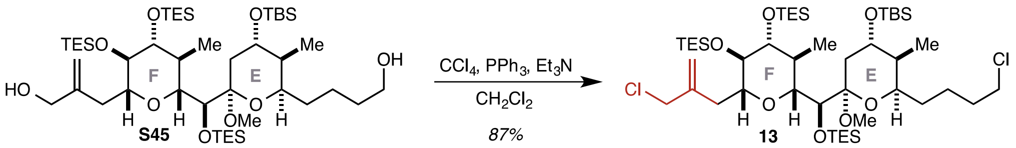

Supplementary Figure 122. Synthesis of **13**.

To a solution of diol **S45** (392 mg, 0.432 mmol) in CH_2Cl_2 (9 ml) was added Et_3N (0.60 ml, 4.32 mmol), PPh_3 (566 mg, 2.16 mmol) and CCl_4 (0.84 ml, 8.64 mmol). After 14 h the reaction mixture was quenched by the addition of saturated aqueous $NaHCO_3$ (10 mL). The layers were separated and the aqueous layer was extracted with Et_2O (2 x 7 ml). The combined organic layers were dried over Na_2SO_4, filtered, and concentrated. Purification of the residue by flash column chromatography (0 → 2% EtOAc/Hexanes with 1% Et_3N) on silica gel afforded dichloride **13** as a colorless oil (357 mg, 0.379 mmol, 87% yield).

OR $[\alpha]_D^{24}$ +31.5° (*c* 1.00, CH_2Cl_2)

IR (ATR) 2952, 2876, 1648, 1459, 1415, 1377, 1238, 1078 cm^{-1}

^{1}H NMR (500 MHz, C_6D_6) δ 5.29 (d, J = 1.5 Hz, 1H), 5.10 (s, 1H), 4.39 (d, J = 11.9 Hz, 1H), 4.23 – 4.17 (m, 2H), 3.98 – 3.94 (m, 1H), 3.86 (s, 1H), 3.57 – 3.48 (m, 3H), 3.46 (d, J = 10.6 Hz, 1H), 3.18 (td, J = 6.5, 2.1 Hz, 2H), 3.12 (s, 3H), 2.79 (dd, J = 13.8, 2.8 Hz, 1H), 2.45 (dd, J = 13.7, 8.7 Hz, 1H), 2.25 (dd, J = 15.2, 3.7 Hz, 1H), 1.96 – 1.91 (m, 1H), 1.68 – 1.50 (m, 5H), 1.14 (t, J = 8.0 Hz, 9H), 1.11 – 1.04 (m, 30H), 0.97 (d, J = 7.2 Hz, 3H), 0.84 – 0.70 (m, 18H), 0.21 (s, 3H), 0.15 (s, 3H)

^{13}C NMR (126 MHz, C_6D_6) δ 144.1, 128.6, 115.6, 101.7, 81.0, 80.6, 77.7, 76.9, 71.6, 71.3, 67.3, 49.4, 46.9, 44.9, 40.2, 39.0, 37.5, 33.1, 32.5, 30.3, 26.2, 24.0, 18.4, 16.1, 10.7, 7.5, 7.5, 7.4, 6.2, 6.2, 6.1, -4.1, -4.6

HRMS (ESI+) calculated for $C_{46}H_{94}Cl_2O_7Si_4Na$ $[M+Na]^+$: 963.5351, found 963.5353

1H NMR (500 MHz, C6D6)

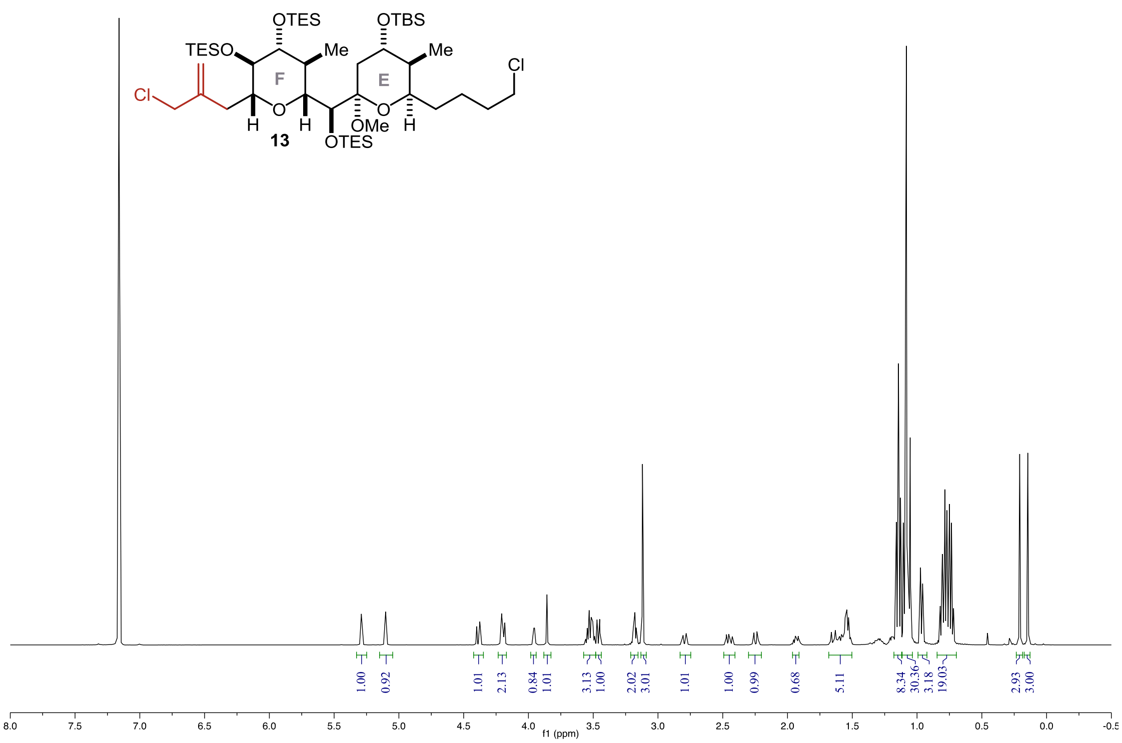

13C NMR (126 MHz, C6D6)

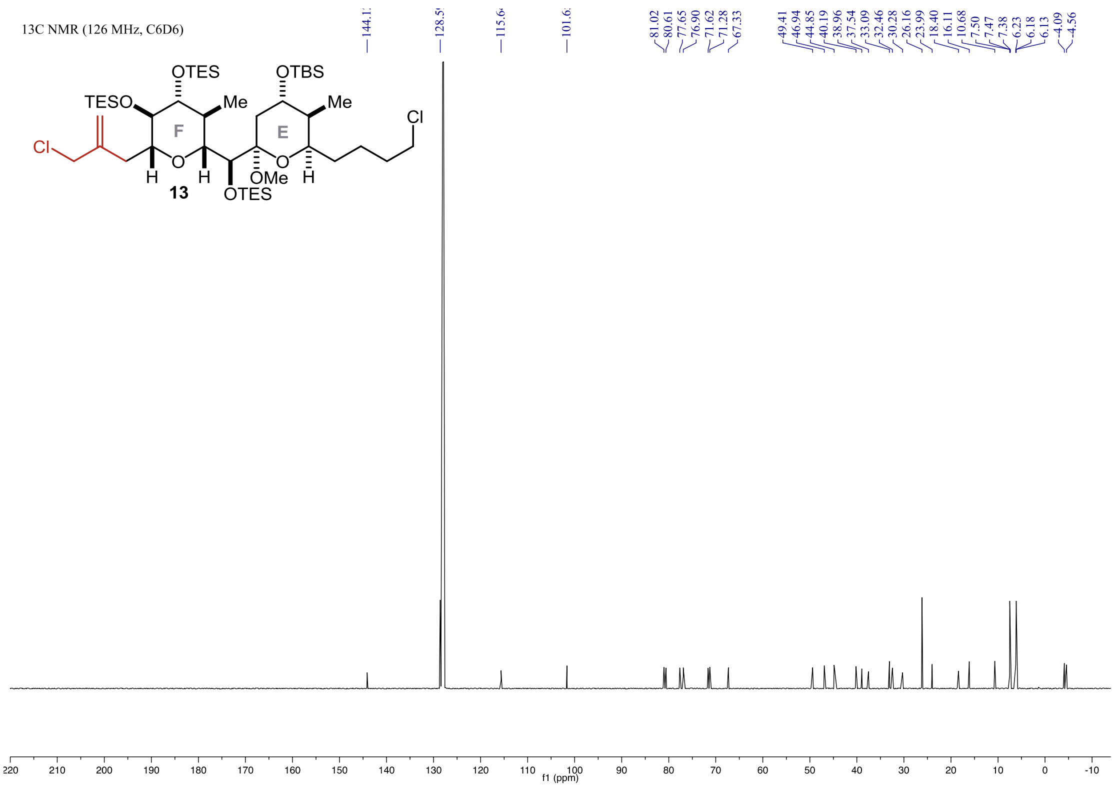

Supplementary Figure 123. ^{1}H NMR and ^{13}C NMR of **13**.

Synthesis of **39** from **13** and **11**

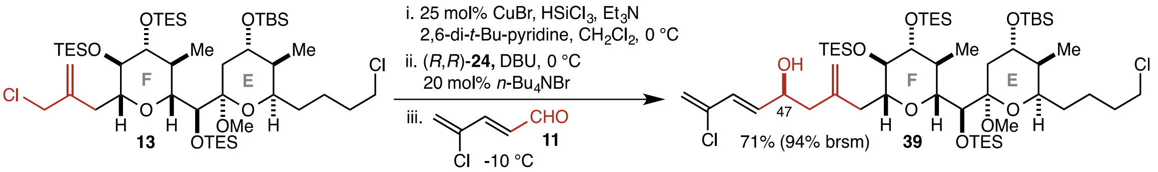

Supplementary Figure 124. Synthesis of **39**.

In an argon-atmosphere glovebox, copper (I) bromide (2.5 mg, 0.017 mmol) was placed in a vial with a stir bar. The vial was capped with a septum and transferred out of the glove box. The vial was charged with CH_2Cl_2 (0.3 mL) and Et_3N (13.5 μL, 0.097 mmol, 1.4 equiv) and then cooled to 0 °C. To a cooled (0 °C) solution of dichloride **13** (65 mg, 0.069 mmol) in CH_2Cl_2 (0.3 mL) in a separate vial under an Ar atmosphere was added 2,6-di-*t*-butyl pyridine (89.5 μL, 0.414 mmol) and Cl_3SiH (8.4 μL, 0.083 mmol). This solution was mixed thoroughly by gentle swirling of the vial, and then was added dropwise by syringe to the CuBr/Et_3N solution with a 0.1 mL CH_2Cl_2 rinse. After 5 h an additional portion of Cl_3SiH (1.4 μL, 0.014 mmol) was added dropwise to the reaction vial. After 4 h a pre-prepared solution of (*R*,*R*)-**24** (17.1 mg, 0.069 mmol), *n*-Bu_4NBr (4.5 mg, 0.014 mmol) and DBU (31 μL, 0.207 mmol) in CH_2Cl_2 (0.2 mL) was then added dropwise by syringe to the reaction mixture with a 0.1 mL CH_2Cl_2 rinse. After 2 h the reaction mixture was then cooled to -78 ºC and a solution of freshly prepared chlorodiene aldehyde **11**[17] (48.3 mg, 0.414 mmol) in CH_2Cl_2 (0.25 mL) was added dropwise by syringe with a 0.1 mL CH_2Cl_2 rinse. The reaction mixture was then warmed to -10 °C and maintained at that temperature for 12 h. The reaction mixture was warmed to 0 °C and *n*-Bu_4NF•$3H_2O$ (83 μL, 1M in THF) was added. After 2 h, the mixture was then filtered through a plug of pH 7 buffered silica gel with EtOAc washes. The filtrate was concentrated. Purification of the residue by flash column chromatography on pH 7 buffered silica (0% → 5% EtOAc/Hexanes) gave allyl chloride **13** as a clear oil (16 mg, 0.017 mmol) and alcohol **39** as a clear oil (50 mg, 0.049 mmol, 71% yield, 94% yield based on recovered **13**).

OR $[\alpha]_D^{22}$ +33.9° (*c* 1.00, CH_2Cl_2)

IR (ATR) 3469, 2953, 2876, 1643, 1590, 1459, 1414, 1378, 1238, 1093, 1006, 835, 727 cm^{-1}

^{1}H NMR (500 MHz, C_6D_6) δ 6.64 (d, J = 14.8 Hz, 1H), 6.42 (dd, J = 14.8, 4.2 Hz, 1H), 5.18 (s, 1H), 5.11 (s, 1H), 5.07 (s, 2H), 4.52 – 4.45 (m, 1H), 4.23 – 4.17 (m, 1H), 3.93 (d, J = 3.1 Hz, 1H), 3.86 (s, 1H), 3.73 – 3.68 (m, 1H), 3.62 – 3.56 (m, 3H), 3.18 (t, J = 6.5 Hz, 2H), 3.13 (s, 3H), 2.98 (d, J = 3.6 Hz, 1H), 2.62 (d, J = 6.7 Hz, 2H), 2.55 (dd, J = 13.5, 3.1 Hz, 1H), 2.33 – 2.24 (m, 2H), 2.07 – 1.98 (m, 1H), 1.70 (d, J = 14.8 Hz, 2H), 1.64 – 1.45 (m, 5H), 1.31 – 1.21 (m, 7H), 1.17 (d, J = 7.8 Hz, 9H), 1.13 – 1.04 (m, 30H), 0.95 (d, J = 7.4 Hz, 3H), 0.91 – 0.68 (m, 26H), 0.24 (s, 3H), 0.15 (s, 3H)

^{13}C NMR (126 MHz, C_6D_6) δ 145.7, 138.9, 138.1, 125.8, 114.4, 113.9, 101.2, 82.3, 79.4, 77.0, 76.2, 71.2, 70.9, 70.4, 67.0, 46.7, 46.5, 44.4, 39.7, 38.6, 34.6, 32.7, 32.0, 31.6, 30.1, 29.0, 25.6, 25.3, 23.6, 22.7, 18.0, 16.3, 13.9, 11.3, 10.3, 7.2, 7.0, 6.9, 5.8, 5.6, 5.5, -4.1, -4.6

HRMS (ESI+) calculated for $C_{51}H_{100}NCl_2O_8Si_4NH_4$ $[M+NH_4]^+$: 1040.6216, found 1040.6221

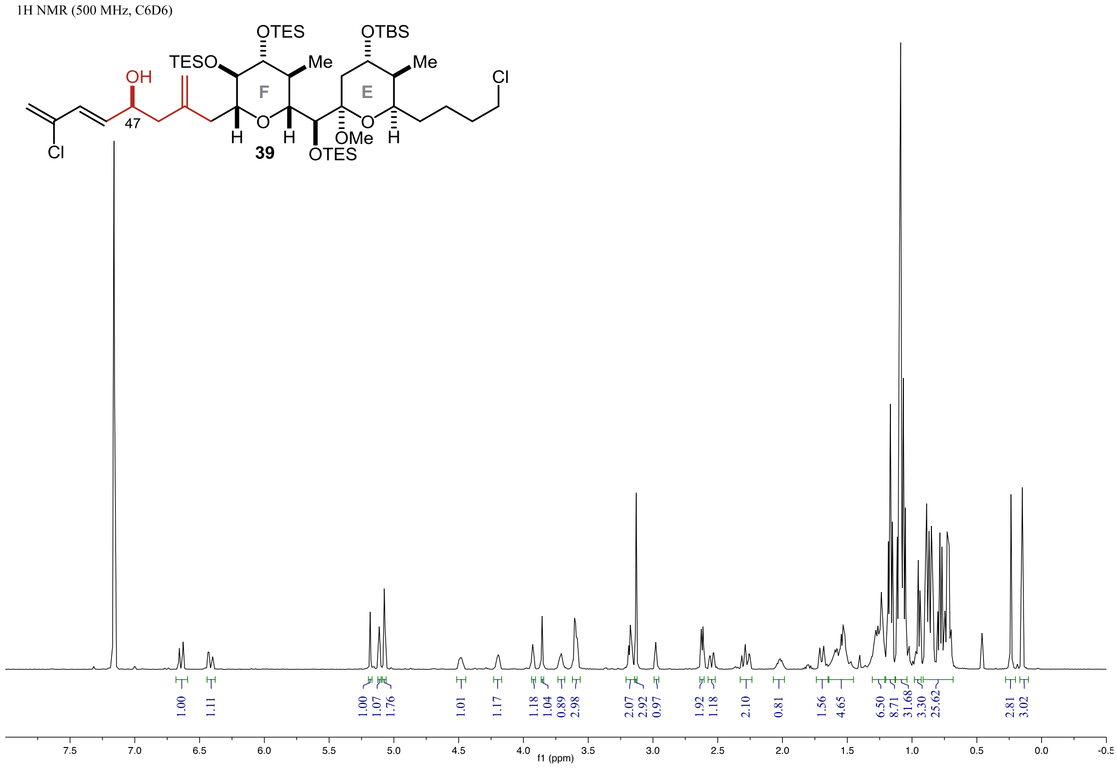

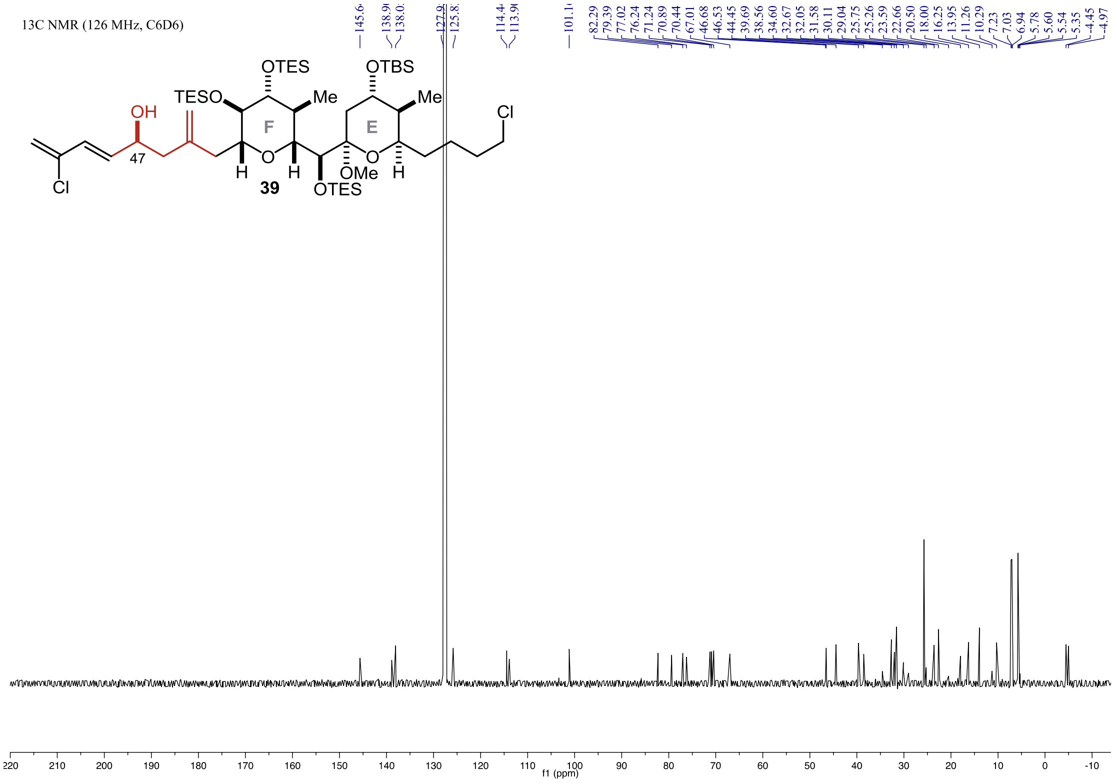

Supplementary Figure 125. ^{1}H NMR and ^{13}C NMR of **39**.

Synthesis of EF fragment **7** from **39**

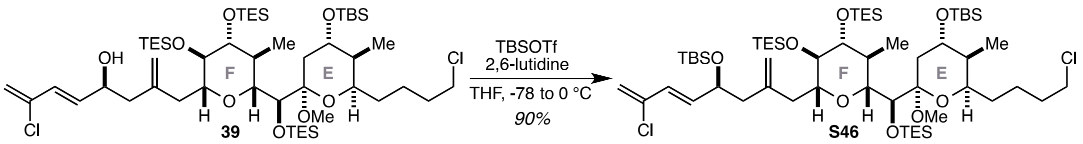

Supplementary Figure 126. Synthesis of **S46**.

To a cooled (-78 °C) solution of alcohol **39** (238 mg, 0.23 mmol) in THF (23 mL) was added a pre-prepared solution of 2,6-lutidine (268 μL, 2.30 mmol) and TBSOTf (121 μL, 0.69 mmol) in THF (4.6 mL) slowly by syringe with a 0.5 mL THF rinse. The reaction mixture was warmed to 0 °C. After 3 h the reaction mixture was quenched by the addition of saturated aqueous $NaHCO_3$ (50 mL) and extracted with Et_2O (3 x 50 mL). The combined extracts were washed with brine (50 mL), dried over Na_2SO_4, filtered, and concentrated. Purification by flash column chromatography (0% → 2% EtOAc/Hexanes) on pH 7 buffered silica gel gave TBS ether **S46** as a colorless oil (237 mg, 90% yield).

OR $[\alpha]_D^{25}$ +31.2° (*c* 1.00, CH_2Cl_2)

IR (ATR) 2953, 2876, 1646, 1590, 1461, 1415, 1378, 1250, 1112, 1079, 1005, 833, 729 cm^{-1}

^{1}H NMR (500 MHz, C_6D_6) δ 6.56 – 6.41 (m, 2H), 5.23 – 5.10 (m, 3H), 5.07 (s, 1H), 4.52 (q, J = 6.1 Hz, 1H), 4.23 (d, J = 7.9 Hz, 1H), 3.98 – 3.92 (m, 1H), 3.89 (s, 1H), 3.63 – 3.44 (m, 4H), 3.17 (d, J = 12.7 Hz, 5H), 2.79 – 2.29 (m, 5H), 2.02 – 1.92 (m, 1H), 1.74 (t, J = 14.9 Hz, 1H), 1.67 – 1.52 (m, 4H), 1.22 – 1.01 (m, 56H), 0.98 (t, J = 7.2 Hz, 3H), 0.87 – 0.74 (m, 18H), 0.31 – 0.09 (m, 12H)

^{13}C NMR (126 MHz, C_6D_6) δ 144.0, 138.5, 128.2, 126.2, 115.5, 114.7, 101.3, 81.4, 80.7, 77.6, 77.3, 71.8, 71.4, 71.2, 66.9, 46.6, 46.1, 44.5, 40.1, 38.9, 32.3, 32.1, 30.1, 26.1, 25.8, 23.6, 18.2, 18.0, 15.7, 10.3, 7.5, 7.4, 7.3, 6.3, 6.2, 6.1, 5.4, -4.1, -4.3, -4.5, -4.6

HRMS (ESI+) calculated for $C_{57}H_{114}Cl_2O_8Si_5NH_4$ $[M+NH_4]^+$: 1154.7081, found 1154.7080

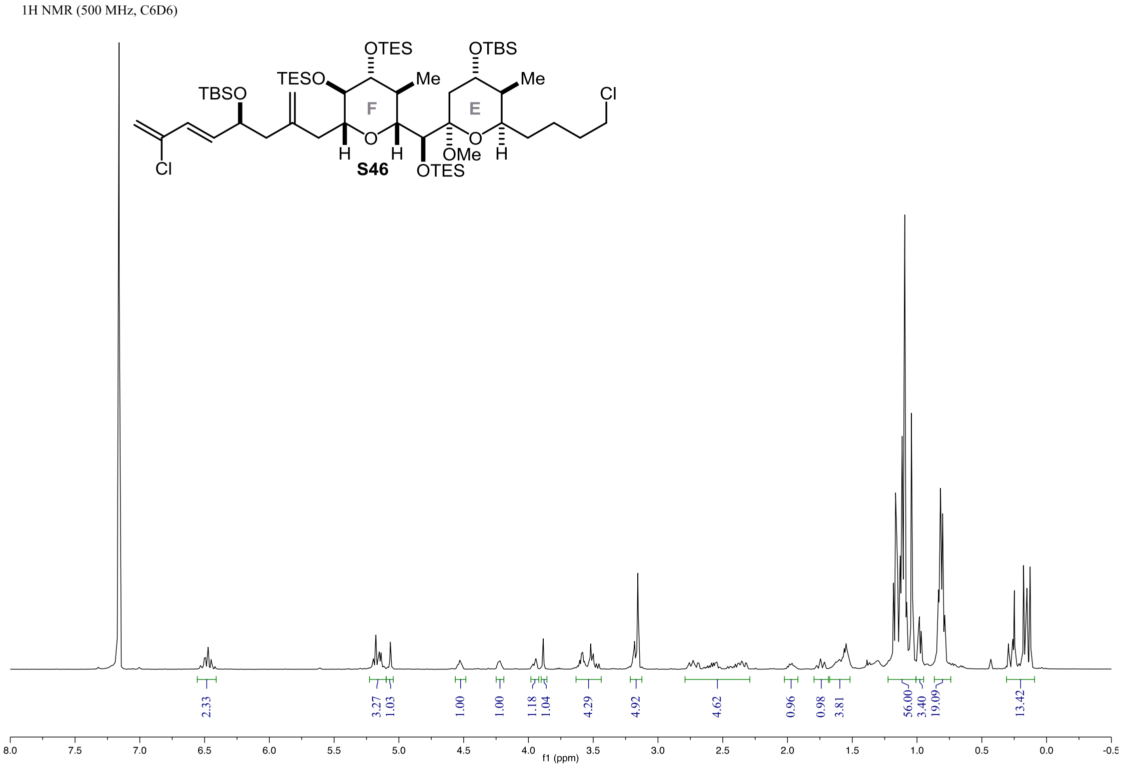

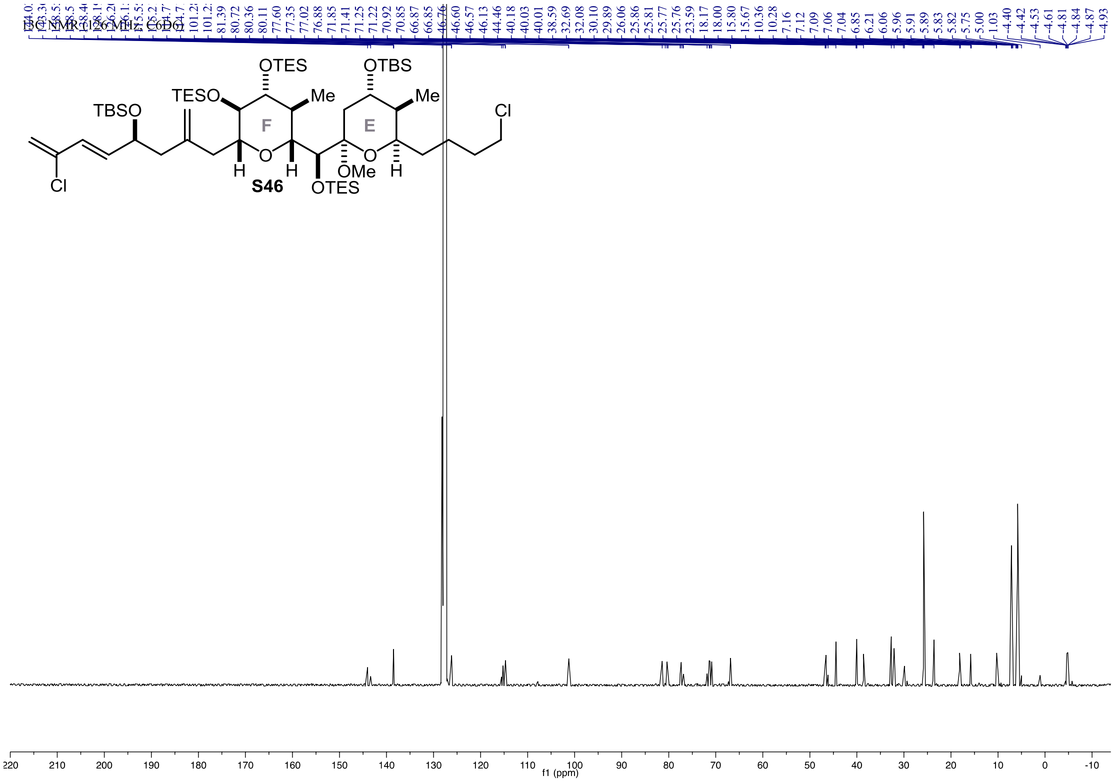

Supplementary Figure 127. ^{1}H NMR and ^{13}C NMR of **S46**.

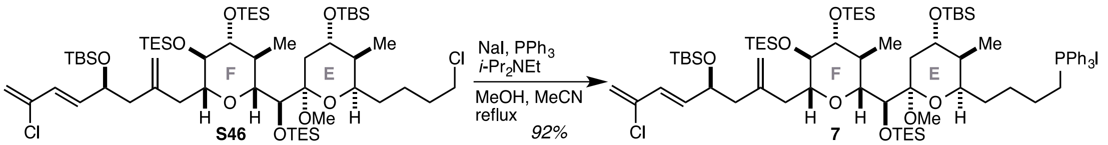

Supplementary Figure 128. Synthesis of **7**.

To a solution of chloride **S46** (235 mg, 0.206 mmol) in MeCN (11.1 mL) and MeOH (1.2 mL) was added *i*-Pr_2NEt (72 μL, 0.413 mmol), NaI (458 mg, 3.08 mmol), and PPh_3 (2.16 g, 8.23 mmol). The resulting mixture was heated at reflux for 12 h. The reaction mixture was cooled to room temperature and an additional portion of PPh_3 (1.08 g, 4.12 mmol) was added. The reaction mixture was heated at reflux for 8 h. The reaction mixture was cooled to room temperature and concentrated. The residue was suspended in CH_2Cl_2 and filtered through a cotton plug, washing with CH_2Cl_2. The filtrate was concentrated. Purification of the residue by flash column chromatography (0% → 5% MeOH/CH_2Cl_2) on pH 7 buffered silica gel afforded phosphonium salt **7** as a yellow foam (274 mg, 0.184 mmol, 92% yield). Spectroscopic data was consistent with that previously reported for this compound.[18]

^{1}H NMR (500 MHz, C_6D_6) δ 7.80 – 7.64 (m, 8H), 7.08 – 6.95 (m, 14H), 6.55 – 6.42 (m, 2H), 5.23 – 5.11 (m, 3H), 5.06 (s, 1H), 4.53 (q, *J* = 6.0 Hz, 1H), 4.45 – 4.37 (m, 1H), 4.28 (s, 2H), 4.22 (dd, *J* = 13.8, 7.2 Hz, 1H), 4.06 (s, 1H), 3.96 (d, *J* = 3.1 Hz, 1H), 3.64 – 3.48 (m, 4H), 3.35 (s, 2H), 2.79 – 2.66 (m, 2H), 2.58 (dd, *J* = 13.2, 7.6 Hz, 1H), 2.43 – 2.31 (m, 2H), 2.01 (dt, *J* = 10.7, 6.9 Hz, 1H), 1.72 (dd, *J* = 31.3, 12.4 Hz, 2H), 1.61 (d, *J* = 7.0 Hz, 1H), 1.48 (q, *J* = 8.2 Hz, 2H), 1.35 (s, 1H), 1.29 (s, 2H), 1.24 – 1.14 (m, 9H), 1.14 – 1.06 (m, 22H), 1.03 (d, *J* = 2.8 Hz, 11H), 0.98 – 0.85 (m, 7H), 0.81 (qd, *J* = 7.9, 4.2 Hz, 12H), 0.26 (s, 3H), 0.18 (d, *J* = 1.0 Hz, 5H), 0.12 (s, 3H)

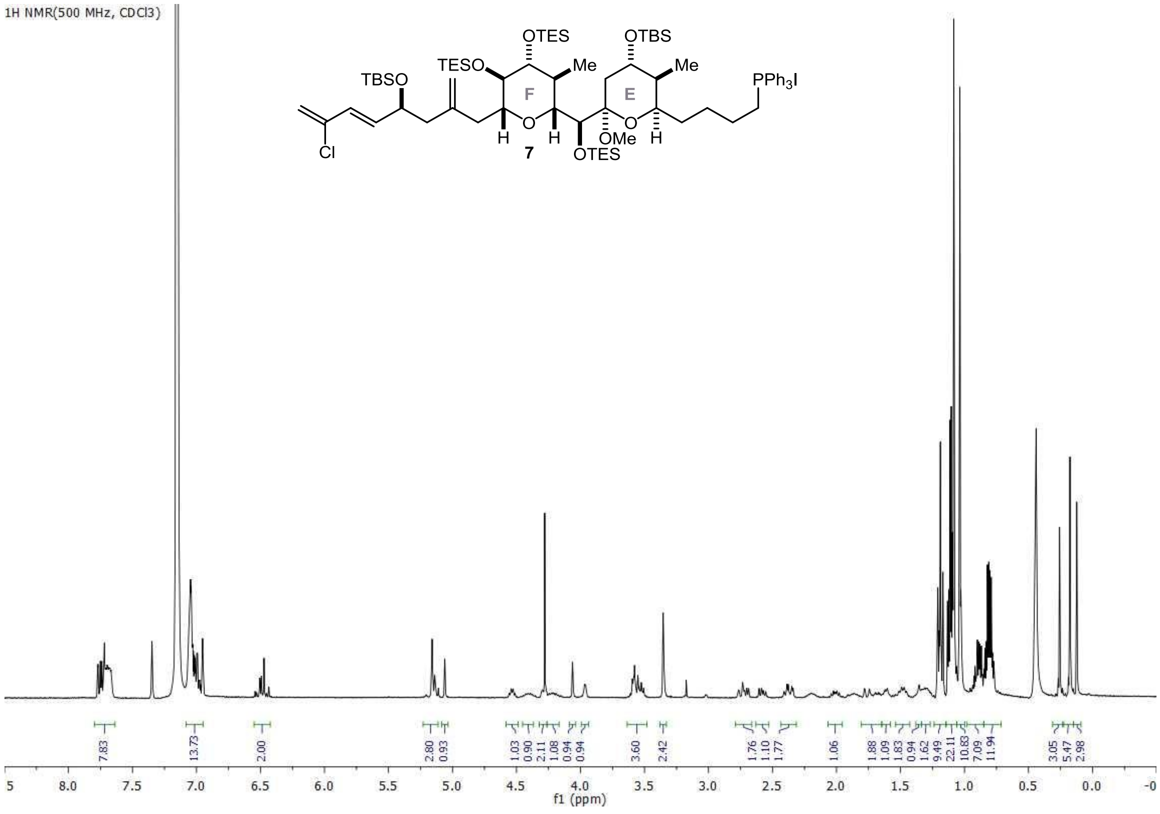

Supplementary Figure 129. ^{1}H NMR of **7**.

Synthesis of **5a** from **7** and **6a**

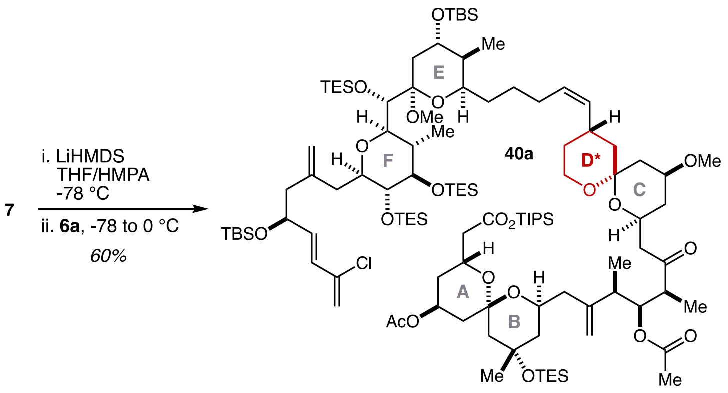

Supplementary Figure 130. Synthesis of **40a**.

Phosphonium salt **7** (90 mg, 0.060 mmol) was azeotroped with dry benzene (3x) and placed under vacuum for 24 h. Aldehyde **6a** (90 mg, 0.090 mmol) was azeotroped with dry benzene (3x) and placed under vacuum for 24 h. The dried Wittig salt was charged with dry 10% HMPA/THF solution (0.63 mL) and cooled to -78 ºC. LiHMDS (66 μL, 0.066 mmol) was added dropwise and the resultant orange solution was stirred 30 min at this temperature. A solution of the dried aldehyde in 10% HMPA/THF (0.45 mL) was added dropwise to the reaction mixture. The yellow solution was allowed to warm to 0 ºC over 1 h and stirred at 0 ºC for 1 h. The reaction was quenched by the addition of a 4:1 mixture of NH_4Cl (sat. aq.) and $Na_2S_2O_3$ (sat. aq.) (5 mL). Et_2O (7 mL) was added the layers were separated. The aqueous layer was extracted with Et_2O (3 x 4 mL) and the combined organic layers were washed with $NaHCO_3$ (sat. aq) (5 mL) and brine (5 mL), dried over $MgSO_4$, filtered and concentrated. Purification by flash column chromatography (10% → 40% EtOAc/Hexanes, then 0% → 10% MeOH/CH_2Cl_2) on pH 7 buffered silica gel afforded Wittig product **40a** as a white solid (75 mg, 0.036 mmol, 60% yield).

OR $[\alpha]_D^{27}$ +8.2° (*c* 1.00, CH_2Cl_2)

IR (ATR) 3332, 2957, 2926, 2877, 288, 1636, 1462, 1419, 1372, 1259, 1080, 1020 cm^{-1}

^{1}**H NMR** (500 MHz, C_6D_6) δ 6.55 – 6.42 (m, 2H), 5.63 (dd, J = 9.1, 3.5 Hz, 1H), 5.48 – 5.40 (m, 1H), 5.24 – 4.99 (m, 8H), 4.59 – 4.50 (m, 2H), 4.42 – 4.26 (m, 1H), 4.03 – 3.95 (m, 2H), 3.91 (s, 1H), 3.81 (td, J = 10.9, 5.3 Hz, 1H), 3.67 – 3.49 (m, 4H), 3.19 (s, 3H), 3.14 (s, 3H), 2.98 (dd, J = 9.1, 7.0 Hz, 1H), 2.93 (dd, J = 15.8, 3.9 Hz, 1H), 2.81 (dd, J = 17.1, 7.5 Hz, 1H), 2.77 – 2.65 (m, 2H), 2.61 – 2.48 (m, 3H), 2.47 – 2.32 (m, 3H), 2.29 – 2.20 (m, 2H), 2.14 – 1.90 (m, 6H), 1.83 – 1.73 (m, 6H), 1.69 – 1.59 (m, 2H), 1.51 – 1.17 (m, 29H), 1.18 – 1.00 (m, 72H), 0.97 – 0.77 (m, 16H), 0.71 – 0.60 (m, 5H), 0.29 (s, 12H), 0.26 (s, 3H), 0.18 (d, J = 3.5 Hz, 6H), 0.13 (s, 3H)

^{13}**C NMR** (126 MHz, C_6D_6) δ 208.5, 170.6, 170.2, 168.8, 148.0, 144.4, 138.9, 138.9, 135.1, 129.4, 128.6, 126.6, 115.6, 115.1, 113.8, 101.6, 97.6, 97.3, 81.8, 80.8, 77.8, 77.4, 74.7, 73.2, 71.8, 71.7, 71.3, 70.8, 67.7, 67.0, 64.9, 64.7, 61.8, 60.0, 55.3, 49.1, 48.9, 47.9, 47.1, 47.0, 45.6, 42.8, 42.7, 42.4, 42.3, 40.4, 39.4, 39.1, 38.8, 37.7, 34.6, 33.0, 32.2, 32.2, 32.0, 30.3, 30.2, 29.9, 29.4, 29.3, 28.1, 27.2, 26.2, 23.1, 23.1, 21.4, 20.6, 18.6, 18.4, 18.1, 16.2, 14.4, 13.4, 13.0, 12.3, 10.8, 7.7, 7.7, 7.5, 7.5, 7.4, 6.3, 6.2, 6.2, 1.4, -4.0, -4.2, -4.4, -4.5

HRMS (ESI+) calculated for $C_{109}H_{203}ClO_{21}Si_7NH_4$ $[M+NH_4]^+$: 2097.3234, found 2097.3267

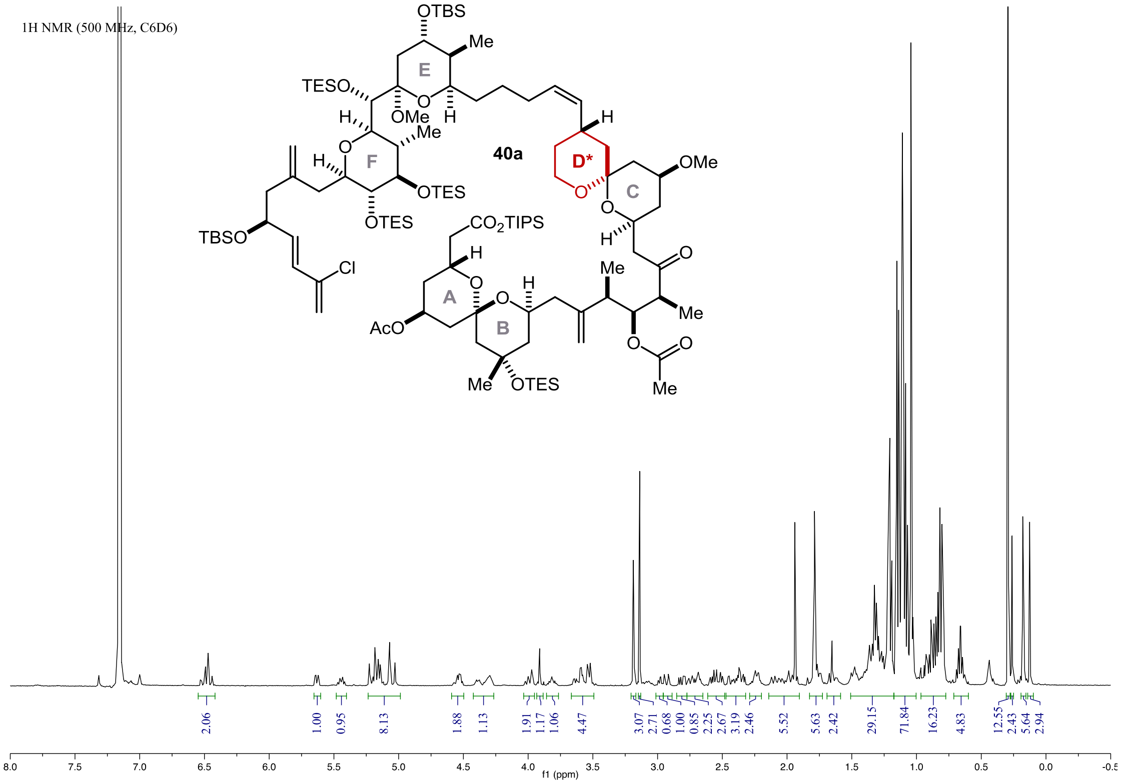

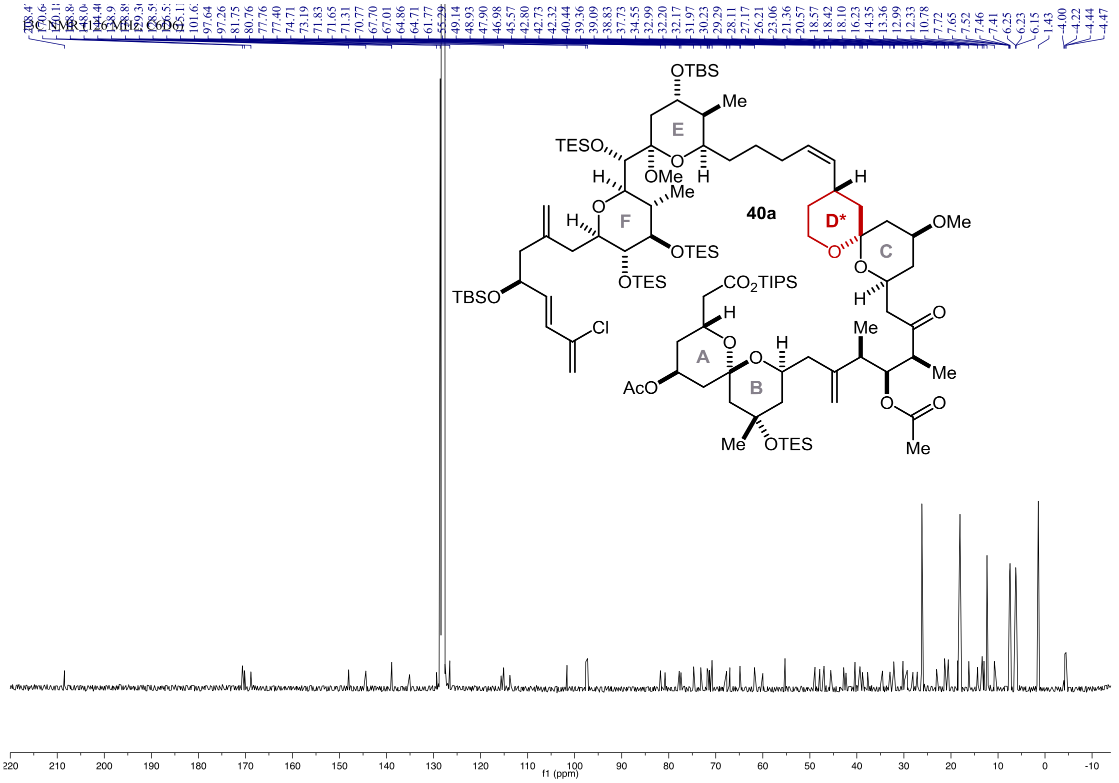

Supplementary Figure 131. ^{1}H NMR and ^{13}C NMR of **40a**.

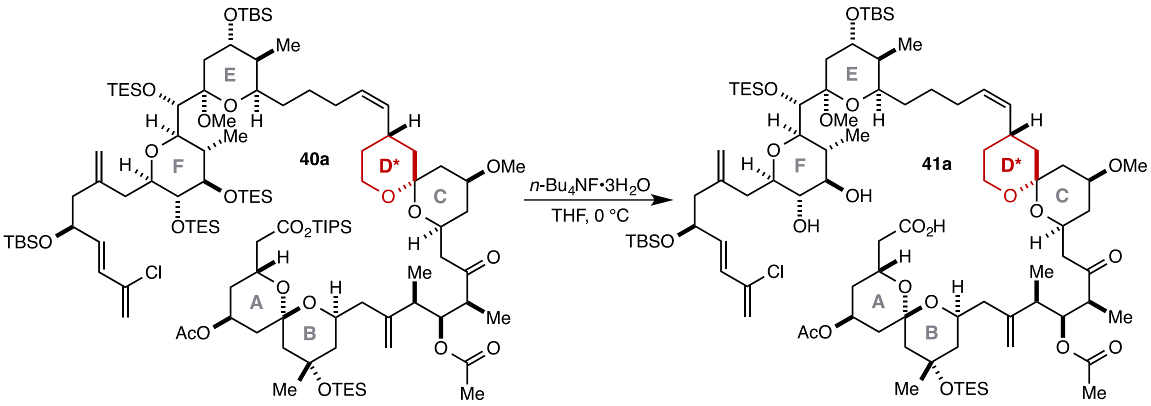

Supplementary Figure 132. Synthesis of **41a**.

Wittig product **40a** (68 mg, 0.033 mmol) was dissolved in THF (7 mL) and cooled to 0 ºC. TBAF•3H_2O (99 µL, 1M in THF) was added in 6 portions over 1 h and stirring was continued at 0 ºC for 3 h. The reaction was quenched by the addition of NH_4Cl (sat. aq.) (10 mL) and EtOAc (10 mL). The layers were separated and the aqueous layer was extracted with EtOAc (3 x 10 mL). The combined organic layers were washed with brine (15 mL). The combined aqueous layers were back-extracted with CH_2Cl_2 (2 x 10 mL). The combined organic layers were dried over Na_2SO_4, filtered and concentrated. Purification by flash column chromatography (0% → 5% MeOH/CH_2Cl_2) on silica gel afforded *seco*-acid **41a** as a white solid (46 mg, 0.0274 mmol, 83% yield).

OR $[\alpha]_D^{25}$ +25.4° (*c* 1.00, CH_2Cl_2)

IR (ATR) 3448, 2951, 2930, 2876, 2860, 1734, 1590, 1551, 1461, 1371, 1241, 1182, 1144, 1106, 1077, 1027, 1004, 959, 895, 835, 775, 739 cm^{-1}

^{1}H NMR (500 MHz, C_6D_6) δ 6.56 – 6.40 (m, 2H), 5.63 (dd, J = 9.0, 3.5 Hz, 1H), 5.53 – 5.40 (m, 2H), 5.24 – 4.93 (m, 12H), 4.58 – 4.35 (m, 2H), 4.34 – 4.24 (m, 4H), 4.02 – 3.87 (m, 3H), 3.86 – 3.78 (m, 1H), 3.66 – 3.46 (m, 4H), 3.23 – 3.17 (m, 7H), 3.14 (s, 3H), 3.03 – 2.95 (m, 1H), 2.89 – 2.66 (m, 3H), 2.62 – 1.85 (m, 12H), 1.83 – 1.68 (m, 7H), 1.63 (d, J = 13.4 Hz, 2H), 1.55 – 1.39 (m, 3H), 1.39 – 0.76 (m, 93H), 0.67 (q, J = 7.9 Hz, 6H), 0.29 – 0.23 (m, 3H), 0.20 – 0.08 (m, 10H)

^{13}C NMR (126 MHz, C_6D_6) δ 208.7, 170.1, 169.1, 167.4, 148.0, 144.4, 143.7, 138.9, 138.9, 135.1, 129.4, 126.6, 115.7, 115.1, 113.8, 101.7, 97.7, 97.6, 81.7, 81.2, 80.8, 77.8, 77.4, 76.9, 74.9, 73.3, 72.2, 71.8, 71.7, 71.3, 70.9, 67.7, 66.7, 64.9, 64.6, 61.7, 60.0, 55.8, 55.3, 53.3, 49.2, 49.0, 48.0, 47.1, 47.1, 45.2, 42.7, 42.4, 42.2, 40.6, 40.4, 39.0, 38.7, 37.7, 34.4, 32.9, 32.4, 32.2, 32.2, 30.2, 30.2, 30.1, 29.9, 29.9, 29.8, 29.7, 29.3, 28.1, 27.8, 26.2, 26.2, 25.1, 23.1, 21.3, 18.6, 18.4, 16.2, 16.1, 14.4, 13.4, 13.1, 10.8, 7.7, 7.5, 7.5, 7.3, 6.4, 6.3, 6.2, 6.2, 6.0, -4.0, -4.2, -4.4, -4.4, -4.5

HRMS (ESI+) calculated for $C_{88}H_{155}ClO_{21}Si_4NH_4$ $[M+NH_4]^+$: 1713.0170, found 1713.0188

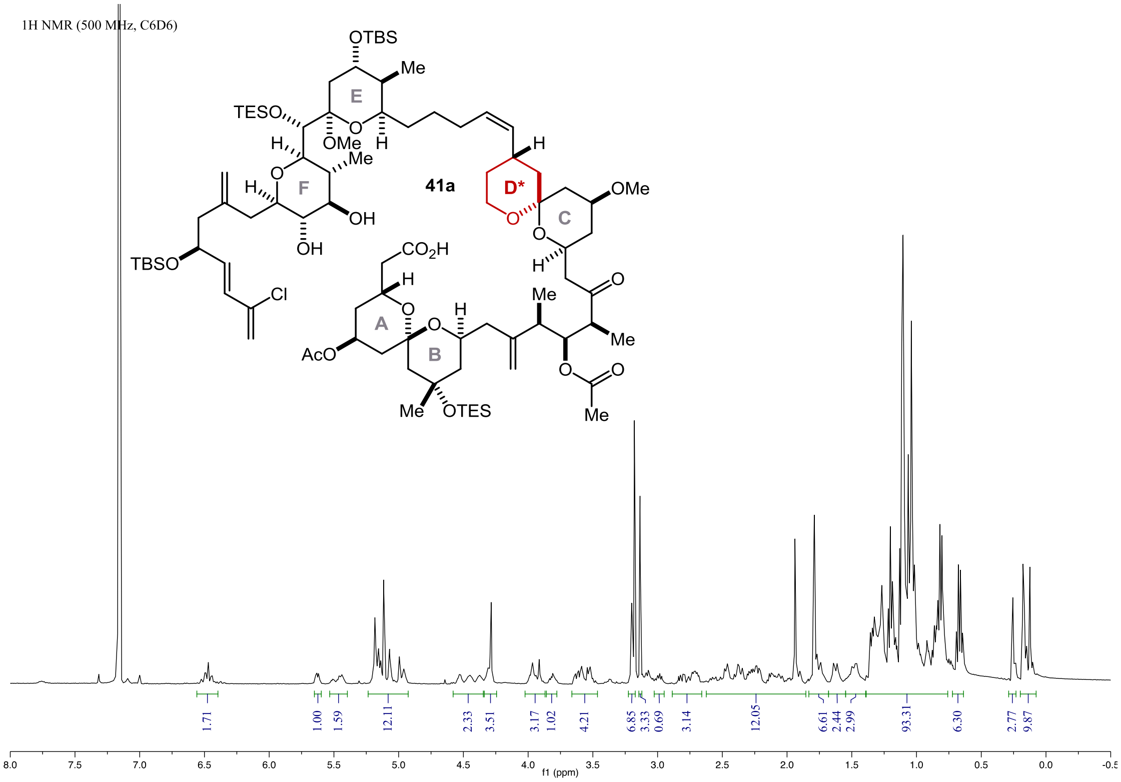

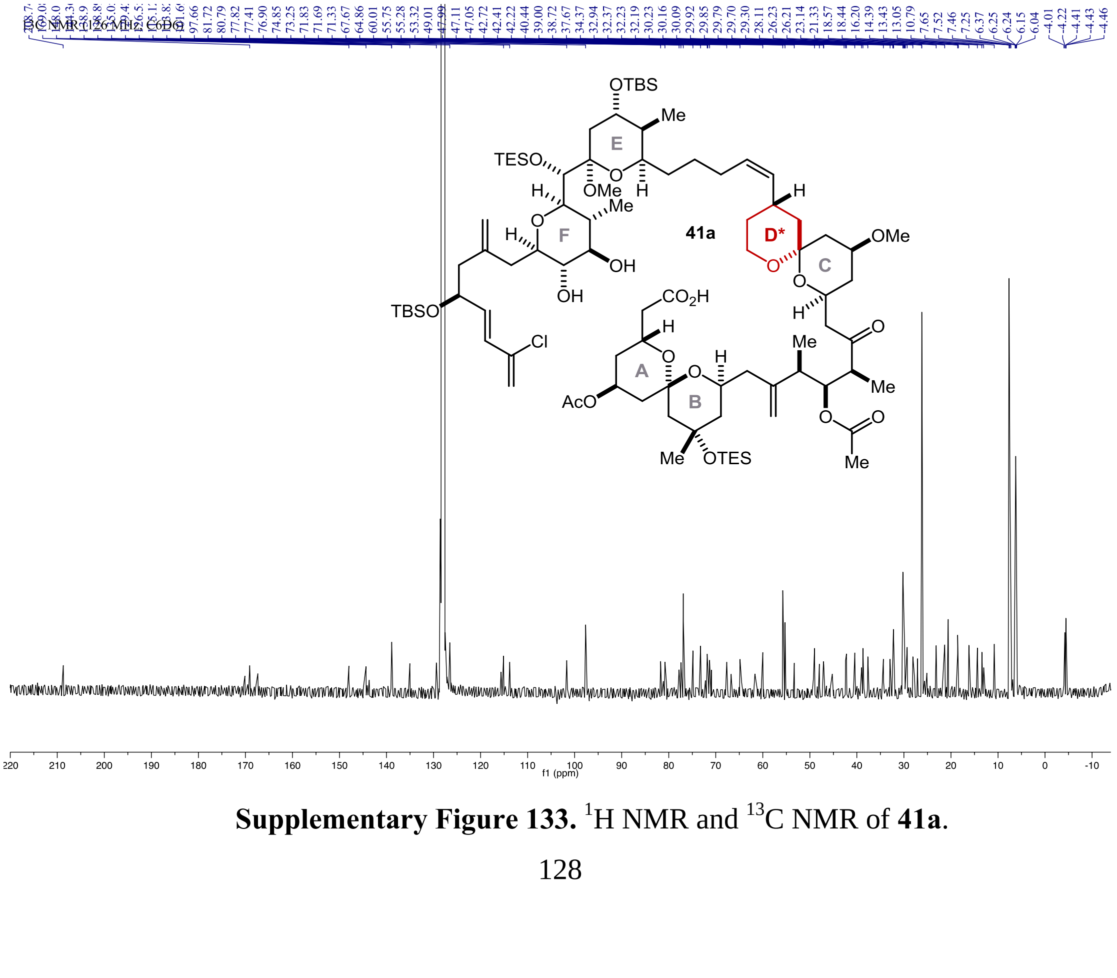

Supplementary Figure 133. ^{1}H NMR and ^{13}C NMR of **41a**.

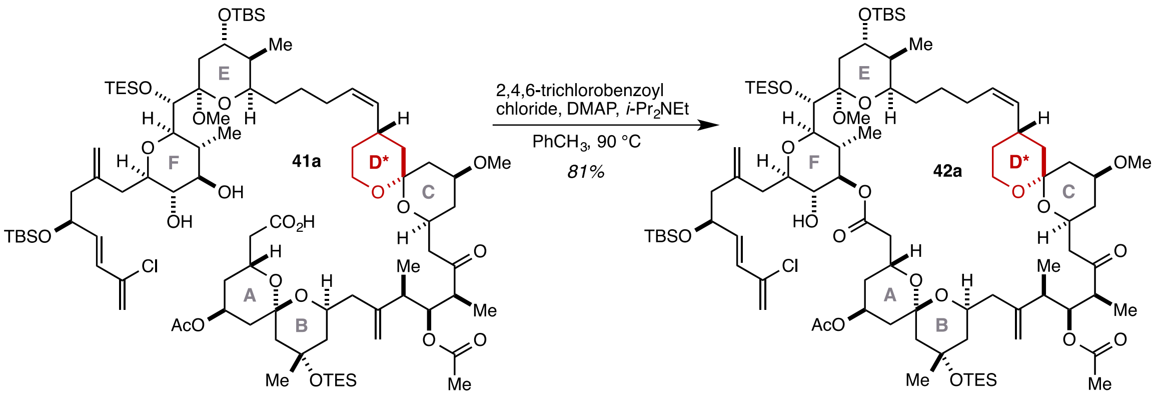

Supplementary Figure 134. Synthesis of **42a**.

Seco-acid **41a** (40 mg, 0.023 mmol) was dissolved in toluene (2.3 mL) and *i*-Pr_2NEt (0.24 mL, 1.38 mmol) and 2,4,6-trichlorobenzoyl chloride (72 µL, 0.46 mmol) were added. This was stirred for 4 h at rt, then diluted with toluene (6.9 mL). The mixture was taken up into a 12 mL gas-tight syringe and added to a 90 ºC solution of DMAP (141 mg, 1.15 mmol) in toluene (34 mL) over 24 h by syringe pump. The vial containing starting material which was sealed and stored at 0 ºC was rinsed with toluene (2.3 mL) and added to the reaction at over 8 h by syringe pump. The starting material vial was rinsed with an additional portion of toluene (2.3 mL) and added to the reaction over 4 h by syringe pump. The reaction mixture was allowed to stir at 90 ºC for 20 h. The reaction mixture was cooled to rt and diluted with Et_2O (50 mL). This was washed with $NaHCO_3$ (sat. aq) (50 mL) and brine (50 mL). The combined aqueous layers were extracted with EtOAc (2 x 50 mL). The combined organic layers were dried over Na_2SO_4, filtered and concentrated. Purification by flash column chromatography (10% → 40% EtOAc/Hexanes) on silica gel afforded lactone **42a** as a white solid (31 mg, 0.0186 mmol, 81% yield).

OR $[\alpha]_D^{24}$ +23.9° (*c* 1.00, CH_2Cl_2)

IR (ATR) 2928, 2876, 2856, 1735, 1647, 1583, 1550, 1461, 1370, 1246, 1143, 1110, 1029, 1005, 961, 891, 835, 775, 740 cm^{-1}

^{1}H NMR (500 MHz, C_6D_6) δ 6.55 – 6.26 (m, 3H), 5.70 (d, J = 10.4 Hz, 1H), 5.41 (q, J = 7.7 Hz, 2H), 5.23 – 4.93 (m, 14H), 4.84 (q, J = 15.7 Hz, 1H), 4.41 (dq, J = 22.9, 12.2, 9.5 Hz, 6H), 4.03 – 3.74 (m, 5H), 3.64 – 3.39 (m, 5H), 3.28 – 3.05 (m, 10H), 2.68 (s, 1H), 2.61 – 2.46 (m, 5H), 2.45 – 2.05 (m, 5H), 2.11 – 1.54 (m, 16H), 1.55 – 0.54 (m, 162H), 0.32 – -0.02 (m, 17H)

^{13}C NMR (126 MHz, C_6D_6) δ 208.8, 171.5, 170.3, 168.5, 148.2, 143.5, 142.8, 139.0, 138.9, 134.5, 130.0, 128.6, 126.5, 126.5, 126.4, 117.2, 116.2, 115.6, 115.2, 113.4, 101.8, 101.7, 97.7, 97.6, 97.1, 97.0, 80.9, 80.1, 79.7, 79.2, 78.9, 76.8, 75.0, 73.2, 73.1, 72.8, 72.3, 72.2, 71.7, 71.5, 71.3, 71.2, 70.4, 67.0, 65.4, 64.6, 63.5, 60.3, 55.7, 55.3, 51.4, 48.4, 48.1, 47.0, 46.6, 46.4, 45.7, 44.2, 42.5, 40.5, 40.3, 39.3, 39.0, 38.6, 38.4, 38.2, 37.8, 36.9, 36.1, 34.8, 33.4, 33.0, 32.8, 32.5, 32.4, 32.3, 30.6, 30.2, 29.9, 29.6, 27.7, 26.2, 26.1, 25.6, 25.1, 23.2, 21.5, 20.6, 18.5, 18.3, 14.6, 14.4, 13.9, 12.7, 10.8, 10.6, 7.7, 7.6, 7.2, 6.2, -4.1, -4.3, -4.5, -4.7

HRMS (ESI+) calculated for $C_{88}H_{153}ClO_{20}Si_4Na$ $[M+Na]^+$: 1699.9619, found 1699.9634

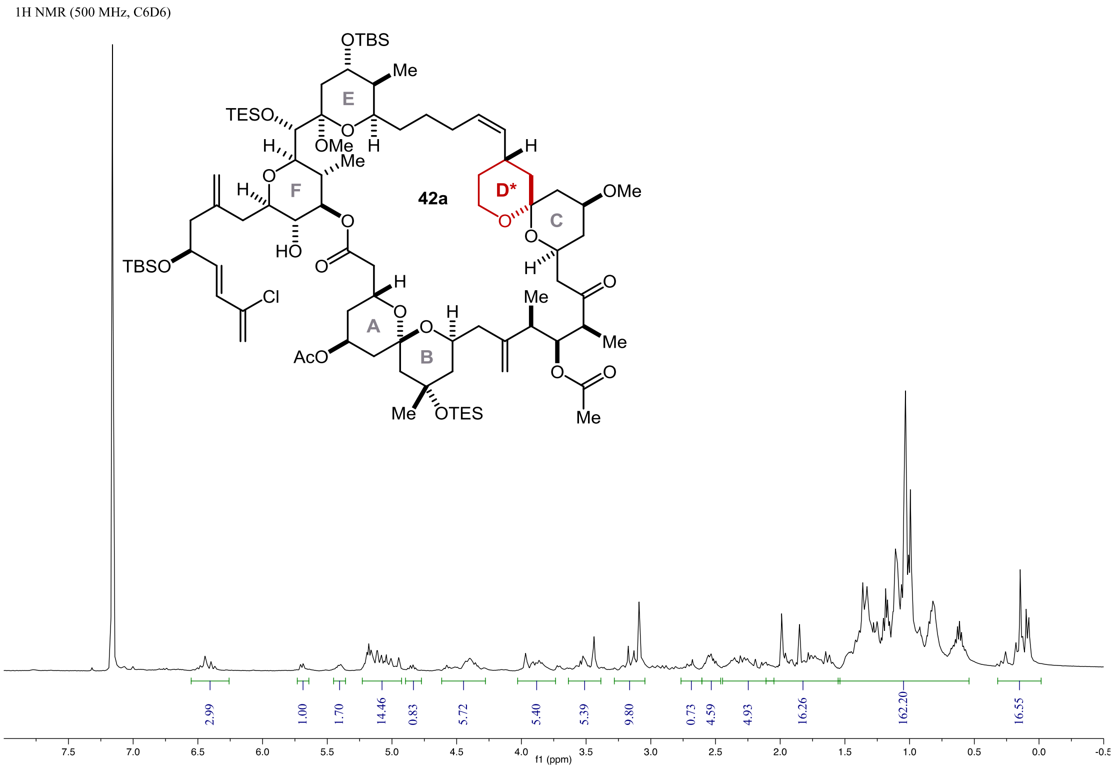

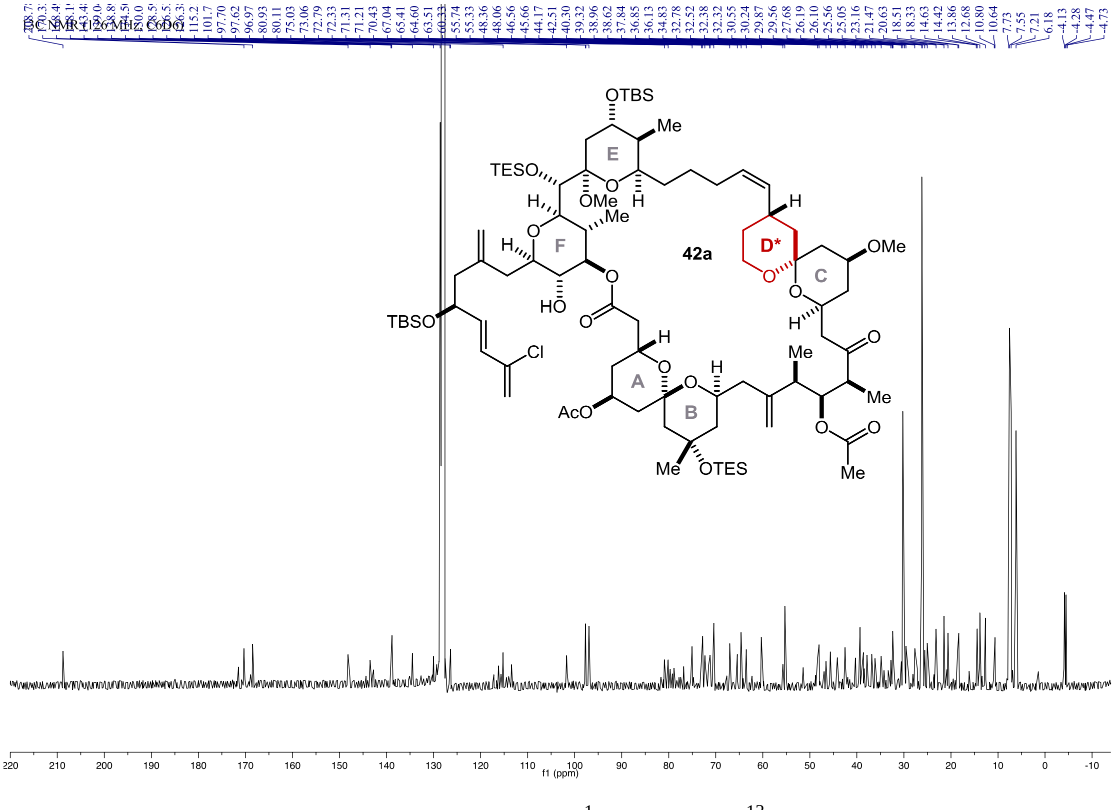

Supplementary Figure 135. ^{1}H NMR and ^{13}C NMR of **42a**.

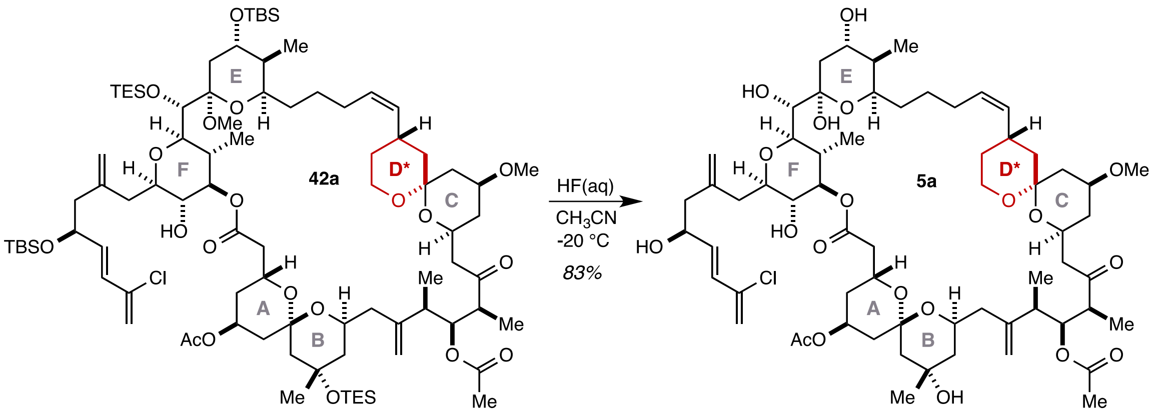

Supplementary Figure 136. Synthesis of **5a**.

Lactone **42a** (26 mg, 0.015 mmol) was dissolved in MeCN (1.08 mL) and cooled to -20 ºC. A freshly prepared solution of HF in MeCN (1.08 mL) [prepared by mixing 48% aqueous HF (1.25 mL) and MeCN (5 mL)] was added over 2 h by syringe pump at -20 ºC. The reaction was allowed to stir an additional 18 h at this temperature. The reaction was quenched by the dropwise addition of Et_3N (2.53 mL) and warmed to rt. The reaction mixture was diluted with a 2:1 mixture of EtOAc/CH_2Cl_2 (13 mL), then washed with $NaHCO_3$ (sat. aq) (13 mL) and brine (13 mL). The combined aqueous layers were extracted with a 2:1 mixture of EtOAc/CH_2Cl_2 (2 x 13 mL). The combined organic layers were dried over Na_2SO_4, filtered and concentrated. Purification by flash column chromatography (0% → 5% MeOH/CH_2Cl_2) on silica gel afforded spongistatin 1 analog **5a** as a white solid (15 mg, 0.0125 mmol, 83% yield).

OR $[\alpha]_D^{22}$ +18.2° (*c* 0.50, MeOH)

IR (ATR) 3435, 2926, 2851, 1735, 1653, 1580, 1551, 1436, 1384, 1233, 1177, 1093, 1059, 894 cm^{-1}

^{1}H NMR (500 MHz, CD_3CN) δ 6.50 – 6.34 (m, 1H), 6.25 – 6.06 (m, 1H), 5.45 (d, J = 6.9 Hz, 2H), 5.36 (s, 1H), 5.33 – 5.11 (m, 3H), 5.07 (s, 1H), 4.97 – 4.79 (m, 6H), 4.81 – 4.67 (m, 1H), 4.48 – 4.22 (m, 3H), 4.21 – 4.08 (m, 2H), 4.08 – 3.96 (m, 1H), 3.93 – 3.47 (m, 4H), 3.47 – 3.31 (m, 1H), 3.27 (d, J = 18.9 Hz, 6H), 3.19 – 3.03 (m, 1H), 3.03 – 2.69 (m, 3H), 2.66 – 2.47 (m, 1H), 2.44 – 2.30 (m, 1H), 2.30 – 2.22 (m, 2H), 1.87 – 1.78 (m, 4H), 1.73 – 1.45 (m, 6H), 1.28 (d, J = 13.7 Hz, 11H), 1.14 (dd, J = 7.0, 2.7 Hz, 3H), 1.09 – 0.97 (m, 7H), 0.88 (d, J = 7.1 Hz, 1H), 0.84 – 0.73 (m, 6H)

^{13}C NMR (126 MHz, CD_3CN) δ 210.7, 172.8, 171.6, 170.1, 148.1, 144.1, 139.2, 139.0, 135.8, 129.0, 126.9, 126.8, 99.3, 99.2, 99.1, 98.0, 81.1, 81.0, 80.3, 78.8, 78.6, 77.8, 74.9, 73.8, 73.2, 73.0, 71.5, 70.6, 69.4, 69.3, 69.2, 67.0, 64.7, 63.4, 62.6, 62.3, 60.7, 56.1, 55.5, 50.6, 48.1, 46.8, 45.1, 44.3, 44.0, 43.8, 42.9, 42.2, 40.4, 40.3, 40.1, 39.7, 39.6, 37.4, 37.3, 37.2, 36.8, 34.5, 33.8, 33.2, 32.6, 32.5, 30.4, 30.2, 30.1, 29.4, 27.7, 27.1, 23.4, 21.8, 21.0, 14.4, 13.1, 12.8, 12.2, 11.3

HRMS (ESI+) calculated for $C_{63}H_{95}ClO_{20}Na$ $[M+Na]^+$: 1229.6003, found 1229.5991

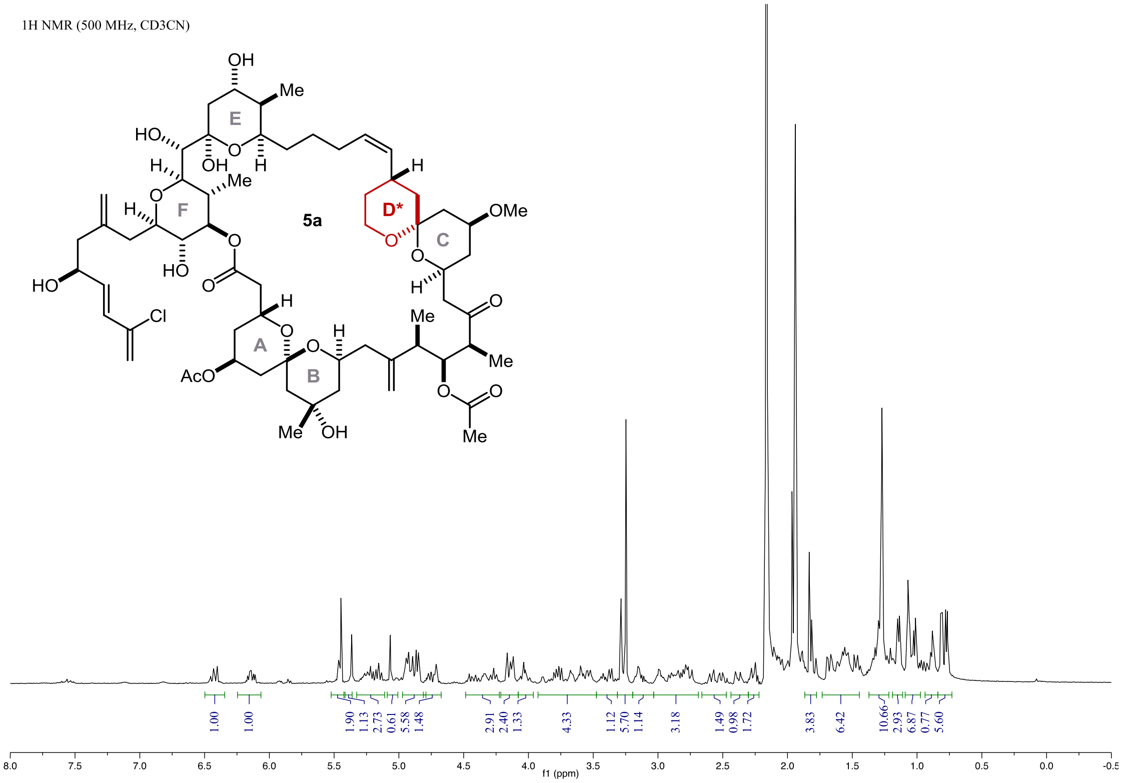

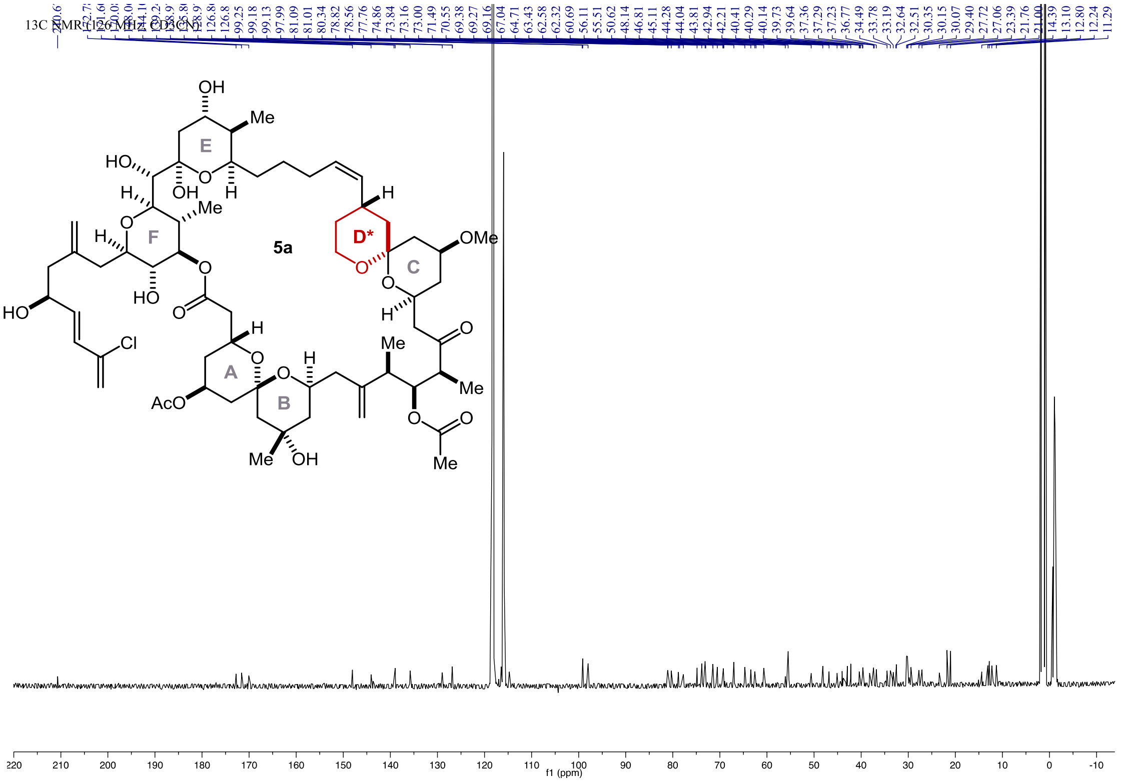

Supplementary Figure 137. ^{1}H NMR and ^{13}C NMR of **5a**.

Synthesis of **5b** from **7** and **6b**

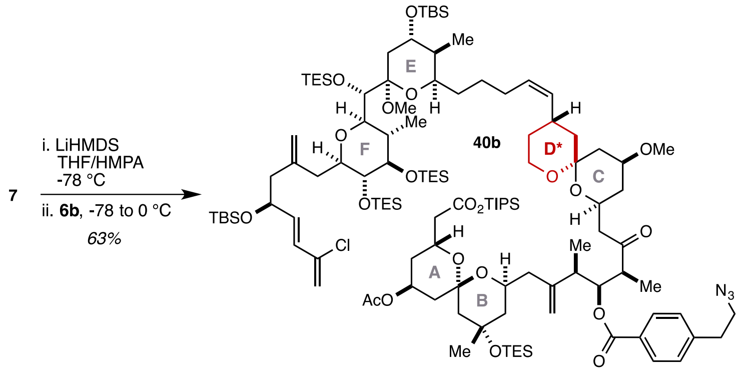

Supplementary Figure 138. Synthesis of **40b**.

Wittig salt **7** (45 mg, 0.03 mmol) was azeotroped with dry benzene (3x) and placed under vacuum for 24 h. Aldehyde **6b** (51 mg, 0.045 mmol) was azeotroped with dry benzene (3x) and placed under vacuum for 24 h. The dried Wittig salt was charged with dry 10% HMPA/THF solution (0.31 mL) and cooled to -78 ºC. LiHMDS (33 μL, 0.033 mmol) was added dropwise and the resultant orange solution was stirred 30 min at this temperature. A solution of the dried aldehyde in 10% HMPA/THF (0.23 mL) was added dropwise to the reaction mixture. The yellow solution was allowed to warm to 0 ºC over 1 h and stirred at 0 ºC for 1 h. The reaction was quenched by the addition of a 4:1 mixture of NH_4Cl (sat. aq.) and $Na_2S_2O_3$ (sat. aq.) (2.5 mL). Et_2O (3.5 mL) was added the layers were separated. The aqueous layer was extracted with Et_2O (3 x 2 mL) and the combined organic layers were washed with $NaHCO_3$ (sat. aq) (2.5 mL) and brine (2.5 mL), dried over $MgSO_4$, filtered and concentrated. Purification by flash column chromatography (10% → 40% EtOAc/Hexanes, then 0% → 10% MeOH/CH_2Cl_2) on pH 7 buffered silica gel afforded Wittig product **40b** as a white solid (42 mg, 0.019 mmol, 63% yield).

OR $[\alpha]_D^{21}$ +20.2° (*c* 1.00, CH_2Cl_2)

IR (ATR) 2952, 2876, 2098, 1729, 1461, 1415, 1382, 1246, 1109, 1005, 739 cm^{-1}

^{1}H NMR (500 MHz, C_6D_6) δ 8.14 (dd, *J* = 8.3, 3.5 Hz, 2H), 6.82 (d, *J* = 7.9 Hz, 2H), 6.47 (td, *J* = 15.0, 12.8, 4.7 Hz, 2H), 5.93 (dt, *J* = 8.7, 4.3 Hz, 1H), 5.41 (q, *J* = 7.8 Hz, 1H), 5.17 (h, *J* = 6.9, 5.4 Hz, 5H), 5.09 – 5.02 (m, 3H), 4.61 – 4.48 (m, 2H), 4.36 (d, *J* = 10.6 Hz, 1H), 4.27 (d, *J* = 8.5 Hz, 2H), 4.02 – 3.93 (m, 2H), 3.91 (d, *J* = 3.1 Hz, 1H), 3.68 (tt, *J* = 10.5, 4.6 Hz, 1H), 3.63 – 3.44 (m, 5H), 3.20 – 3.13 (m, 4H), 3.04 (s, 3H), 2.93 (dt, *J* = 15.6, 3.7 Hz, 1H), 2.89 – 2.66 (m, 6H), 2.60 – 2.46 (m, 4H), 2.35 (tdd, *J* = 16.8, 10.9, 4.6 Hz, 3H), 2.27 (t, *J* = 7.2 Hz, 2H), 2.23 – 2.03 (m, 4H), 1.96 (dd, *J* = 17.9, 10.7 Hz, 2H), 1.82 (d, *J* = 2.5 Hz, 4H), 1.80 – 1.59 (m, 7H), 1.51 (d, *J* = 13.0 Hz, 1H), 1.45 (q, *J* = 7.2, 5.7 Hz, 6H), 1.32 (p, *J* = 8.8, 7.2 Hz, 11H), 1.27 – 0.90 (m, 107H), 0.89 – 0.79 (m, 19H), 0.78 (s, 3H), 0.66 (dh, *J* = 14.6, 7.1 Hz, 10H), 0.26 (d, *J* = 4.3 Hz, 3H), 0.17 (d, *J* = 2.7 Hz, 7H), 0.12 (s, 3H)

^{13}C NMR (126 MHz, C_6D_6) δ 208.1, 170.3, 169.8, 164.6, 147.2, 144.0, 143.3, 138.5, 138.5, 134.7, 129.9, 129.1, 128.9, 128.7, 128.2, 126.1, 115.6, 115.2, 114.7, 114.0, 101.2, 97.2, 96.9, 81.3, 80.8, 80.4, 80.0, 77.6, 77.4, 77.0, 76.9, 75.4, 72.7, 71.8, 71.4, 71.2, 71.0, 70.9, 70.4, 67.3, 66.6, 64.5, 64.2, 61.4, 59.6, 54.8, 51.3, 48.6, 47.5, 46.6, 46.1, 45.2, 42.4, 42.1, 42.0, 41.9, 40.1, 39.5, 38.7, 38.4, 37.0, 36.5, 34.7, 34.1, 32.6, 31.8, 29.8, 28.9, 27.7, 26.8, 25.8, 25.8, 24.7, 20.9, 18.2, 18.0, 17.7, 15.8, 15.7, 13.1, 11.9, 10.5, 10.4, 7.4, 7.3, 7.1, 7.1, 7.0, 6.0, 5.9, 5.9, 5.8, 5.8, -4.4, -4.6, -4.8

HRMS (ESI+) calculated for $C_{116}H_{212}ClO_{21}Si_7N_4$ $[M+NH_4]^+$: 2228.3717, found 2228.3748

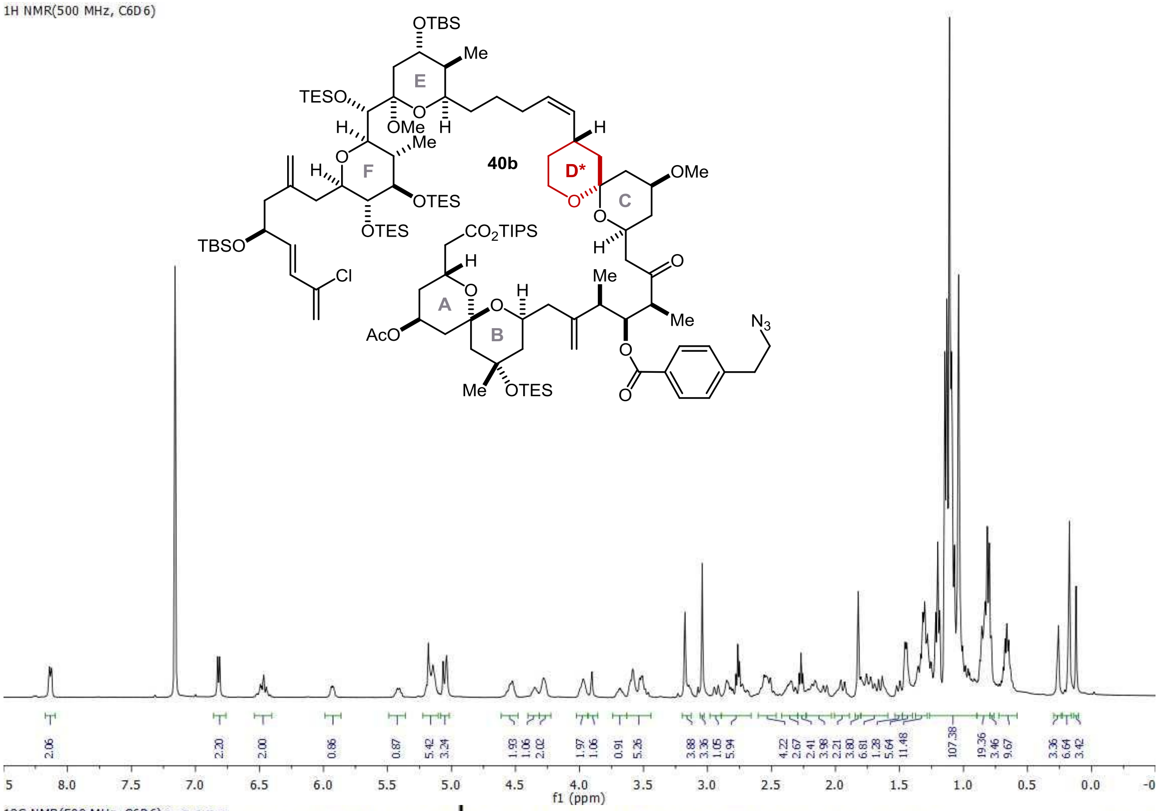

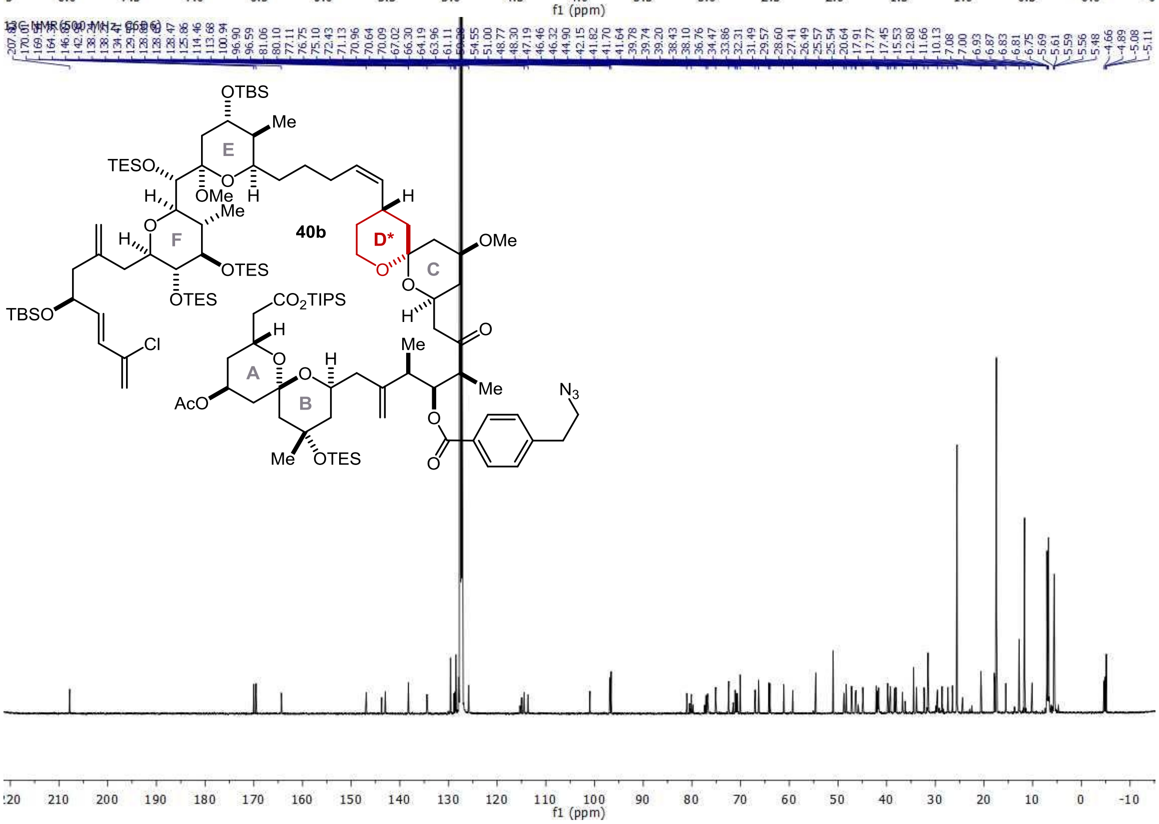

Supplementary Figure 139. ^{1}H NMR and ^{13}C NMR of **40b**.

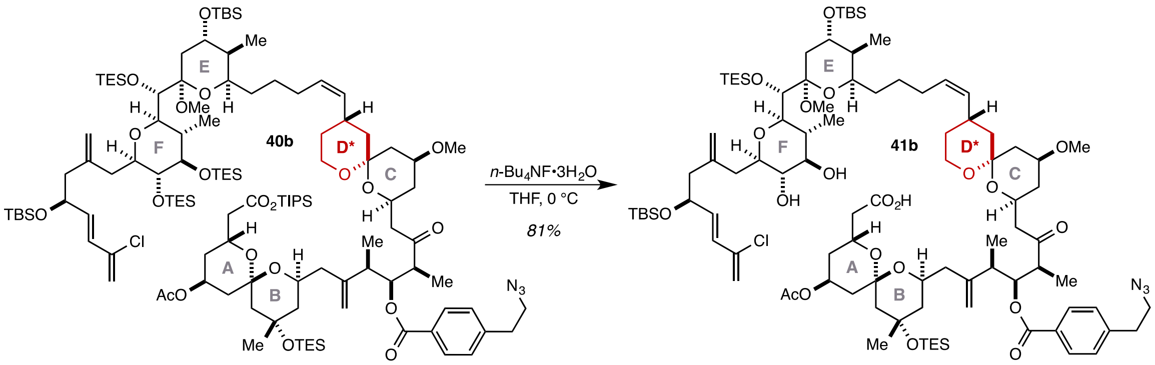

Supplementary Figure 140. Synthesis of **41b**.

Wittig product **40b** (35 mg, 0.016 mmol) was dissolved in THF (3.4 mL) and cooled to 0 ºC. TBAF•$3H_2O$ (47 μL, 1M in THF) was added in 6 portions over 1 h and stirring was continued at 0 ºC for 3 h. The reaction was quenched by the addition of NH_4Cl (sat. aq.) (3.5 mL) and EtOAc (3.5 mL). The layers were separated and the aqueous layer was extracted with EtOAc (3 x 3.5 mL). The combined organic layers were washed with brine (5 mL). The combined aqueous layers were back-extracted with CH_2Cl_2 (2 x 3.5 mL). The combined organic layers were dried over Na_2SO_4, filtered and concentrated. Purification by flash column chromatography (0% → 5% MeOH/CH_2Cl_2) on silica gel afforded *seco*-acid **41b** as a white solid (23 mg, 0.013 mmol, 81% yield).

OR $[\alpha]_D^{23}$ +21.5° (*c* 1.00, CH_2Cl_2)

IR (ATR) 3439, 2952, 2928, 2876, 2854, 2099, 1729, 1553, 1461, 1382, 1248, 1179, 1144, 1101, 1005, 835, 775, 739 cm^{-1}

^{1}H NMR (500 MHz, C_6D_6) δ 8.15 (d, *J* = 7.9 Hz, 2H), 6.86 – 6.80 (m, 2H), 6.55 – 6.33 (m, 2H), 5.94 (s, 1H), 5.42 (s, 1H), 5.24 – 5.04 (m, 7H), 5.01 (s, 1H), 4.95 (s, 1H), 4.57 – 4.40 (m, 2H), 4.33 (d, *J* = 29.6 Hz, 3H), 4.02 – 3.85 (m, 3H), 3.69 (s, 1H), 3.60 (d, *J* = 11.1 Hz, 2H), 3.53 (d, *J* = 10.1 Hz, 1H), 3.39 (d, *J* = 11.4 Hz, 1H), 3.28 – 3.12 (m, 6H), 3.11 – 2.97 (m, 5H), 2.86 (d, *J* = 27.3 Hz, 2H), 2.77 (t, *J* = 7.0 Hz, 3H), 2.70 (s, 1H), 2.64 – 2.42 (m, 5H), 2.35 (d, *J* = 15.5 Hz, 3H), 2.27 (s, 3H), 2.21 – 2.14 (m, 2H), 1.89 (t, *J* = 10.6 Hz, 2H), 1.83 (s, 4H), 1.74 (t, *J* = 14.1 Hz, 4H), 1.63 (d, *J* = 14.1 Hz, 3H), 1.53 – 1.39 (m, 8H), 1.36 (s, 8H), 1.34 – 1.28 (m, 10H), 1.25 – 1.14 (m, 15H), 1.14 – 1.01 (m, 53H), 0.98 (s, 3H), 0.95 – 0.84 (m, 9H), 0.84 – 0.77 (m, 10H), 0.68 (q, *J* = 7.9 Hz, 9H), 0.29 – 0.23 (m, 3H), 0.21 – 0.09 (m, 10H)

^{13}C NMR (126 MHz, C_6D_6) δ 209.2, 170.8, 170.5, 167.7, 165.5, 165.4, 147.9, 147.8, 144.7, 144.2, 144.1, 144.1, 143.4, 139.4, 139.3, 139.2, 135.4, 130.6, 129.8, 129.7, 129.5, 128.9, 126.9, 126.8, 116.1, 116.0, 115.5, 114.9, 102.1, 102.0, 97.9, 82.0, 81.1, 77.8, 77.2, 76.2, 73.5, 73.4, 72.6, 72.1, 72.0, 71.7, 71.2, 68.0, 67.2, 65.1, 65.0, 64.8, 62.0, 60.3, 56.1, 55.5, 55.5, 52.0, 49.5, 49.4, 48.3, 47.4, 46.8, 45.6, 42.6, 40.8, 39.9, 39.4, 39.3, 39.0, 37.7, 35.5, 34.7, 33.3, 33.1, 32.7, 32.6, 30.6, 30.2, 29.6, 28.4, 28.2, 28.1, 27.4, 27.1, 26.6, 23.5, 21.7, 21.6, 18.9, 18.8, 16.5, 14.7, 14.3, 13.9, 13.6, 11.2, 11.1, 8.0, 8.0, 8.0, 7.9, 7.8, 7.6, 7.6, 6.7, 6.6, 6.6, 1.8, -3.7, -3.9, -4.1, -4.1

HRMS (ESI+) calculated for $C_{95}H_{164}ClO_{21}Si_4N_4$ $[M+NH_4]^+$: 1844.0654, found 1844.0664

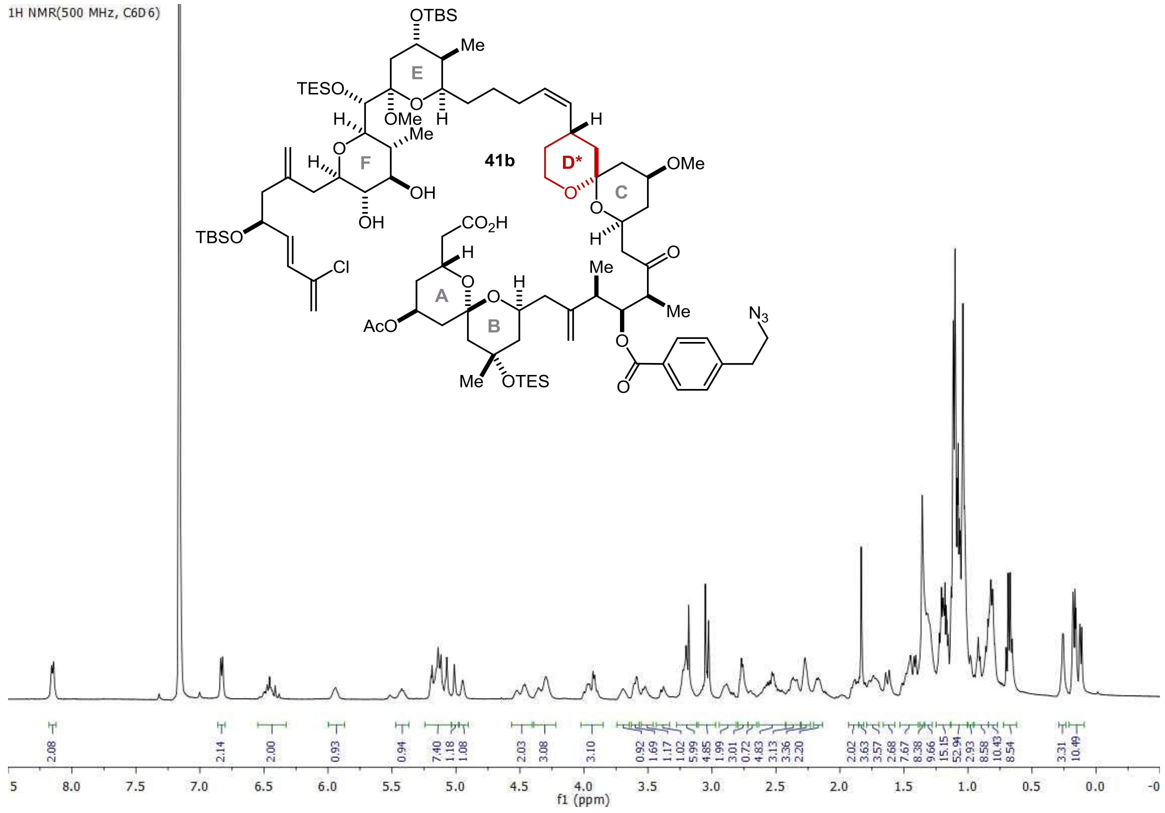

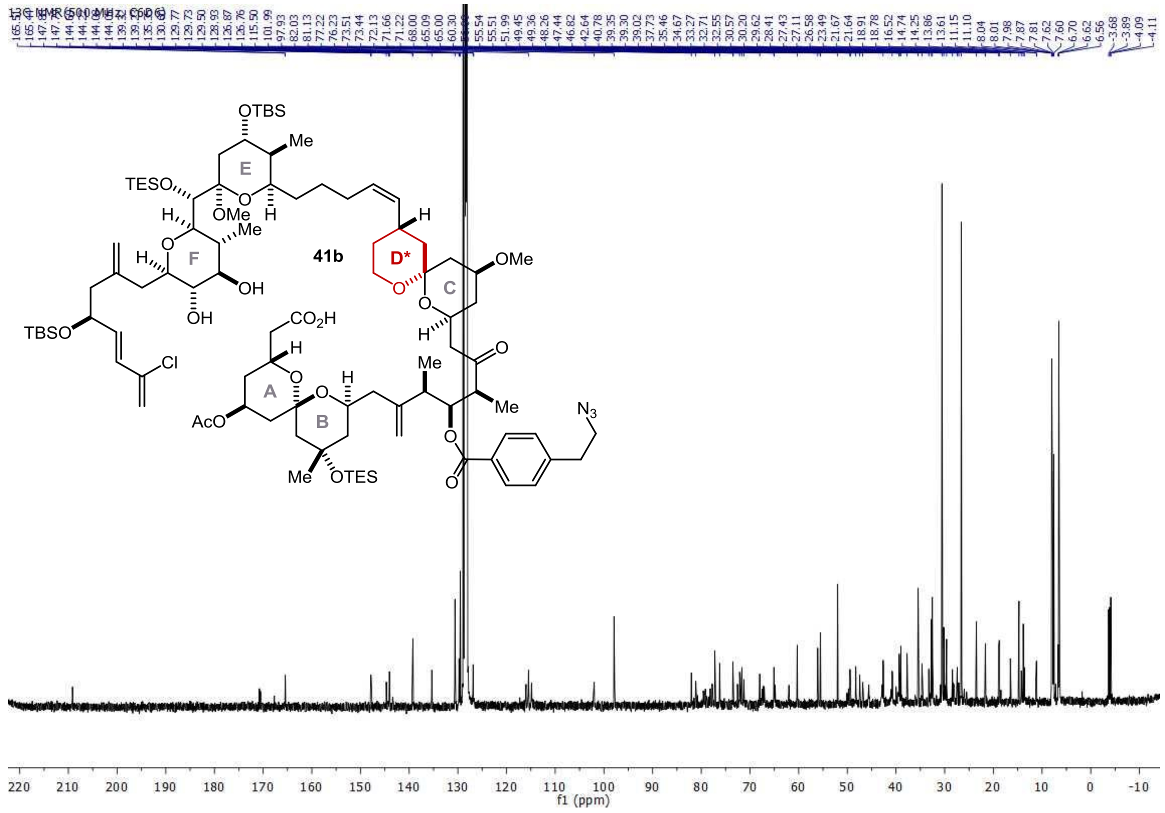

Supplementary Figure 141. ^{1}H NMR and ^{13}C NMR of **41b**.

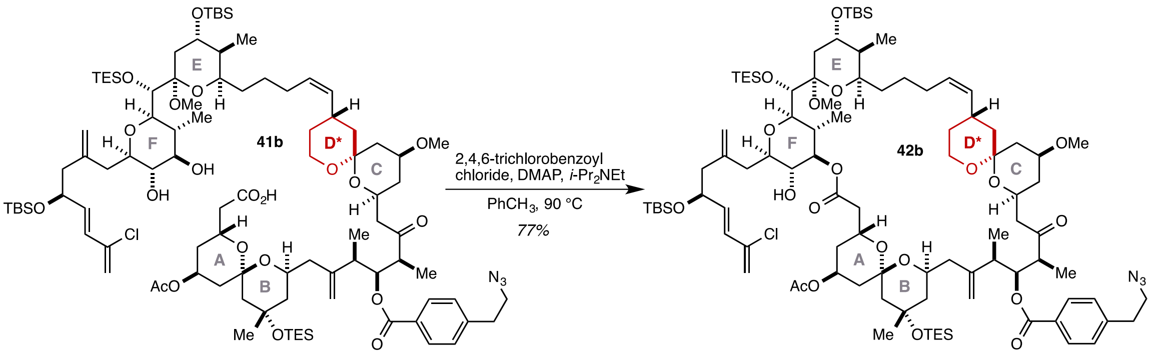

Supplementary Figure 142. Synthesis of **42b**.

Seco-acid **41b** (21 mg, 0.011 mmol) was dissolved in toluene (1.1 mL) and *i*-Pr_2NEt (115 μL, 0.66 mmol) and 2,4,6-trichlorobenzoyl chloride (34 μL, 0.22 mmol) were added. This was stirred for 4 h at rt, then diluted with toluene (3.3 mL). The mixture was taken up into a 6 mL gas-tight syringe and added to a 90 ºC solution of DMAP (67 mg, 0.55 mmol) in toluene (16 mL) over 24 h by syringe pump. The vial containing starting material which was sealed and stored at 0 ºC was rinsed with toluene (1.1 mL) and added to the reaction at over 8 h by syringe pump. The starting material vial was rinsed with an additional portion of toluene (1.1 mL) and added to the reaction over 4 h by syringe pump. The reaction mixture was allowed to stir at 90 ºC for 20 h. The reaction mixture was cooled to rt and diluted with Et_2O (25 mL). This was washed with $NaHCO_3$ (sat. aq) (25 mL) and brine (25 mL). The combined aqueous layers were extracted with EtOAc (2 x 25 mL). The combined organic layers were dried over Na_2SO_4, filtered and concentrated. Purification by flash column chromatography (10% → 40% EtOAc/Hexanes) on silica gel afforded lactone **42b** as a white solid (15 mg, 0.0085 mmol, 77% yield).

OR $[\alpha]_D^{25}$ +16.9° (*c* 1.00, CH_2Cl_2)

IR (ATR) 2927, 2881, 2859, 2097, 1731, 1650, 1582, 1546, 1462, 1380, 1250, 1143, 1109, 1030, 835, 742 cm^{-1}

^{1}H NMR (500 MHz, C_6D_6) δ 8.25 – 8.09 (m, 2H), 6.83 (d, *J* = 8.2 Hz, 2H), 6.53 – 6.33 (m, 2H), 6.03 (d, *J* = 10.4 Hz, 1H), 5.49 – 5.29 (m, 1H), 5.07 (ddq, *J* = 48.4, 24.6, 9.4, 8.3 Hz, 8H), 4.67 (d, *J* = 10.7 Hz, 1H), 4.54 – 4.23 (m, 4H), 4.03 – 3.79 (m, 3H), 3.69 (d, *J* = 6.0 Hz, 1H), 3.63 (d, *J* = 10.3 Hz, 1H), 3.58 (s, 1H), 3.51 (d, *J* = 3.5 Hz, 2H), 3.42 (s, 1H), 3.27 (d, *J* = 4.0 Hz, 1H), 3.18 (d, *J* = 1.9 Hz, 1H), 3.11 – 3.00 (m, 2H), 2.98 – 2.88 (m, 1H), 2.87 – 2.68 (m, 5H), 2.65 – 2.38 (m, 4H), 2.32 – 2.21 (m, 4H), 2.19 – 2.08 (m, 3H), 2.01 (d, *J* = 18.5 Hz, 1H), 1.84 (d, *J* = 6.1 Hz, 3H), 1.73 – 1.55 (m, 4H), 1.44 (ddd, *J* = 7.3, 4.6, 2.6 Hz, 5H), 1.38 – 1.28 (m, 5H), 1.28 – 1.15 (m, 11H), 1.15 – 0.90 (m, 43H), 0.84 (dq, *J* = 15.9, 7.6 Hz, 13H), 0.71 – 0.54 (m, 9H), 0.28 (d, *J* = 12.3 Hz, 2H), 0.21 – 0.01 (m, 12H).

^{13}C NMR: We were unable to generate a useful ^{13}C NMR spectrum for this compound due to a lack of a minimally sufficient amount of material.

HRMS (ESI+) calculated for $C_{95}H_{162}ClO_{20}Si_4N_4$ $[M+NH_4]^+$: 1826.0548, found 1826.0526

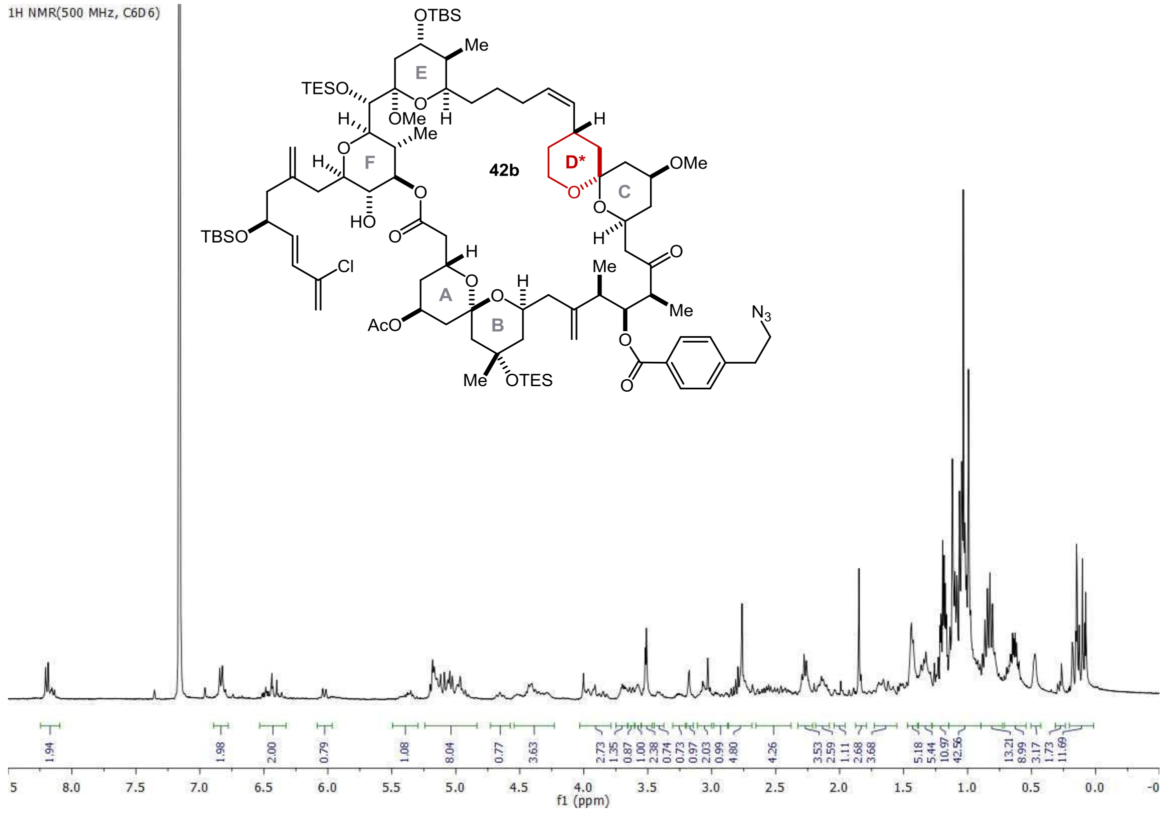

Supplementary Figure 143. ^{1}H NMR of **42b**.

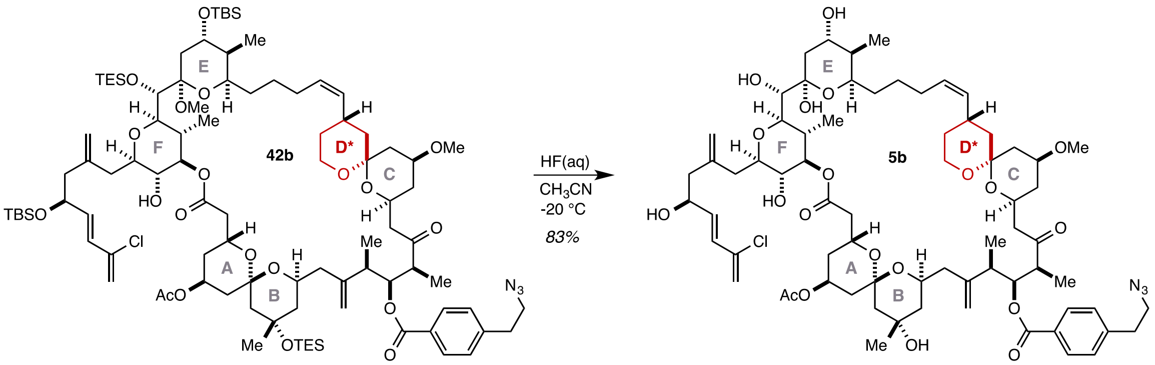

Supplementary Figure 144. Synthesis of **5b**.

Lactone **42b** (13 mg, 0.0072 mmol) was dissolved in MeCN (0.52 mL) and cooled to -20 ºC. A freshly prepared solution of HF in MeCN (0.52 mL) [prepared by mixing 48% aqueous HF (1.25 mL) and MeCN (5 mL)] was added over 2 h by syringe pump at -20 ºC. The reaction was allowed to stir an additional 18 h at this temperature. The reaction was quenched by the dropwise addition of Et_3N (1.21 mL) and warmed to rt. The reaction mixture was diluted with a 2:1 mixture of EtOAc/CH_2Cl_2 (6 mL), then washed with $NaHCO_3$ (sat. aq) (6 mL) and brine (6 mL). The combined aqueous layers were extracted with a 2:1 mixture of EtOAc/CH_2Cl_2 (2 x 6 mL). The combined organic layers were dried over Na_2SO_4, filtered and concentrated. Purification by flash column chromatography (0% → 5% MeOH/CH_2Cl_2) on silica gel afforded spongistatin 1 analog **5b** as a white solid (8 mg, 0.006 mmol, 83% yield).

OR $[\alpha]_D^{22}$ +18.2° (*c* 0.50, MeOH)

IR (ATR) 3435, 2926, 2851, 1735, 1653, 1580, 1551, 1436, 1384, 1233, 1177, 1093, 1059, 894 cm^{-1}

^{1}H NMR (500 MHz, CD_3CN) δ 7.98 – 7.81 (m, 2H), 7.43 – 7.33 (m, 2H), 6.50 – 6.42 (m, 1H), 6.22 – 6.14 (m, 1H), 5.55 – 5.45 (m, 2H), 5.39 (d, *J* = 4.3 Hz, 1H), 5.29 (dd, *J* = 10.8, 6.4 Hz, 1H), 5.17 (t, *J* = 10.6 Hz, 1H), 4.95 (d, *J* = 12.8 Hz, 2H), 4.87 – 4.78 (m, 2H), 4.76 (d, *J* = 4.9 Hz, 1H), 4.37 (s, 1H), 4.31 (t, *J* = 11.1 Hz, 1H), 4.23 – 4.07 (m, 2H), 4.00 (t, *J* = 10.4 Hz, 2H), 3.85 – 3.77 (m, 1H), 3.71 (s, 1H), 3.59 (t, *J* = 6.9 Hz, 2H), 3.50 (d, *J* = 25.7 Hz, 2H), 3.42 (d, *J* = 10.2 Hz, 1H), 3.30 – 3.26 (m, 1H), 2.96 (t, *J* = 6.9 Hz, 2H), 2.91 – 2.73 (m, 3H), 2.60 (d, *J* = 9.3 Hz, 1H), 2.55 (d, *J* = 11.2 Hz, 1H), 2.46 – 2.31 (m, 2H), 1.79 (s, 2H), 1.71 (d, *J* = 4.3 Hz, 2H), 1.58 (d, *J* = 12.8 Hz, 4H), 1.50 (d, *J* = 14.5 Hz, 2H), 1.41 (d, *J* = 6.1 Hz, 1H), 1.38 (s, 3H), 1.30 (s, 7H), 1.26 – 1.15 (m, 8H), 1.11 (s, 3H), 1.06 – 0.99 (m, 3H), 0.90 (q, *J* = 6.9 Hz, 3H), 0.84 (t, *J* = 5.6 Hz, 3H)

^{13}C NMR: We were unable to generate a useful ^{13}C NMR spectrum for this compound due to a lack of a minimally sufficient amount of material.

HRMS (ESI+) calcd for $C_{70}H_{100}ClO_{20}N_3Na$ $[M+Na]^+$: 1360.6486, found 1360.6436

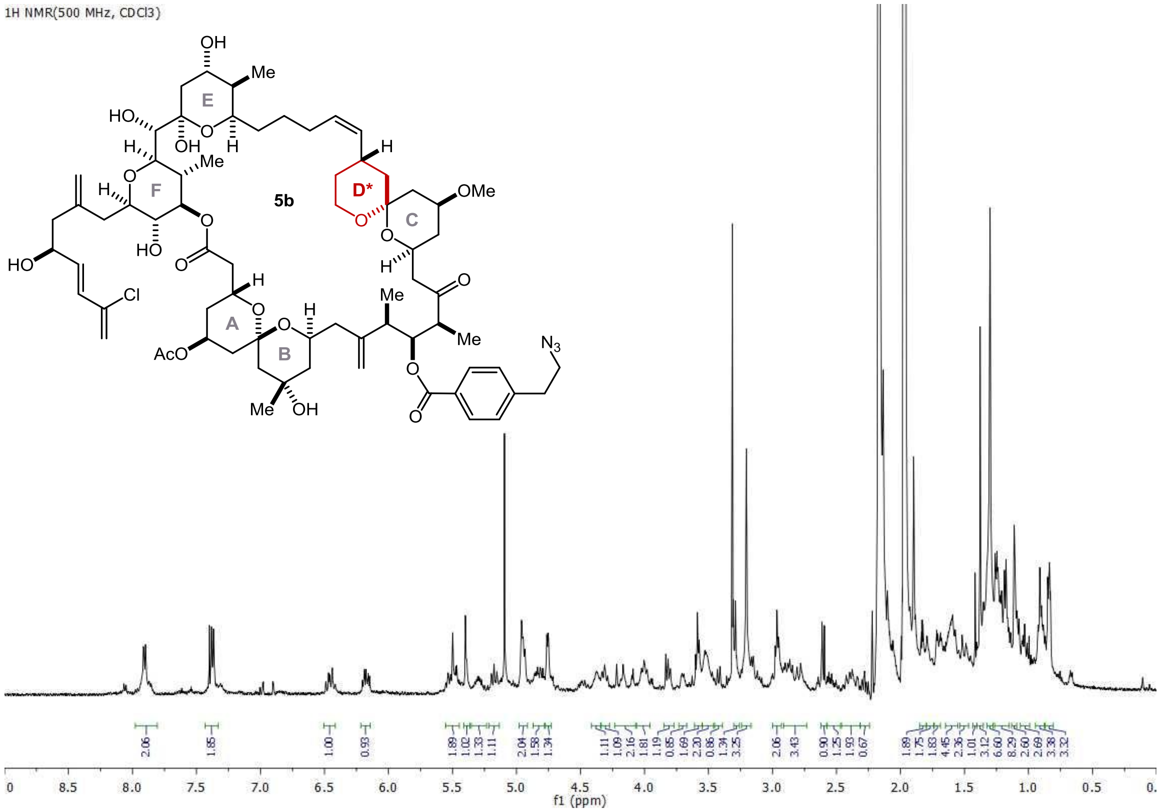

Supplementary Figure 145. ^{1}H NMR **5b**.

Synthesis of **5c** from **7** and **6c**

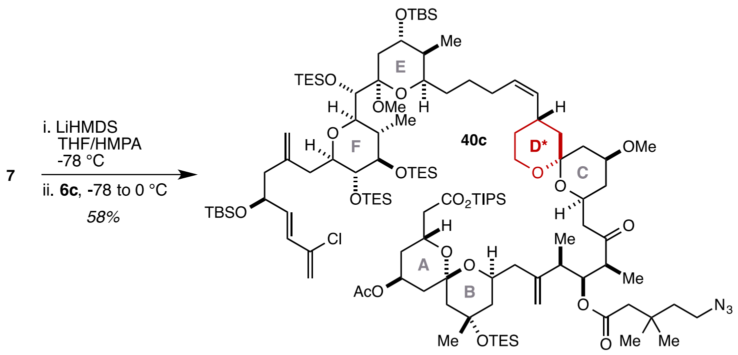

Supplementary Figure 146. Synthesis of **40c**.

Wittig salt **7** (35 mg, 0.023 mmol) was azeotroped with dry benzene (3x) and placed under vacuum for 24 h. Aldehyde **6c** (38 mg, 0.035 mmol) was azeotroped with dry benzene (3x) and placed under vacuum for 24 h. The dried Wittig salt was charged with dry 10% HMPA/THF solution (0.24 mL) and cooled to -78 ºC. LiHMDS (25 µL, 0.025 mmol) was added dropwise and the resultant orange solution was stirred 30 min at this temperature. A solution of the dried aldehyde in 10% HMPA/THF (0.18 mL) was added dropwise to the reaction mixture. The yellow solution was allowed to warm to 0 ºC over 1 h and stirred at 0 ºC for 1 h. The reaction was quenched by the addition of a 4:1 mixture of NH_4Cl (sat. aq.) and $Na_2S_2O_3$ (sat. aq.) (2.5 mL). Et_2O (3.5 mL) was added the layers were separated. The aqueous layer was extracted with Et_2O (3 x 2 mL) and the combined organic layers were washed with $NaHCO_3$ (sat. aq) (2.5 mL) and brine (2.5 mL), dried over $MgSO_4$, filtered and concentrated. Purification by flash column chromatography (10% → 40% EtOAc/Hexanes, then 0% → 10% MeOH/CH_2Cl_2) on pH 7 buffered silica gel afforded Wittig product **40c** as a white solid (29 mg, 0.013 mmol, 58% yield).

OR $[\alpha]_D^{22}$ +11.7° (*c* 0.50, CH_2Cl_2)

IR (ATR) 2950, 2932, 2873, 2095, 1734, 1462, 1370, 1243, 1182, 1076, 1003, 739 cm^{-1}

^{1}H NMR (500 MHz, C_6D_6) δ 6.55 – 6.40 (m, 2H), 5.69 (dd, *J* = 9.1, 3.3 Hz, 1H), 5.49 – 5.40 (m, 1H), 5.27 (s, 1H), 5.22 – 5.11 (m, 4H), 5.07 (d, *J* = 7.1 Hz, 3H), 4.52 (p, *J* = 6.0, 4.9 Hz, 2H), 4.42 (q, *J* = 6.5 Hz, 1H), 4.30 (d, *J* = 7.8 Hz, 2H), 4.07 – 3.94 (m, 2H), 3.91 (s, 1H), 3.85 (tt, *J* = 10.8, 4.6 Hz, 1H), 3.64 (dt, *J* = 11.3, 5.6 Hz, 1H), 3.61 – 3.54 (m, 2H), 3.54 – 3.45 (m, 2H), 3.18 (d, *J* = 1.2 Hz, 3H), 3.16 (s, 3H), 3.08 (t, *J* = 10.6 Hz, 1H), 2.97 (dd, *J* = 9.1, 7.0 Hz, 1H), 2.94 – 2.80 (m, 4H), 2.78 – 2.64 (m, 3H), 2.64 – 2.48 (m, 4H), 2.45 (dd, *J* = 15.2, 4.0 Hz, 1H), 2.42 – 2.31 (m, 3H), 2.31 – 2.16 (m, 3H), 2.16 – 2.07 (m, 2H), 2.05 (s, 2H), 2.01 – 1.91 (m, 6H), 1.76 (t, *J* = 13.9 Hz, 4H), 1.64 (td, *J* = 14.3, 4.7 Hz, 3H), 1.56 – 1.44 (m, 6H), 1.44 – 1.25 (m, 18H), 1.21 (q, *J* = 8.0 Hz, 15H), 1.16 – 0.96 (m, 84H), 0.94 – 0.76 (m, 28H), 0.65 (pd, *J* = 7.9, 6.0 Hz, 8H), 0.49 (s, 2H), 0.30 – 0.22 (m, 3H), 0.22 – 0.14 (m, 6H), 0.12 (s, 3H)

^{13}C NMR (126 MHz, C_6D_6) δ 207.9, 170.3, 169.8, 169.5, 147.6, 144.0, 143.3, 138.6, 138.5, 138.5, 138.5, 134.7, 129.0, 126.1, 115.6, 115.3, 114.8, 114.8, 113.6, 101.2, 97.3, 96.9, 81.4, 80.8, 80.4, 77.6, 77.4, 77.0, 76.9, 73.9, 72.9, 71.8, 71.4, 71.2, 70.9, 70.4, 67.3, 66.6, 64.5, 64.2, 61.4, 59.7, 55.0, 48.7, 48.6, 47.5, 47.3, 46.7, 46.6, 45.2, 45.2, 42.4, 42.2, 42.1, 41.9, 40.1, 39.4, 38.8, 38.7, 38.4, 37.5, 34.2, 32.6, 31.8, 31.7, 29.9, 29.9, 28.9, 27.7, 27.1, 27.0, 26.8, 25.8, 21.0, 18.2, 18.0, 17.7, 15.8, 15.7, 13.0, 12.6, 11.9, 10.5, 10.4, 7.4, 7.3, 7.2, 7.1, 7.0, 5.9, 5.8, 5.8, -4.4, -4.6, -4.8, -4.9

HRMS (ESI+) calculated for $C_{114}H_{216}ClO_{21}Si_7N_4$ $[M+NH_4]^+$: 2208.4030, found 2228.3748

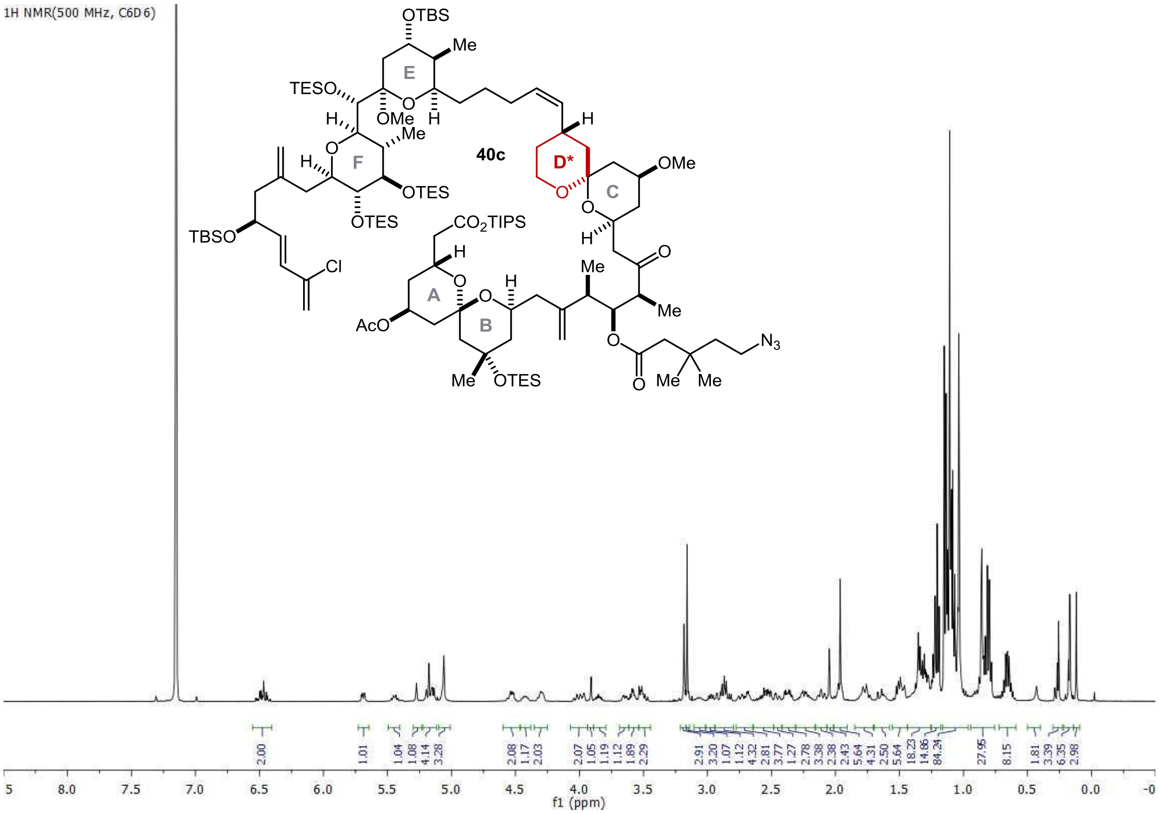

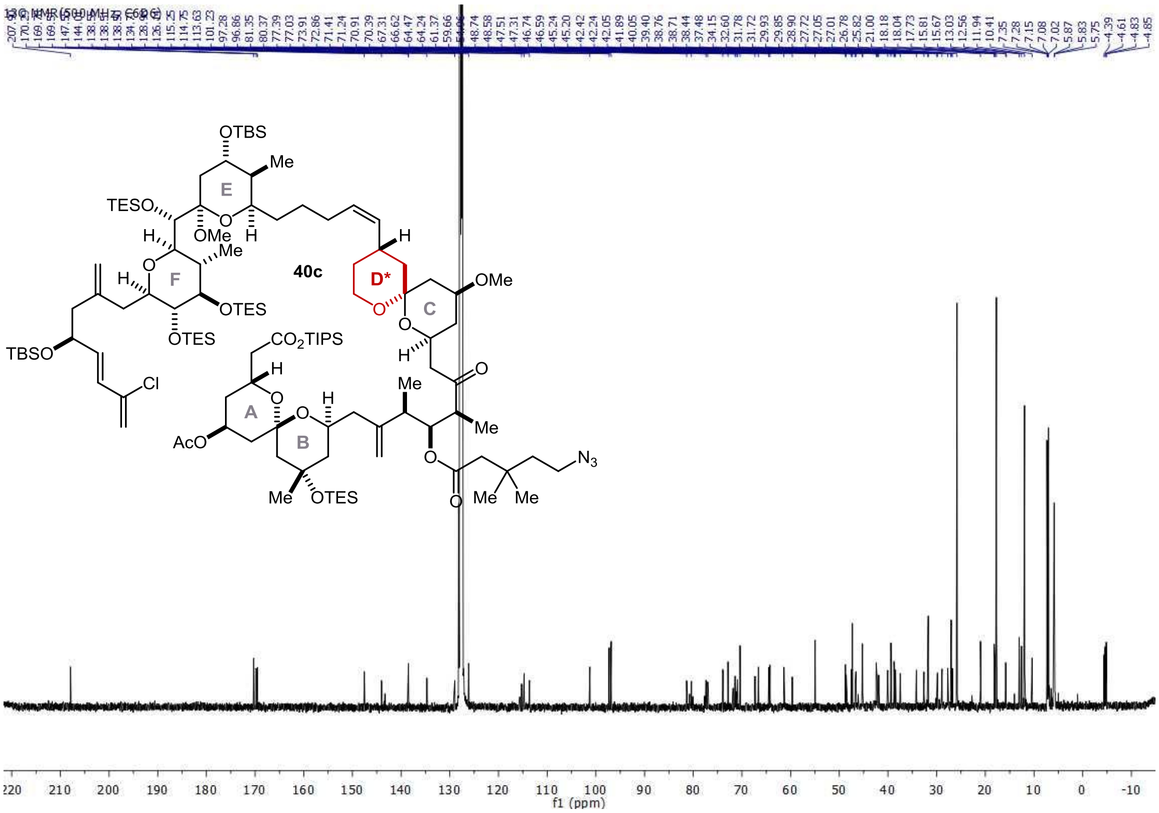

Supplementary Figure 147. ^{1}H NMR and ^{13}C NMR of **40c**.

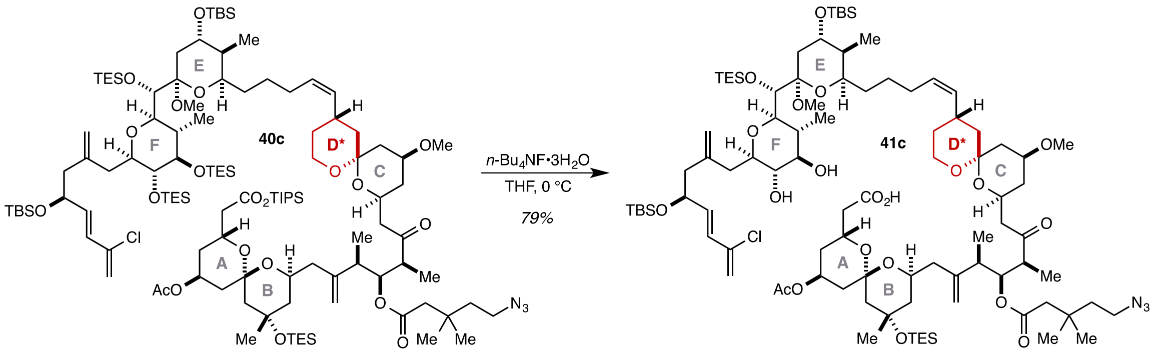

Supplementary Figure 148. Synthesis of **41c**.

Wittig product **40c** (20 mg, 0.0091 mmol) was dissolved in THF (1.95 mL) and cooled to 0 ºC. TBAF•3H_2O (27.3 μL, 1M in THF) was added in 6 portions over 1 h and stirring was continued at 0 ºC for 3 h. The reaction was quenched by the addition of NH_4Cl (sat. aq.) (2 mL) and EtOAc (2 mL). The layers were separated and the aqueous layer was extracted with EtOAc (3 x 2 mL). The combined organic layers were washed with brine (5 mL). The combined aqueous layers were back-extracted with CH_2Cl_2 (2 x 2 mL). The combined organic layers were dried over Na_2SO_4, filtered and concentrated. Purification by flash column chromatography (0% → 5% MeOH/CH_2Cl_2) on silica gel afforded *seco*-acid **41c** as a white solid (13 mg, 0.0072 mmol, 79% yield).

OR $[\alpha]_D^{26}$ +41.5° (*c* 1.00, CH_2Cl_2)

IR (ATR) 3449, 2953, 2930, 2876, 2095, 1734, 1553, 1461, 1383, 1246, 1181, 1144, 1108, 1005, 959, 835, 775, 741 cm^{-1}

^{1}H NMR (500 MHz, C_6D_6) δ 6.55 – 6.31 (m, 2H), 5.69 (t, *J* = 9.7 Hz, 1H), 5.55 – 5.37 (m, 1H), 5.27 – 4.90 (m, 10H), 4.52 (s, 1H), 4.47 – 4.37 (m, 3H), 4.30 (s, 2H), 3.97 (t, *J* = 10.7 Hz, 1H), 3.92 (s, 1H), 3.85 (tt, *J* = 10.7, 4.6 Hz, 1H), 3.63 (dd, *J* = 11.1, 5.3 Hz, 1H), 3.59 – 3.46 (m, 1H), 3.42 – 3.31 (m, 1H), 3.22 (dd, *J* = 14.1, 3.4 Hz, 3H), 3.18 – 3.11 (m, 6H), 3.09 – 2.95 (m, 3H), 2.85 (dt, *J* = 14.7, 7.5 Hz, 3H), 2.78 – 2.63 (m, 3H), 2.63 – 2.51 (m, 2H), 2.47 (dd, *J* = 15.6, 7.6 Hz, 3H), 2.36 (dt, *J* = 16.7, 6.9 Hz, 2H), 2.24 (dd, *J* = 12.3, 4.6 Hz, 3H), 2.11 (d, *J* = 13.7 Hz, 1H), 2.05 (dd, *J* = 7.4, 4.9 Hz, 2H), 1.99 – 1.93 (m, 4H), 1.90 (t, *J* = 14.4 Hz, 2H), 1.82 – 1.67 (m, 4H), 1.61 (t, *J* = 12.0 Hz, 3H), 1.49 (ddd, *J* = 23.6, 15.8, 6.9 Hz, 6H), 1.40 – 1.25 (m, 18H), 1.25 – 1.13 (m, 15H), 1.12 – 0.94 (m, 54H), 0.94 – 0.71 (m, 28H), 0.66 (qd, *J* = 7.8, 3.1 Hz, 11H), 0.25 (dd, *J* = 7.2, 4.0 Hz, 4H), 0.19 – 0.07 (m, 11H)

^{13}C NMR: We were unable to generate a useful ^{13}C NMR spectrum for this compound due to a lack of a minimally sufficient amount of material.

HRMS (ESI+) calculated for $C_{93}H_{168}ClO_{21}Si_4N_4$ $[M+NH_4]^+$: 1824.0942, found 1824.0967

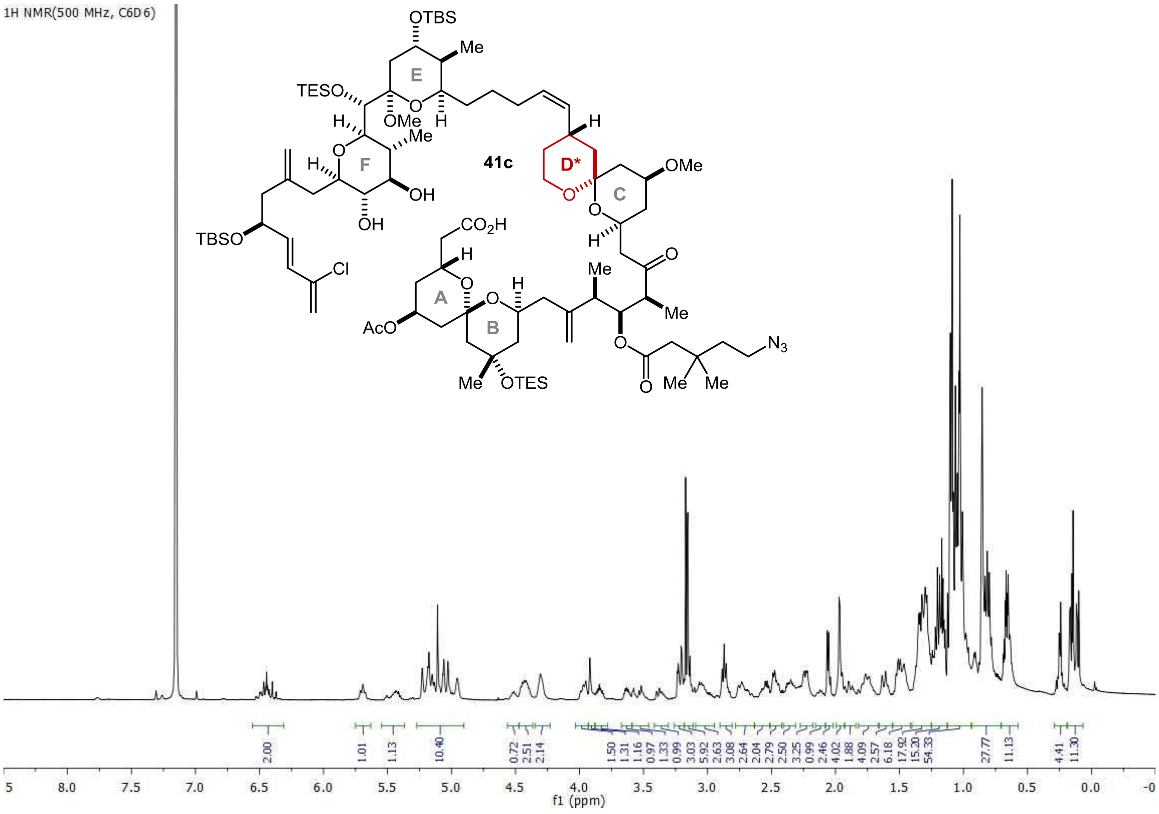

Supplementary Figure 149. ^{1}H NMR of **41c**.

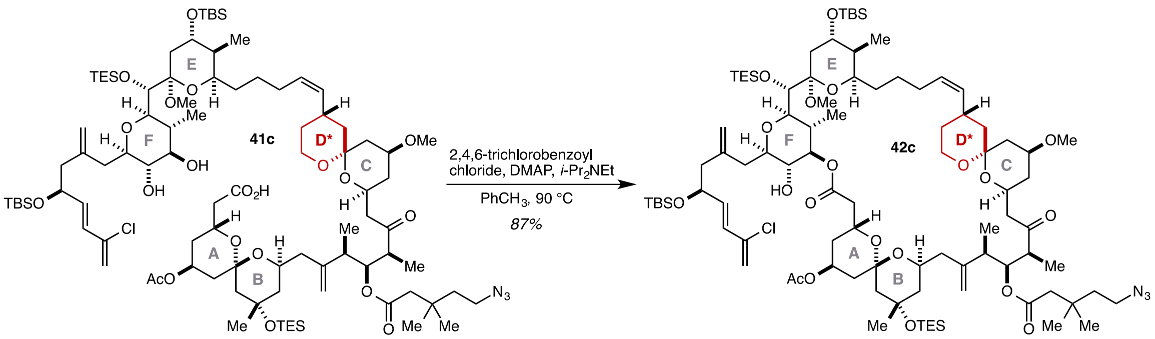

Supplementary Figure 150. Synthesis of **42c**.

Seco-acid **41c** (10 mg, 0.0058 mmol) was dissolved in toluene (0.58 mL) and *i*-Pr_2NEt (61 μL, 0.35 mmol) and 2,4,6-trichlorobenzoyl chloride (18 μL, 0.12 mmol) were added. This was stirred for 4 h at rt, then diluted with toluene (1.74 mL). The mixture was taken up into a 6 mL gas-tight syringe and added to a 90 ºC solution of DMAP (35 mg, 0.29 mmol) in toluene (8.5 mL) over 24 h by syringe pump. The vial containing starting material which was sealed and stored at 0 ºC was rinsed with toluene (0.56 mL) and added to the reaction at over 8 h by syringe pump. The starting material vial was rinsed with an additional portion of toluene (0.56 mL) and added to the reaction over 4 h by syringe pump. The reaction mixture was allowed to stir at 90 ºC for 20 h. The reaction mixture was cooled to rt and diluted with Et_2O (12 mL). This was washed with $NaHCO_3$ (sat. aq) (12 mL) and brine (12 mL). The combined aqueous layers were extracted with EtOAc (2 x 12 mL). The combined organic layers were dried over Na_2SO_4, filtered and concentrated. Purification by flash column chromatography (10% → 40% EtOAc/Hexanes) on silica gel afforded lactone **42c** as a white solid (9.1 mg, 0.005 mmol, 87% yield).

OR $[\alpha]_D^{25}$ +13.7° (*c* 1.00, CH_2Cl_2)

IR (ATR) 2925, 2875, 2854, 2094, 1735, 1647, 1579, 1550, 1461, 1371, 1249, 1109, 892, 835, 775, 741 cm^{-1}

^{1}H NMR (500 MHz, C_6D_6) δ 6.52 – 6.29 (m, 2H), 5.72 (t, *J* = 14.0 Hz, 1H), 5.45 – 5.33 (m, 1H), 5.26 – 4.96 (m, 7H), 4.82 (q, *J* = 9.8 Hz, 1H), 4.59 – 4.47 (m, 1H), 4.41 (d, *J* = 29.6 Hz, 2H), 3.96 (d, *J* = 4.3 Hz, 1H), 3.88 (d, *J* = 22.7 Hz, 2H), 3.72 – 3.60 (m, 1H), 3.54 (dd, *J* = 22.4, 12.7 Hz, 2H), 3.45 (d, *J* = 3.1 Hz, 1H), 3.21 – 3.11 (m, 4H), 3.09 (q, *J* = 9.1, 8.2 Hz, 2H), 2.99 (s, 1H), 2.94 – 2.80 (m, 2H), 2.69 (d, *J* = 22.6 Hz, 1H), 2.63 – 2.45 (m, 3H), 2.44 – 2.28 (m, 2H), 2.28 – 2.18 (m, 2H), 2.17 – 2.07 (m, 2H), 2.07 – 1.94 (m, 4H), 1.80 – 1.44 (m, 8H), 1.42 – 1.23 (m, 29H), 1.17 (tt, *J* = 7.8, 5.2 Hz, 9H), 1.14 – 0.71 (m, 62H), 0.63 (dqd, *J* = 19.5, 7.9, 4.8 Hz, 8H), 0.28 (d, *J* = 2.9 Hz, 3H), 0.20 – 0.03 (m, 10H)

^{13}C NMR: We were unable to generate a useful ^{13}C NMR spectrum for this compound due to a lack of a minimally sufficient amount of material.

HRMS (ESI+) calculated for $C_{93}H_{166}ClO_{20}Si_4N_4$ $[M+NH_4]^+$: 1806.0861, found 1806.0861

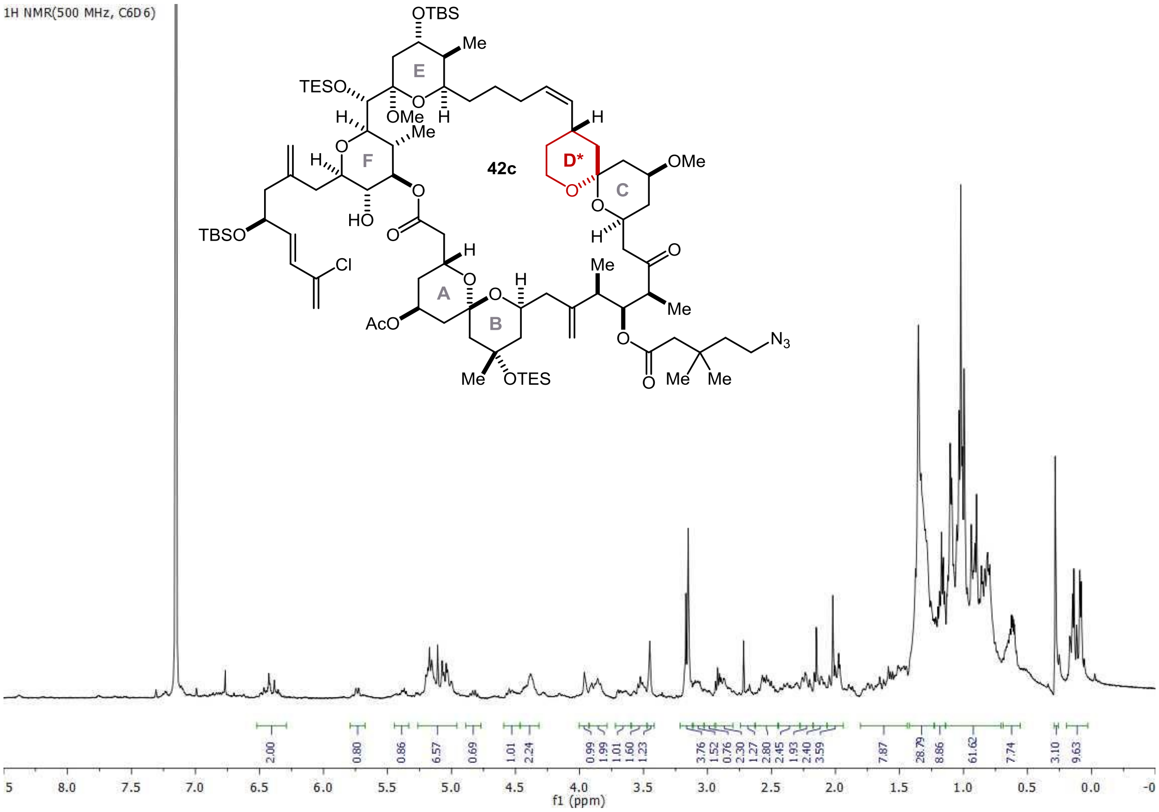

Supplementary Figure 151. ^{1}H NMR of **42c**.

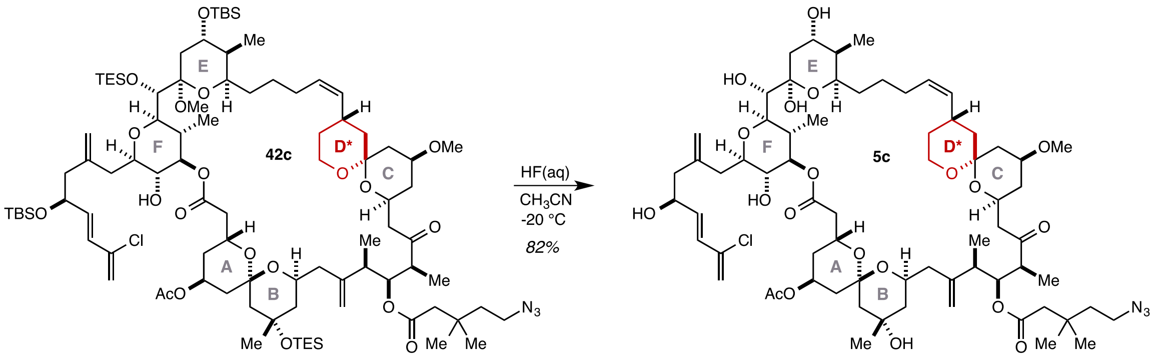

Supplementary Figure 152. Synthesis of **5c**.

Lactone **42c** (6.8 mg, 0.0038 mmol) was dissolved in MeCN (0.27 mL) and cooled to -20 ºC. A freshly prepared solution of HF in MeCN (0.27 mL) [prepared by mixing 48% aqueous HF (1.25 mL) and MeCN (5 mL)] was added over 2 h by syringe pump at -20 ºC. The reaction was allowed to stir an additional 18 h at this temperature. The reaction was quenched by the dropwise addition of Et_3N (0.64 mL) and warmed to rt. The reaction mixture was diluted with a 2:1 mixture of EtOAc/CH_2Cl_2 (4 mL), then washed with $NaHCO_3$ (sat. aq) (4 mL) and brine (4 mL). The combined aqueous layers were extracted with a 2:1 mixture of EtOAc/CH_2Cl_2 (2 x 4 mL). The combined organic layers were dried over Na_2SO_4, filtered and concentrated. Purification by flash column chromatography (0% → 5% MeOH/CH_2Cl_2) on silica gel afforded spongistatin 1 analog **5c** as a white solid (4.1 mg, 0.0031 mmol, 82% yield).

OR $[\alpha]_D^{22}$ +18.2° (*c* 0.50, MeOH)

IR (ATR) 3435, 2926, 2851, 1735, 1653, 1580, 1551, 1436, 1384, 1233, 1177, 1093, 1059, 894 cm^{-1}

^{1}H NMR (400 MHz, CD_3CN) δ 6.46 (dd, *J* = 14.9, 7.1 Hz, 1H), 6.16 (dd, *J* = 15.0, 5.6 Hz, 1H), 5.49 (s, 1H), 5.39 (s, 1H), 5.30 (dd, *J* = 17.8, 8.7 Hz, 1H), 5.25 – 5.14 (m, 1H), 4.98 – 4.89 (m, 3H), 4.87 (s, 1H), 4.81 – 4.69 (m, 1H), 4.37 (ddd, *J* = 35.8, 27.1, 14.7 Hz, 3H), 4.21 – 4.11 (m, 2H), 4.08 – 3.98 (m, 1H), 3.83 – 3.75 (m, 1H), 3.71 (s, 1H), 3.66 – 3.52 (m, 2H), 3.42 (dd, *J* = 19.2, 9.6 Hz, 1H), 3.28 (s, 3H), 3.21 – 3.09 (m, 2H), 3.07 – 2.94 (m, 1H), 2.92 – 2.82 (m, 2H), 2.78 (d, *J* = 15.2 Hz, 1H), 2.67 – 2.46 (m, 2H), 2.45 – 2.35 (m, 1H), 2.35 – 2.23 (m, 2H), 2.14 (s, 24H), 2.07 – 2.00 (m, 2H), 1.95 (s, 31H), 1.84 (s, 2H), 1.71 (dd, *J* = 15.1, 4.3 Hz, 3H), 1.65 – 1.46 (m, 9H), 1.37 (d, *J* = 2.1 Hz, 3H), 1.18 (dd, *J* = 6.9, 2.8 Hz, 4H), 1.12 – 1.01 (m, 8H), 0.96 (s, 7H), 0.94 – 0.88 (m, 5H), 0.83 (dd, *J* = 16.3, 6.9 Hz, 5H)

^{13}C NMR: We were unable to generate a useful ^{13}C NMR spectrum for this compound due to a lack of a minimally sufficient amount of material.

HRMS (ESI+) calculated for $C_{68}H_{108}ClO_{20}N_4$ $[M+NH_4]^+$: 1335.7245, found 1335.7234

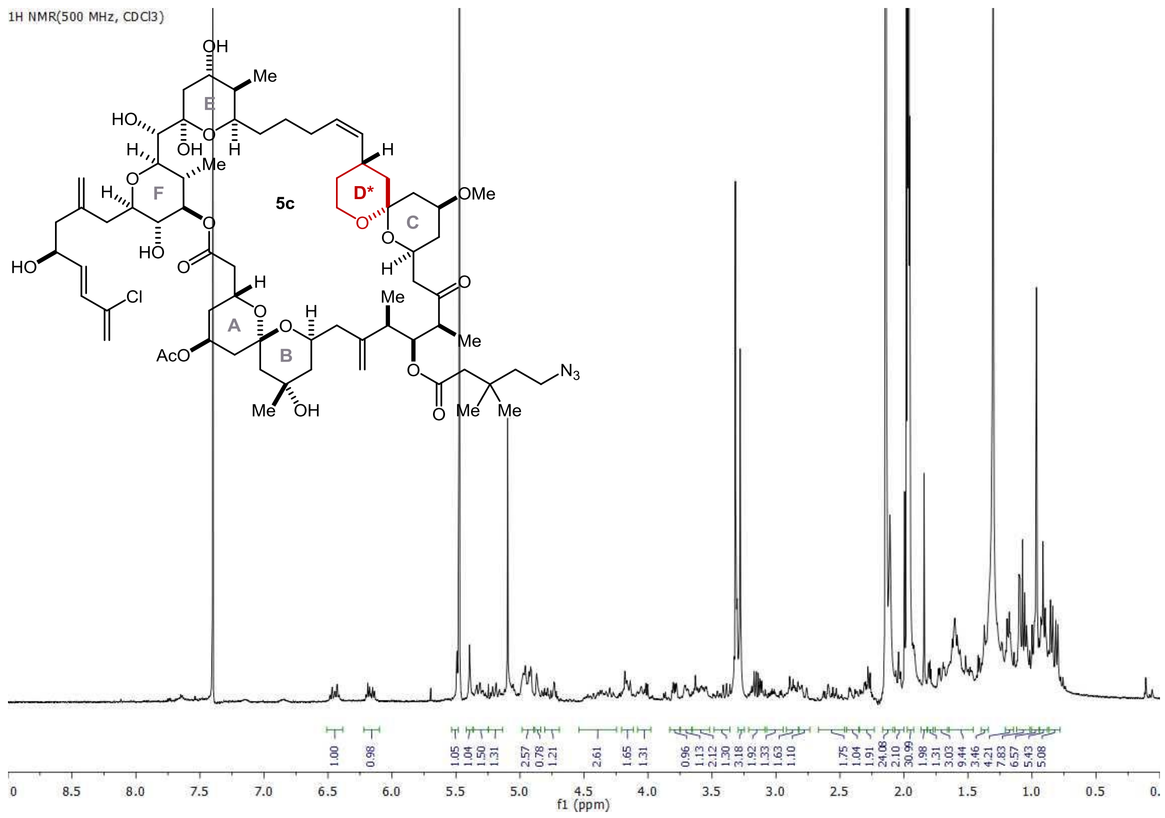

Supplementary Figure 153. ^{1}H NMR of **5c**.

In Vitro Cell Growth Inhibition Assays

Assay protocols

PC3, human prostate cancer, and Ca46, Burkitt's lymphoma, cell lines were obtained from the NCI anticancer drug screen. A2780(1A9) cells, human ovarian cancer, were the kind gift of Dr. Tito Fojo, Medical Oncology Branch, NCI, NIH. All cells were maintained in RPMI medium supplemented with 10% fetal bovine serum. Growth inhibition was determined by the resazurin method, following exposure to serial dilutions of each compound for 4 days prior to assay and GI(50) determinations as described.[19] Each compound was tested with an initial 10-dose dilution series, followed by narrower, replicate 6-dose series, each of which was a separate experiment. The results presented are mean values based on multiple determinations of the GI(50), where the number of replicates, n, is shown. The standard deviation was determined from the replicate determinations of GI(50).

Supplementary Table 1: Full details for assay results reported in Fig. 5b

Supplementary Table 1. *In vitro* cell growth inhibition data for **5a-c**.

Cmpd	PC3 (prostate)			A2780(1A9) (ovarian)			Ca46 (lymphoma)			U937 (lymphoma)		
	GI_{50} nM	s.d.[a]	n[b]	GI_{50} nM	s.d.[a]	n[b]	GI_{50} nM	s.d.[a]	n[b]	GI_{50} nM	s.d.[a]	n[b]
**5a**	0.1	0.03	9	0.06	0.02	6	0.15	0.09	9	0.1	0.04	3
**5b**	2.5	0.5	9	1.4	0.8	6	2.7	1.1	9	2.4	0.06	3
**5c**	0.3	0.09	7	0.06	0.08	5	0.5	0.06	8	0.3	0.07	3
paclit axel	2.9	0.9	8	1.1	1.1	6	3	1.4	9	1.2	0.5	3

[a]s.d. = standard deviation. [b]n = number of repeats.

Supplementary References

1. Chen, L.-A.; Ashley, M. A.; Leighton, J. L. Evolution of an Efficient and Scalable Nine Step (Longest Linear Sequence) Synthesis of Zincophorin Methyl Ester. *J. Am. Chem. Soc*. **139**, 4568-4573 (2017).
2. Myers, A. G.; Yang, B. H.; Chen, H.; McKinstry, L.; Kopecky, D. J.; Gleason, J. L. Pseudoephedrine as a Practical Chiral Auxiliary for the Synthesis of Highly Enantiomerically Enriched Carboxylic Acids, Alcohols, Aldehydes, and Ketones. *J. Am. Chem. Soc.* **119**, 6496-6511 (1997).
3. Michel, B. W.; Camelio, A. M.; Cornell, C. N.; Sigman, M. S. A General and Efficient Catalyst System for a Wacker-Type Oxidation Using TBHP as the Terminal Oxidant: Application to Classically Challenging Substrates. *J. Am. Chem. Soc.* **131**, 6076-6077 (2009).
4. Yu, Z.; Eno, M. S.; Annis, A. H.; Morken, J. P. Enantioselective Hydroformylation of 1-Alkenes with Commercial Ph-BPE Ligand. *Org. Lett.* **17**, 3264-3267 (2015).
5. Suen, L. M.; Steigerwald, M. L.; Leighton, J. L. A New and More Powerfully Activating Diamine for Practical and Scalable Enantioselective Aldehyde Crotylsilylation Reactions. *Chem. Sci*. **4**, 2413-2417 (2013).
6. Tekle-Smith, M. A.; Williamson, K. S.; Hughes, I. F.; Leighton, J. L. Direct, Mild, and General *n*-Bu_4NBr-Catalyzed Aldehyde Allylsilylation with Allyl Chlorides. Tekle-Smith, M. A.; Williamson, K. S.; Hughes, I. F.; Leighton, J. L. *Org. Lett.* **19**, 6024-6027 (2017).
7. Cha, J. Y.; Yeoman, J. T. S.; Reisman, S. E. A Concise Total Synthesis of (−)-Maoecrystal Z. *J. Am. Chem. Soc.* **133**, 14964-14967 (2011).
8. Cordero, F.; Pisaneschi, F.; Gensini, M.; Goti, A.; Brandi, A. Stereodivergent Approach to Enantiopure Hydroxyindolizidines Through 1,3-Dipolar Cycloaddition of 3-Hydroxypyrroline *N*-Oxide Derivatives. *Eur. J. Org. Chem.* **2002**, 1941-1951 (2002).
9. Schleicher, K. D.; Jamison, T. F. A reductive coupling strategy towards ripostatin A. *Beilstein J. Org. Chem.* **9**, 1533-1550 (2013).
10. Reznik, S. K.; Leighton, J. L. Toward a more step-economical and scalable synthesis of spongistatin 1 to facilitate cancer drug development efforts. *Chem. Sci.* **4**, 1497-1501 (2013).
11. Kubota, K.; Leighton, J. L. A Highly Practical and Enantioselective Reagent for the Allylation of Aldehydes. *Angew. Chem. Int. Ed.* **42**, 946-948 (2003).
12. Röhrig, C. H.; Loch, C.; Guan, J.-Y.; Siegal, G.; Overhand, M. Fragment-Based Synthesis and SAR of Modified FKBP Ligands: Influence of Different Linking on Binding Affinity. *ChemMedChem* **2**, 1054-1070 (2007).
13. Tanis, P. S.; Infantine, J. R.; Leighton, J. L. Exploiting Pseudo C_2-Symmetry for an Efficient Synthesis of the F-Ring of the Spongistatins. *Org. Lett.* **15**, 5464-5467 (2013).
14. Yamada, K.; Fujita, H.; Kunishima, M. A Novel Acid-Catalyzed *O*-Benzylating Reagent with the Smallest Unit of Imidate Structure. *Org. Lett.* **14**, 5026-5029 (2012).
15. Weigand, S.; Brückner, R. Building Blocks for the Stereocontrolled Synthesis of 1,3-Diols of Various Configurations. *Synlett* 225-228 (1997).
16. Crimmins, M. T.; Katz, J. D.; McAtee, L. C.; Tabet, E. A.; Kirinich, S. J. An Aldol Approach to the Synthesis of the EF Fragment of Spongistatin 1. *Org. Lett.* **3**, 949-952 (2001).
17. Fernandez-Megia, E.; Gourlaouen, N.; Ley, S. V.; Rowlands, G. J. Studies Towards the Synthesis of the C29-C51 Fragment of Altohyrtin A. *Synlett* 991-994 (1998).
18. Smith, III, A. B.; Sfouggatakis, C.; Risatti, C. A.; Sperry, J. B.; Zhu, W.; Doughty, V. A.; Tomioka, T.; Gotchev, D. B.; Bennett, C. S.; Sakamoto, S.; Atasoylu, O.; Shirakami, S.; Bauer, D.; Takeuchi, M.; Koyanagi, J.; Sakamoto, Y. Spongipyran Synthetic Studies. Evolution of a Scalable Total Synthesis of (+)-Spongistatin 1. *Tetrahedron* **65**, 6489-6509 (2009).
19. Präbst, K.; Engelhardt, H.; Ringgeler, S.; Hübner, H. Basic Colorimetric Proliferation Assays: MTT, WST, and Resazurin. *Methods Mol. Biol.* **1601**, 1-17 (2017).
